# Posters

**DOI:** 10.1155/1992/26384

**Published:** 1992


### V030

### IMPROVEMENT OF SURGICAL TREATMENT OF HYDATID DISEASE OF THE LIVER

XU MING-QIAN
Research unit of hydatidology of people's hospital of Xinjiang Autonomous Region, Urumqi, 830001 P.R. China

There were 1,640 patients with hydatid disease who underwent surgical treatment in our hospital during 1953-1990. of these, 1,204 cases were liver hydatid disease, and 1,319 operations yielded 1,716 Echinococcus cysts. The methods of removal of hydatid cyst were: 1) hepatic lobectomy 2) removal of intact hydatid cyst 3) removal of hydatid cyst by aspiration.

Removal of the cysts left in the liver residual ectocyst cavities as large as the cysts and bile-containing bloody retention cyst, usually resulted in secondary infection, causing liver abscess. The clinical observations and experimental studies of this series revealed the mechanism of intrahepatic biliary fistula formation and the natural rule of cavity closure, allowing rational cavity management and modification of operative methods. This reduced the frequency of postoperative biliary fistula and of secondary infection, and also shortened the healing period.

### V031

### HYDATID DISEASE OF THE LIVER: CISTOPERICISTECTOMY

PROF. V. ROVATI, DR, E. FALESCHINI, DR. G. NERVETTI, DR. G. TAGLIABUE, DR. F, COLTURANI.
Istituto Scienze Biomediche L. Sacco - Catt. Patologia Chirurgica - Universita di Milano - Italia

The video shows a case of hydatidosis of the left hepatic lobe, appearing on the surface of the parenchyma. Cisto-pericistectomy was performed. This is the treatment of choice, because it doesn't cause reduction of the functional parenchyma and has better possibilities for radical.

### P001

### NEONATAL BILE DUCT PERFORATION AND STRICTURE: TWO OF A KIND?

W.A. BEMELMAN, H.A. HEY, A. VOS and M.N. van der HEYDE
Departments of Paediatric Surgery and Surgery, Academic Medical Center, University of Amsterdam, 1105 AZ Amsterdam

Benign bile duct strictures are occasionally seen in early infancy. Usually, no cause can be identified and the stricture is said to be "congenital". Spontaneous bile duct perforation is a rare entity which occurs in the first 12 weeks after birth. This disease may present as a subacute illness with jaundice, acholic stools, failure to thrive and progressive biliary ascites. A pseudocyst may be found in the hepatoduodenal ligament at laparotomy. Both spontaneous perforation and the stricture of the duct tend to be located just proximal of the junction of common bile duct and cystic duct. We have analyzed our patient material in order to asses whether infants treated for a neonatal bile stricture could have had a bile duct perforation prior to the clinical manifestation of the stricture. Three cases were found. Two of them had a Roux-en-Y hepaticojejunostomy for a bile duct stricture located at the junction of the common bile duct and the cystic duct at an age of three and 20 months respectively. One infant had a transient period of moderate jaundice associated with dilated intrahepatic ducts at an age of one week. The other child had a period of melaena and haematemesis presumably caused by haemobilia two months after birth. In both cases the symptoms resolved spontaneously. The third infant had an explorative laparotomy for extrahepatic cholestasis five weeks after birth. A pseudocyst filled with bile was found which was located in the hepatoduodenal ligament. The cyst was drained. The perforation site could not be identified. Two weeks after surgery, a biliary fluid collection was drained percutaneously. These cases suggest that spontaneous bile duct perforation might pass unobserved when located in the hepatoduodenal ligament, and might proceed to a bile duct stricture.

### P002

### RESULTS OF RECONSTRUCTIVE PROCEDURES FOR BILE DUCT INJURIES AND BENIGN STRUCTURE

COLOVIC R., SAVIC M., MILICEVIC M. AND BILANOVIC D.
Institute for Digestive Diseases, University Clinical Center, Belgrade 11000, Yugoslavia, Koste Todorovica br. 6.

The aim of this retrospective study is to evaluate the long-term results of reconstructive procedures for bile duct injuries and benign strictures. A total of 86 pts. were analyzed, 57 women (66.3%) and 29 men (33.7%). Two pts. had congenital bile duct stricture, 6 had stricture due to chronic pancreatitis and 78 pts. had bile duct injuries. One bile duct injury was due to an abdominal penetrating wound and 77 were iatrogenic (12 "acute" injuries and 65 presenting as postoperative benign strictures). In 66 pts. bile duct injury occurred during cholecystectomy, in 9 during gastrectomy and in 2 during operation for liver hydatid disease. Two pts. had disinsertion of the papilla of Vateri and 75 pts. had section or resection of the bile duct. Unsuccessful previous reconstructive procedures were done in 52 pts. 1 to 6 times. According to the Bismuth classification 25 pts. (29.0%) were type I, 25 pts. (29.0%) were type II, 23 pts. (26.9%) were type III, 12 pts. (14.0%) were type IV and 1 pt. (1.16%) was type V. Six pts. had intrahepatic bile duct stricture and 25 pts. had intrahepatic stones. Reconstruction of the previously done Roux-en-Y jejunal limb was necessary, since it was found to be too short, in the majority of pts.

The reconstructive procedures in the 12 pts. with "acute" injuries were: 1 implantation of the papilla into the duodenal stump and 1 into a Roux-en-Y loop, 8 end-to-end anastomosis over a "T" tube brought out through a separate bile duct incision. Reconstructive procedures in the 74 pts. with postoperative benign strictures were: 75 cm long Roux-en-Y hepaticojejunostomy in 67 pts., choledochoduodenostomy in 3 pts., choledochoplasty in 1 pt. and resection of stricture with end-to-end anastomosis in 2 pts. Reconstruction was impossible in 1 pt.

Overall operative mortality was 1.2%. The pt. with portal vein thrombosis in whom reconstruction was technically impossible, died 4 months after operation due to uncontrollable variceal bleeding. Two pts. died three years after successful reconstruction due to unrelated causes. All remaining pts. are alive and entered into a follow-up protocol. The average follow-up period was 5 years (median 4.2 years). According to Blumgart's criteria good results were achieved in 76 pts. (89.4%), satisfactory in 6 pts. (7.0%) and unsatisfactory results in 3 pts. (3.5%). One pt. with unsatisfactory results was reoperated and later entered into the "good results" group. Factors influencing outcome were the level of stricture, portal hypertension, time from injury to definitive operation, number of unsuccessful previous attempts at reconstruction and the hypertrophy/atrophy complex. The authors conclude that the majority of "acute" bile duct lesions and benign bile duct strictures can be managed successfully.

### P003

### SURGICAL PROCEDURES IN PATIENTS WITH MIRIZZI SYNDROME. A CRITICAL REVIEW

A. COGLIANDOLO, G. GIACOBBE, M.A. GIOFFRE, F.M. GULINO, B. MICALI
General Surgery, University of Messina, Italy

Mirizzi Syndrome (MS) indicates a clinical picture of obstructive jaundice by a stone impacted in the cystic duct and/or the surrounding inflammation of the common bile duct. Its incidence has been reported from 0.5 to 1.3% of patients undergoing surgery for benign biliary disease (Tulassay; Csendes). The goal of this study was to examine the value of surgical options in the treatment of the syndrome.

Among 1880 patients operated on for gallstones we found 6 MS (0.3%). There were 4 males and 2 females (mean age 63 years). According to Csendes classification (slightly modified), 3 patients (50%) were a type I lesion, 1 a type II (17%) and 2 a type III (33%). In type I patients, jaundice was caused by gallstone compression in 1 patient (type Ia) and by inflammatory reaction in the remaining 2 (type Ib). A correct diagnosis was preoperatively established in 4 patients (66.6%) by endoscopic cholangiography. In all patients a cholecystectomy was performed. In 4 cases (2 type I, 1 type II and 1 type III) a choledocostomy with T-tube was performed. In 2 patients (type I and III respectively) a bilioenteric anastomosis was constructed. No mortality or major complications were observed. In 1 patient type III operated on choledocostomy, bile drainage was more productive than 3 lts/day to require a reinstillation of biliary juice. T-tube was removed on 20th postoperative day without complications.

In conclusion, MS is a rare complication of gallstones disease requiring an appropriate surgical treatment which must be calibrated on the type of lesions and local conditions.

TULASSAY et al.: Acta Endoscopica 17 (2): 61-65, 1987.

CSENDES et al.: Br. J. Surg. 76 (11): 1139-1143, 1989.

### P004

### MANAGEMENT OF IATROGENIC INJURIES TO BILIARY DUCTAL SYSTEMS

L.C. JIMENEZ, E. MORENO, J. BERCEDO, C. LOINAZ, M.A. GARCIA, J. SEOANE, J. IBAÑEZ, I. GONZALEZ-PINTO
Hospital 12 de Octubre. Service of General and Digestive Surgery "C". Madrid, Spain

The bile duct injuries usually occurs during cholecystectomy, being related to variations in the anatomy, traction, bleeding, etc.

We operated upon 60 patients (pts) because biliary iatrogenic injuries. Nine were diagnosed during operation, eight in the postoperative period and 43 later in the follow-up (strictures).

The location of the early injuries was: complete transection of common hepatic duct in 7, complete of common duct in 5, partial of common duct in 3, complete of common hepatic duct in 1 and desinsertion of papilla in 1. The location of the strictures was: common hepatic duct in 13, common duct in 12, hepaticojejunostomy in 9, hepatico duodenostomy in 4, choledocho-choledochostomy in 3, choledocho (cyst)-duodenostomy in 1 and cholecysto-duodenostomy in 1. The repair techniques were: cholangio-jejuno-duodenostomy in 36, Roux-en-Y central cholangio-jejunostomy in 11 and peripheral in 1, total sphincterotomy in 5, choledochoduodenostomy in 2, suture and T-tube in 2, choledochotomy and T-tube in 1 and choledocho-choledochostomy over T-tube in 1.

The postoperative mortality was 5.9% in the early diagnosed injuries and 6.9% in the late one (strictures). The postoperative morbidity was 17.6% in the early diagnosed group and 4.6% in the late one. In the follow-up we studied 14 pts of the early injuries, 11(78.5%) being well and 37 of the late one, 33(89.2%) being well.

We conclude that the postoperative mortality was similar in early and late diagnosed biliary injuries, being the postoperative morbidity and long-term results better in the late diagnosed injuries.

### P005

### TRANSDUODENAL SPHINCTEROPLASTY AND TRANSAMPULLARY SEPTECTOMY-INDICATIONS AND RESULTS

S.B. KELLY, A. YONG, B.J. ROWLANDS
Department of Surgery, Institute of Clinical Science, Royal Victoria Hospital, Grosvenor Road, Belfast, BT12 6BJ. Northern Ireland

Thirteen patients received transduodenal sphincteroplasty and transampullary septectomy between 1987 and 1991. All patients had severe pain for greater than 2 years which was refractory to medical therapy. There were no serious complications. Mean age was 46 years (range 20-60 years) and sex distribution was 8M:5F. Mean hospital stay was 14 days (range 8-45 days). Six patients had chronic pain unrelieved by cholecystectomy. At follow-up (mean 3.2 years) pain is much improved or abolished in 5 of 6 patients. Four patients had chronic pancreatitis. At follow-up (mean 2.1 years) pain is much improved or abolished in 3 of 4 patients. Two patients had recurrent pancreatitis. At follow-up (mean 2.2 years) pain is much improved or abolished in 1 of 2 patients One patient was found to have papillary stenosis when undergoing cholecystectomy. Four years later pain is much improved. Pancreas divisum was present in 3 patients; 1 chronic pancreatitis, 1 recurrent acute pancreatitis and 1 postcholecystectomy pain. Accessory sphincteroplasty was performed in the two patients with pancreatitis. Transduodenal sphincteroplasty and transampullary septectomy can relieve pain in patients with postcholecystectomy pain and recurrent pancreatitis, presumably by improving drainage of the obstructed ducts. Careful patient selection maximises the chance of a successful outcome.

### P006

### IATROGENIC LESIONS OF BILE DUCTS IN CHOLECYSTECTOMIES BETWEEN 1980 AND 1989

M. KOZELJ, V. FLIS, S. POTRC
Maribor Teaching Hospital, Maribor, Slovenia

In 10 years 6646 cholecystectomies were done (1927 men, 4179 women, average 52.6 years, range 14-91). In 8 cases (6 women, 2 men, average 62 years, range 28-75) iatrogenic lesion of the bile duct occured. In 7 cases elective, in one case urgent operation was carried out. 7 lesions were detected and treated in the case of surgery, once the lesion was overlooked. This patient was reoperated 6 months later for choledochus stricture. 6 cases were operated by senior surgeon (with more than 500 performed cholecystectomies) (incidence 0.1% or 1 lesion per 974 operations) and 2 by residents (up to 25 performed cholecystectomies) (incidence 0.2% or 1 lesion per 398 operations). 6 times operation was done antero- and twice retrogradely). All lesions occured before intraoperative cholangiography was carried out. Bile duct was injured 4 times (twice transsection, twice partial lesion), the common hepatic duct once (complete transsection), right hepatic duct twice (partial lesion) and left once (longitudinal lesion). No patient was adipose and no other technical problems during operation were reported. In partial lesion direct suture was applied once and 3 times suture over the T tube. In complete transsection termino-terminal reconstruction over the T tube was performed. In patient with overlooked lesion after 6 months stricture developed. She was reoperated and biliodigestive anastomosis after Roux was performed. In two female patients reoperations were required after primary reconstruction over T tube. In both cases finally biliodigestive anastomosis after Roux was done. All patients are now without complaints (follow up between 12 months and 5 years). Authors concluded that iatrogenic bile duct lesion is a serious complication that should be reconstructed by experienced surgeon only.

**P007**

### HUMAN PERICARD FOR RECONSTRUCTION OF BENIGN IATROGENIC COMMON BILE DUCT (CBD) STRICTURES IN THE PIG

G. LEXER, G. MEISER, H. KAINDL, H.W. WACLAWICZEK, O. BOECKL, W. PIMP
1st Surg. Dept. and L. Boltzmann-Inst. for Exp. Surg., Landeskrankenanstalten Salzburg, Austria

Extrahepatic benign biliary tract strictures are usually iatrogenic. In an attempt to avoid bilioenteric anastomosis various biologic and artificial materials have been employed without satisfactory results. The purpose of this study was to determine whether human pericard could be used as a CBD substitute in exp.bile duct repair. Material and methods: In 8 pigs (20-25kg) a midline laparotomy was performed under general anesthesia and a subtotal stenosis produced over a 1 mm thick needle. Pre- and postop. once a week cholestasis parameters were evaluated and CBD diameters measured by sonography. 3 weeks later the CBD obstruction was replaced by a human pericard patch plastic using 7,0 Prolene single sutures, without endoluminal stenting. Another 6 weeks later the CBD was dissected and a duodenotomy performed for intra-op. retrograde cholangiography. Finally the CBD was excised for histology and electrone microscopy.

Results: CBD diameters in mm, x SD preop.: 4,5±0,54; 3 weeks after OP 1:14, 0±3,59; 6 weeks after OP 2:9,0±1,68. Bilirubin levels in mg/dl x SD preop.: 0,375±0,09; 3 weeks after OP 1:0,5±0,27; 6 weeks after OP 2:0,325±0,05.

Morphology: In all cases the pericard patch was overgrown by immature biliary epithelium, no fistula or stenosis was observed within 6 weeks. Conclusion: In the exp. model of bile duct stricture that has been presented, bile duct injuries were repaired using human pericard as a free graft which proved to be an acceptable material for reconstruction of benign common bile duct strictures.

**P008**

### TREATMENT OF IATROGENIC INJURY OF THE BILE DUCT

T.J. LIU, C.C. WU, C.J. HUANG, R.Y. HUANG, M.D. YANG, T.C. WU, F.K. P'ENG
Division of General Surgery, Dept. of Surgery, Taichung Veterans General Hospital, Taichung, Taiwan, R.O.C.

Unrecognized iatrogenic injury of extrahepatic bile duct usually causes obstructive jaundice, repeat cholangitis and subsequent liver cirrhosis and failure. The experiences of treatment in 19 patients with iatrogenic injury of bile duct were reviewed. The original operations which caused bile duct injury were cholecystectomy in 15, cholecystectomy with choledochotomy in 3, suture ligation of bleeding duodenal ulcer in 1. In 5 patients injuries were recognized during cholecystectomy and end to end anastomosis of the bile duct was carried out in 2 and Roux-en-Y hepaticojejunostomy was carried out in 3. For the other 14 patients, injury of the bile duct was found in 8-105 days after operation. The operations of bile duct reconstruction of these patients were Roux-en-Y hepaticojejunostomy in 8, Longmire's left hepaticojejunostomy in 2, hepaticoduodenostomy in two gastrectomized patients and ductoplasty with T-tube stenting in 2. Percutaneous biliary drainage is necessary for relieving obstructive jaundice in 12 of these patients. One patient (5.3%) died after reconstruction of the bile duct. Of the 18 patients survived after operation, two (11.1%) had stenosis of bilioenteric anastomosis (average 52.1 months, range 8-99 months) after reconstruction. Both of them had multiple operations (>4) on their bile duct before our operation. However, no operative management with per cutaneous balloon dilatation of the stenotic site was effective without requiring re-operation.

Meticulous bile duct dissection and anastomosis are mandatory in reconstruction for iatrogenic bile duct injury and percutaneous balloon dilatation is suggested when stenosis of the bile duct occurs after reconstruction.

**P009**

### CHOLEDOCODUODENOSTOMY FOR BENIGN BILIARY DISEASES IN OLD AGE

DR. NOCE ROBERTO; DR. GALUPPI GAETANO; DR. ABBONDATI ANTONIO; DR. STEINWEG MICHELE; PROF, DR. ERNESTO NICORA
IV Divisione Chirurgica Ospedale "S. Martino" Genova, Italy.
Head: Prof. Ernesto Nicora

In the last ten years we performed 54 side-to-side CDS in patients of 70 years or more for benign biliary diseases 44 (81.4%) underwent for cholelitiasis associated with choledocus dilatation by a papillary stenosis, 6(11.1%) for stenotic cholangitis of the terminal choledocus, 4(7.5%) for choledochostenosis by a chronic pancreatitis. The age of the patients ranged from 70 to 95.29(53.7%) were women and 25(46.3%) men. Only one patient died of cardiac failure on the fifth day after operation. Some complications occurred in 9 patients: 3 bad biliary fistula (only in one of them we were compelled to reoperate the patient in order to perform another bilio-intestinal anastomosis); 1 had severe metabolic troubles following a severe diabetes; 3 had bronchopneumonia medically cured. The jaundice disappeared after 3-10 days after operation in almost all the patients, and the hepatic enzymes brought back to normal as well. Only one patient showed a bad cholangitis after a few months, not easily controlled. So, we can assert:

1) CDS can be performed when ERCP was unsuccessful or impossible especially in old patients

2) CDS is an easy and quick operation with an acceptable rate of mortality and morbidity

3) CDS isn't matched with a high rate of post operative cholangitis.

**P010**

### DIAGNOSIS AND TREATMENT OF BENIGN BILE DUCT STRICTURES

T.SUWA, S.HIRAKATA, K.HAYASHIDA, and F.KIMURA
Surgical Department, Omiya Red Cross Hospital, Yono City, Saitama, 330, Japan

The purpose of the study: We investigated usefulness of pecutaneous transhepatic cholangiocatheterization (PTCC) by diagnosing and treating of 41 cases through examining the kinds of bacterias contained in bile juice and dilation extent of the bile duct.

Method and material: Drainage of bile juice and cholangiography were performed under PTCC in 41 patients with 16 males and 25 females whose average age was 54 and ages rainged from 19 to 81. Stricture cases of the bile duct proper were 27 (papillitis and primary sclerosing cholangitis etc.). 4 stricture cases were caused by the surrounding organs diseased (tumour forming pancreatitis etc.) and 10 cases were done by operation and/or trauma.

Results: Bacterial culture became positive with 29 cases (66%). Measurements of diameter of the intrahepatic bile duct were conducted during PTCC and extent of dilations were examined based upon total bilirubin level of less than 3.0mg/dl and more than 3.1mg/dl. In the former case, 6 out of 13 cases showed heavy dilation of the intrahepatic bile duct with 10mm or more in diameter and in the latter case, 6 out of 25 cases showed slight dilation of 4.9mm in diameter. Radical operations were performed in 19 out of 41 cases. Remaining 22 patients were treated by means of drainage through an indwelling catheter. In 8 cases stricture of the bile duct was dilated by a bougie and the patients were cured with no complication.

Conclusion: PTCC is useful to mitigate jaundice and cholangitis caused by benign bile duct stricture and also to treat the stricture itself.

### P011

### UNUSUAL CASES OF BENIGN STRICTURE OF THE BILIARY TRACT

H.TAKEHARA, N.KOMI, M.NISHI, A.OKADA, Y.MIYOSHI, K.MASAMUNE
First Department of Surgery, School of Medicine
The University of Tokushima, Tokushima, 770 Japan

Three patients, one child and 2 adults, with unusual benign stricture of the extra biliary duct are reported.

First case is a 3 year-old boy complicated with biliary obstruction from ischemic change of the biliary ducts due to devascularization of the extrahepatic duct at 10-month after extended right hepatectomy for mesenchymal hamartoma of the liver.

Second is a 18 year-old male with obstructive jaundice due to compression of the common bile duct caused by enlarged collateral varicose veins, so-called cavernous transformation following extrahepatic obstruction of the portal vein.

Third is a 63 year-old male with obstructive jaundice caused by sclerotic vascular ring of the right hepatic artery.

The definitive diagnosis was obtained by some imaging evaluations and laparotomy. They have been released from jaundice by surgical interventions. These causations are uncommon, especially the first case illustrate a heretofore unrecorded entity of biliary obstruction from ischemic change of the bile ducts following resection for tumour.

### P012

### SURGICAL THERAPY OF IATROGENIC LESIONS OF THE COMMON BILE DUCT

K.RADEBOLD, J.R. SIEWERT
Dept. of Surgery, Technical University of Munich, Germany

The high rate of late complications indicates that iatrogenic lesions of the common bile duct are a high surgical challenge for well experienced surgeons.

Patients: From July 1st 1982 to Sept. 30th 1991, we operated on 24 patients (16 women and 8 men, mean age 45.1 years) with iatrogenic lesions of the common bile duct. The location of the injury was 2x the right hepatic duct, 3x the confluens region, 11x the middle part and 8x the distal part of the choledochus.

Diagnosis was established by ERC and ultrasonography.

Surgical therapy: Intraop, detected lesions (16 patients, 66%) were sutered (4x), a completed disrupted bile duct was operated by end-to-end anastomosis (5x), hepato-jejunostomy Y-Roux (5x) and choledochoduodenostomy (once). 8 late detected lesions were treated by suturing (3x) and biliodigestive anastomosis (5x).

Results: Post-operative complications resulted in 11 patients (45.8%): 2x postoperative bile fistula, 2x early cholangitis, 1x early restenosis followed by reoperation. Late complications were: 2x chronic cholangitis, 5x restenosis followed by bouginage (3x) and reoperation (2x), in one case after six years.

Conclusion: Even after early successful reconstruction patients remain at a high risk for late complication, like chronic cholangitis with biliaric cirrhosis and restonosis followed by reoperation.

### P013

### THE SURGICAL MANAGEMENT OF BENIGN STRICTURE OF THE BILE DUCT COMPLICATING WITH CHOLELITHIASIS

LIANG-FANG XIA
Dept. of Surgery, Affiliated Hospital of Guiyang Medical College, Guizhou Province P. R. China

From 1973–1986, 443 cases of primary cholelithiasis had been operated in our hospital. Among them 146 (32.96%) were complicated with bile duct strictures. There were 243 strictures altogether. The locations of stricture were as follows. 1 Left lobe: LHD 50m 20ms 1mi 18 ls 13 li 22. 2 Right lobe: RHD 41 as 1 ai 15 ps 1 pi 4. 54 patients had lesions in the intrahepatic and common bile duct (B 26 CHD 28 DCD 3). The diagnosis was made mainly by PTC, ERCP, B-US and CT in the preoperative period, by cholangiography, selective segmental cholangiography and/or surgical exploration during operation, by cholangiography through a T or U tube post-operatively. 87 patients were treated with plastic incision on the high positioned strictures combined with choledoenterostomy, 11 cases combined with support by T tube and choledoenterostomy. 12 cases of right lobe lesions were managed by plastic incision and U tube support. 20 patients were treated by partial lobectomy plus T tube drainage. 16 cases were deal with dilatation of the high positioned bile duct plus U tube support. The mortality was 2%. The results of follow-up were: Excellent 68.7%, Good 19.8%.

### P014

### TREATMENT FOR KLATSKIN-TUMORS

ST. ARENS, P. DECKER, A. HIRNER
Bonn University, Surgical Department, Sigmund-Freud-Str. 25, 5300 Bonn 1, Germay

In our department 17 patients, 9 female and 8 male, suffering from Klatskin-tumors have been treated during the period from 1.2.89 to 1.10. 91. The mean age was 63,8 years.

In stage I ($T_1N_0M_0$) was one patient, in stage II ($T_2N_0M_0$) two patients, in stage III ($T_{1,2}N_1M_0$) one patient, in stage IVa ($T_3N_xM_0$) 12 patients and in stage IVb ($T_4N_xM_1$) one patient.

Klatskin-tumors often are locally resectable. In about 20–50% a part of the liver needs to be resected (4 pat.=23.5%),either as a hilar-limited resected (1 pat. =6%) or as an extented right or left hemihepatectomy (3 pat. =17.5%). In 10 – 20% the large vessels have to be resected and reconstructed.

The rate of resection for hilar cholangiocarcinoma of 23.5% in our group is very close to the average of 30%, described in the literature.

The mortality rate was 0%.

In spite of the bad prognosis, resection of the tumor and reconstruction of continuity seems to offer the best therapy and all patients suffering from Klatskin-tumors have to be approached with assessment for resection in mind.

### P015

### RADIATION ASSOCIATED MALIGNANCIES OF THE BILIARY TRACT

C.W. BIERMANN; G. FRöSCHLE; R. SCHWARZ; U. MEYER-PANNWITT; CH.E. BROELSCH
Department of Surgery and Radiotherapy; The University of Hamburg, Germany

Malignancies of the biliary tract are rare diseases. Tumor induction after radiotherapy are reported in several papers. We report about three cases with consecutive biliary tract carcinoma 18-32 years after manifestation of an urogenital carcinoma. All three patients ( 52–63 years) were treated with additive radiotherapy after surgical-urological therapy. Icterus was the top rancing symptom of this secondary carcinoma. In two of the three cases surgical tumor resection was performed (biliary duct resection with central liver resection; pancreatico-duodenectomy). Because of the bad general condition-of health the third patient only got a biopsy of the tumor. All patients died within two years after diagnosis. The accidental accumulation of the rare biliary tract carcinoma after urogenital malignancies in our collections of clinical cases (two seminoma; one bladder tumor) give rise the question about the causal associatio between primary carcinoma, additive therapy and the development of secondary carcinoma.

### P016

### EPIDEMIOLOGIC AND CLINICAL DATA OF PATIENTS WITH CARCINOMA OF THE GALLBLADDER: HYSTOTYPE, BILE ANALYSIS, STONE COMPOSITION ASSOCIATED FACTORS AND RISK FACTORS

F.CETTA, F.LOMBARDO, V.GARGANO
Institute of Surgical Pathology, University of Siena, Italy

Clinical data and stone and bile analysis in 17 consecutive patients with gallbladder carcinoma (GBC) who had a complete surgical removal of their gallbladder, alone or together with a minor or major liver resection, were related to histological findings.

Patients with adenocarcinoma (ADC) (n=13) had large cholesterol or combination stones in 11 cases, black mud in 1 case and combination stones with black mud in one case. Bile culture was positive in 25% of cases, pancreaticobiliary reflux (PBR) was evident in 2 of 7 cases.

Squamous cell carcinoma (SQC) was found in 4 cases. It was always associated with large (> 1.5-2 cm) long lasting cholesterol stones. Bile culture was negative. Evident PBR was not detectable.

On the basis of present data and of the literature review concerning gallbladder carcinoma and premalignant lesions or conditions it is suggested that ADC (in particular papillary ADC) and SQC seem to be associated with possible different "pathogenetic" factors. ADC, the most frequent histologic type, is sometimes associated with PBR, but less frequently with gallstones.

On the contrary, SQC is more closely related to long lasting cholesterol stones and to risk factors affecting their formation (female sex, obesity, multiple pregnancy, dietary factors).

### P017

### NUTRITIONAL & METABOLIC ALTERATIONS, DIETARY HABITS AND ASSOCIATED GALLSTONES IN THE PATHOGENESIS OF BILE TRACT CANCER

CETTA F, LOMBARDO F, *JEPPSSON B, **DE ALMEIDA A
Institute of Surgical Pathology, University of Siena Italy, *Department of Surgery Lund University, Sweden, **Surgical Clinics University of Lisbon, Portugal

The design of the study has been to show differences in the incidence of gallstones (and of various gallstone types) and bile tract carcinoma in 4 different European countries: a Mediterranean country as Italy, an Atlantic country as Portugal and two Northern countries as Germany and Sweden. It has been hypothesized that Mediterranean countries with a higher intake of vegetables, olive oil and carbohydrates could have a different incidence of both gallstones and related cancer as compared to countries of North Europe, with a higher intake of butter, animal fats, smoked meat or fish. All patients who are scheluded for bile tract surgery will be enrolled in one major hospital centre per country. They will have systematic bile and stone analysis, in addition to histology of the specimen. Preliminary studies performed in Italy on consecutive surgical patients with bile tract diseases, who had bile, stones and gallbladder (or bile tract) wall prospectively examined have shown that different types of gallstones are associated with gallbladder carcinoma (GBC) and periampullary carcinoma (PC). In particular, GBC is associated with gallstones in more than 95% of cases and stones are large long lasting cholesterol stones. On the contrary, PC is associated with gallstones in only 33% of cases and stones are almost always pigment stones (a peculiar type of pigment stones, that are concomitantly black and brown). Concerning gallbladder carcinoma, in a series of 1200 consecutive surgical cases with gallstones, 8% of the patients older than 50 years and with gallstones larger than 1.5cm had a GBC. On the basis of these preliminary results, it has been hypothesized that; 1) cholesterol, black and brown pigment stones are related to different factors and are expression of physicochemical, bacteriological and nutritional conditions that are different for the various types of gallstones; 2) Patients older than 50, with large long lasting cholesterol stones are at higher risk of developing gallbladder carcinoma; 3) On the contrary, patients with large cholesterol gallstones in the gallbladder have a very low risk of developing PC.

### P018

### PROXIMAL EXTRAHEPATIC BILE DUCT TUMORS: EXPERIENCE WITH 23 CASES

I.DIAZ, V.TONINI, J.BALSELLS, J.L.LAZARO, R.CHARCO, E.MURIO, C.MARGARIT
Liver Trasplantation Unit, Department of Surgery, Hospital General Vall d'Hebrón, Universidad Autónoma, Barcelona, Spain

Twenty-three patients with proximal extrahepatic bile duct tumors (EBDT) were treated at our institution between 1975 and 1990. There were 17 males (73.9%) and 6 females (26%). The age ranged from 35-86 years (mean age 62 years). At presentation, all patients were jaundiced and three had cholangitis. Diagnosis was established by ultrasonography, ERCP or PTC, and CT-scan. Localization of the tumour at the bile duct bifurcation was: type I 21.7%, type II 43.5% and type III 34.8% (Bismuth-Corlette classification). Complete resection of all macroscopic tumour was accomplished in 8 patients (34.8%, Group I). The final histology report revealed microscopic evidence of tumour at the proximal resection margin in 6 of these 8 patients. In the remaining 15 patients the cancer was in advanced stage, with involvement of surrounding structures or distant metastasis. Those patients underwent palliative procedures: Group II was submitted to palliative resection (5 patients, 21.7%), and in Group III (10 patients, 43.3%) intrahepatic cholangiojejunostomy was performed in 3 patients and transtumoral intubation was carried out in 7 patients. The 30-day hospital mortality rate was none in Group I, 40% in Group II, and 57.1% in Group III. The morbidity rate was of 25% in Group I, 80% in Group II, and 60% in Group III. Significant difference in mean survival time was observed in Group I as compared with the two other Groups (30.4 months versus 6 months). Long term survival in patients with EBDT is rarely achieved even in those cases (35-50%) in which a complete resection of the tumour can be performed. Local recurrence is the usual cause of death, the tumour spreads along the periductal vessels and may infiltrate the intrahepatic ducts over a wide distance, the retroduodenal tissues of the hilar area and the hepatoduodenal ligament. EBDT should be considered as regional rather than local affections, therefore total hepatectomy with complete resection of the biliary system and its lymphatic drainage tract by means of a Whipple procedure followed by liver trasplantation may offer a more radical approach and provide a better survival time or a definitive cure in a selected group of patients.

### P019

### UPPER CBD TUMORS: RESECTABILITY CRITERIA

G.M.GAZZANIGA, M.FILAURO, C.BAGAROLO, E.CIFERRI
1st Department of Surgery - S.Martino Hospital, Genoa, Italy

Tumors origing of biliary confluence are rarely treated with a curative resection; this is due to the advanced endo- and extrabiliary diffusion of the neoplasm at the diagnosis. On a total number of 100 primitive hepatic hilum tumors observed from 1970 to 1990, 55 (55%) cases underwent to surgical approach; the remaining 45 (45%) were treated with endoscopic or percutaneous palliative methods, or suffered no treatment at all. 26 (47.3%) of operated patients had "curative" resection of the tumor, 17 (30.9%) "palliative" resection, 12 (21.8%) a simple biliary decompression with various methods. Long-term results are significantly better for resective surgery (curative + palliative), compared with biliary decompression (without tumor resection). Criteria adopted to decide the opportunity of a laparotomic approach are mainly: the absence of general contraindication to surgery; the absence of local factors, such as liver metastasis (or massive direct invasion); portal thrombosis (main trunk or bilateral branches), bilateral arterial occlusion, peritoneal dissemination. During laparotomy, tumor resection is adopted when absence of local contraindication is conferred, and a bilio-digestive anastomosis on uninvolved biliary branches is feasible. In conclusion, the "gold standard" of upper CBD tumor treatment is curative resection.

### P020

### DIAGNOSIS AND TREATMENT OF HILAR BILE DUCT CARCINOMA: CLINICAL AND PATHOLOGICAL STUDIES

Z.Q.HUANG, W.H.LEE
Department of Hepato-Biliary Surgery and Pathology
The General Hospital of P.L.A., Beijing, People's Republic of China

Surgically treated cases of hilar bile duct carcinoma were revealed. 50 cases were explored between 1986 and Dec, 1990, resection was carried out in 31 cases, a resectability rate of 62%. In the same period of time, 5 autopsy cases and 27 resected specimens were studied pathologically. In this series, major hepatic resections was done in 14 cases. No operative mortality rate. 6 patients were still living and well for 1-4 years after the operation. Most late deaths died of biliary obstruction and infection as the result of local recurrence of the tumor. The median survival period was 15 months. Pathologically, papillary adenocarcinoma in 6 cases, well differentiated in 21, low differentiated in 3, carcinoma simplex in 2. The causes of death in 5 autopsy cases were due to sepsis instead of tumor metastasis. Since many of the cases were seen late, earlier diagnosis and treatment is needed. We preferred the prolonged use of U-tube drainage after resection.

### P021

### RADICAL SURGERY FOR CARCINOMA OF HEPATIC HILUS

L.C. JIMENEZ, E.MORENO, C.LOINAZ, M.A.GARCIA, J.BERCEDO, P.RICO, I.GARCIA, D.HERNANDEZ
Hospital 12 de Octubre. Service of General and Digestive Surgery "C", Madrid, Spain

The carcinoma of hepatic hilus is slow-growing tumor.

Radical surgery have been performed with curative or palliation purpose.

Between January 1974 and June 1991, we treated 47 patients with bile duct carcinoma, performing a resection in 16 (34%). The mean age was 55.4 years (rang: 27-75). All patients were jaundiced with a mean bilirubin value of 18.8 mg%ml.

The certain preoperative diagnosis was carried out in 7 pts. (P.T.C. and E.R.P.C.), being suspicious in 9 (ultrasonography and C.T.).

The techniques of resection were: 1) Tumor resection without hepatectomy in 7 pts (lymphadenectomy in 5); 2) Tumor resection, lymphadenectomy and left hepatectomy in 5; 3) Tumor resection, lymphadenectomy and right hepatectomy in 2 (1 with added segmental portal resection); 4) Tumor resection, lymphadenectomy and IV segmentectomy in 1 and 5) Liver transplantation in 1.

There was not postoperative mortality. Among postoperative complications we outline: bile leakage in 4 (25%), subhepatic abscess in 1 and right subphrenic abscess in 1. The mean survival of the patients who left the hospital was 26.2 months, being alive 3 at 6.9 and 32 months, respectively. One patient (6.2%) died without recurrence 63 months after operation.

With radical resection the 5-year survival is very low, but we can obtain a good palliation.

### P022

### SURGICAL PROBLEMS FOR MIDDLE AND DISTAL BILE DUCT CANCER

KAYAHARA M, NAGAKAWA T, UENO K, OHTA T, NAKANO T, MORI K, TAKEDA T, AND MIYAZAKI I
Second Department of Surgery, School of Medicine, Kanazawa University, 13-1 Takaramachi, Kanazawa, Japan

Operative problems for middle and distal bile duct cancer were studied from the clinicopathological point of view in an effort to identity those factors which influence the operative procedures. Forty patients were treated by surgical resection for middle and lower bile duct cancer during the 17-year period. Of the forty cases, only nineteen patients underwent curative resection. Three and five-year survival rates for curatively resected cases were 62.7%, and 48.4%, respectively. No patients who underwent noncurative resection survived for 2 years. There was significant difference between two groups. As to the noncurative factor, ew(+): evidence of cancer cells at external cut surface, was most important. For middle bile duct cancer, cancer cells were seen at exposed surface to portal vein. Mode of recurrence revealed that recurrence of choledochojejunostomy was most frequent for middle bile duct cancer (five of six cases), and periaortic or retroperitoneal recurrence for lower bile duct cancer (all cases).

It is concluded that radical resection is necessary to perform the curative operation; pancreatoduodenectomy with combined resection of portal vein including the resection of lymph nodes and nerve plexus is needed except for early carcinoma.

**P023**

### THE EFFECT OF PARTIAL ARTERIALIZATION OF THE PORTAL VEIN ON THE DEARTERIALIZED LIVER

NAKANO T, NAGAKAWA T, UENO K, TAKEDA T, MORI K, NAKANO Y, MAEDA K, KAYAHARA M, OHTA T AND ITSUO MIYAZAKI
Department of Surgery (II), School of Medicine, Kanazawa University, Kanazawa, Japan

For curative operation of the cases of biliary tract carcinoma with hepatic hilar involvement, dissection of the total hepatoduodenal ligament containing hepatic artery and portal vein is required. As a management of preserving liver function during the early hazardous period after dearterialization of the liver, the effect of partial arterialization of the portal vein was studied during 7 days. 15 mongrel dogs were divided into 3 groups: control group, a group in which the collaterals to the liver were dissected and the hepatic artery was ligated (hepatic artery ligated group) and a group in which a femoral artery-portal vein shunt was produced with the urokinase coating catheter with the dissection of the hepatic arterial flow (partial arterialized group). In the partial arterialized group, portal blood was sufficiently oxygenated, and the oxygen demand and supply and tissue metabolism were kept approximately normal. GOT and GPT increased but not statistically. On the other hand, the hepatic artery ligated group showed acidosis in hepatic venous blood, reduction of oxygen supply at 7 days after the operation. GOT and GPT markedly increased and remained high level for 7 days. These results indicate that partial arterialization of the portal vein effectively preserves the liver function on the dearterialized liver in the early period.

**P024**

### THE USE OF WIDE BORE SELF EXPANDING STAINLESS STEEL ENDOPROSTHESIS FOR DRAINAGE OF MALIGNANT OBSTRUCTIVE JAUNDICE (MOJ)

AA POLYDOROU, N HARITOPOULOS, C VAGIANOS, A VEZAKIS, C POTARIS, B GOLEMATIS
Departments of Surgery, Hippocration Hospital, Athens Rion University Hospital, Patrai, Greece

The palliative treatment of MOJ by the insertion of an endoprosthesis (EP) is well established. With this procedure, there are two problems: A. The limited size (10-12 Fr) of the EP that can be used and B, The tube blockage which necessitates replacement of the EP. To address this problem, a new wide bore self expanding stainless steel EP (Wallstent, Medinvent) has been used. This EP in the compressed form has a diameter of 9 Fr and expands to a diameter of 30 Fr when released in a stricture. Of 12 patients with MOJ treated (M/F 9/3, mean age 67, range 47-87), 6 had pancreatic Ca, 4 cholangiocarcinoma and 2 metastatic Ca. All patients had serum bilirubin more than 15 mg/dL. A total of 13 EP were used. Eleven EP were placed endoscopically. There were no serious complications related to the procedure. Three patients died 2, 6 and 12 weeks after EP insertion and the rest are alive 2-34 weeks after drainage. Biliary decompression was successful in all with normal serum bilirubin in all alive patients. One patient with pancreatic Ca required a second EP 5 months later because of tumor overgrowth. This new EP provides a considerably wider lumen for bile duct drainage comparing to currently used plastic EP. Procedural complication rate is not increased. Late blockage seems to be less frequent and EP migration is almost impossible. Further studies with new self expanding WP are warranted.

**P025**

### DIFFERNET SURGICAL APPROACH IN KLATSKIN'S TUMORS

G. GOZZETTI, A. PRINCIPE, A. MAZZIOTTI, G.L. GRAZI, M.L. LUGARESI, F. RUBERTO
Clinica Chirurgica 2°, University of Bologna, Bologna, Italy

The prognosis for patients with Klatskin's tumors is conditioned by the site and extension of the lesion. From 1981 to 1991, 42 pts have been observed. The average age was 60 (32-81) years with a male:female ratio 2:1. The pts selection and the indication for a correct management were assessed on the basis of detailed biliary imaging (Bismuth-Corlette's classification). Liver resection was carried out in 10 pts (resectability: 23.8%). Twenty-three cases (54.7%) had a palliative treatment: 5 external biliary drainage, 7 transtumoral drainage and 11 bilio-digestive anastomosis. In 9 pts (21.4%) no treatments were possible. Of the 10 pts with a radical treatment, 5 are currently alive (mean follow-up 37.8 mo.; range 4-96 mo.) the remaining 5 pts had a mean follow-up of 18 mo. (range 4-42 mo.). Of the 23 pts where a palliative procedure was performed, 5 are still alive (mean follow-up 10.6 mo.; range 2-24 mo.); the mean survival of the 18 pts who have died was 10 mo. (range 1-30 mo.). Best results were obtained in the cases of trans-tumoral drainage. Conclusions: In Klatskin's tumors the prognosis is related to the grading and staging of the lesion. Liver resections have the best long term prognosis with a good quality of life.

**P026**

### STAGING AND TREATMENT OF GALLBLADDER CANCER

A. PRINCIPE, M.L. LUGARESI, F. RUBERTO, G. GOZZETTI
2nd Department of Surgery-University of BOLOGNA

Gallbladder carcinoma (Ca) is characterized by rapid growth and fatal prognosis. Early diagnosis is usually fortuitous and only in I, II, III stage of Nevin, resection surgery have radical results. MATERIAL AND METHODS. During the period 1981-1991 we have observed 50 pts. (33F, 17M;2/1). The average age was 64 yrs (44-91yrs). Diagnosis was carried out as follows: ultrasonography, ERCP, PTC, arteriography, CT scan, i.v. cholangiography. In 41 pts, Ca was at the V stage: in 4 the operation was not carried out; in 12 only explorative laparotomy was performed; in 17, a palliative operation was carried out: in 12 cases with external biliary drainage and in 5 biliodigestive anastomosis was performed. Only in 8 cases was it possible to do a wedge-resection (WR). The remaining, 9 pts I at the II and 8 at the III stage, were treated with a radical removal of the tumour: in 5 cases cholecystectomy was considered to be sufficient, in 4 cases also WR was performed. RESULTS: The average survival rate, after a year on the V stage was 11.4%. 4 pts., on stage II and III are still alive with an average survival rate of 3.7 yrs (range of 1-7 yrs) and 5 have died, 4 for recurrence of the tumour (average survival rate 27.2mo). CONCLUSIONS: early diagnosis of gallbladder Ca, useful for a radical treatment, is rare. Histological testing of the gallbladder removed per lithiasis is therefore the only valid procedure for timely treatment and resective surgery have significant results in the first 3 stages.

### P027

### A CASE OF ADENOSQUAMOUS CARCINOMA OF THE LIVER

H. SASAKI, M. HAYASHI, N. OHHASHI, K. NAKAMURA, K.AMANO
Department of Surgery, Owase Municipal Hospital, Owase City, Mie 519-36, Japan

A case of adenosquamous carcinoma arised from the left hepatic duct is reported with a literature review.

Case: A 65-year-old male was admitted to our hospital because of epigastric discomfort.

Physical examination revealed a 5cm mass in epigastric region. Laboratory studies showed increase of serum Al-p, LAP, and γ-GTP. While CEA and AFP were within normal limits, CA19-9, SPAN-1, DUPAN-2 levels were high.

US and CT scan demonstrated a mass, 5x4cm in size, in the lateral and medial segment of the liver. An ERCP revealed an abnormal tapering at the left hepatic duct. Arteriography demonstrated no tumor stain but encasement around the left hepatic artery. Under the diagnosis of cholangiocellular carcinoma of the left hepatic duct, left hepatic lobectomy was performed. The resected specimen showed a encapsulated tumor, 6x5x6cm in size, with central necrosis associated with several satellite nodules.

Histologically, the tumour was confirmed to be adenosquamous carcinoma.

(Discussion) Adenosquamous cell carcinoma is a very rare tumor of the liver with poor prognosis. Since the original report by Pianzola in 1971, 24 cases were collected through 1991. Clinicopathological features are discussed with the literature review.

### P028

### EXPERIENCE WITH 60 PATIENTS WITH CHOLANGIOCARCINOMA

R.L.V.D. HUL, P.W. PLAISIER, L.S. LAMÉRIS, O.T. TERPSTRA
Departments of General Surgery and Radiology, University Hospital 'Dijkzigt', Rotterdam, The Netherlands

We analyzed 60 patients (25m; 35f) in our hospital who were diagnosed to have a proximal or midductal cholangiocarcinoma in the period of 1980–1990. 56 Patients presented with jaundice, 3 with pain in the upper abdomen and 1 with haemobilia. Most prominent atypical symptoms were loss of weight (n=30) and fatigue (n=20). Most commonly used diagnostic tool was ultrasonography (n=59), which showed dilated intrahepatic bile ducts in 50 and hilar mass in 18 cases. Endoscopic cholangiography (n=48) raised suspicion for malignancy in 46, whereas percutaneous cholangiography (n=31) did this in 30 patients. Localisation of tumor was: bifurcation of common hepatic duct (42), common hepatic duct (13) and midcholedochal (4), 1 case could not be traced. All tumors were adenocarcinomas except for 1 leiomysarcoma. According to the TNM-classification of tumor there were 6 T2- and 39 T3- tumors; of 15 tumors size was unknown. Lymphnode involvement was found in 12, other metastases in another 12 cases. Treatment consisted of: stent placement in 32 followed by local irradiation in 13 cases. Radical resection was performed in 16 with additive irradiation in 6 cases. A bilio-digestive bypass was performed in 8 with additive irradiation in 4 cases. If divided in radical treatment (n=10) and palliation (n=39), mean survival was 108±32wk (SEM) and 39±7wk (SEM), respectively. Of the remaining 10 patients 6 are still alive (4 with radical treatment, 2 with palliation) and 4 were not treated We conclude that early diagnosis followed by radical treatment is essential in this otherwise fatal disease.

### P029

### NON INVASIVE CARCINOMA OF THE GALLBLADDER: A RETROSPECTIVE MULTICENTER STUDY OF 72 CASES

G. ZEITOUN, J.M. HAY
French Association for Surgical research. 8, avenue des Peupliers, 92270 Bois-Colombes, France

The purpose of this study was to analyze the survival of patients treated for carcinoma of the gallbladder at stage I (intramucosal only) or II (involvement of mucosa and muscularis).

Methods: 420 carcinomas of the gallbladder, operated from 1978 to 1985 were analyzed, Among them, 72 were non-invasive: 20 stage I patients, 52 stage II. Symptoms, diagnosis, treatment and survival have been studied in both groups.

Results: There were 16 men (22%) and 56 women (78%).

Results are summarized in table.

| | (%) | GROUP I | GROUP II |
|---|---|---|---|
| **Symptoms** | Past history of gallstones | 24 | 31 |
| | Pain | 71 | 76 |
| | Jaundice | 32 | 30 |
| | Fever | 40 | 36 |
| | Palpable mass | 21 | 18 |
| **Ultrasonography** | Thickenedwall | 32 | 33 |
| **Preoperative diagnosis** | | 5.8 | 5.6 |
| **Treatment** | cholecystectomiy | 80 | 77 |
| | bilioenteric anastomosis | 5 | 9 |
| **Intraoperative diagnosis** | | 39 | 22 |
| **Histology** | adenocarcinomas | 85 | 86.3 |
| | lymph nodes involved | 0 | 8 |
| **Radiation therapy** | | 0 | 9.6 |
| **Survival (actuarial)** | 1 year | 72 | 58 |
| | 4 years | 25 | 19 |

Conclusion: Survival rates show no difference (p<0.01) between the two groups. The poor rate of survival after 4 yrs in group I suggests that more aggressive treatment should be performed (wider resection. adiuvant therapy).

### P030

### PALLIATIVE SURGICAL APPROACHES FOR MALIGNANT EXTRAHEPATIC OBSTRUCTIVE JAUNDICE

KOCOKPINAR T., GONGOR A., HASDEMIR .A.O. ALTUN S. ARAN Y.
S.S.K Ankara Hospital I. Surgery Department Ankara-Turkey

Various operative techniques can be performed for palliation of irrespectable cancers of malignant extrahepatic obstructive jaundice. In our clinic the techniques tended to this palliation are inspected in two major groups; the first is drainage procedures with participation of the ductus choledochus and the second is drainage procedure with the participation of gall bladder (or Ductus cysticus)

I GROUP: Consist of , choledochoduodenostomi (CDS) (31 cases), T-tube drainage (4 cases), Choledochal stent application (1 case), Choledochojejuostomi (2 cases) Hepaticojejunostomy (1 case), and Hepaticoduodenostomy (1 case), (40 cases totally)

II GROUP; consist of ; cholecystojejunostomy (CJS) (24 cases), cholecystoduodenostomy (8 cases), (32 cases totally), (CJS subgroup was continued on with a Braun anastomosis)

In both groups the effectiveness of the drainage procedure was appraised with the measurement of bilirubin, alkaline phosphatase (AP), SGOT, SGPT levels pre and postoperatively. The results are shown at the table below.

| | Hospital. time | T.Bil | D.Bil | AP | SGOT | SGPT | Op. time |
|---|---|---|---|---|---|---|---|
| Preop Group I | | 16.7 | 12 | 60.3 | 86.2 | 114.8 | |
| Group I | | 18.7 | 13.3 | 34.1 | 82.3 | 128.2 | |
| Postop Group I | 12days | 6.6 | 4.7 | 23.7 | 55.6 | 80.2 | 154 |
| Group II | 12 days | 6.2 | 4.4 | 15.3 | 50.6 | 65.6 | 128 |

Reduction ratios of t.he postoperative values are as follows (%)

| | | | | | | | |
|---|---|---|---|---|---|---|---|
| Group I | 0 | 60.5 | 60.9 | 60.7 | 43.5 | 30.2 | 12.9 |
| Group II | 0 | 63.4 | 66.9 | 55.2 | 48.6 | 48.9 | |

Inhospital mortality rate for the GROUP I was 5% (2 cases, myocardial infarction) and it was 9.3% (3 cases, lung edema (1), G.I.S. bleeding (2)) for the second group. Early morbidity rate of GROUP I was 20% and it was 25% for the GROUP II.

The difference between the numerical values of two groups are not to be found meaningful biostatistically (p>0.05). But, from the point view of affectiveness at the postop period. The reduction ratios of the values were significantly meaningful for both off the two groups.

COMMENT: In both groups, there was not a meaningful difference between CDS and CJS from the view of affectivity of the drainage, morbidity rate and mortality rate. Nevertheless, CDS showed a little more superiority to the other kind of surgical techniques.

KEY WORDS: Palliation, Extrahepatic malignancy, Drainage.

### P031

### INTRAPORTAL ULTRASONOGRAPHY: A NEW IMAGING MODALITY OF BILIARY TRACT MALIGNANCY

T. NOGUCHI, F. MARUTA, T. KAKAZU, T. IKEGAMI, Y. HASHIKURA, H. MATSUNAMI, S. KAWASAKI, M. MAKUUCHI
First Department of Surgery, Shinshu University School of Medicine, Matsumoto, Japan

We used a newly devised system for intraportal ultrasonography (IPUS) in order to evaluate tumor invasion into portal venous trunk in patients with biliary tract malignancy.

Methods: The subjects studied were five patients (one with Caroli disease; two with bile duct cancer; two with gallbladder cancer in advanced stage). Ultrasound Imaging System with specially designed Imaging Catheter (CVIS; 20MHz-8F, 30MHz-5F, 4.3F) was used for IPUS. The catheter was inserted through the small mesenteric venous branch and was advanced into the main portal trunk where the IPUS was performed. Obtained pictures from IPUS were compared with portal venography and intraoperative ultrasonography (IOUS) in terms of tumor invasion.

Results: No signs of tumor invasion were demonstrated on IPUS, IOUS, or portal venogram in a patient with Caroli disease. In two patients (one with bile duct cancer and the other with gallbladder cancer), portal venography showed compression of the main portal trunk while IPUS revealed no tumor invasion into the wall of the portal vein. Similar results were obtained using IOUS in these two patients who subsequently underwent extended right lobectomy. Although compression of the portal vein was demonstrated on portal venography similarly in the remaining two patients, abnormal density area of the portal venous wall indicative of tumor invasion was noted on IPUS, which was confirmed by IOUS.

Conclusions: IPUS is useful in detecting tumor invasion into the wall of the portal vein in patients with biliary tract malignancy.

### P032

### LYMPHATIC SPREAD OF GALLBLADDER CANCER: CT EVALUATION

T. OOTANI, Y. SHIRAI, K. TSUKADA, K. YOSHIDA, T. MUTO
Department of Surgery, Niigata University School of Medicine, Niigata, Japan

[Aim] The aim of this study is to evaluate the role of CT in depicting lymphatic spread in gallbladder cancer (GBC) [Patients and methods] The CT findings of lymph nodes in 47 patients with GBC were analyzed. The size of lymph nodes was recorded. The shape was classified into four types: flat, oval, round, lobular. The enhanced pattern was classified into three types: homogeneous, ring-like, macular. All patients underwent radical resection with lymph nodes dissection. Dissected lymph nodes were examined histologically. [Results] 79 lymph nodes were visualized on CT imaging in 47 patients. Metastasis was histologically proven in 48 of the 79 nodes. In 46 (96%) out of 79 nodes, the size measured more than 10mm. In 47 nodes (98%), the shape was oval, round, or lobular. In 47 nodes (98%), the enhanced pattern was ring-like or macular. We decided the following criteria for a diagnosis of positive nodes: a soft tissue mass with (1) size of more than 10mm, (2) oval, round or lobular shape, and (3) ring-like or macular enhanced pattern. This criteria showed sensitivity of 90%, specificity of 91%, accuracy rate of 67% for detection of positive nodes. Our criteria detected metastasis of the cystic nodes in 33%, paricholedochal nodes in 56%, posterior superior pancreatoduodenal nodes in 60% hepatic nodes in 74%, retroportal nodes in 88%, inter-aortico-caval nodes in 90%, superior mesenteric artery nodes in 33%. [Conclusions] (1) The criteria for positive nodes were a soft tissue mass of more than 10mm with oval, round or lobular shape, which changed ring-like or macular. (2) CT scan is a reliable examination for detection of metastasis of the peripancreatic nodes and inter-aortico-caval nodes.

### P033

### ACUTE CHOLANGITIS IN ASSOCIATION WITH ACUTE PANCREATITIS: INCIDENCE, DIAGNOSIS AND PROGNOSIS

A. AGOROGIANNIS, E. NAOUM, IOANNA AGOROGIANNI
Surgical Department, General Hospital of Larisa-Greece

During the last 13 years (from Jan 1978 to Dec 1990) at the Surgery Department of the General Hospital of Larisa, we operated on 2,650 patients with benign diseases of the biliary tract. Of these patients 267 (10.09%) had acute pancreatitis (AP) and 160 (6.04%) had acute cholangitis (ACH). This study is referred to 37 consecutive patients presenting with ACH in association with AP. These patients were operated at the same period of time. Despite the association of common bile duct stones, and material after intrabiliary rupture of hydatid desease of the liver, with ACH and AP, the coexistence of the condition in the same patient is very rare and has not received the proper attention by surgeons. The aim of this study is to examine the incidence of the condition, the clinical and biochemical findings, when the two diseases coexist, and analyse and compare the same parameters in case each diseases appears alone. The incidence of the condition in our patients was 1.4% for gall-stone and hydatid disease cases, 23.1% for the group with ACH and 13.9% for the cases with AP. The features that were measured and compared in our patients were clinical (jaundice, pyrexia, liver tenderness, rigors, shock) and the criteria of Ranson and Imrie. The characteristic operative findings of ACH were choledochitis, redness of the mucosa of the CBD, pus into the CBD and multiple abscesses of the liver.

The results indicate that the association of the two conditions makes the disease more serious, the clinical and biochemical indices are all worse and the mortality is higher (22%). Gallstones or hydatid material are found more often operatively and the operation must be done earlier.

### P034

### ACUTE CHOLANGITIS BY BENIGN CAUSES OF BILIARY OBSTRUCTION: CLINICAL, BIOCHEMICAL AND PATHOLOGICAL CORRELATIONS

A. AGOROGIANNIS, E. NAOUM, I. PRAPPAS
Surgical Department, General Hospital of Larisa-Greece.

The last 13 years (Jan 1978 to Dec 1990) at our Surgical department 2,650 patients underwent operation for benign diseases of the liver and biliary tract. Among them 160 patients were operated for acute cholangitis (6.04%) .58% of these patients were females and the 42% males. The mean age of all the patients was 62 years. All the patients had jaundice, fever, chills and abdominal pain within 72 hours of operations, were defined as having clinical cholangitis and this was confirmed operatively. The purpose of this study is to determine the correlations between microscopic changes in the liver ductural system, the clinical status of the patient, the gross findings of suppuration in the CBD, the clinical postoperative course and the final outcome of the patients. 104 patients (group A) had clinically preoperatively the Charcot's triad (fever > 38°, chills, jaundice and abdominal pain). 56 patients (group B) had clinically the Reynolds five signs (mental confusion and septic collapsous). Preoperative laboratory values included white blood count, serum bilirubine, SGOT, alcaline phosphatase, BUN and creatinine. During the operation liver biopsy was taken and macroscopic findings were obtained. Bile and blood cultures were done. Microabscesses of the liver were found in 5 patients belonging to Group A. Pus into the CBD was found in 47 patients, the 46 belonging to Group A. The data of this study indicate that patients with suppurative and nonsuppurative ACH demonstrate a correlation between liver histology, clincial presentation of the disease and biochemical findings. The more are the clinical and biochemical features, the more severe is the liver inflamation, the longer is the postop course of the patient and worst the outcome.

### P035

### EARLY SURGICAL TREATMENT OF ACUTE CHOLECYSTITIS

P. BACCARI, C. STAUDACHER, P.L. PAESANO**, D.PAROLINI, E. BRAMBILLA, V. DI CARLO*.
Chirurgia d'Urgenza, Pat. Chirurgica* e Radiologia Diagnostica**
IRCCS S. Raffaele, Univ. Milan, Italy

This study was carried out to retrospectively assess the validity of early cholecystectomy for acute cholecystitis. 734 cholecystectomies were performed over a 10-year period in our surgical unit, of which 198 were early cholecystectomies for acute cholecystitis. The average preoperative hospital stay for acute cholecystitis was 2.9 days The overall hospital stay for elective and early surgery was 14.9 days and 11.6 days, respectively. Ultrasonography was carried out at admission in all the cases of acute cholecystitis, with 91.4% of positive results confirmed at operation. Complications of acute cholecystitis (empyema, gangrene, perforation) were found in the 28.7% and calculi of the common bile duct in the 15.1% of the cases. The mortality for early cholecystectomy was 0.5%. No patient submitted to elective cholecystectomy died. There were no significant differences in postoperative complications between early and elective surgery. Patients over 70 years of age who underwent early surgery (24.2%) had more frequent complications of acute cholecystitis ($P<0.05$), choledochal stones ($P<0.001$), and postoperative septic complications ($P<0.05$). In conclusion early surgical treatment is a safe procedure and more cost-effective than the delayed approach. Also elderly patients often necessitate this approach for the more serious complications of acute cholecystitis.

### P036

### DIAGNOSTIC AND MANAGEMENT PECULIARITIES OF CHOLANGITIS IN GERIATRIC PATIENTS

A.P. CHADAEV
Department of General Surgery, 2nd Medical Institute, 1 Ostrovityanova, Moscow, USSR

A review of 251 patients in the old and aged with marked clinical picture of cholangitis was found in 5% cases. The main deciding diagnostic factor in the rest cases was the orderly carrying out of Ultrasound, Endoscopic Retrograde Cholangiography and Laparoscopy. In 23% cases Obstructive Cholangitis was present with bile hipertension which demanded decompression of the bile ducts. As a result of Endoscopic Papillosphincterotomy (EPST) and extraction of gallstones in 80% cases, choledocholithiasis was removed, which made complex management highly effective. In the remaining 11% cases endoscopic correction of bile flow made it possible to avoid biliodigestive anastomosis and operation was restricted to choledochotomy with external drainage of bile ducts. In destructive cholecystitis accompanied with cholangitis (14%) EPST, Laparoscopic transcutaneus, transhepatic puncture, sanitation and drainage of the gallbladder was carried out in first stage of management, which made it possible in high risk patients to carry out radical operations with comfortable conditions for the second stage of operative management. Differential tactics in the management of cholangitis in the old and aged makes it possible to lower the postoperative mortality rate.

### P037

### RECURRENT PYOGENIC CHOLANGITIS - DOES IT EXIST IN YUGOSLAVIA?

R. COLOVIC, M. SAVIC and M. MILICEVIC
Institute for Digestive Diseases, University Clinical Center,
11000 Belgrade, Koste Todorovica Br. 6, Yugoslavia

A retrospective analyses of patients who fulfill the strictest criteria for primary recurrent cholangitis was carried out. Eighteen patients were operated during a 5 year period ranging from 1.01.1986. to 31.12. 1990. Men and women were equally represented. The youngest patient was 30 years old, the oldest 64 the average age being 50. Ten patients were operated earlier 1 to 4 times. Five patients (27.8%) did not have gallbladder stones and another five patients (27.8%) did not have stones in the common bile duct. All 18 patients had intrahepatic stones, 10 pts. (55.8%) in the left lobe, 6 pts. in the right lobe and 2 patients (11.0%) had stones in both lobes. Fourteen patients had recurrent attacks of cholangitis, 2 patients had obstructive jaundice and 2 patients had upper abdominal symptoms at admission. Twenty operations were carried out in 18 patients. One patient had an additional postoperative endoscopic sphincterotomy. In 12 pts. (66.6%) all intrahepatic stones were removed. Left lobectomy (segments II and III) was performed in 7 pts. and hepaticojejunostomy in 7 patients. Two patients had both procedures simultaneously performed.

There was no mortality. All patients have been followed up from 1 to 5 years, average follow up time being 3.5 years. Good results were achieved in 15 patients (83.3%), satisfactory results in 1 pt. (5,5%) and unsatisfactory in 2 pts. (11.0%).

That authors conclude that recurrent pyogenic cholangitis is a rare but present condition in our area and that it can be successfully managed by a combination of operative modalities in the majority of patients with good results. The retention of one or even more stones does not necessarily mean a treatment failure, since stones can pass spontaneously later on.

### P038

### EVOLUTION FO THE DIAGNOSIS AND THE SURGICAL MANAGEMENT IN BILIARY LITHIASIS ON 5500 OPERATIONS

D. MARIAN, L. AZAMFIREI
1st Department of Surgery, Tirgu-Mures, Romania

The authors are presenting a retrospective study on 5500 operations. Two groups of patients operated between 1964 - 1975 (n=1695) and 1981 - 1990 (n=3805) for biliary lithiasis were studied comparatively. The average number of operations raised yearly from 166 to 380. Suppurative acute cholecystitis increased from 8, 90% (151) to 22, 12 (842) to the prejudice of gangrenous and perforation acute cholecystitis. The association with pancreatic lesions increased from 3, 60% (61) to 12, 92% (550). Combined gallbladder and choledocholithiasis raised from 4, 82% (82) to 7, 17% (223) because a better preoperative and operative exploration. Gallbladder carcinoma increased twice from 0, 35% (6) to 0, 60% (23) with an unmodiffied operability rate of 78%. The coincidence of others tumors increased from 2, 48% (42) to 3, 86% (148). The biliary interventions were: simple cholecystectomies decreased from 79, 35% (1355) to 67, 30% (2561); the association with biliary drainage increased from 16, 63% (282) to 20, 34% (774). The interventions on the pancreas increased from 1, 17% (20) to 4, 46% (170). Further associate operations more frequents were : hemicolectomy - 0, 68% (26), gastrectomy - 1, 49% (57%), vagotomy - 0, 78% (36), all for the second group. Cholecystectomies for acalculous cholecystitis decreased from 6, 76% (114) to 4, 94% (188) because of the preoperative exploration and a more efficient conservative treatment. The mortality decreased from 1, 94% (33) to 1, 31% (50) thus: after emergency operations from 8, 60% (16) to 4, 30% (16) and after elective operations from 1, 12% (17) to 0, 99% (34).

### P039

### THE PLACE OF SURGICAL AND ENDOSCOPIC OPTION IN ACUTE OBSTRUCTIVE SUPPURATIVE CHOLANGITIS (AOSC)

G. GIACOBBE, T. MANGANARO, A.COGLIANDOLO, M.A.GIOFFRÉ, B.MICALI
General Surgery, University of Messina, Italy

AOSC is a rare, severe complication of stones, biliary stricture and cystic dilatation of CBD. Despite recent advances, surgery has different morbidity and mortality rates, whereas endoscopic approach seems to be more safe. However, the relative value of primary surgical or endoscopic drainage has not been determined.

A retrospective study concerning the last ten years included 45 patients with specific symptoms of AOSC. Surgery was reserved to 32 patients (13 males, 19 females, mean age 61 years) unresponsive to medical treatment at most 72h from the onset. Lithiasis was the main factor in 26 patients (81%). In 4 (13%) a biliary stricture was documented and in the remaining 2 (6%) an ampulloma and a Mirizzi syndrome, respectively. Surgical operations are shown below.

Cholecystectomy + choledocostomy ..............................10(31.2%)
Cholecystectomy + sphincterotomy................................8 (25.0%)
Cholecystostomy + sphincterotomy................................3 (9.4%)
Cholecystectomy + bilioenteric derivation.....................10(31.2%)
Transtumoral intubation...................................................1 (3.2%)

Mortality was seen in 5/32 (16%), post-surgical complications in 8/32 (25%).

Endoscopic sphincterotomy (ES) and/or associated drainage were applied in 13 patients (7 males, 6 females, mean age 64 years) with AOSC, 9 of whom (69%) had biliary stones and 4 (31%) malignant obstruction. No mortality occurred. Complications were observed in 3/13 (23%).

In conclusion, our results suggest that ES is a safe, effective option to make up the primary decompression of biliary tree in patients with AOSC. Surgery should be employed in patients with poor response to ES and whenever the cause of AOSC has to be removed.

### P040

### AN EVALUATION OF ULTRASONIC GUIDED CHOLECYSTIC PUNCTURE AND SUCTION AS A NEW INITIAL MANAGEMENT FOR ACUTE CHOLECYSTITIS

KITA I[1]., KINAMI Y1, [2]., KOJIMA Y[1]., AKIYAMA T[1], SAITO H[1].,
HAGIWARA H[1]., TAKASHIMA, S[1].
The Second Department of Surgery[1],
Division of Cancer Research, Medical Research Institute[2].
Kanazawa Medical University, Ishikawa, Japan

As an initial management for acute cholecystitis, we have developed the percutaneous transhepatic cholecystic puncture and suction (PTCPS). This study was performed to evaluate the efficacy of PTCPS in the management of acute cholecystitis. Fifty-four patients with acute mild or moderate cholecystitis received this method during the past 4 years from 1986 to 1990. Of them, 29 patients were accompanied with cholecystolithiasis, and 25 patients had no stones. The PTCPS was carried out as follows. Under ultrasonic guidance, the gallbladder was punctured using a 22-gauge needle, and its contents removed by suction. After cholecystography, antibiotics (Amino-glycoside 40 mg) was infused into the gallbladder, and then the needle was removed. The relief of abdominal pain, the removal of fever, the decrease of white blood cell counts, and the improvement of us and/or CT findings were observed within 3 days after PTCPS in the patients obtained the therapeutic effects. Of 29 patients with cholecystolithiasis, 22 patients (76%) revealed availability, 7 patients (24%) indicated no or unclear effect. Of 25 patients with acalculous cholecystitis, 22 patients (88%) showed availability, 3 patients (12%) indicated no or unclear effect. In the latter group, 22 patients recovered from acute cholecystitis. These results suggest that the PTCPS is effective as an initial management for the patients with acute cholecystitis.

### P041

### STUDY OF THE CELLULAR IMMUNITY IN PATIENTS WITH ACUTE CHOLECYSTITIS

L. LORENTE, J.I. ARIAS, A. MILLA, M.P. SUAREZ, J.J. GARCIA, G. GERMAN, M.A. ALLER, J. ARIAS, H. DURAN, J.L. BALIBREA.
Department of Surgery I. Hospital, Universitario San Carlos, Madrid, Spain.

A study was made of cellular immunity in patients with acute lithiasic cholecystitis who underwent delayed cholecystectomy at different moments of the evolution. The results are compared to those obtained in patients in whom elective cholecystectomy was realized for cholelithiasis.

In patients with acute cholecystitits, alterations of the subpopulations of T lymphocytes are associated with an increment of macrophages that do not express Ia antigen (BL2).

The increment of macrophages at the expense of macrophages BL2 demonstrates that there are no quantitative variations in the macrophage subpopulations that express Ia antigens. It is hypothesized that macrophages BL2 constitutes the subpopulation that induces alterations in the lymphocellular subpopulations in patients with acute cholecystitis.

### P042

### DETERMINATION OF COPPER, IRON, ZINC IN SERUM AND URINE IN THE PATIENTS WITH CHRONIC CHOLECYSTITIS AND CHOLELITHIASIS PRE AND POST OPERATION

LI JIN MING, ET AL.
Surgery Dept of Henan Provincial Hosp of Armed Police Forces
Zheng Zhou China 450052

Determination of copper, iron, zinc, copper/zinc in serum and urine in 34 patient with chronic cholecystitis and cholelithiasis as control was carried on with flame atomic absorption method pre and post operation. All cases have been confirmed by B ultrasonid, pathological examination and cholecytectomy. Statistical results show that serum copper is markedly higher in patients of chronic cholecystitis and cholelithiasis pre and post operation ($P<0.001$). Urine copper does not significant difference in patients of pre and post operation ($P>0.05$). Serum zinc and urine zine of pre are lower than post operation ($P<0.001$). Copper/Zinc Ratio in serum is quite different in pre and post operation ($P<0.001$). The serum ferrum and urine in pre is much lower than post operation ($P<0.01$ or $P<0.001$). Our study result shows that abnormity metabolism of copper zinc ferrum in serum and urine of patients with chronic cholecystitis is a dynamic state observed value in treatment. It will be very helpful for diagnosis, treatment and prognosis of chronic cholecystitis with cholelithiasis.

### P043

### ACUTE CHOLECYSTITIS IN AGED PATIENTS

PROUSALIDIS J., FAHADIDIS E., APOSTOLIDIS A., KATSOHIS C., ALETRAS H.
A. Prop. Surg. Clinic. Ar. University, Thessaloniki

The aim of this study is the analysis of the results in 62 patients over 70 years of age with acute cholecystitis treated in our department from 1970 to 1990. The clinical picture in 47 patients was mild and in 15 severe. In 14 cases (10 calculous, 4 acalculous) the acute cholecystitis subsided with antibiotics (Group A). In 48 more cases (45 calculous, 3 acalculous) following 1-3 days conservative treatment, operation was undertaken. Besides acute cholecystitis there was gangrene of gallbladder in 10, choledocholithiasis in 7 and choloperitoneum in 7 cases. Cholecystostomy in 25, cholecystectomy in 15 and cholecystectomy with exploration of the bile duct in 8 cases was performed (Group B). There was one death in group A and 3 deaths in Group B. The hospital stay was 20 days. In conclusion the clinical findings in acute cholecystitis in the aged are usually mild. In the case of failure of medical treatment, after 2-3 days emergency surgery should be performed.

### P044

### URGENT SURGERY IN ACUTE CHOLECYSTITIS

F. SEFERIADIS, A. MYSTAKIDIS, G. SVORONOS, K. KINDYLIDIS, G. VELMACHOS
"Sotiria" General Hospital, Athens, Greece

The management of any patient with the diagnosis of an acute biliary condition, must be argued for the individual case, guided by certain general indications. Indications for surgery during an acute attack of cholecystitis are: a sustained fever beyond 48 hours, signs of peritoneal irritation, features of cholangitis, gerneral peritonitis, presence of heart disease.

450 cholecystectomies were performed over a 6 year period in our surgical department of which 48 were early cholecystectomies for acute cholecystitis, cholecystostomies were carried out in 4.2%. The overall hospital stay was 16.2 days. Ultrasonography was carried out in all cases. Empyema and gangrene were found in 34.2% and calculi of the common bile duct in 14.2% of the cases.

The mortality rate for early cholecystectomy was 0.4%. There were no significant differences in postoperative complications between early and ellective surgery. But if the structures cannot be recognized because of the presence of oedema it is safer to abandon cholecystectomy in favour of cholecystostomy.

Cholecystectomy is clearly ideal provided it is safe. Experts in gallbladder surgery regard cholecystostomy with condescension because they always find it possible to remove the gallbladder during an acute attack. Thus no shame should be experienced in occasionally carrying out cholecystostomy in an emergency.

### P045

### STUDIES OF INFLUENCE FACTOR ON BILIARY EXCRETION OF ANTIMICROBIAL AGENTS

TAKEAKI SHIMIZU, YOSHIAKI TSUCHIYA, OSAMU SATO
Department of Surgery, Shinrakuen Hospital, Niigata, Japan

The faculty of biliary excretion of antimicrobial agents was very important for the therapy to the biliary tract infection. The aim of the present study was to evaluate the influence factor on biliary excretion of antimicrobial agents. Previously, we found the fact with the correlation of bile acid metabolism and biliary excretion of cephalosporin and tetracycline antibiotics such as CFIX, CEZ, CPZ, CMD, CZX and MINO. The biliary excretion of all these antibiotics closely correlated to the total bile acid concentration in bile.

The LFLX, TA167 (new quinolone antimicrobial agents) and meropenem (carbapenem antibiotic) were examined the biliary excretion and bile acid in bile to the 35 patients with external biliary drainage. The concentration of these agents in serum and bile and the bile acid were measured by HPLC method. The meropenem levels in bile were correlated with secondary/primary (S/P) bile acid ($r=0.694571$, $p<0.1$). NY198 concentration in bile was very closely correlated with free bile acid in bile ($r=0.851643$, $p<0.02$). These were not correlated with total bile acid concentration in bile.

Conclusion. In biliary tract infection, total bile acid in bile was decreased and free bile acid, S/P in bile were increased. Therefore, it was considered that the biliary excretion of these antimicrobial agents was an excellent result in the infectious condition.

### P046

### IS THE INVESTIGATION BY ULTRASOUND A RELIABLE METHOD FOR ACUTE CHOLECYSTITIS EARLY DIAGNOSIS?

L. SPAGGIARI, P. CARBOGNANI, P. DELL'ABATE, P. SOLIANI, M. RUSCA, E. CUDAZZO, R. SABBAGH AND E. FOGGI.
Department of General, Thoracic and Vascular Surgery, University of Parma, Parma, Italy

The purpose of this retrospective study was to analyse the data obtained from the use in emergency cases of ultrasonography (US) as an instrumental means of accurately diagnosing acute cholecystitis, helping the surgeon to choose the right moment for cholecystectomy. The period covered by the study was from January 1987 to June 1991; during this time, 94 patients (52 males and 42 females of mean age 65.5 years) with suspected clinical diagnosis of acute cholecystitis, underwent early cholecystectomy (within 72 hours of onset of symptoms) following clinical, laboratory and US evaluation. The US findings considered for the study were: gallbladder (gb) stone/s (A); wall thickening (B); gb distension (C); bile duct stone/s (D); bile duct dilatation (E); gas in the gb (F); echogenic mass shadow about the gb (G); opacities which moved with change in patient's position (H). US examination was performed with the patient in supine position, using 3.5 mHz probes; the same radiologist performed the examination for 88% of the cases. The data, collected and inserted into a computrized card designed specially for the study, were subsequently compared with those from the laboratory and with histopathological results. In all cases histology confirmed the pre-operative diagnosis, and in 19 of them presented a picture of serious cholecystitis (empyema in 4 and gangrene in 15 cases). The US findings most frequently seen together were B+C in 69 cases (73.5%) and A+B+C in 63 cases (67%); if we consider only the serious cases of cholecystitis (19/94-20%) the US finding B+C was seen in 84.2% of the patients. In 15% (14/94) of all cases leucocyte count was less than 8000 w.c./mm3: in 11 (78.6%) of these 14 patients, the US showed A+B+C together. 8 of the 94 patients (8.5%) presented with leucocytosis more than 20000 w.c./mm3; in all cases (100%) the US showed A+B+C together and in 5 cases a fourth US finding was present. The data emerging from our experience lead to several considerations: the US finding most often seen in emergency conditions during acute cholecystitis were B+C (73.5%) and A+B+C (67%); the percentage of association of these findings rose (A+B+C 84.2%) in the more serious cases; US was reliable (A+B+C 78.6%) even in cases where leucocyte counts were silent, confirming the diagnosis in all cases in which the latter was considerably high (A+B+C 100%). Ultrasonography, although dependent upon the observer and therefore giving variable results from one centre to another, is a simple method of early diagnosis, of relatively low cost noninvasive, easily available and with good sensitivity (73.5%) increasing to 84.2% in serious cases. We thus confirm that the emergency diagnosis of acute cholecystitis is still based on evaluation of clinical and instrumental parameters, and that ultrasound is the simplest and most reliable method of diagnosing acute cholecystitis.

**P047**

### PREVENTIVE CHOLECYSTECTOMY FOR ACUTE ACALCULOUS CHOLECYSTITIS (A.A.C.)

P. VRACHNOS, L. LAPIDAKIS, A. KORDONIS, G. HESKETH, L. PAPASTAMATIOU,
Dept. of Surgery, Apostle Paul Hospital, Athens, HELLAS

Many factors have been implicated in the pathogenesis of "post stress" cholecystitis. As the aetiology remains obscure, pronounced biliary stasis, shock, high viscosity of bile, disturbed hormonal regulation following parenteral nutrition, massive transfusions and endotoxaemia, etc., have been suggested to be involved.

Based on a 5 year experience, during which 18 cases of A.A.C. were surgically treated, with a mean time of delay of diagnosis of 2,3, days, early perforation of the gallbladder in 27.8% of the cases and a 22.2% mortality rate was observed (Papastamatiou, 1989). In another 13 cases, postoperative acute cholecystitis was seen in patients with prior cholelithiasis, but it is hard to discern whether or not the stones were the cause of the acute episode.

For the last 18 months, preventive cholecystectomy during the course of emergency laparotomy for blunt abdominal trauma, was applied in all suspected cases. In spite of this strategy, 5 patients, out of 47, were not cholecystectomized and 2 of them developed A.A.C. All patients with cholelithiasis were also subjected to cholecystectomy.

It should be pointed out that A.A.C. is a rare condition with a fulminant course and high mortality. Diagnosis is not easy, as pain is difficult to evaluate in multiple trauma patients, especially in intubated patients; fever and jaundice may be of diverse origin; lab tests are of secondary value and only repeated ultrasonography and diagnostic peritoneal lavage may confirm the first clinical suspicion. It is concluded that the removal of an innocent gallbladder, in the course of an emergency laparotomy, is less dangerous for the patient than allowing the possibility of the occurence of A.A.C.

**P048**

### MRI DIAGNOSIS OF HEMORRHAGIC CHOLECYSTITIS: CLINICAL AND EXPERIMENTAL STUDIES

F. YAMAMOTO[1), YONGLIN PU[2), H.I. GIMI[3)
Yamamoto Hospital, Imari, Japan[1) Beijing Medical, University, China[2) Shionogi Institute, Osaka, Japan[3)

The pathognomonic MRI appearance of hemorrhagic cholecystitis was described in two surgically proven cases (one is acute cholecystitis and another is chronic cholecystitis). The MR imaging was obtained using a 0.5T RESONA unit. Spin echo sequences were used. On T1-weighted image (TR/TE=620/25) and proton-weighted image (TR/TE=1800/40) in the lumen of the gallbladder there is an irregular hyperintense area. On the T2-weighted image, the area was hypointense in its center and hyperintense in its periphery relative to the surrounding bile.

An in-vitro experiment was performed in order to determine what amount of blood can be detected. 0.2 ml of blood in 10ml of bile can be detected as a hyperintense area on T1-weighted image (TR/TE=500/20), and on proton-weighted image (TR/TE=2000/30) 0.4ml of blood in the 10ml of bile can be detected as a slightly hyperintense area. 0.6ml blood mixed with bile cannot be detected at any time in any sequences. If the blood is not mixed with bile there is a significant correlation between the blood/bile signal intensity contrast and amount of blood (0.0-0.6 ml in 10ml bile) on T1-weighted (r=0.966, p=0.0004) and proton-weighted images (r=0.935, p=0.002). If the amount of blood is large, in this study 3.0ml in 10ml of bile, there are two layers in the blood. The signal intensity in the upper layer is higher than that in the lower layer in all the MR images. The above blood/bile signal intensity contrast and signal intensity difference are because of different paramagnetic effects of intracellular and extracellular deoxyhemoglobin and methemoglobin. In the upper layer of large amounts of blood, in the small amounts of blood and in the periphery of the blood in the gallbladder the red blood cell underwent lysis. In the lower layers of large amounts of the blood and in the center of blood in the gallbladder the red blood cell was intact.

**P049**

### ACUTE ACALCOLOUS CHOLECYSTITIS IN EMERGENCY SURGERY

N.V. JARAMOV, I.V. VIACHKI, N.M. PENKOV, D.I. VIACHKI
Centre of Urgent Medikal Aid, Sofia Bulgarian Academi of Medicine, Sofia, Bulgaria

The authors discuss their experience in surgical treatment of 48 patients who were operated on for acute acalcolous cholecystitis/AAC/. Poiting out the multifactorial developement of the disease, they attribute the principle role to the vascular and enzymatic genesis. All patients had had acutely inflamed gallbladders; 40/83, 3%/ had areas of necrosis or gangrene. The mortality rate was 4.1% /2 patients/.

Computed tomographic /CT/ or sonographic evidence of gallbladder wall thickness 4mm, pericholecystitis fluid or subsersal edema without ascites, intramural gas, or a sloughet mucosal membrane was considered diagnostic criteria for AAC.

THE authors emphasize that active surgical Tactics is necessary and that approach to the management of patients with AAC must be differentiated.

**P050**

### SURGICAL TREATMENT OF CHOLEDOCHALCYST

H. FUJIWARA, T. ISHIDA, K. OGINO, T. ICHIHARA, K. WAKITA, M. HAMANO, A. YAMADA, T. ASATSU, K. TSUJIMOTO, T. MITSUNO
Yodogawa Christian Hospital, Osaka, Japan

Choledochalcyst is a rare congenital condition in Western cultures and details of the disease have never been reported. In Japan it seems that choledochalcysts occur more frequently than in other countries. Ten cases of choledochalcysts were observed in this decade (1981-1990) in Yodogawa Christian Hospital.

In ten patients, one male and 9 females, the mean age was 32.5 years (range 8-49 years), and in each one pancreaticobiliary maljunction was noted. While the classic triad of pain, jaundice, and a palpable right upper quadrant mass are pathognomonic, these three were not all present in these ten patients. Eight were admitted for abdominal pain, one for jaundice, and one had no symptom. Later one 43 year old woman was diagnosed with this by ultrasonography in a medical examination.

According to "Todani Classification", five patients had type I cyst, one had II cyst and four had type IV cyst.

All ten patients were treated with cholecystectomy and extrahepatic cystic resection plus hepaticojejunostomy by Roux-en-Y jejunum loop. Through the histological examination it was discovered that a 49 year old woman had a malignant tumor in the gallbladder.

The postoperative course was uneventful except for two patients with mild cholangitis from technical problems and all patients survived.

The purposes of treatment is first to free the patient of all symptoms and second to remove the propensity for malignant degeneration. When there are indication of choledochalcyst we believe that the surgical treatment of choice is extrahepaticcystectomy and Roux-en-Y hepaticojejunostomy for divergence of bile and pancreatic secretion even if the patient has no visible symptom.

### P051

### SURGICAL TREATMENT OF CYSTIC ENLARGEMENT OF EXTRAHEPATIC BILE DUCTS

H.-J. MEYER, CH. SCHMID, B. RINGE, G. GUBERNATIS, J. JÄHNE, R. PICHLMAYR
Klinik für Abdominal - und Transplantationschirurgie, Medizinische Hochschule Hannover, Hannover, Germany

Cystic enlargement of the extrahepatic bile duct system denote rare abnormal findings. Although the pathogenesis is still unknown, congenital origin is still supposed.

We report on our experiences with extrahepatic bile duct cysts treated between 1976 and 1991. Among a total of 33 pts. with various types of cystic lesions 13 pts. were obtained with choledochal cysts. 8 of these pts. showed Alonso-Lej/Todani type I, one pt. each revealed type II and III or could not be categorized whereas two pts. had multiple cysts (type IV). The medical history of 7 pts. revealed cholecystectomy 4 to 22 years ago; predominating clinical feature was recurrent cholangitis in other 7 pts. 12 of 13 pts. were treated by surgical resection of the cystic malformation; in 11 pts. a bilio-jejunal anastomosis with a Roux-en-Y loop was performed and in one pt. an end-to-end anastomosis of the common bile duct was carried out. One pt. died postoperatively caused by septic complications following intraabdominal bleeding. After a mean follow up of 68 months 10pts. are still alive, the other three pts. died, whereas one pt. within two years after surgery with a carcinoma of the bile duct.

Cystic enlargement of an extrahepatic bile duct may develop within all anatomic structures, but no current classification includes all types of cysts described so far. Surgical treatment is always indicated favoring complete resection of the cyst. Postoperative follow up is necessary to detect recurrency as well as malignant transformation.

### P052

### THE CURRENT MANAGEMENT OF BILIARY CYSTS

G. GOZZETTI, A. PRINCIPE, A. MAZZIOTTI, M.L. LUGARESI, F. RUBERTO
Clinica Chirurgica 2°, University of Bologna, Bologna, Italy

Biliary cysts are a rare congenital anomaly occurring in all the biliary tree. The high rate of neoplastic degeneration (3-28% in the extrahepatic cysts; 7-14% in the intra-hepatic cysts; 50% in the cystic-enterostomies) influences surgical treatment, with the preference given to the complete resection, instead of the more comfortable biliary derivation. From October 1981 to November 1991, 15 pts with biliary cysts were observed, 12 female and 3 male, mean age of 36 years (range 6-72 years). Pre-op. work-up consisted in US scan, CT scan, ERCP and PTC. According to Todani's classification, 5 cysts were classified as type I, 5 as type IVa, and 5 as type V. Twelve pts (80%) had a concomitant lithiasis and 6 of them had a previous biliary derivations. All the extrahepatic cysts (type I and IV) were excised and in one case a carcinoma was found on the posterior wall (10%). Three hepatic resections were carried out for segmentary intrahepatic lesions (1 type IVa, 2 type V). The remaining 6 pts had multiple intrahepatic localizations: 5 biliodigestive anastomosis and 1 left hepatectomy, extended at the V and VI segment, were performed in those cases. The patient with neoplastic degeneration died 14 months after surgery. The good results obtained with the others pts (F.U. 6 month - 11 years) demonstrated that total excision, when feasible, is the only and definitive therapeutical choice.

### P053

### 'HANGING' NONCALCULOUS GALLBLADDER

TZARTINOGLOU E., PROUSALIDIS I., APOSTOLIDIS A., KATSOHIS C., ALETRAS H.
A Prop. Surg. clinic, Ar. University, Thessaloniki

The removal of acalculous and not acutely inflamed gallbladder in patients with typical biliary pain remains a questionable procedure. This study was conducted to present our experience in some cases with this problem. In the period 1982-90, 1089 cases of calculous and acalculous gallbladder disease were treated in our clinic. In this period, 27 patients with acalculous, not inflamed, but in abnormal position, with long spiral cystic duct, oblong gallbladder were subjected to cholecystectomy. The mean duration of symptoms, compatible with cholelithiasis, was 5 years. Oral cholecystogram and ultrasonography, provided that other causes of chronic abdominal pain have been excluded, led to the diagnosis. There were 13 lumbar, 9 pelvic and 5 iliac long size gallbladders, with poor function in 20 of them. During cholecystectomy, the organ was invested by peritoneum, suspended in 7 cases from cystic duct and cystic artery, or in 20 cases from a kind of mesentery. On pathological examination, mild chronic inflammation in 19 cases and minimal changes in 8 cases were reported. The minimum follow up was one year and the maximum 9 years. Complete relief of symptoms was achieved in all the cases. In conclusion, cholecystectomy could be tried in these symptomatic 'hanging' gallbladders, probably more readily than is usually done.

### P054

### SURGERY OF BILIARY LITHIASIS IN AN AGED PATIENT. OUR EXPERIENCE ON 238 CASES.

M.V. TATA, P.CRETI, S. MORINI, R. PAGANI
Surgical Department, San Giacomo in Augusta Hospital, Roma, Italy

Biliary lithiasis is a pathology very frequent in the advanced age and represents 20% of our surgical experience on the aged patient. In the time included between 1980 and 1990, in the Surgical Department of San Giacomo Hospital in Augusta of Rome, 238 patients (61 M and 177 F) affected by calcolosis of biliary tracts have been operated of age included between 75 and 93 years, average (79.42). 154 patients 65% (41 M and 113 F) have been operated with 13 deceases (8%) 2 M and 11 F during the hospitalization. 84 patients (35%) 20 M and 64 F have been operated in urgency with 14 deceases (16%) 6 M and 8 F during the hospitalization. 9 patients have undergone a re-operation from the 15th day postoperative to 18 months. In 11 patient an acute pancreatitis was present. The operations executed have been the following: cholecystectomy (230), cholecystostomy (4), choledocholithotomy (22), drainage of main bile duct (48), papillostomy (64), biliodigestive anastomosis (23), close of biliary fistula (10), deletion of infundibulum of the cholecystis (1), drainage of thorax and abdomen (1). In 37 patients an associated pathology was present that has required a contemporary surgical treatment. Surgical therapy of biliary lithiasis presents in the aged patient a risk moderately increased as to the classes of inferior ages. Unfortunately the reluctance to refer an aged patient ot surgical therapy often compels the surgeon to intervene on complex situations and often in urgency, greatly increasing the operative risk.

**P055**

### NONINVASIVE DIAGNOSIS OF HEMANGIOMA OF THE LIVER

ATHLIN L, EKELUND L, BJÖRNEBRINK J.
University of Umeå, Sweden

It is important to separate benign cavernous hemangiomas of the liver from primary and secondary malignant tumors. Ultrasonography (US), computed tomography (CT) and magnetic resonance imaging (MRI) have been reported to be of value in the differential diagnosis. In this study we compared CT and low-field MRI in the diagnosis of hemangioma in the liver in 12 pts with median age 52 years. Hematological and liver function tests, CEA and AFP were within normal limits. MRI, CT and US were performed in all pts and angiography in 3 pts. MR images of the liver were obtained with a low-field resistive magnet operating at 0.02 T or 0.04 T. Prior to i.v. contrast administration all tumors were low attenuated at CT, followed by varying degrees of contrast enhancement. At MRI a considerably increased homogenous signal intensity on T2-weighted images was characteristic - considered more or less as patognomonic. In conclusion we found it possible to demonstrate hemangioma of the liver with MRI - also at ultra-low field strength.

**P056**

### UNUSUAL PRESENTATIONS OF BRUCELLAR LIVER ABSCESS REQUIRING SURGICAL TREATMENT

I. DIAZ, J. BALSELLS, C. MARGARIT
H-B-P. Unit, Department of Surgery, Hospital General Vall d'Hebron. Universidad Autonoma. Barcelona, Spain

Brucellar liver abscess is an exceptional complication of chronic brucellosis, although liver involvement in brucellosis is frequent, as nonspecific or granulomatous hepatits. We present two cases that required surgical treatment. First case: a 48 years old female patient was diagnosed of brucellar liver abscess and treated with percutaneous drainage and antibiotics. One year later a chronic bile fistula remained after the PT drainage. A CT scan showed a persistent small lesion with calcium deposits in the liver. The patient underwent a surgical excision of the fistulous tract and the hepatic granuloma without complications.

Second case: a 33 years old female patient presented with abdominal pain. An abdominal US and CT scan showed a 6 cm low atttenuation lesion with central calcification in the right hepatic lobe. A solid liver tumor involving the right lobe and diaphragm was found at operation. A right hepatectomy with partial excision of the right diaphragm was performed. The patient made and uneventful recovery, The pathology exam showed a tuberculoid liver abscess with caseous necrosis. Cultures and Ziehl exam were negatives. The diagnosis of brucellar hepatic abscess was made because brucella agglutination titer was reported positive. Past history revealed an febrile episode 2 years before that resolved spontaneously, serology test were then negative. Despite medical therapy and percutaneous drainage, most of the reported cases of brucellar abscess had required surgical drainage or resection. Furthermore, sometimes a diagnosis of liver tumor cannot be ruled out and hepatic resection is indicated.

**P057**

### UNRELIABILITY OF THE PRE-OPERATIVE DIAGNOSIS IN CASES OF FOCAL NODULAR HYPERPLASIA

G. GOZZETTI, G.L. GRAZI, E. JOVINE, A. FRENA, A. MAZZIOTTI
Clinica Chirurgica 2°, University of Bologna, Bologna, Italy

The correct diagnosis of benign liver lesions is often difficult on the simply basis of the pre-operative imaging, especially in pts monitored for neoplastic diseases. PATIENTS AND METHODS: We retrospectively reviewed the hospital charts of 14 pts with different pre-operative diagnosis who had a liver resection done and in whom the presence of FNH could be assessed only after the examination of the surgical specimen. Three pts were male and 11 female. The mean age was 41.3 years (range 21-62). All the pts had a pre-op US scan, 12 a CT scan, 8 an hepatic angiography and 4 an MR scan. RESULTS: Two lesions were detected intra-op. during surgery for colo-rectal cancer. FNH was correctly diagnosed in 3 (21.4%) pts at US, in 2 (16.6%) pts at CT scan and in 1 (12.5%) at angiography. No lesions at all were seen in 1 (7.1%) US scan and in 2 (16.6%) CT scans. In the remaining pts uncorrected diagnosis of HCC, hamartoma, metastasis, hydatidosis and cholangiocarcinoma where formulated.

CONCLUSIONS: The large diffusion of the non-invasive imaging revealed a ever more frequent number of hepatic lesions. Nevertheless the pre-op. diagnosis of FNH remains a diagnostic dilemma and FNH is often a post-operative finding. In dubious cases surgical removal the lesion has to be performed.

**P058**

### INDICATION AND RESULTS OF RESECTION IN BENIGN LIVER TUMORS

U. KANIA, J. KALFF, K. WALGENBACH, A. HIRNER
Department of Surgery, Bonn University Medical School, Sigmund-Freud-Str. 25. 5300 Bonn 1, Germany

The indication for a surgical intervention of benign lesions of the liver can be summarized as followed:

Benign adenoma of the liver are an indication for hepatic resection because of their incidence of bleeding complications and the possibility of malignant transformation. Focal nodular hyperplasia should only be operated on for diagnostical reasons or for symptoms caused by extensive tumor size. Scintigraphy (HIDA-Scan) plays a major role as a diagnostical tool for the differential diagnosis of focal nodular hyperplasia. The potential danger of a ruptur of hemangioma of the liver is often overestimated. The incidence is approx. 5%. Surgical treatment is only necessary for cavernous giant hemangioma and hemangioendothelioma in pediatric patients with symptomatic a-v shunting and resulting cardiac insufficiency.

Cautious indication for hepatic resection in benign hepatic tumors led to an operative treatment in 30 of our patients during the last 2 1/2 years. Hospital mortality was 0 %. The morbidity rate was below 10%.

**P059**

### PERCUTANEOUS DRAINAGE OF PYOGENIC HEPATIC ABSCESSES

D.CUZZOCREA, C. FAMULARI.
G. Mazzeo, S. Pante', T. Centorrino, M.T. Fonti, V. Lepore, A. Certo, O. Freni.
Depart. of Surgical Pathology (Chief: D. Cuzzocrea M.D.) Messina University - Italy

The goal of our study aimed to value the efficacy of percutaneous drainage (PD) in the management of patients with single pyogenic hepatic abscesses (SPHA). Between 1989 - 1991, 4 patients, average 52 years with SPHA, all in the right lobe, were treated with PD, by US or CT guidance. In 2 patients was necessary to insert two catheters to get a better drainage of the abscess cavity (AC). The aspirated purulent material was sterile in 3 cases and an association of broad-spectrum antibiotics was administered by systemical and topical way; the bacterial growth was positive (Stafilococcus aureus) in 1 case and a specific antibiotic was given by the same way. No morbidity or mortality is reported. Drainage of the hepatic AC was maintained for an average of 30 days and the average hospital stay was 38 days. Follow up confirmed in all cases the AC disappearance and no disease relapse. We believe that PD of SPHA combined with systemic and topical antibiotic therapy should be the treatment of first choice because this method, characterized by a low morbidity and mortality rate, can avoid to the critically ill patient the surgical stress.

**P060**

### HEPATOCELLULAR ADENOMA: EXPERIENCE IN 29 PATIENTS

A. WEIMANN, B. RINGE, J. KLEMPNAUER, P. LAMESCH, R. PICHLMAYR
Klinik für abdominal- und Transplantationschirurgie Medizinische Hochschule Hannover, Hannover, FRG

INTRODUCTION: In hepatocellular adenoma (HCA) differential diagnosis including focal nodular hyperplasia (FNH) and hepatocellular carcinoma (HCC) may be difficult. Indication for surgery in HCA arises from the tendency for spontaneous bleeding and development of malignancy.

PATIENTS AND RESULTS: From 1980 to October 1991 diagnosis of HCA was established in 29 of 308 patients (pts) with benign liver tumors (9.4%). Female/male ratio was 3:1:1, median age 31 (range 15-58) years. Median tumor diameter was 12 (range 4-20) cm. Use of oral contraceptives was known in 11 women (50%). Upper abdominal symptoms led to diagnosis in 19 pts (65.5%). Tumor bleeding occured in 7 pts (24.1%) requiring emergency laparotomy in 3 pts (10.3%). Prior to surgery, dignity of the tumor remained unknown in 22 pts (75.9%). Resection of the tumor was performed in 22 pts with an operative death of one pt (mortality 4.5%). Liver transplantation was successfully performed in four pts (three with glycogenosis type I). One pt with glycogenosis had undergone previous resection for HCA with incidental HCC. At the time of transplantation the resection specimen revealed multiple HCA without signs of malignancy.

CONCLUSIONS: This study confirms that HCA resection is required to prevent bleeding and development of HCC. According to previously published results our strategy in benign liver tumor is to diagnose hemangioma and FNH by bloodpool- and cholescintigraphy and to operate on all tumors with uncertain dignity because HCA cannot be ruled out.

**P061**

### THE DIFFICULTIES OF AN EXACT DIAGNOSIS OF BENIGN LIVER TUMORS - AND THE SURGICAL CONSEQUENCE

K. RADEBOLD, J.R. SIEWERT
Dept. of Surgery, TECHNICAL UNIVERSITY OF MUNICH, GERMANY

Diagnostic procedures often fail to evaluate the dignity of liver tumors. Operative resection is the consequence to establish an accurate diagnosis. This is of high importance in patients with an underlying malignancy.

Patients: Between 1982 and 1991 we diagnosed 51 benign liver tumors in 17 men (33%) and 34 women (67%) with an average age of 52.7 years. In 13 patients (25.5%) there was a coexisting malignant tumor.

Histology: There were hemangiomas n=31 (M:F = 1:1.3), focular nodular hyperplasias n=13 (M:F=1:3), adenomas n=5, lymphangioma n=1, cholangiofibroma n=1.

Diagnosis: 23 tumors (45%) were discovered with ultrasound in patients with unspecific abdominal pain (of these 14 were resected). 17 tumors (33.3%) were found accidentally during an operation, 5 (10%) in routine diagnostic evaluation before cancer resection (4X operation) and 6 (11.7%) in follow-up studies (2x op.).

Treatment: There was an indication for operative resection in 34 patients (66.6%) and hepatic artery embolisation in one: 10 x radiological confirmation (CT-scan, MR, ultrasonography) could not be obtained, so resection followed. 10 tumors discovered intraoperatively were easily removed; 9 tumors were resected because of coexisting cancer, and in 5 cases patients became symptomatic as an indication for operation.

Resection is the treatment of choice 1. when there is an uncertainty of diagnosis 2. in patients with a coexisting malignancy (to plan further cancer therapy) 3. in symptomatic patients 4. in large adenomas with the risk of malignancy and hemorrhage.

**P062**

### AMOEBOMAS OF LIVER TREATED BY PERCUTANEOUS DRAINAGE - report of two cases

N. IVANIS, M. RUBINIC,
Department of Internal Medicine, Gastroenterology Division, KBC Rijeka, Croatia

This is the report of two patients in whom the diagnosis and treatment of liver amoebomas was made by ultrasound guided percutaneous aspiration puncture. Both were young patients, 33 year old woman and 38 year old man. After the establishment of diagnosis, we decided to treat these amoebomas by percutaneous drainage and systemic intravenous use of metronidazol. In the case of young woman, the result of treatment was little residual cyst of the left lobe which contained clear and sterile liquid. In the second patient amoeboma completely dissapeared with total restitution of the liver parenchyma.

In both cases the successful management of liver amoebomas was done by percutaneous drainage with Angiomed LADS set, guided under the ultrasound control (Hitachi EUB 410).

On the poster all phases of the treatment of our patients are shown.

**P063**

### SIMPLIFIED TECHNIQUE FOR PLACEMENT OF THE NEW DENVER PERITONEOVENOUS SHUNT

R.H. LUND
Associate Professor of Surgery, Washington University School of Medicine
St. Louis, Missouri, U.S.A.

In 1990, the Denver Biomaterials Company developed a new peritoneovenous shunt, with a reduced venous calibre, allowing for easier access to the superior vena cava. The new shunt reduces the operative time, is more conductive to local anesthesia, and is associated with reduced complications.

The flow dynamics of the shunt are presented.

A technique of inserting the shunt with the use of peel-away introducers for both the peritoneo and venous insertion is described in detail.

The new Denver shunt, with this technique, is associated with decreased operative morbidity and with post-op complications. The shunt has been used satisfactorily in both malignant and non-malignant ascites.

**P064**

### GENETIC ASPECTS OF LIVER CIRRHOSIS AND CHRONIC ACTIVE HEPATITIS

A.V. SHAPOSHNIKOV, S.A. SHAPOSHNIKOV, V.G.R. NAYANAR,
Department of Surgery, Rostov Medical Institute, Rostov-on-Don, Russia.

We studied the following genetic characters in 107 patients with liver cirrhosis(LC) & 58 patients with chronic active hepatitis (CAN); Geneological analysis: types of body constitution, antropometric indices (height, weight, types of hands & feet); Dermatoglyphics of palms & fingers; the ABO system & Rh(D) factor. These characters were also studied in 250 healthy men & women. In the Child's classification of LC, the patients under Child A were 7; B-57; C-43. Out of these 40 patients were subjected to different types of operations. Investigations showed that in the first generation the heritability of LC & CAH was 30.4% & 41.6% resp. Ectomorphic type of body constitution was more predominant in LC patients (men-44.6% women-36.2%, in healthy 14.2% & 6.3% resp.). Analysis of finger & palm dermatograms revealed that indices of Furrugata & Cummings in male patients with LC was low (44.7 & 57.6 resp.), in healthy - 68 & 101. These indices were extremely low in female patients with CAH (5.2 & 18.9) in healthy -62.2 & 101.4. Dunkmeirs index raised by 10 times (333.3), in healthy - 30.9. Amongst patients with LC palm line "E" goes right across the palm in 12.5%, in healthy 0%. Patients with LC more often have A-antigen (60.0%) & Rh negative (50.0%), in healthy -36.7% & 17.6% resp. In conclusion it can be presumed that LC & CAH have hereditary predisposition.

**P065**

### ALTERATION OF FLUIDITY OF LIVER CELL MEMBRANES (LCM) FOLLOWING PORTAL BRANCH LIGATION IN CIRRHOTIC AND NORMAL RATS

MITSUAKI KOHMOTO, JUNJI TANAKA, WANG BEN ZHANG, MASANORI YOSHIDA, TAKAYUKI KASAMATSU, KEN-ICHI FUJITA, JUN TAMURA
The First Department Of Surgery, Kyoto University School Of Medicine

Recent interventional modalities have enabled to apply preoperative portal branch embolization thereby leading to safe hepatectomy. On the other hand, membrane fluidity is suggested to play an important role on cellular function. Present study was aimed to clarify the LCM function following portal vein ligation (PBL) in cirrhotic and normal rats. Materials and methods: Cirrhosis was induced by an intraperitoneal injection of thioacetamide for 3 months. PBL refferring to 70% volume of liver was conducted. Membrane fraction was collected by gradient centrifugation. Fluidity was determined by measuring fluorescence polarization (P-value) with DPH as probe dye. Results: P-values of normal LCM are maintained in narrow range of 0.181±0.012 (mean±SD). In normal liver, the P-values were decreased to 0.172 and 0.165 at 12 and 24 hours after PBL, then followed by a gradual recovery to normal level on 7th day. In contrast, P-values of LCM from ligated lobe were increased to 0.196 at 12 hours after PBL and persistently high until 7th day. On the other hand, in cirrhotic liver, the P-values were decreased to 0.175, 0.161, 0.165, 0.164, 0.165, 0.180 and 0.188 at 12, 24, 48 hours, 1, 2, 3 and 5 weeks after PBL. In ligated lobe, the high P-values were continued until 5 weeks studied. Conclusions: Above results indicate that cirrhotic liver requires for longer time in restoring membrane function than normal.

**P066**

### CHANGES IN CHEMICAL MEDIATOR RELEASE AND SURFACE MARKER EXPRESSION OF PRIMARY CULTURED RAT AND HUMAN HEPATIC MACROPHAGES IN LIVER CIRRHOSIS

N.FUNAKI, S.ARII, T.SASAOKI, Y. ADACHI, H. HIGASHITSUJI, S. FUJITA, M. FURUTANI, M. MISE, J. TANAKA, AND T.TOBE
First Department of Surgery, Kyoto University, Faculty of Medicine, Kyoto, Japan

Liver cirrhosis has been well-know by the weakened host defence and altered metabolism which tend to cause serious perioperative complications. In our attempt to clarify this mechanism and to improve the perioperative treatment, we analyzed hepatic macrophages (HMΦ) from male Wistar rat and human of their secretary ability of highly potent mediators and surface markers thought to serve for intercellular communications.

HMΦ from rat with $CCl_4$-induced cirrhosis showed reduction in $O_2^-$, $PGE_2$, and TNF releases, and increases of IL-1 release compared to that from normal liver rat. Surface expressions of IL-2 receptor, and Ia and asialo-$GM_1$ antigens were also decreased in cirrhotic rat.

Human HMΦ from cirrhotic specimen also showed reduction of $O_2^-$ release, and increase of IL-1 release compared to that from normal liver specimen, and decreased $PGE_2$ production in accordance with deterioration of liver function.

Our results may suggest the possible participation of functionally-altered HMΦ in the changes of host defence and metabolism in liver cirrhosis.

**P067**

### CELL KINETICS FOLLOWING PORTAL BRANCH LIGATION IN NORMAL AND CIRRHOTIC RATS

KEN-ICHI FUJITA, JUNJI TANAKA, JUN TAMURA, MITSUAKI KOHMOTO, MASANORI YOSHIDA, TAKAYUKI KASAMATSU, WANG BEN ZHANG

It is well known that portal branch ligation (PBL) induces a regeneration and an atrophy in the nonligated and ligated lobes. This phenomenon has been clinically applied for safe hepatectomy. The present study was aimed to investigate cell kinetics following PBL in normal and cirrhotic rats. Materials and methods: Cirrhosis was conducted to an intraperitoneal injection of thioacetamide for 12 weeks. PBL referring to about 70% volume of liver was performed in normal and cirrhotic rats. Cell kinetics were analyzed using flow cytometry with 2-color analysis of propidium iodide and FITC-conjugated antibody for bolusly injected BrdU. Results; DNA histogram changed as follows; Diploid nuclei in non-ligated lobe: 57% (25), 76.8 (36.5), 52.6 (26.5), 57 (37), 42 (18.5), 50 (26) at 24, 48, 72, 168 and 336 hours after PBL in cirrhotic rats (normal). In ligated lobe, C2 nuclei were; 57% (25), 73 (36), 66 (32), 60 (57), 48 (29), 55 (21) at the given time course. DNA histogram of cirrhotic rats showed similar tendency to those of normal. On the other hand, the peak of BrdU uptaken nuclei in nonligated lobe were delayed up to 3 days, in reference to 2 days in normal. No significant measurement were observed either normal, cirrhosis or ligated and non-ligated lobes. Conclusion: Above results suggest that cirrhotic liver has similar cell kinetics after portal branch ligation but delayed peak of S-phase. Such time-consuming regeneration in cirrhotic liver may need longer observation until safe operation.

**P068**

### MAJOR SURGERY FOR HEPATIC HYDATIDOSIS

G. BELLI, A. MONACO, M.F. ARMELLINO, F. PASTENA AND M.L. SANTANGELO
Istituto Chirurgia generale e Trapianti II Faculty University of Naples, Naples, Italy

During the last years hepatic hydatidosis has been usually treated by marsupialization. Only recently aggressive surgical procedures, such as hepatic resections and pericystectomy, have gained worldwide acceptance. At our Istitution between 1983 and 1990, fifteen patients with hepatic hydatidosis were treated, including three recurrences. There were 9 females and 6 males with an average age of 48.7 years (range 31-71). The location of the cyst was: right lobe 11, left lobe 1, caudate lobe 1, right lobe plus IV segment 2. The marsupialization was performed in 5 cases, capitonnage in 1, pericystectomy in 8 and right trisegmentectomy in 1. Two biliary fistulas after marsupialization and one digestive emorragy by peptic ulcer after pericystectomy were observed. We had no postoperatory mortality and no recurrence was observed in the follow-up (1 - 7 years). Due to the low incidence of morbidity, mortality and recurrence we conclude that pericystectomy or hepatic resection, when indicated, should be the treatment of choice for hepatic hydatidosis.

**P069**

### BILIARY COMPLICATIONS IN LIVER HYDATIDOSIS: INCIDENCE, SURGICAL TREATMENT AND OUTCOME

G.M. DANIELE, G. PISANO, G. GARAU, M. POMATA
Dept. Gen. Surgery, University of Cagliari
Ospedale S. Giovanni di Dio, 09124 Cagliari, Italy

Biliary complications in liver hydatidosis include rupture of the cyst into the biliary tract and what is commonly called parahydatid pathology: that is gallstones, common bile duct stones and papillitis. The present study was undertaken to evaluate the real incidence of such complications and the results of surgery over a large series of patients operated on for hydatid cysts of the liver. Over a total of 261 patients, biliary rupture occurred in 57 cases (21.8%) and parahydatid pathology in 58 cases (22.2%). Biliary rupture was found most frequently in single cysts (78.9%), mainly large ones (45.6%). The site of rupture involved the right and left hepatic duct in 61.4% of the cases. In 9 patients (15.9%) rupture was found in recurrent cysts. Radical procedures (hepatic resection and total pericystectomy) were possible in 61.4% of the cases. Biliary drainage was accomplished in all cases of biliary rupture, in 31 patients with parahydatid pathology and in other 21 cases of the whole series. For this purpose elective procedure was transduodenal sphyncteroplasty performed in a total of 107 patients. Mortality related to the 57 cases of biliary rupture was 5.2% and postoperative complications in the same group was 30.3%. The authors stress the importance of transduodenal sphyncteroplasty as the procedure of choice in such complications of liver hydatidosis.

**P070**

### HAEMOPERITONEUM AS INITIAL SIGN OF POST-TRAUMATIC RUPTURE OF HEPATIC HYDATID CYST (PRHHC): A CASE REPORT

C. FAMULARI, G. MAZZEO, A. MACRI', M.L. TERRANOVA, T. CENTORRINO, M.T. FONTI, V. LEPORE, D. CUZZOCREA.
Surgical Pathology Messina University - Italy

A young man was admitted as emergency with a clinical feature of severe hypovolaemic shock and an history of abdominal trauma. The US and CT scan revealed a cystic lesion (ø 82 mm) of type I according Gharbi classification in the 7th segment and an unhomogeneous area of deep parenchymal laceration (ø 80 mm) interesting the 5th and the 6th segment of the liver; a considerable hemoperitoneum was present. At emergency laparotomy the exploration of the liver pointed out a right hilar echinococcal cyst with an anterior and posterior tear of pericyst, full of clotted blood; the endocyst was found in pelvis and taken away. A total pericystectomy with US guidance was performed, the residual cavity was closed. We preferred to leave "in situ" the cyst in the 7th segment and treat it afterwards by PAIR technique. The peritoneal cavity was washed with 1% cetrimide solution to prevent metastatic hydatosis. The patients was discharged well in 18th day with albendazole. Follow up at 9 months results free of relapse. We emphasize that hemoperitoneum as intial sign of PRHHC put diagnostic doubts for the unusual clinical presentation and for the US and CT images suggestive of liver parenchyma lesion. Total pericystectomy is the most efficient surgical technique in the treatment of hydatid cysts.

### P071

### HEPATIC ECHINOCOCCOSIS. A BENIGN DISEASE?

T. LIAKAOS, J. KOUFOPOULOS, D. KAKOULIDIS. S. DENDRINOS
2nd Surgical Department of Athens General Hospital.
152 Mesogeion Av. Athens Greece.

To assess the efficacy of various surgical techniques in the treatment of hepatic enchinococcosis we reviewed 325 operations which were carried out from January 1973 till December 1990 on 254 patients with liver hydatid disease. We performed 70 radical pericystectomies with or without typical hepatic resections (group A) and 247 conservative surgical procedures (group B). There was 1 postoperative death in group A and 3 in group B (overall mortality rate 1.5%). Mean hospital stay was 14 and 14.5 respectively. 12 major complications (17%) were noted in group A,5 of which (7.1%) required reoperation. In group B there 55 major complications (22.2%) $p<0.05$ and 13 of those (5.2%) were also reoperated $p<0.05$.

A total of 221 patients (88.4%) were available for follow-up (mean follow up time 8.7 years). Recurrent disease was detected with liver nucleotide scan (in the early patients), ultrasonography, CT-scan and serologic tests. 63 reoperations were carried out for recurrent disease in 51 patients from both groups (overall recurrence rate 23%). The recurrence rate was 11.6% (8 patients) and 23.7% (43 patients) for group A and B respectively $p<0.05$.

Though the mean hospital stay, the rate of major complications and reoperations were almost identical in both groups the control of the recurrence seems to be better in group A. We think that the use of radical procedures on every case could be extremely hazardous and the increased morbidity will outweigh the benefits of the recurrence control. The surgical treatment of the hepatic hydatid cyst is a difficult and complicated procedure and the recurrence rate of the disease is still very high. We believe that the future of its treatment lies on the development of new chemotherapeutic agents.

### P072

### MANAGEMENT OF HYDATID CYST-INTRAHEPATIC BILE DUCT COMMUNICATIONS

M.MILICEVIC, B.STEFANOVIC, Z.RADOVIC, M.BULAJIC, M.PETROVIC, I.JEKIC, P.BULAJIAC AND D. STEFANOVIC
Institute for Digestive Diseases, University Clinical Center,
Belgrade 11000, Yugoslavia, Koste Todorovica br. 6

A total of 368 patients with hydatid cysts of the liver were operated at the Institute during a period ranging from 01.01. 1966. to 01.01. 1990. Hydatid cyst-intrahepatic bile duct communications were found in 90 pts. (24.5%). Age of the patients ranged from 12 to 66 years with a median age of 39.9 years. The diameter of the cysts ranged from 5.5cm to 20cm with a median diameter of 10cm. Solitary cysts were found in 76.4 pts and the right lobe was involved in 68.1% pts.

When possible (visible large communication) bile ducts were visualized (intraoperative cholangiography or intraoperative ultrasound) and level of communication assessed.

Operative management was suture in 73 pts. (81.1%-minor, peripheral ducts), suture combined with "T" drainage and choledochoduodeno-anastomosis in 10 pts. (11.1%), while 7 pts. (7.8%) had "T" drainage only. The postoperative stay ranged from 7 to 93 days, the average stay was 24.7 days. The most frequent complications were wound infection 21 pts. (23.3%), pleural effusion in 13 pts. (14.3%) and biliary fistula in 7 pts. (7.7%).

The long hospital stay was predominantly due to infection and secretion of the wound. Biliary fistula closed spontaneously in 4 pts., and endoscopic sphincterotomy was necessary in 3 pts. The authors conclude that hydatid cyst-intrahepatic bile duct communications are frequent and during operation they have to be looked for regardless of the color of the cystic content. Prolonged hospital stay is due to a high incidence of infected cystic contents and resulting postoperative infection. Long term results are good, conservative management spares liver tissue and postoperative duct fistulas can be managed without operation.

### P073

### RUPTURE OF HYDATID CYST OF THE LIVER INTO THE BILIARY TRACT

A.PAPAVASILIOU, C.SIMOPOULOS, A.POLYCHRONIDEDS, A.BOUNOVAS
Propedeutic Dept. of Surgery, Thrace University, Alexandroupolis, Hellas.

Rupture of hydatid cyst of the liver into the biliary tract is the most common complication of the disease, with an incidence up to 5-25%

18 patients underwent operation for intrabiliary rupture of hydatid cysts. In our series, 10 were females, (aged 32-80 years, mean 48 years) and 8 males (aged 15-73 years, mean 47 years).

Four types of operations were performed:

1. Exploration and drainage of the common bile duct by T-tube, and external drainage of the cyst. (11 patients).

2. Choledochoduodenostomy and external drainage of the cyst. (3 patients).

3. Common bile duct exploration and choledochoduodenostomy without external drainage of the cyst. (2 patients).

4. Common bile duct exploration and external drainage with T-tube, without external drainage of the cyst (2 patients).

Choledochoduodenostomy is prefered in dilated ducts, whereas sphincteroplasty seems to be more suitable in cases with normal common duct.

### P074

### SUCCESSFUL ENDOSCOPIC MANAGEMENT OF POSTOPERATIVE BILE LEAK (PBL) AFTER SURGICAL TREATMENT OF LIVER HYDATID CYST DISEASE (HCD)

AA POLYDOROU, C VAGIANOS. E SKANTZOS, E KRESPIS, I ANDROULAKIS
Departments of Surgery, Hippocration Hospital, Athens Rion University Hospital, Patrai, GREECE

Persisted PBL is a serious complication following surgical treatment of liver HCD. Until recently relaparotomy was necessary. We describe five cases of external PBL after surgical drainage of liver HCD successfully treated by endoscopic sphincterotomy (EST) and nasobiliary drain (NB) placement. Four patients were male and the mean age was 45 yrs (25-70). The patients had high output external biliary fistulas (500-1800 ml/day) persisted for a period of 4-20 weeks. ERCP demonstrated the site of biliary leakage in all patients. EST was performed and a 7 Fr NB was placed at the closest possible site where the bile leak was detected by the cholangiography. Bile was continuously aspirated through the NB using an underwater drainage system, applying a negative pressure of 20cm $H_2O$. After this procedure the bile leak was decreased dramatically and ceased in all patients 5-15 days later. Repeat cholangiograms confirmed the fistulas healing and the NB were then removed. There were no complications of EST or NB placement. ERCP is highly effective in determining the site of biliary leakage. In high output fistulas with multiple communications with the biliary tree EST may not be sufficient treatment. The selective placement of a NB drain combined with continuous aspiration of bile seems to be the treatment of choice in patients with high output external PBL following surgical treatment of liver HCD.

### P075

### CT DIAGNOSIS OF HEPATIC ECHINOCOCCOSIS

XU MING-QIAN, DONG ZHAO-HO
The People's General Hospital of Xinjiang Uygur Autonomous Region, Urumqi, Xinjiang, 830001 China

The diagnosis of hydatid disease is based on: (1) a history of contact with dogs or sheep in prevailing areas; (2) absence of subjective symptoms in early stage of infection and the gradual occurrence of pressure symptoms with the slow growth of hydatid cyst; (3) the formation of liver abscess as a result of complicated infection of hydatid cyst and the development of acute abdomenal pain and anaphylactic shock as a consequence of rupture of hydatid cyst; (4) the typical sign on palpation of hydatid cyst projecting under the liver; (5) the acoustic images displayed by ultrasonic detection; and (6) immunological diagnosis.

These procedures, however, are difficult to find early pathological changes. The use of computerized X-ray tomography in the diagnosis of hepatic echinococcosis not only makes it possible to diagnose an asymptomatic parasite-carrier but also detects accurately images of various pathological pictures of hydatid cyst. The preoperative diagnostic accuracy rate in this series was 98.8%.

### P076

### PALLIATIVE TREATMENT OF PRIMARY AND SECONDARY MALIGNANT LIVER NEOPLASMS

EMM.BANDOUVAS, N.ALEANDRIDOU, J.PARHARIDOU, E.ANDROULAKAKIS, C.KOUNDOURIS, KALISTROS, ASTERIADIS, E.KIVERNITAKI, P.KADMAKIS, A.MITSIS, A.SALMARIDIS, H.MARGARI, E.KOURI, H.KARAIOSIFIDOU
"Helena Venizelou" Hospital, 2 E. Venizelou Sq., GR-11521 Athens, Greece

The majority of patients with primary or secondary liver malignancies are not suitable for radical surgical procedures aiming at removal of the tumours.

We are referred to 102 patients, 30 of whom with colorectal metastatic cancer were treated with chemotherapy and had got median response total rate 40%, median duration of responmders 6 months, median survival rate of responders 12 months and non responders 6 months. Forty patients had got local arterial administration of chemotherapeutics with 80% response and 10 months duration of it and median survival 18 months against 13 months median survival of non responders. Twenty patients with single or metastatic tumours were treated by resection and showed 5 years survival time in up to 50%, 10 year survival in 25%. Twelve patients treated by transient occlusion of the hepatic artery combines with regional cytostatic infusion had got total response rate: 90%, duration 16 months, survival rate: 22 months.

The most preferable method of treatment in unresectable cases is transient occlusion of the hepatic artery combined with intra-arterial cytostatic infusion, because of comparatively low peroperative mortality.

### P077

### ATTITUDES TOWARDS DETECTION AND MANAGEMENT OF HEPATIC METASTASES: A SECOND LOOK AFTER FIVE YEARS

D.J. BRUINVELS, L.M. DE BRAUW, J.KIEVIT, C.J.H. VAN DE VELDE,
Department of Surgery, University Hospital Leiden, The Netherlands

In order to obtain information about attitudes towards detection and management of hepatic metastases of colorectal origin, five years ago a questionnaire was sent to 248 hospitals in 13 western countries. The response rate was 98%.

In 98% of all hospitals a standardised follow up regimen was used, in 84% cea-levels were measured, and hepatic resection of liver metastases was performed in 95%. However, resectability creteria varied considerably between countries and hospitals, ranging from solitary metastases in most Dutch and British hospitals to multiple bilobar metastases in most German hospitals.

Concerning non-operative treatment, the majority (74%) of all hospitals administered some form of chemotherapeutic treatment to patients with irresectable metastases, while 33% gave supplied adjuvant chemotherapy after resection of hepatic metastases.

By using a simplifed decision analytic model based on the utility of follow up, an extended questionnaire has been designed, and has been sent to all 1960 members of the World Association of Hepato-Pancreato-Biliary Surgery in November 1991. The results of this new investigation will be compared with those of the preceding one, and will be evaluated using decision analysis in order to assess the variability in and the potential usefulness of liver surgery for colorectal cancer metastases.

### P078

### PATTERN OF RECURRENCE AFTER LIVER RESECTION FOR COLORECTAL LIVER METASTASES

G. GOZZETTI, G.L. GRAZI, E. JOVINE, A. GALLUCCI, A. MAZZIOTTI
Clinica Chirurgica 2 o, University of Bologna, Bologna, Italy

Liver resection (LR) is still discussed in the cases of pts with colorectal matastasis for the high recurrence rate after surgery. PATIENTS AND METHODS: In order to evaluate the patten of recurrence after curative LR for colorectal metastases, 23 clinical variables of 64 pts operated on over the past 10 years have been crosstabulated with the appearance of recurrence and results have been evaluated by means of the chi-square test. RESULTS: Thirty (46.9%) pts are currently alive and 34 (53.1%) died. Recurrence appeared in 24 (40%) pts and it has been the cause death in 16 pts. Four pts received a repeated LR for recurrence and 3 of them are currently alive with a follow-up ranging from 1 to 34 months. Univariate analysis has shown that recurrence appeared more frequently in pts with lesions of more than 5 cm and who had major LR ($p<0.05$), but the use of perioperative blood transfusions seemed to be the most important single factor for the development of new neoplastic lesions ($p<0.001$). CONCLUSIONS: Care has to be take in monitoring patients after LR for colorectal metastases, especially if some risk factors are present for recurrence. Early detection of new lesions allows the performance of repeated resections in selected cases, with satisfactory long-term survivals.

**P079**

### DO ALTERATIONS IN RENAL HAEMODYNAMICS PREDICT THE PRESENCE OF HEPATIC MICROMETASTASES?

SA JENKINS, DM NOTT, J YATES & T. COOKE
University Department of Surgery, Liverpool, UK

We have previously shown that the elevation in the hepatic perfusion index (HPI) in the presence of overt tumour in the rat is accompanied by a significant decrease in renal blood flow (RBF). Since there is little information on the temporal relationship between alterations in RBF and the growth and development of hepatic tumour, we have undertaken such a study in the rat. Male Fisher rats received an intraportal injection of 1.6 x $10^7$ Walker cells and controls with the same number of dead cells. RBF and hepatic blood flow were measured by the microsphere method and by dynamic scintigraphy 2,4,6, and 21 days after intraportal administration of viable or dead Walker cells. Micrometastases were present histologically at 4 days and the HPI (51.8±1.68) was significantly greater (p<0.0001 Mann Whitney U test ) than in controls (33.78±1.77). The increase in the HPI at 4 days was accompanied by a significant increase in the mean renal transit time (3.95±1.18 sec) compared to controls (2.28±0.48 secs). Conversely, the RBF was significantly reduced in animals with micrometastases (7.32±2.81 ml/min) compared to controls (14.02±3.42 ml/min). These changes in HPI, renal transit time and RBF were maintained at 6 and 21 days. These results suggest that RBF is reduced during the early stages of growth and development of hepatic tumour. Furthermore, since renograms with a flow phase are technically easier to perform in man that estimations of the HPI, determination of RBF may be of value in the detection of occult liver metastases.

**P080**

### SANDOSTATIN (SMS) INHIBITS THE GROWTH OF TWO ANIMAL MODELS OF COLORECTAL LIVER METASTASES IN THE RAT

S.A. JENKINS, N. DAVIES, J. YATES, H. KYNASTON, B.A. TAYLOR
University of Liverpool Dept of Surgery, Liverpool, UK

The treatment of colorectal liver metastases cancer remains poor, the majority of patients dying within one year of diagnosis. We have developed two models of liver metastases which reliably produce hepatic tumour following intraportal (IP) inoculation of tumour cell lines in which we have investigated the effects of SMS on their growth and development. Following IP injection of 1 x $10^7$ K12/Tr cells (an adenocarcinoma of colonic origin syngeneic to the BDIX rat), or 4x$10^6$ HSN cells (a fibrosarcoma syngeneic to the Hooded Lister (HL) rat), groups of 12 rats received either SMS 2 ug bd or saline (control) for 3 (HL rats) or 4 (BDIX rats) weeks. There was a significant reduction (Mann Whitney U, p<0.001) in the percentage hepatic replacement of hepatic tumour in the SMS treated grpups compared to controls, 17.5%, 5.7-24.2% and in HL rats; median 2.7%, 0-26.5%; controls; median 76.4%, 56.3-85%. The reduction in the growth and development of both types of tumour was associated with a marked stimulation of hepatic RES activity and a decrease in liver blood flow compared to controls, offering an explanation, at least in part, for the mechanism of action of the somatostatin analogue. These results indicate that SMS significantly inhibits the progression of hepatic tumour in two animal models of liver metastases and may be of benefit in the treatment of hepatic metastases in man. Further studies are required to evaluate this hypothesis.

**P081**

### RETICULOENDOTHELIAL SYSTEM (RES) STIMULATION: LEVAMISOLE COMPARED WITH OTHER KNOWN RES STIMULANTS

S.A. JENKINS, N. DAVIES, J. YATES, B.A. TAYLOR
University of Liverpool Dept of Surgery, Liverpool, UK

Combined adjuvant therapy with fluorouracil and the immunomodulatory drug levamisole has been shown to significantly increase survival in patients with Dukes C colorectal cancer. The reason for levamisole's efficacy is not known. We have compared the effect of levamisole on the hepatic and splenic reticuloendothelial system (RES) with other known RES stimulants. Groups of 10 male wistar rats received either saline (control), glucan, zymosan, chlormethiazole, sandostatin or levamisole. RES was assessd by the hepatic and splenic uptake of 99m Tc sulphur colloid (SC), 20 minutes after an intravenous injection of 2.5 MBq of colloid. Hepatic uptake of SC was significantly increased in all the treatment groups (p<0.001 Mann whitney U), when compared to the control group (medican, range). Controls (4.8, 2.6 - 16.3), glucan (14.9, 7.5 - 30.1), zymosan (12.6, 6.8 - 35.2k), chlormethiazole (24.1, 16.6 - 34.3), sandostatin (34.4, 29.9 - 45.1) and levamisole (15.4, 6.3 - 17.6 ). Splenic uptake of SC was significantly increased in all groups except the levamisole group. Compared to controls, sandostatin increased uptake of sulphur colloid significantly more than levamisole in both liver and spleen (p<0.005). The results of this study suggest that levamisole is a stimulator of hepatic RES function and this may account for its efficacy in adjuvant therapy. Sandostatin is a more potent stimulator of RES activity and its use as an adjuvant in the treatment of colorectal cancer deserves further investigation.

**P082**

### EFFECTIVE TREATMENT OF LIVER TUMOURS IN RATS WITH MELPHALAN IN ISOLATED LIVER PERFUSION (ILP)

A. MARINELLI, LM DE BRAUW, P KUPPEN, CJH VAN DE VALDE
Departments of Surgery and Pathology, University Hospital Leiden,
P.O. Box 9600, 2300 RC, Leiden, The Netherlands

The achieved concentration of cystostatic drugs in tumour tissue is an important factor in determining the effectiveness of anticancer therapy. An ILP technique has been developed to allow treatment with higher and more effective doses of anticancer agents. Administering the maximally tolerated doses (MTD) of melphalan in ILP (12mg/kg) and in hepatic artery infusion (HAI; 6mg/kg), ILP resulted in a four times higher concentration in liver tumour tissue. To determine the bonus effect of this higher concentration in liver tumours, tumour bearing rats were randomly assigned to five groups: three control groups (no treatment, sham HAI, sham ILP) and two groups treated with the respective MTD of melphalan in HAI and ILP. Both sham treatments had no effect on the tumours. In a cell kinetic study flow cytometric DNA analysis revealed that tumour cells in rats treated with melphalan in ILP were blocked much earlier in their cell cycle than tumour cells in HAI treated rats. This suggests that higher concentrations were achieved in tumour cells of ILP treated rats. In the tumour growth study complete remissions were observed in nine out of ten ILP but in none of the HAI treated rats. It can be concluded that treatment with the MTD of melphalan in ILP did, but in HAI did not result in high enough concentrations of melphalan in the tumour cells to kill them and induce complete remissions. In our model the results with melphalan were better than those with 5-fluorouracil and those with mitomycin C. Therefore, in 1991 a clinical phase II study with melphalan in ILP has been started in our department.

### P083

### THE USE OF SAPHENOUS VEIN GRAFTS TO CIRCUMVENT ANATOMICAL VARIATIONS ENCOUNTERED AT HEPATIC ARTERY CATHETERISATION

C S MCARDLE, J A GOLDBERG, J H ANDERSON, T G COOKE, D P LIEBERMAN, I STEWART

University Department of Surgery, Royal Infirmary, Glasgow, Scotland

The advantages of hepatic arterial chemotherapy for colorectal liver metastases are an increase in tumour drug levels and reduction in systemic toxicity. Complex surgical procedures have been described to insert and maintain hepatic artery catheters. We describe a simple method of inserting catheters.in patients with anomalous vascular anatomy.

Of 55 consecutive patients with colorectal liver metastases undergoing hepatic artery catheterisation, anatomical variations were present in 18 (33%). Nine patients had a dual blood supply, most commonly an unbranching right hepatic artery arising from the superior mesenteric artery and a left hepatic artery arising from the coeliac axis.

In the latter patients, adequate perfusion was obtained by inserting two catheters, one into the gastroduodenal artery to perfuse the left lobe and a second into the right hepatic artery using a saphenous vein graft as a conduit.

This technique is widely applicable to patients with abnormal hepatic arterial anatomy.

### P084

### THE VALUE OF DUPLEX/COLOUR DOPPLER SONOGRAPHY IN THE DETECTION OF COLORECTAL LIVER METASTASES

C S MCARDLE, E LEEN, J A GOLDBERG, T G COOKE, J ROBERTSON, G R SUTHERLAND
University Department of Surgery and Department of Radiology, Royal Infirmary, Glasgow, Scotland

Conventional imaging techniques are unable to detect small liver metastases due to limited resolution and contrast differentiation. It is know however that the presence of hepatic metastases leads to subtle changes in liver blood flow, and it is possible that by monitoring these haemodynamic changes, earlier detection of liver metastases may be feasible. In this study, a novel method of detecting liver metastases by measuring liver blood flow using a Duplex/Colour Doppler System was assessed.

Hepatic arterial and portal venous blood-flows were measured in 16 controls, 50 patients with gastro-intestinal cancer and 6 patients with breast cancer. The ratio of hepatic arterial: total liver blood-flow (Doppler perfusion index, DPI) and the ratio of hepatic arterial: portal venous blood-flow (Doppler flow ratio, DFR) were calculated.

The DPI and DFR values in patients with overt liver metastases were significantly elevated when compared with controls ($0.52\pm0.13$ V $0.13\pm0.07$ and $1.27\pm0.71$ V $0.15\pm0.10$ respectively: $P<0.0001$).

The results suggest that Duplex/Colour Doppler ultra-sound measurement of hepatic perfusion changes may be of value in the detection of liver metastases.

### P085

### RESULTS OF REGIONAL THERAPY FOR COLORECTAL LIVER METASTASES

C S MCARDLE, J A GOLDBERG, J H ANDERSON, T G COOKE, N WILLMOTT, A WHATELEY, D KERR
University Department of Surgery, Royal Infirmary, Glasgow, Scotland

Until recently the results of systemic chemotheapy have been regarded as disappointing. Attempts to improve response rates have been limited by systemic toxictiy. Attention has therefore turned to the use of regional therapy whereby high levels of therapeutic agent are achieved in the target organ whilst systemic exposure is reduced.

We have developed and tested a variety of biodegradable and non-biodegradable cytotoxic-loaded and radioactive microspheres to be administered via an indwelling hepatic artery catheter with or without Angiotensin II infusion. Systemic toxicity was minimal. Unfortunately, although regression of stable disease was achieved in most patients, survival was limited by the development of extrahepatic disease, mainly lung metastases.

We have recently completed a phase I study of weekly 24 hour regional 5-FU infusion in combination with folinic acid. Dose limiting toxicity was encounted at 2.0 $g/m^2$/week 5-FU, a level substantially above that used systemically. Disease progression was delayed in all patients. To date, none of the patients have died (follow up 8-16 months). A phase II study is now under way.

### P086

### PREOPERATIVE HEPATIC IMAGING IN THE DETECTION OF COLORECTAL METASTASES

C.G. SCHULZE, H. KELLER
Departments of Surgery and Radiology, University of Freiburg, Germany

Over a 36 month period, 26 patients with hepatic metastases of colorectal carcinoma were preoperatively evaluated with 4 imaging techniques. The results of ultrasonography (US), intravenously enhanced computerized tomography (CT), selective hepatic arterial contrast CT, and magnetic resonance imaging (MRI) were compared with operative findings including intraoperative US. A total of 49 lesions were identified, the sensitivity for tumour detection being: arteriographically enhanced CT 86%, MRI 70%, CT 66%, and US 64%. This advantage for arteriographically enhanced CT was most marked for lesions less than 1 cm in diameter. MRE was clearly superior to arteriographically enhanced CT or CT in detecting vascular involvemnet (85% versus 30% and 12%, respectively; $p<0.01$). A combination of ateriographically enhanced CT and MRI should be used for preoperative assessment of hepatic metastases.

### P087

### SURGICAL TREATMENT FOR HEPATIC METASTASES FROM COLORECTAL CANCER - INFLUENCE OF CLINICOPATHOLOGICAL FACTORS ON SURVIVAL

M. SHIMAZU, Y. KOMORI, J. SUZUKI, T. ESAKI, A KIMURA, Y. YASUDA, J. FUJITA, H. SUGENOYA, A. HASUMI, M. MARUTA, H. AOKI
Department of Surgery, Fujita Health University School of Medicine, Toyoake, Japan

From 1974 to 1991, 52 patients with hepatic metastases from colorectal cancer underwent 57 curative hepatic resections. Thirty-two patients had unilobular metastases (mutiple in 8 patients), and 20 patients had bilobular mutiple metastases. The 1-month operative mortality rate was 0%. The overall 5-year survival rate was 40.6%. Five-year survival rate of the patients with unilobular metastases was 54.2%, significantly higher than 17.5% of the patients with bilobular metastases. The tumor distribution rather than the number of metastases appeared to markedly influence the survival rate after hepatic resection. In the patients with unilobulaar lesions, metachronous metastases seemed to have survival advantages when compared with synchronous metastases. In the patients with bilobular lesions, the presence of small tumors ≤10mm indicating the latency of micrometastases seemed to prove poor survival within 19 months, althought 5-year survival rates of the patients with tumors>10 and>20 mm were 27.9% and 37.5%, respectively. Multivariate analysis using Cox's proportional hazard model showed that microscopic invasion to sinusoidal space or intrahepatic portal vein was an independent prognostic factor.

### P088

### PATTERN OF F-18-FLUORO-D-GLUCOSE (FDG) ACCUMULATION IN LIVER TUMOURS: PRIMARY AND SECONDARY CANCERS

F.W. SMITH, S.D. HEYS, N.T.S. EVANS, D. ROEDA, P. SHARP, J.R. MALLARD & O. EREMIN.
Depts of Med. Physics, BioEng. and Surg, Aberdeen Univ, Medical School, Foresterhill, Aberdeen AB9 2ZD

Thirteen patients, aged 56-72 years, with advanced coloretal cancer, have been studied using F-18-FDG and Positron Emission Tomography (PET). One patient had a histologically proven hepatoma and twelve had adenocarcinoma of colon-rectum treated surgically. Ten of latter had liver metastases but 2 had no evidence of hepatic involvement. In 7 of the patients with metastatic tumour, a second examination was performed 4 weeks after treatment; in 3, a third examination was performed 5 months later. In all cases, the radioactivity was seen to accumulate in the periphery of the tumours with a large central area showing no uptake, and matching the periphery of the tumour as demonstrated by magnetic resonance imaging (MRI). In 5 cases imaged after immuno-chemotherapy (static response - ultrasound, x-ray CT and MRI), there was no change in tumour uptake of 18-F-FDG when imaged with PET. In 2 patients, however, tumour regression (>50%) was documented with serial 18-F-FDG and PET and confirmed by other imaging modalities. In the 2 cases of suspected but not proven metastases, no abnormal accumulation of F-18-FDG was seen.

PET is a non-invasive and sensitive method for detecting hepatic metastases and for monitoring response to treatment.

### P089

### INDUCTION INTRAARTERIAL CHEMOTHERAPY PLUS CYTOREDUCTIVE SURGERY FOR MULTIPLE LIVER METASTASES

M. STEVES, J. VIDAL-JOVE, P. SUGARBAKER
The Cancer Institute, Washington Hospital Center, 110 Irving St., NW Washington, DC 20010

An estimate of 12% of patients with colorectal cancer will develop metastases limited to the liver. Fifty percent of these will present multiple liver metastases and therefore they will not be candidates for standard surgical approach. Intraarterial chemotherapy alone results in high response rates but little improvement in survival. The hepatic cytoreduction protocol has combined the benefits of regional chemotherapy with consolidation surgery and/or interstitial radiation therapy in a population of patients with multiple liver metastases. At present, seven patients have been entered into this protocol study. Patients were treated with induction hepatic artery infusion of mitomycin C (20 $mg/m^2$ infused via a peripherally placed catheter). Two cycles with 4 week intervals were employed before consolidation treatments. All metastatic disease remaining in the liver was then resected using loop and ball-tip electrocautery. Unresectable tumor nodules were treated by radiation therapy using 125I seeds placed at 0.5cm intervals. No major complications of this technique were observed. Follow-up has been short and 3 patients are awaiting surgery. The hepatic cytoreduction protocol with induction intraarterial chemotherapy provides a comprehensive multimodality treament plan for patients with mutiple liver metastases.

(1)-R. Sarper, W.A. Fajman, V.A. Tarcan and D.W. Nixon. J Nucl Med 1981; 22:318-321

### P090

### RSU-1069 WITH DEARTERIALIZATION FOR LIVER TUMOUR

LIQING WANG, BO G. PERSSON AND STIG BENGMARK
Dept. of Surgery, Lund University, Lund Sweden

In this work we wanted to find out if a single dearterialization for 2 hours combined either with the new bioreductive drug RSU-1069 (a nitroimidazole agent), or mitomycin C (MMC) was more effective than either treatment alone to delay the growth of a liver tumour (adenocarcinoma transplanted to the rat liver). Both these drugs are known to be preferentially cytotoxic under hypoxic conditions. Five days after inoculation forty-seven rats were randomly divided nto the following groups: A (n=7), RSU-1069 (49mg/kg) injected intoperitoneally 15 minutes before initiation of a single dearterialization for 2 hours with an implanted minioccluder; B (n=6). a single dearterialization for 2 hours; c (n=6). MMC (5mg/kg) before 2 hours of dearterialization; D (n=6). MMC alone; E (n=6). RSU-1069 alone; F (n=6) sham operation as control. Before the treatment, on day 12 and 18 the tumours were measured.

Results: The tumour growth was dramatically delayed in group A compared to all the other groups (p<0.05) and only modestly delayed in group C compared to D, E and F (p<0.05).

Conclusion: These results showed the effectiveness of dearterialization in potentiating the bioreductive toxicity of RSU-1069 and, though to a lesser extent, MMC. Thus bioreductively activated cytotoxicity may be of potential use together with dearterialization to the treatment of hepatic tumours.

### P091

### PREDICTABILITY OF HEPATIC METASTASES AND HEPATIC PERFUSION INDEX

E. XAVIER DA CUNHA, ANA FERRER ANTUNES, J. RODRIGUES BRANCO, F. CASTRO SOUSA.
Dept. of Surgery III and Dept. of Nuclear Medicine - Coimbra University Hospital; Coimbra, Portugal.

Diagnosis of hepatic micrometastases is an unsolved problem in digestive surgical oncology. Hepatic perfusion Index (HPI), the relative hepatic arterial contribution to total liver blood flow, derived by dynamic hepatic scintigraphy (DHS) (1) has been suggested as a promising method in the definition of a high risk group of patients prone to develop overt hepatic metastases after an apparently curative resection. In this prospective study we attempted to evaluate the utility and reproducibility of preoperative determination of a HPI in 147 patients with a digestive cancer. A normal range has been obtained in a group of 17 non neoplastic patients with an upper limit of 0.28. Overt liver metastases were found in 34 patients undergoing laparotomy, and 26 had elevated HPI values (76.4%). No apparent hepatic metastases were found in the remaining 113 patients submitted to a "curative" resection of the primary tumour; 64 had elevated HPI values. These patients were followed up by periodic clinical examination, hepatic ultrasonography and CT scans. At one year of follow-up 64 patients were evaluated. Seven patients developed hepatic metastases; six of these patients had initial elevated HPI values and one had an initial normal value. Fifthy-seven patients did not developed hepatic metastases, 36 had elevated initial HPI values and 28 had normal HPI values. These data result in a sensitivity of 76.9% and a specificity of 43.3% for DHS and a predictive value for a positive HPI of 16.7% at one year, and a predictive value for negative HPI of 96.4%. These results showed that although initial elevated HPI values did not identify high risk group of patients prone to develop hepatic metastases, patients with initial normal HPI values have a confirmed low risk of developing hepatic metastases.

### P092

### CEA IN BLOOD AND BILE PROBES IN BENIGN AND MALIGNANT GASTROINTESTINAL DISEASE

K. W. JAUCH, M. HEISS, G. MEYER, S. VOGEL, R. LAMERZ
Surgical and Medical Department, University Clinic Großhadern, Munich FRG

The clinical relevance of increased CEA-levels in bile as early sign of liver metastases or hte risk of metastases in colorectal cancer was described by Yeatman et al (Ann. Surg. 213, 113, 1991).

We determined CEA concentrations in blood and bile in 50 patients with gall stones (group1), 10 patients with benign bowel disease (group2) and 65 malignant disease (group3). CEA determination used a conventional RIA and percloric CEA extraction of bile.

Serum CEA was elevated only in 34% of group3 patients. CEA of bile in group 1 and 2 was between 2 and 153ng/ml, while in group3 hte concentrations were significantly higher and reached levels up to 12700ng/ml. In group 3 we found a significant correlation of serum and bile CEA witha r=0,632.

Using ROC analysis an optimal cut off level of bile CEA was determined at 45 ng/nl. Above this level were 16 and 20% of group 1 resp. 2 and 52% of group3 resp 83% when liver metastases were apparent. Follow up will show if elevated bile CEA exceeds prognostic beside diagnostic value.

### P093

### THE TIMING OF SURGERY IN NECROTIZING ACUTE PANCREATITIS

A. AGOROGIANNIS, E. NAOUM, IOANNA AGOROGIANNI
Surgical Department.General Hospital of Larisa-Greece.

During the period of the last 13 years pancreatic and peripancreatic debridement combined with wide drainage to manage severe necrotic acute pancreatitis (AP) was used in 32 patients. At the same period we operated at our Institution on 2650 cases of benign diseases of the biliary tract. Among them 267 patients were operated for AP (10,07%) and the 54 of them (20,2%) had the necrotic severe type of the disease. The Ranson scores and Imrie scores confirm the initial severity of the attacks. Pancreatic necrosis was defined as the presence of necrotic areas entopancreatically or in the peripancreatic tissue, suspected clinically and confirmed by CT combined with intravenous contrast enhancement and operatively. Debridement and necrosectomy was done in different dates from the onset of the attack of AP. 7 patients (Group A) were operated from the 4th to the 8th day, 10 patients from the 9th to 20th, and 15 after the 21st day. The 3 groups were comparable. In case overwhelming sepsis was manifested the operation was urgently. The numbers of the operations, the days of hospitalization, the time of recovery and the final outcome were calculated with other parameters for the 3 groups. All were statistically better in the 3rd group. The mortality in group A was 57,7%, in group B 30% and in group C 6,6%. More tedious was the operation and we had more bleeding in the early operative group.

According to our experience we suggest the timing of debridement in AP, after the 21st day of the initial attack. As this time a clear plane of demarcation between necrotic and viable tissue, by formed granulation tissue was formed in the late group C, the necrotic material separated and the cavity develops well defined walls.

### P094

### THE ACTIVATION AND INACTIVATION OF KININ-KALLIKREIN SYSTEM IN ACUTE PANCREATITIS.

TH. PAVLIS, KR. DASKALAKIS, EV. ANAGNOSTOU, TH. MAYROMATIS, SP. MILINGOS.
3rd Surgincal Department, "Evangelismos" Medical Center
Athens, Greece,

One of the main enzymic systems activated in acute pancreatitis (AP) is the Kinin-Kallicrein system. Bradykinin is degraded by kininases. Kininase II, is an enzyme also known as angiotensin-1 converting enzyme (ACE).

In the last two years, ACE activity levels were investigated in 20 patients suffering from AP, at different stages of their disease. Serum ACE activity levels were found significantly increased on admission (accute phase of the disease) compared with ones found 4 days after their admission and ones obtained when the disease was subsided (post oral refeeding). The increased serum ACE activity levels found in the acute phase of pancreatitis represent probably a homeostatic responce of the organism to counteract the increased levels of bradykinin in AP.

We do not propose serum ACE activity as a diagnostic AP test, however is a quantitive expression of the Kinin-Kallikrein system activation and inactivation, system that participates in the disease's pathophysiology.

### P095

P095

### BIO-CLINICAL PROGNOSTIC FACTORS IN SEVERE ACUTE PANCREATITIS: A PROSPECTIVE, MULTICENTRIC STUDY

D. PEZET, N. ROTMAN, D. CHERQUI, C. CHASTANG, B. CHEVRET, P.L. FAGNIEZ

Hôpital Henri Mondor, Créteil, et Hôpital Saint-Louis, Paris, France.

From October 1986 to January 1991, the prognostic value of several bio-clinical parameters were studied in 234 patients with severe acute pancreatitis requiring admission in an intensive care unit. Bio-clinical parameters were collected on admission and at 48 hours. The collected data allowed to establish specific (Ranson, Glasgow) and non specific (MOF, SAPS) severity scores. Survival curves and curves of pancreatic abcess occurrence were constructed by the method of Kaplan-Meier and compared with the Log Rank test. A mutivariate analysis was made according to the Cox model. Complete Ranson score was available in 145 patients (mean: 3.9+/-2.0). Bio-clinical parameters influencing survival were: age>55 years, arterial pressure<120mmHg, BUN>9mmol/1, serum creatinine level> 120 µmol/1, LDH>1.5N, Pa02<60mmHg (p 0.05). Criteria influencing pancreatic abscess formation were: age>55 years, arterial pressure<120mmHg, temperature>38°C, Creatinine serum level>120 µmol/1, hematocrit <30% (p<0.05). The scores predicting survival were: SAPS (p<0.0006), Ranson criteria>3 (p<0.000I) and the Glasgow score (p<0.001). The occurence of a pancreatic abscess was correctly predicted only by the Ranson criteria (p<0.02). Independant variables influencing survival were age over 55 years and more than 3 Ranson criteria. Independant variables influencing the occurance of an abscess were: age, hematocrit value and glucose serum level. 11mmol/1.

### P096

### DEATHS DUE TO ACUTE PANCREATITIS

Z.Y.GU, K.H. ZHANG
Dept. of Surgery, Chinese PLA General Hospital, Beijing, China

From October, 1957 to March, 1985, 908 patients with acute pancreatitis (AP) were admitted to our hospital. 77 belonged to necrotic AP, 34 cases died, including 14 sudden deaths. Diagnosis was verified by autopsy in 24 cases, by surgical exploration in 8, and by other means in 2. It was found that the local pathological changes of pancreas were not so serious in the group of sudden deaths as in others. However, the systemic changes, ie, pneumonedema, rupture of the cardiac muscle, tonsillar herina and others, were more severe in the sudden deaths than the remainings. This is implicated that a systemic lethal factor released at the early stage of AP. We used systemic support of AP patients at the early stage and explored the patients only when complications ensued. Neither Ranson nor Imire risk factors was helpful in predicting sudden deaths of AP cases.

### P097

### THE EFFECTS OF SANDOSTATIN (SMS) ON PANCREATIC BLOOD FLOW (PBF), STRUCTURE AND FUNCTION DURING THE DEVELOPMENT OF EXPERIMENTALLY INDUCED ACUTE PANCREATITIS (AP).

SA JENKINS, S ELLENBOGEN, H KYNASTON, N DAVIES, R SUTTON, N ROBERTS AND JN BAXTER
University Department of Surgery, Liverpool, UK

Since there is little data on the temporal changes in PBF during the development of AP, we have carried out such a study in rats and investigated the effects of SMS. AP was induced in male Wistar rats by common bile duct ligation and an i.v. infusion of Caerulin (5ug/kg/h) for 2h. Control rats received a s.c. injection of saline immediately before and 8h after the induction of acute pancreatitis and the experimental group 2µg of SMS s.c. Organ blood flow (microsphere method), serum amylase levels and pancreatic structure were determined 4 and 16h after the start of the study 4h after the induction of AP the pancreas in the controls was oedematous and all displayed marked hyperamylasaemia. At 16h the pancreas in the controls was haemorrhagic and necrotic but the serum amylase levels were decreased. SMS ameliorated both the change in pancreatic structure and function at 4 and 16h. SMS significantly reduced (p<0.01 ANOVA) PBF (2.3ml/min/g dry wt.) compared to control animals at 4h (4.1 ±0.7), but maintained pancreatic perfusion (2.1 ±0.4) compared to the reduction observed in the controls (0.6 ±0.9) at 16h (p<0.001). The results of this study indicate that SMS prevents early hypofusion and late hyperfusion during the development of AP in the rat. Further studies are required to determine whether the changes in PBF elicited by SMS and responsible for its protective effect on pancreatic function and structure.

### P098

### SANDOSTATIN (SMS) AMELIORATES THE DELETERIOUS EFFECTS OF EDOTOXIN ON PANCREATIC STRUCTURE, FUNCTION AND BLOOD FLOW IN RATS

SA JENKINS, H. KYNASTON, N DAVIES, J YATES, N ROBERTS, K PARSONS AND BA TAYLOR
University Department of Surgery, Liverpool, UK

Previous studies have shown that experimentally induced pancreatitis results in a systemic endotoxaemia which is ameliorated by SMS. The aim of this study was to establish whether endotoxaemia resulted in changes in pancreatic structure, function and blood flow in the rat and whether SMS influenced any of these effects. Groups of male Wistar rates received i.p. endotoxin (2mg/100g) or saline. Half of the animals in each group received SMS (2µg s.c) immediately before and 12h after administration of endotoxin or saline. Organ blood flow (microsphere method) hepatic reticuloendothelial system (RES) activity (liver: blood 99 Tc sulphur colloid) serum amylase and pancreatic structure were determined 16h after the administration of endotoxin or saline. Endotoxin administration resulted in an oedmatous haemorrhagic pancreatitis which was prevented by SMS. Pancreatic blood flow (1.06 ±0.6 V 2.4ml/min/g dry wt; p<0.01) and hepatic RES activity (4.33 ±0.44 v 6.3 ±0.23; p<0.001 ANOVA) were significantly less in endotoxin treated rats compared to those treated with endotoxin plus SMS. SMS also prevented the hyperamylasaemia observed in endotoxin treated rats. The results of this study indicate that endotoxin has a deleterious effect per se on pancreatic structure, function and blood flow in the rat which are ameliorated by SMS, thereby offering a partial explanation, for its beneficial effect in experimentally induced acute pancreatitis.

### P099

### THE EXPERIENCE OF TREATMENT OF THE PANCREATIC PSEUDOCYSTS IN TALLINN STATE HOSPITAL

M. EIVIN, J. LIND, A. MÄESALU, A. JOSING
Tallinn State Hospital, Tallinn, Estonia

During the period from 1974 to 1990 we have treated 53 patients with pancreatic pseudocysts. They were divided into 3 groups - I Postnecrotic pseudocysts (41); II Retention pseudocysts (7) III Postnecrotic pseudocysts, having a communication with the pancreatic duct (5).

The patients were treated as following-

1. External draining I gr-33, III gr 2 times
2. Cystodigestive anastomosis I-3, III - 1 times
3. Distal pancreatic resection I - 4 times
4. Whipple's maneuvre II - 2 times
5. Pancreatodigestive anastomoses I - 1, II - 5, III - 2 times.

Altogether we had 3 lethal results (5,6%).

As a conclusion we can say, that for acute pseudocysts external draining is usually curative, for retention pseudocysts surgical manipulations should address the ductal pathology.

### P100

### GABEXATE MESILATE VS APROTININ IN THE TREATMENT OF ACUTE PANCREATITIS. RESULTS OF A MULTICENTRIC TRIAL

P. PEDERZOLI, C. BASSI, S. VESENTINI, R. GIRELLI, M. FALCONI, D. LOMBARDI, H. ABBAS, A MACCAGNANI, A BONORA AND G. CAVALLINI*
Surgical and *Medical Department, University of Verona.

In order to verify the efficacy of Gabexate Mesilate (FOY) versus Aprotinin (A), a prospective multicenter randomized double-blind trial was carried out in moderate-severe acute pancreatitis (AP), using FOY 3g/day vs A 1500000 UIK/day for seven days. 199 pts suffering from AP were enroled by 34 centers from Jan '89 to Dec '90. The main admission criteria were: onset from not more than 72h, at least 2 Ranson positive criteria and CT evidence of acute pancreatic damage. In the 182 analyzed pts (106 male, 76 female; mean age 57.6 years) the etiology was: biliary 56%, alcoholic 25.8%, post-operative 7.1%, unknown 7.1%, others 3.8%. Median Ranson' score was 3. Edema was detected by CT scan in 66 pts; necrosis in 116 (63.7%). 91 pt were treated with A and 91 with FOY. The main prognostic factors were comparable in the two groups. Every pts was followed-up for 8 days after end of the treatment to assess the development of systemic complications (c), pseudocysts (p) and early sepsis (s), need of surgery (h) and mortality rate (m). A long term follow-up was then performed at 3 months: 7 pts out of the 116 necrotic cases died within 15 days (6%) with an overall incidence of c of 14.7% (23.5% treated with A vs 7.7% with FOY: Chi-square test p 0.05). h was necessary in 25.5% in A group and 10.8% in FOY group (Chi-square test p 0.05). No statistical differences were found in p,s and m also at the 3 months follow-up. These data suggest that, in the early phases of AP, FOY treatment is more effective than A in reducing c and h.

### P101

### C-REACTIVE PROTEINE AND C3-C4 FACTORS OF COMPLEMENT CHANGES, IN PROGNOSIS OF ACUTE PANCREATITIS

G. PERPIRAKIS, M. VELEGRAKIS, N. NIKOLAKAKIS, F. SEFERIADIS, S. KANDILAKIS, N. PARASKAKIS.
Department of Surgery Gen. Rethimnon hospital, Crete Greece.

The prognosis of an acute pancreatitis attack (AP) is decisive for its proper treatment. For this reason besides the multiple factor scoring systems there has been an effort to identify a simple biological index which will discriminate with accuracy the cases of mild severity from those of high severity. We studed the CRP and $C_3$-$C_4$ changes in 54 patients with (AP) during the day 1,2 and 7. The results indicate that the high levels of CRP which remain 48 hours from the the onset can precisely discriminate the cases of mild severity from those of high severity, $p<0.001$. Similar results were observed in the fluctuations of $C_3$ ($p<0.001$) amongst complicated and non complicated cases during all measurements. $C_4$ changes on day 1, were different between the two groups ($p<0.001$) while the day 2 and 7 non significant differences was observed (p=NS). In conclusion the determination of CRP and $C_3$ fraction of the complement on day 1,2 and 7, can precisely identify the cases with complication tendencies from those with satisfactory outcome.

### P102

### ETIOLOGICAL FACTORS OF ACUTE PANCREATITIS IN SURGICALLY TREATED PATIENTS

TH. POLIMEROPOULOS, E. YETTIMIS, P. VACHLIOTIS, E. KALOKERINOS, H. TSIPRAS, H. COSTOPANAYIOTOU
1st Dep. of Surgery, Athen's General Hosp., Greece

The examination of the etiological factors of acute pancreatitis (A.P.) in 223 surgically treated patients is the aim of the study. The morphologic pictures seen during operation were: steatonecrotic alterations (144 pts) pancreatic abscess (43pts) and pancreatic pseudocyst (36 pts). The most common causative factor was biliary tract disease, present in 203 pts (91.0%). Lithiasis was responsible for A.P. in 163 pts. In 40 pts with no stones in the billiary tract, cholesterolosis of gallbladder's mucosa was found and this was confirmed by microscopic examination. It was also found in 38 pts with lithiasis. The total incidence of cholesterolosis was 38.4% and was associated with 13.2% of steatonecrosis (18 pts), 33.3% of pancr. abscesses (12 pts) and 32.2% of pancr. pseudocysts (10 pts). Other etiologic factors were: alcoholism (3 pts), familiar lyperlipedemia (2 pts), surgery (3 pts) and unknown in 8 pts. Conclusions: (a) Biliary tract lithiasis is the main cause of A.P. in Greece (b) Cholesterolosis of gallbladder's mucosa is a serious cause of A.P. and is related with severe complications. (c) It is wise to perform cholecystectomy whenever A.P. is treated by surgery even if there are not palpable stones.

**P103**

### TIMING AND EXTENT OF SURGERY FOR GALLSTONE PANCREATITIS (GP)

H. POOLA, J. TROOST, A. VIIKLEPP, P. ALAS
Dept. of Surgery, Tallinn Central Seamen's Hospital, Estonia

Timing and extent of surgery for GP remain controversial. A retrospective analysis of the results of 74 consecutive patients, operated on for GP during the same admissions presented. Choledocholithiasis was in 43.2%, pancreonecrosis in 27%, destructive cholecystitis in 16.2%, suppurative cholangitis in 8.1% and diffuse peritonitis in 8.1% of cases. Mean age 55 years, 86.5% female.6 patients died (8.1%) and morbidity was 13.5% (10 patients). In group (gr) 1 (n=27) early surgery was performed during 3 days in patients who failed to respond to intensive treatment. From this gr 13 patients needed immediate surgery and there were 2 lethal cases with acute haemorrhagic pancreatitis. Mortality in gr 1 was 7.4%. In gr 2 (n=47) early delayed surgery was used. From this group 18 patients had exacerbation of disease after temporary subsiding and they had to be operated on emergently. All 4 deaths in gr 2 (8.5%) occured with latter patients reason of death being a large myocardical infraction in 1 and progressive peripancreatic necrosis in 3 cases. Cholecystectomy was performed in 91.9%, choledochotomy in 41.9% and various decompression drainage in 87.5% of cases. Operations on the pancreas were carried out in 14.9% of cases. Timing and extent of surgery depend on severity of case and its response to conservative treatment. It's sometimes difficult and unrealistic to plan the time of performing surgery.

**P104**

### THE ROLE OF PANCREATIC RESECTION FOR SEVERE ACUTE NECROTIZING PANCREATITIS

B.M. WANG, Y. TANG, X.G. HU, M.C. WU
Department of Surgery, First Teching Hospital (Chang Hai)
Second Military Medical University, Shanghai, China

Acute necrotizing pancreatitis accompanied by any one of the following conditions, such as shock, respiratory or renal failure, GI bleeding, psychic disturbance, and operative finding or CT scan showing extrapancreatic infiltration and area of necrosis greater than 2/3 pancreas, is classified as having the severe form of this disease. From January 1976 to December 1990, 25 patients fullfiled the above criteria. Eight patients were managed conservatively, and 4 died with a mortality of 50%; while 17 patients were managed by vigorous supportive therapy followed by resection of the clearly demaarcated portion of pancreatic necrosis: 12 by resection of the tail, 4 by resection of the body and tail, and 1 by resection of the head. Intra-abdominal abscess, colonic or pancreatic fistula, intestinal obstruction, and GI bleeding occurred postoperatively in 7 patients. Five patients (29.4%) died between several days and weeks postoperatively; death being due to sepsis and multisystem failure in 2, respiratory failure, massive intraperitioneal hemorrhage, and septic shock in each one of the other 3, respectively. Of the 12 survivals, 1 requires insulin. During the same period 59 patients with different severity of the disease were managed by simple capsulotomy plus drainage, necrosectomy plus drainage, or necrosectomy plus local lavage, respectively. Twenty patients died postoperatively with a mortality of 33.89%. Although the optimum therapy of this disease is still controversial, necrosectomy plus local lavage seems to be the recent trend of managment due to its lower mortality and morbidity. Nevertheless, the authors believe that the optimum therapy should be highly individualized. Timely resection of well demarcated portion of necrotic pancreas may still play a role in some carefully selected patients to salvage those who are otherwise succumbed.

**P105**

### POSTOPERATIVE PANCREATIC AND PERIPANCREATIC ABSCESSES OF ACUTE HEMORRHAGIC -NECROTIZING PANCREATITIS

YANG SEN HUA, YANG YI CUN,
Chengdu Third People's Hospital, Chengdu, Sichuan, China.

Postoperative pancreatic and peripancreatic abscesses (PPA) of acute hemorrhagic-necrotizing pancreatitis (AHNP) are often difficult to prevent, diagnose and treat effectively. Incidence of PPA is very high. PPA occured in our 28/77 cases (36.36%). Its diagnosis depended on the properties of peripancreatic exudates, its culture and pathology, image means and exploration. PPA, the so-called engymatic abscess coexisted with numerous other serious complications, might be the cause of some of the latter such as hyperglycemia. MOF, etc. Overall mortality was 51.14% (16/28 cases) - those of operative and non-operative treatment were 50% (5/10) and 61.11% (11/18) respectively. Expect operative procedures there were no significant differences between groups of various related surgical situations. As yet how to lower the incidence of PPA is still very difficult due to some proper belonged factors such as the lingering course of pancreatic inflammation. No specific clinical manifestations of PPA and diverse display of complications making diagnosis difficult, continuous BUS and CT might be helpful. The treatment of PPA cannot be too emphasized on surgical drainage, often for several times.

**P106**

### SURGICAL TREATMENT OF ACUTE HEMORRHAGIC-NECROTIZING PANCREATITIS IN THE AGED

YANG SEN HUA,YANG YI CUN
Chengdu Third People's Hospital, Chengdu, Sichuan, China.

How about the surgical treatment of acute hemorrhagic-necrotizing pancreatitis (AHNP) in the aged? Among the 55 cases treated surgically the age of 20 cases (36.36%) was above 60. 6 cases were operated upon before 43 hours and 14 after 49 hours after onset of AHNP. The operations included capsulectomy and drainage (12 cases), necrosectomy or regular resection of the pancreas and peritoneal and pancreatic bed lavage (8) with gastrostomy, jejunostomy and cholecystostomy or choledochostomy or both(6). Postoperative complications were numerous and serious - the chief causes of death. The mortality was 60% (12/20 cases), though very high, yet not significantly higher 45.71% (16/35 cases) of the younger (p>0.05). We prefer earlier timing for operation when necessary and choose easier and more conservative operative procedure - necrosectomy as basic operation. For putting the pancreas in good rest, triple-stomies seems worth-while to adopt. It is very important for improving curative effect to manage the complications intensively.

### P107

### PLASMAPHERESIS IN ACUTE NECROTIZING PANCREATITIS: CIRCULATORY EFFECTS - AN ANIMAL STUDY

H. ZIRNGIBL, S. MANN
Department of Surgery, University of Regensburg, FRG

Acute necrotizing pancreatitis (a.n.p.) still causes a high lethality from 60 to 80 %.

After activation of enzymes, complement system and coagulation system multiple organ failure (MOF) develops. Surgical therapy is not able to change fatal outcome in the socalled vasoactive phase. In an animal experiment we have investigated the effect of extracorporal detoxication with plasmapheresis in taurocholat induced a.n.p. in the dog.

21 male dogs were randomized in 3 groups. 8 with plasmapheresis and a.n.p., 8 only with a.n.p. and 5 sham. Circulatory monitoring, using Swan-Ganz-Catheter measuring CO, PCWP, CVP and peripheral resistance, arterial blood pressure was installed about 9 hours. 2 hours after steady state a.n.p. was induced. Observation started for a 7 hour period. Afterwards lung and heart were prepared for histopathological investigations.

We saw the very best effects in circulatory system in the group of animals treated by plasma pheresis. Also histopathological investigatins showed, that plasmapheresis is able to reduce toxic effects of pancreatitis in the lung.

We conclude, that plasmapheresis is able to reduce systemic toxic effects and is helpful in the management of a.n.p. in the first vasoactive phase of this disease.

### P108

### OUR EXPERIENCE IN THE TREATMENT OF ACUTE BILIARY PANCREATITIS

VIACHKI IV., N.JARAMOV, N.PENKOV, D.VIACHKI
BULGARIAN ACADEMY OF MEDICINE
Centre of Urgent Medical Aid

The authors have studied 358 cases of acute biliar pancreatitis. Among them 192 men and 166 women. In all the cases in the complex medical therapy the following was included: Ftorafur by scheme, forced diuresis, aqua-physiological and protein solutions, depending on the ionogramme, proteinogramme, the central venous pressure, diuresis, as well as stopping of the pancreatic pain.

In 132 of all the cases endoscopic papilosphincterotomia was carried out. From all the patients only 71 were operated, and for 10 of them splanchicectomia was done.

The authors proposed practical conclusions on the basis of the analysis of the cases.

### P109

### OCTREOTIDE IN THE TREATMENT OF POSTCHOLECYSTECTOMY ACUTE PANCREATITIS

V.PERCOPO,L.SORRENTINO,F.P.LEZZOCHE,S.PERCOPO,B.TESAURO
1st Surgery Dept.2nd Medical School, University of Naples

We want to value the octreotide effectiveness in treatment of postcholecystectomy acute mild pancreatitis (PAMP) (amilasemia:300-10000). Study has been performed about a uniform pattern of 30 females, age average 48.2 (range 25-65). Cholecystectomies have been performed by the same surgical team. Patients have been stratified into 3 groups: A) 10 patients affected by PAMP treated with i.v. continuous infusion of natural somatostatin (0.25 mg/h) for 7days; B) 10 patients with PAMP treated with i.v. octreotide (0.3 mg/day) followed by the same dosage s.c. from the day of canalization and for total 7 days; C) 10 patients with out PAMP (contol group). Therapeutic effectiveness has been valued on the basis of clinical symptoms (pancreatic pain), seric and urinary amilase levels, canalization time. Pancreatic pain has been decreased in A and B groups within 48 h, but more quickly in B group. Seric and urinary amilase levels has become regular within 7 days in A and B group, however decrease has been faster and higher in patients treated with octrotide. S Statistical differences between two groups aren't significant yet. Canclization average time has been 68h in A group, 78h in B group,50,5h in C group. Longer canalization time in B group compared with A and C groups is due to greater powerful of octreotide. These data proved that natural somatostatin and octreotide have an overlapping usefulness in the treatment of PAMP. It is to be under lined, however, the better compliance of the octreotide because of: subcutaneous administration, shorter time of parenteral nutrition, economic advantages.

### P110

### MANAGEMENT OF PERIAMPULLARY CARCINOMA

P. DECKER, U. KANIA, D. DECKER, A HIRNER
Bonn University, Surgical Department, Sigmund-Freud-Str. 25, 5300 Bonn 1, Germany

The survival rate of patients having a periampullary carcinoma depends on histology, tumor size and patients age.

There are different possibilities of treatment:

- pancreaticoduodenal resection (Whipple's operation)
- local excision of the tumor
- biliodigestive anastomosis

In contrast to the carcinoma of the pancreas with a five-year survival rate of 4.9%, a better healing rate can be achieved in periampullary carcinoma. The five-year survival rate of 4.9%, a better healing rate can be achieved in periampullary carcinoma. The five-year survival rate following local excision about 18.6%, whereas the five-year survival rate following pancreaticoduodenal resection about 50%.

The following concept of surgical treatments should be discussed:

1. In old patients having a lot of risk factors a local excision of the tumor should be the treatment of choie.

2. The smaller the carcinoma the more "radical" should be the surcical treatment.

3. The better the histopathology (for example: carcinoma of the Papilla Vateri) the more radical should be the treatment.

In the University Boon three patients were treated according to this surgical management in the last year. Twice the patients were treated by Whipple operation, one 77 year-old patient had a local excision with biliodigestive anastomosis.

### P111

### WHIPPLE'S OPERATION IN THE TREATMENT OF COLON CANCER WITH DUODENAL INFILTRATION

M. MORENO, J.C. RUIZ ADANA, C. CERQUELLA, J.M. FRADEJAS, A. CARABIAS y G. PEREZ
Hospital Universitario de Getafe. Getafe. Madrid.

Colonic neoplasm in the hepatic flexure of the colon infiltrates and fistulize to the duodenum in 19-25% of cases. These tumours can reach a high volume and in absence of liver metastases they cannot be considered as non resectable and must be treated with extended surgery with oncological criteria.

For the treatment of these tumours in 1960 GALLAGHER proposed the term of Extended right colectomy that includes a cephalic duodenopancreatectomy. This form of treatment has not been widely used and few cases have been treated.

Two cases are presented. The first one corresponds to a patient of 67 years of age with colo-duodenal fistula and the other one to a 59 years-old male wide with infiltrationof the duodenum. Both cases were treated with extended right colectomy and in the latter a wedge liver resection was performed because of infiltration of the liver.

Postoperative course was uneventful and the survival is 6 years in the first case and one year in the second. This last case died of recurrence.

Postoperative course was uneventful and the survival is 6 years in the first case and one year in the second. This last case died of recurrence.

Extended surgery can be safely performed in these patients and long term survival is possible as it occured in our former patient.

### P112

### CLINICO-PATHOLOGIC STUDY OF CARCINOMA OF THE PAPILLA OF VATER.

MORI K, NAGAKAWA T, TAKEDA T, NAKANO T, KAYAHARA M, OHTA T, UENO K, MIYAZAKI I
Department of Surgery (II), School of Medicine,
Kanazawa University, Kanazawa, 920 Japan

A total of 34 cases of carcinoma of the papilla of Vater were studied by preparing 5mm step-wise whole tissue sections from each patient in order to clarify the tumor's extension histopathologically. The 5-year survival rate was 47.0%. The 5-year survival rate for the patients with lymph node metastases was significantly worse than that for those without lymph node metastases ($P<0.05$). Lymph node metastases were located primarily in the retropancreatic area and around the superior mesenteric artery. There was a long-term survivor who had lymph node metastases at the first and second barrier. He received complete dissection of the first and secoond barrier lymph nodes. We suggest that dissection of the second barrier lymph nodes, especially around the superior mesenteric artery, is a necessary component of radical surgery for carcinoma of the papilla Vater.

### P113

### ENDOSCOPIC RETROGRADE CHOLANGIOPANCREATOGRAPHY IN THE DIAGNOSIS OF PAPILLARY TUMORS

M. RUBINIC, M. IVANIS, M. URAVIC A DEPOLO,
University Medical Center, Rijeka, Croatia.

The introduction of ERCP, and in recent years also of endoscopic ultrasonography, presents a great step forward in the diagnostics and therapy of tumours of the ampular region. In a five-year period 1370 examinations of this kind were performed. The patients were divided into two groups. In 610 patients (Group A) indications for ERCP was obstructive jaundice, tumors of the papilla of Vater were found in 12 (1.2%). In 760 patients (Group B) with other various biliary and pancreatic disorders papillary tumor was found in only one female patient. Summarized results showed that incidence of papillary tumors in series of 1370 ERCP-s was 13 (0.9%)

This paper points out the importance of ERCP in diagnosis of papillary tumors and helps in planning further treatment, which may be palliative as well as more radical surgical procedures.

### P114

### GUIDELINES FOR PERCUTANEOUS CATHETER DRAINAGE OF PANCREATIC PSEUDOCYSTS

A. D'EGIDIO
Department of Surgery, University of Witwatersrand, Johannesburg, South Africa.

Between November 1987 and April 1991 we submitted to percutaneous catheter drainage (PCD) all patients with post-necrotic cysts (due to autodigestion and extravasationof pancreatic juice). There were 16 type I cysts (in acute pancreatitis) and 10 type II cysts (in chronic pancreatitis). Overall recurrence rate was 4%, three patients had fistulization into bowel; no deaths occured. PCD was successful in all type I cysts; in type II cysts was associated with prolonged drainage and complications when cyst-duct communication was present.

INDICATIONS FOR PCD: 1) immature symptomatic cysts 2) enlarging cysts 3) infected cysts 4) large cysts > 8 cm. 5)cysts not resolving.

CONTRAINDICATIONS: 1) cysts associated with ductal stricture 2) cysts not accessible to percutaneous route 3) doubtful diagnosis 4) presence of infected pancreatic necrosis.

PROTOCOL FOR PCD: 1) CT scan prior to catheter insertion 2) fluid for cytology, microbiology and amylase 3) catheter in situ until drainage stopped 4) sinogram through the catheter prior to its removal 5) E.R.C.P. prior to PCD in cases of chronic pancreatitis, in acute pancreatitis when indicated.

During this period no surgical internal drainage procedures performed in our unit.

**P115**

### RESECTIVE AND DERIVATIVE SURGERY FOR CHRONIC PANCREATITIS

G.R. FRONDA, S. ENRICO, M.P. CAPOZZI, M. TOPPINO, U. CATTANEO
Department of Surgery. University of Torino (Italy)
(Head: Prof. F. Morino)

Surgery for Chronic Pancreatitis (CP) is recommanded for intractable pain and complications. The choice of the type of operation (resection vs. derivation) depends on the extension of the lesion, the type of complications and the morphology of the Wirsung. During the period 1976-1991, a total of 111 operations for CP were performed in 98 pts. (m.85, f.13). The mean age was 47.6 years (31-72). Alcoholic intake was positive in 73 cases (74.5%). All the pts. had intractable pain, while pseudocysts were present in 67 (68.3). Fifty-three were the derivative operations (28 Wirsungo-jejunostomy; 19 cysto-jejunostomy; 6 cystom-gastrostomy) 34 the resection (21 spleen-pancreatectomy; 4 distal pancreatectomy; 9 duodenopancreatectomy) and 24 other different interventions of which 14 bilio-digestive anastomosis. The mortality rate was 18.3% (18 pts.); the postoperative complications were 13 (11.7%), on the other hand late complications occurred in 9 pts. and required 13 interventions. The average follow-up was of 75.8 months (6-168) and concerned 64 pts.; we observed a good resolution of the pain in 54 pts. (84.3%). Pseudocyst or Wirsung drainage is the goal of derivative surgery, meanwhile resection is indicated for irreversible lesions of the Pancreas. In our experience, the Partington-Rochelle's intervention preferred, because it permits to spare pancreatic tissue and an eventual subsequent intervention; it's also characterized by minor mortality and complications. Cystic drainage hasn't given good results, probably because it has't drained a sufficient portion of the gland. For this motive, for some years, we have abandoned these techniques since valid radiologic methods have been developed such us U.S. o C.T.-guilded drainage or Percutaneous Pancreatic Cystogastrostomy. Resection has been reserved to those cases with complications not otherwise resolvable, or in the case of derivation failure. We have nearly always performed a distal pancreatectomy - with or without splenectomy - with acceptable long-term results as for as pain is concerned. We still prefer the Duodenopancreatectomy in the cephalic forms.

**P116**

### MAJOR BLEEDING COMPLICATING PANCREATITIS: ETIOPATHOGENESIS AND RISK FACTORS

G. GLATI, B. POROWSKA, P. NEGRO, D. FLATI, M. CATARCI, P. TORDIGLIONE AND M. CARBONI
V. Surg. Path. - University of Rome "La Sapienza" - Italy

Massive haemorrhage, although rare, represents one of the most feared sequelae of pancreatitis. On the basis of a personal experience of three cases and of a comprehensive review of 277 cases reported in the literature during the last century the AA hereby discuss the etiopathogenesis and the most important risk factors of this rather uncodified pancreatitic complication.

Major bleeding may complicate the course of either acute or chronic pancreatitis, the latter being more frequently involved. Pseudocysts, severe inflammation, regional necrosis and infection may cause major vessel erosion with or without pseudoaneurysm formation which eventually may result in severe bleeding into the gastrointestinal tract, retroperitoneum and peritoneal cavity. Mortality rate was found to be related to the etiology of the bleeding along with its localization and the underlying anatomo-pathologic findings. In patients with chronic pancreatitis it was 27.4% while in patients with acute panoreatitis or chronio pancreatitis with acute exacerbation it was 61.4% and 57.1% respectively. Splenic, gastroduodenal and inferior pancreaticoduodenal arteries were the most commonly involved vessels being associated respectively with a mortality rate of 21.5%, 38.4% and 61.1%. Massive haemorrhage complicating infected necrosis or abscesses implies a worse prognosis when compared to bleeding associated with pseudocysts with or without pseudoaneurysms (mortality rates being 53.8% and 50% vs 37.1% snf 26.9% respectively).

In conclusion the AA claim that the knowledge of the etiopathogenetic mechanisms and risk factors involved (infected necrosis, insufficient debridment, abscesses, long standing pseudocysts), might contribute to improve the prognosis of the most dramatic of pancreatitis.

**P117**

### HISTOPATHOLOGICAL CHANGES OF THE LIVER IN CHRONIC PANCREATITIS.

RP JALLEH, JA GILBERTSON, CS FOSTER & RCN WILLIAMSON
Department of Surgery and Histopathology, RPMS, Hammersmith Hospital, London, United Kingdom.

Many patients with severe, alcohol-related chronic pancreatitis appear to have normal livers at operations. We report the histological appearances of intraoperative liver biopsies in a series of 52 patients (40 men, 12 women; mean age=41 years) with histologically proven chronic pancreatitis. Alcohol was the aetiological agent in 40 patients with an estimated daily consumptionof 216±32g for 12.8±1.4 years (mean±SD). Causes in the other 12 patients were previous acute pancreatitis (5), choledochal cyst (1) and unknown (6). Liver function tests were abnormal in only 7 patients. Histological changes included steatosis (n=18), intrahepatocyte iron (11) and copper (2). Cholestasis was present in biopsies from 5 patients with extrahepatic billiary obstruction. Fibrosis cirrhosis and Mallory's hyaline were not seen. Immunohistochemistry revealed a dense portal lymphoid infiltration in 47 specimens (90%) comprising T-lymphocytes (45±5 cells per portal tract). B-lymphocytes (10±3 cells per tract) were seen in only 8 biopsies, macrophages (43 specimens) had a parenchymal distribution T-lymphocytes were more common in alcoholics than non-alcoholics (95 vs 75%, $p<0.05$). All other histological were similar in the two groups. The minor hepatic changes and protal infiltration seen in chronic pancreatitis of all aetiological types suggest migration of inflammatory cells from the pancreas rather than a direct hepatotoxic process.

**P118**

### SONOGRAPHIC FOLLOW-UP OF CYSTOGASTROSTOMY FOR PANCREATIC PSEUDOCYST

P.G. SFIKAKIS, J.A. BIRIS, S.B. KONTOSTOLIS, P.J. TZARDIS, N.L. LEKACOS
2nd Surgical Unit, Tzanion Gen. Hospital, Piraeus, Greece

We present our experience on postoperative sonographic imaging and evaluation of transgastric cystogastrostomies following elective surgery for pancreatic pseudocysts. Our method consists of performing an "acoustic" window, by giving the patient 500cc of water to drink prior to examination, which fills the lower half of the stomach as well as the residual pseudocyst cavity (RPC) and the patient is examined in the prone or erect positions, with transducers of 3 and 3.5MHz.

Sixteen patients aged 48-76 were examined at the 10th, 60th and 120th post.op.day and the results were compared to simultaneous CT-scanning. In 94% of our cases the RPC had disappeared by the 120th day, and one patient is still under evaluation and his RPC has greatly dicreased. The distention of the stomach by water, improves visualization of the body and tail of the pancreas, overcoming the problem of bowel gas the presence of which thwarts the ultrasonographer.

Thus, our method is less costly, easily and rapidly performed, with relatively high specificity and sentitivity and is considered to be the gold standard in evaluating patients after cystogastrostomy, compared to the CT-scan

**P119**

### ULTRASOUND CONTROLLED CLEAVAGE OF THE PANCREATIC DUCT: less operation time, less trauma

J. H. SIMANOWSKI, V. MENDEL, TH. REUHL, H. HEYMANN
Klinik und Poliklinik fuer Allgemeinchirurgie der Medizinischen Hochschule Hannover, Podbielskistr. 380, D–W 3000 hannover 51, Germany

Introduction: The detectionof a dilated duct in a chronically inflamed calcified pancreas can be a time consuming task for the surgeon, while blind puncture with a needle may cause a traumatizing failure.

Methods: A sterilized 5 MHz linear array transducer (Siemens AG, Germany) was put crosswise and directly on the pancreas. Under ultrasound control a needle was positioned into the duct and the cleavage was performed using a cautering scalpel. Meanwhile we possess a specially designed puncture setup which allows a stable guidance of the needle while under ultrasound control.

Results: In 21 cases of pancreatico-jejunostomy we did an ultrasound controlled cleavage of the pancreatic duct. 1-2 trials took 2-4 minutes to puncture the duct. No mispuntures or bleeding were caused and all patients recovered fast from the operation.

Conclusion: Ultrasound controlled cleavage of the pancreatic duct reduces operation time as well as the trauma caused by mispuntures and is therefore highly recommended.

**P120**

### THE SURGICAL TREATMENT OF THE CHRONIC PANCREATITIS

F.I. TODUA, G. SH. SVANIDZE
Regional Scientific-Research Clinical Diagnostic Centre, Tbilisi, Georgia

The aim of this study is the perfection of the method of surgical treatment of the patients affected with chronic pancreatitis. 148 cases out of the 191 cases of the patients affected with pancreatitis, were chronic alcoholics. 102 cases were affected with calculus of the pancreas and its ducts. In 48% of patients after US and CT control were noticed widening of the ducts and the cystic widening of the ducts. On 91 patients with chronic calculus pancreatitis were performed operations with the internal drainage of the pancreatic ducts pancreatojunoanostomosis. In 18 cases anastomosis was combined with the distal resection of the Pancreases to 20 patients affected with the chronic calculus pancreatitis with the cyst of the head of the pancreas, the marginal cystopancreatojunoanastomosis was performed. In 26 cases the external drainage through anastomoses was added to anastomosis. To 4 patients affected with chronic calculus alcohol pancreatitis, a distal resection was perfomed. The leading method of surgical treatment of the chronic pancreatitis is the operation, with the internal drainage of the pancreatic ducts. The surgical treatment of chronic alcholic pancreatitis gives us good results in 78% of the cases.

**P121**

### THE CLINICAL MORPHOLOGICAL FINDINGS OF SCLEROSING PANCREATITIS WITH CHOLANGITIS MIMICKING PANCREATIC CARCINOMA

MARRANO D., SANTINI D.,* CAMPIONE O., CASADEI R., SALERNO A.,* PASQUALINI E., GRECO V.M.
Ist. I^Clinica Chirurgica Generale e Terapia Chirurgica Università di Bologna (Italy)
(*) Ist. Anatomia Patologica - Università di Bologna (Italy)

Primary sclerosing cholangitis (PSC) is characterized by a chronic fibrotic inflammation of bile ducts involving the entire extra and intrahepatic system. The involvement of the pancreas in PSC is extremely rare and histologically poorly documented. We present the detailed clinical-pathological features of an unusual chronic pancreatitis with sclerosing cholangitis involving extensively the pancreas which clinically appeared undistinguishable from pancreatic cancer. A 35 old man, with no history of alcohol or drug abuse, presented with sudden and increasing jaundice. Ultrasonographic and computed tomographic examination showed a cephalo pancreatic mass with marked stricture of choledocus. A pancreatico-duodenectomy was performed. Grossly the pancreatic head was firm with a mass-like enlargement. The bile ducts were fibrously thickened. Histological findings showed interstitial fibrosis, acinar atrophy and marked periductal lymphoplasmocitic inflammation, diffuse infiltration within and around pancreatic ductal tree by neutrophilic leucocytes and vasculites. Sclerosing pancreatitis with diffuse cholangitis seems to be the better term for this condition. Tests for antinuclear antibodies was positive while antimitocondrial antibodies and the LE cell test were negative. All serological examinations for infectious diseases were negative. In conclusion we report our experience with an unusual case of chronic pancreatitis suggesting that this form may represent a separate entity. Some clinical-pathological features lead us to consider this lesion as a rare variant of PSC with extensive involvement of the pancreas.

**P122**

### FOLLOW UP RESULTS OF PANCREATICOJEJUNOSTOMY IN CHRONIC PANCREATITIS

H.KOHLER, FE.LUDTKE, R. NUSTEDE, A.SCHAFMAYER, HJ. PEIPER
Dept. of General Surgery University of Goettingen, Goettingen, FRG

Between 1968 and 1981 in 138 patients (101 male, 37 female, mean age 41.2 years) a side-toside pancreaticojejunostomy was performed. Follow up studies were performed in 95 patients, mean follow up period was 10 years (4-14). Late mortality was 19%, general condition improved in 62%, pain relief was observed in 65% of the patients. Latent or manifest diabetes occured in 21%, stool fat changed to normal in 30%, relapses of pancreatitis occured in 41% of the cases. In general, results were good in patients who denided alcohol postoperatively.

### P123

### SOLID AND PAPILLARY NEOPLASM OF THE PANCREAS. REPORT OF A CASE

R. CHARCO, I. DIAZ, A. EDO AND C. MARGARI
Liver Transplantation Unit. Department of Surgery. Hospital General Vall d'Hebron. Universidad Autónoma de Barcelona, Spain

Solid and papillary neoplasm of the pancreas is an extremely rare and not widely known tumour with predilection for young female patients. Its low incidence ranges from 0.17% to 2.7% of nonendocrine pancreas cancers. Only 27 well-documented cases have been reported in the literature. Its histogenesis remains a matter of controversy.

We report a 28-year old woman with a 1-year history of epigastric pain. Clinical examination was normal. CT-scan revealed a tumour with solid and cystic areas located in pancreas tail.

The patient underwent distal pancreatectomy and esplenectomy. Postoperatively, low flow pancreatic fistula was resolved in one week. Grossly, the tumour appeared well circumscribed and measured 10 cm in diameter. On cross section, it was multilocular with yellow solid areas discolored from haemorrhage. Microscopically, the tumour was composed of polygonal cells with eosinophilic granular cytoplasm and round to oval nuclei with a low mitotic rate. There was a solid and pseudopapillary pattern around blood vessels. Many cystic areas with fibrin clots inside showed cholesterol deposits and histiocytes inits walls. Immunohistochemical staining for a alpha-1-antitrypsin and pre-keratin were positives. The findings support an origin from exocrine pancreas. Diagnosis was compatible with the solid and papillary tumour of the pancreas. Nine months after surgery patient has no evidence of recurrence. Long survival or even cure can be expected, but the accumulated experience indicate that this neoplasm should be regarded as a low-grade potentially malignant tumour.

### P124

### PROGNOSTIC VALUE OF KLOPPEL TUMOR GRADING VS. TNM IN PANCREATIC CARCINOMA

P.C. GIULIANOTTI, *G. FORNACIARI, *J. BRUNO, °G. ROSSI, A. COSTA, U. BOGGI, A. PIETRABISSA, T. BALESTRACCI, A. GUADAGNI, R. DI STEFANO, F. MOSCA
Istituto Di Chirurgia Generale e Sperimentale, *Istituto di Anatomia Patologica, °Dipartimento di Epidemiologia e Biostatistica C.N.R., Università di Pisa, Italy

An improvement in defining the tumor grading of pancreatic cancer might contribute to surgical decision making and help selecting those cases which might benefit from adjuvant treatments. A relationship between survival and new grading system was proposed by Kloppel et al. in 1985. A retrospective analysis was therefore carried out at our Institution comparing Kloppel grading to standard TNM grading in 57 pts. with histologically proven ductal adenocarcinoma out of 116 who underwent a pancreatic head resection for malignancy between 1982 and 1990. Grading was assessed through examination of the following histologic and cytologic factors: numbers of ducts, mucus production, epitelial shape, nucleous position and shape, presence of anisonucleosis and number of mitosis out of 10HPF at X500. A score from 0 to 2 was given for each factor by two different pathologists and the mean value obtained from at least 5 repeated oservations for each patient was used to allocate the patient to his Kloppel or TNM grading. The numeric data of 57 cases were included in the present study. To correlate the two grading systems (3 grades for Kloppel and 4 grades for TNM), TNM grade 4 were downgraded to grade 3. By this arbitrary modification, the concordance index (K) between the two systems was relevant (K=0.85) (P<0.001[TestT]). There was no correlation neither between grading (TNM or Kloppel) and TNM staging (Exact Test di Fisher) nor between grading and preoperative duration of symptoms (Test di Kruskal-Wallis).

In conclusion our analysis suggests that neither the Kloppel nor the TNM grading are reliable tools to predict survival in patients with pancreatic cancer (Breslow, Mantel-Cox test). In addiction Kloppel grading did not show any significant over the classical TNM grading.

### P125

### SURGICAL STRATEGY COMBINED WITH INTRAOPERATIVE RADIOTHEPY FOR RESECTABLE PANCREATIC CANCER

T. HIRAOKA, K. KANEMITSU, S. TASHIRO, I. KAMIMOTO, S. SUGIHARA, H. TAKAMORI, H. NISHIDA, Y. MIYAUCHI
First Department of Surgery, Kumamoto University Medical School, Kumanoto, Japan

The policy of surgical treatment for pancreatic cancer was studied retrospectively. We have changed historically our policy of treatment four times upto date as follows; standard operation (n=19), standard operation plus intraoperative radiotherapy (IOR) (n=15), extended operation alone (n=9) and extended operation plus IOR (n=19). Five year survival rate of extended operation plus IOR group was 27.8%, but the longest survivor in patients of 3 other operation groups lived for only 3.3 years. Five year survival rate was calculated according to the stage of TNM classification in the 19 with extended operation plus IOR; stage 1 (n=1), 100%, stage 2 (n=3), 0%, stage 3 (n=11), 37.5%, stage 4 (n=4), 0%. Two of three cases with stage 2 tumor died of liver metastases without local recurrence and another one is still alive. On the other hand, survival of 12 cases with small cancer (a diameter of less than 2 cm) in our hospital and affiliated hospitals was studied. Two cases of stage 1 tumor died in the postoperative period. Survival rate of 6 cases of stage 1 tumor was 66.7%. They underwent only standard operation. Of 3 cases of stage 3, only one case with extended operation plus IOR is still alive 4.2 years. One case with stage 4 is still alive 1.3 years after standard operation. We would like to indicate standard operation for patient with stage 1 tumour and extended operation plus IOR for patient with stage 4 tumour, only conservative operation should be indicated.

### P126

### THE FACTORS FOR CURATIVE TREATMENT IN PANCREATIC CANCER

H. IZUMIKA, M.YOSHIDA, K. SHIMADA,K. FURUTA, Y. ITOH, T. SUZUKI, H. YOKOTA, G. KANEDA, S. FUNAMOTO, N. KOBAYASI, K. ASO, H. MIENO, K. SATO, H. OHMIYA, Y. HIKI, A. KAKITA
Department of Surgery, Kitasato University, School of Medicine, Kanagawa, Japan

This is the study to evaluate the factors to affect the prognosis of pancreatic cancer after surgery. Following analysis was done according to "General Rules for Cancer of the Pancreas" in Japan.

Fifty-three resected cases out of 163 pancreatic cancer cases were divided into 4 groups by their tumour size; $T_1$ ($\leq$ 20cm) eight, $T_2$(2.1 - 4.0 cm) 33, $T_3$ (4.1 - 6.0cm) 11 and to $T_4$ (6.1cm $\leq$) one cases. Curative resection was performed in 29 patients (54.7%). Noncurative rates of T1 to T4 were 12.5%, 48.5%, 54.5%, and 100% respecitively. The factors affecting on curative resection were distance from tumour to dissected margin (ew), pancreatic stump (pw) and bile duct stump (bdw). Recurrent rates of $T_1$ to $T_4$ were 75.0%, 63.6%, and 100% respectively.

In conclusion, because of anatomical factors, pancretic cancer has limitation for curative resection and for survival after surgery. To progress the prognosis of pancreatic cancer, we need not only to extend surgical procedures but also to find out more effective adjuvant therapy.

### P127

### RESECTION OF CARCINOMA OF THE BODY AND TAIL OF THE PANCREAS

C D JOHNSON, M SCHWALL, J FLECHTENMACHER, M TREDE
University Surgical Clinic, Mannheim & University Surgical Unit, Southampton

Carcinoma of the body and tail of the pancreas is regarded by many as incurable. Resection is rarely attempted, because the tumour often presents at an advanced stage.

In a consecutive series of 102 cancers of the body and tail of the pancreas, we have performed a resection in 13. All patients have had a minimum 12 months follow up. When possible a left pancreatic resection was performed (n=7); growths extending up to the neck were treated by total pancreatectomy (n=6). Adjacent organs were excised when tumour invasion was present and complete clearance could be achieved (n=3).

There were no postoperative deaths. Median survival is 26 months (range 3-40). Six patients have survived two years (46%) and 6 are still alive 13-36 months after surgery. In contrast 11 patients with locally invasive, non-metastatic tumours all died within 12 months of diagnosis.

Carcinoma of the body and tail of pancreas may be resected with a good chance of survival beyond two years in suitable cases.

### P128

### PANCREATIC STUMP MANAGEMENT AFTER PANCREATODUODENECTOMY

G. NANNI, G. SARRO, G. BRAGHERIO
Division of General Surgery, C. Fornaroli General Hospital Magenta (Milan), Italy

The surgical treatment of resectable cancer of the head of the pancreas is still controversial: pancreatoduodenectomy (Whipple's Operation) or total pancreatectomy. The arguments in favour or against these procedures are the maintenance of endocrine function, the avoidance of the risk of pancreaticojejunostomy, the operative mortality and morbidity, the oncological radicality as well as the longterm survival.

From 1985 to 1990, 34 pancreatoduodenectomies were performed, with the following kinds of management of the pancreatic stump: pancreaticojejunostomy 11 cases (32%), with 2 dehiscences and reoperation; pancreatic stump closure and Neoprene injection in the pancreatic duct 15 cases (44%), with 8 cases of diabetes in the follow-up; pancreatic duct ligation and pancreatic remnant closure iwth fibrin glue protection and perioperative administration of Octreotide 8 cases (24%), with 2 cases of pancreatic fistula which closed spontaneously. The operative mortality was zero.

It appears that the last procedure could reach all the proposed objectives: the maintenance of endocrine function without the risk of pancreaticojejunostomy, and the same oncological radicality and longterm survival. The very low perioperative mortality and morbidity and the good quality of life, justify the further use and evaluation of this technique.

### P129

### PALLIATIVE SURGERY OF THE PANCREATIC CANCER

F. SEFERIADIS, A. MYSTAKIDIS, I. KONSTANTINIDIS, B. BASTAS, K. STAVRIDIS, G. VELMACHOS
"SOTIRIA" General Hospital, Athens, Greece

If the malignant tumor proves to irremovable, the majortiy of surgeons perform one of the many short-circuiting or decompressive operations.

The choise of these palliative procedures continues to be cholecystojejunostomy using the Roux-en-Y loop or the simple jejunal loop technique combined with side-to-side jejunojejunostomy or end-to-side hepaticojejunostomy is carried out to circumvent the possibility of subsequent duodenal obstruction.

Authors report a retrospective study of patients with primary pancreatic tumors who underwent palliative procedures in our department.

They were 28 male and 19 female who ranged in age from 48 to 82 years. Surgical treatment included laparotomy with biopsy alone in 6 patients choledochojejunostomy in 29 patients and cholecystojejunostomy in 12 patients. The mortality rate was 9%. In 36 patients a side-to-side jejunojejunostomy was carried out. A number of surgeons consider that cholecystogastrostomy is the operation of choice for anatomical reasons. This procedure, however, has disadvantages.

The results of the palliative operations are disappointing, as the average mortality rate is about 5 to 10% and within 5-9 months of the operation nearly all such patients are dead. When survival exceeds 1 year the question always arises as to whether or not the primary lesion in the pancreas was in fact cancerous.

### P130

### ULTRASOUND CONTROLLED FINE-NEEDLE BIOPSY, ADDITIONAL METHOD IN CASES OF PANCREAS TUMORS?

J.H.SIMANOWSKI, V.MENDEL, TH.REUHI, H. HEYMANN.
Klinik und Poliklinik fuer Allgemeinchirurgie der Medizinischen Hochschule Hannover, Podbielskistr. 380, D–W 3000 hannover 51, Germany

Sometimes it is impossible to clarify the dignity of tumours in the pancreas head or in the choledochus previous to the operation. Parts of duodenum and pancreas have to be removed, even if the final histology shows a benign tumour. Is it possible do decrease this number using the operative ultrasound examination?

After the usual inspection of the operation field the pancreas head tumour is punctured under ultrasound control. Using a 5 MHz finger- or T-shaped linear array transducer the tumour is punctured in its central and peripheral portions with a 0.6 or 0.7mm wide Chiba-needle under ultrasound view. The collected cells are transferred immediately to a cytology laboratory.

In 13 cases without a preoperative diagnosis of a malign tumour we performed this work- and costintensive operative ultrasound guided puncture. In 3 out of 13 cases we were able to detect malignity of the tumour using the described method. In 8 cases the pancreas was resected and the final histoloth showed in 6 cases a carcinoma, while in 2 cases the tumor was caused by an inflammation. 2 early cases with an operative earned cytology of PAP II were not resected and died 1.5 e.g. 2 years later due to a pancreas carcinoma. In two cases the histology failed.

Cytology gained from operative, ultrasound guided puncture of pancreas tumours does not give a usefull result during the operation. Obviously the cytology fails in cases of highly differentiated pancreas head carcinomas. Until now it seems impractical to detect a tumour inside the inflammative tissue using ultrasound guidance and collecting cells this way from different areas of the tumour. The theory to find the area of high mitoses in a specific area inside the pancreas tumor with ultrasound control lide it is done in cases of liver carcinoma has failed.

### P131

### EVALUATION OF SERUM CA 19-9 IN PANCREATOBILIARY CARCINOMAS

UENO K, NAGAMORI M, INOUE T, TAKEDA T, NAKANO T, MORI K, KAYAHARA M, OHTA T, NAGAKAWA T, MIYAZAKI I.
The Department of Surgrey II, School of Medicine, Kanazawea University, Kanazawa, Japan

Serum concentrations of the CA 19-9 antigens were determined in 149 patients with pancreatobiliary carcinomas. Seventy six patients had pancreatic carcinomas (57 head and 19 body/tail cancer) and seventy three had biliary carcinomas (29 gallbladder, 31 bile duct and 13 ampula cancers. The overall sensitivbity of CA 19-9 was 75.2% (73.7% in pancreatic cancers, 79.3% in gallbladder cancers, 83.7% in bile duct cancers and 53.8% in apulla cancers). In 25 patients with obstructive jaundice underwent the biliary decompressions, serum CA 19-9 in 19 cases (76.0%) decreased apparently after the decompressions. Staging and resectability of these cancers were related to the serum CA 19-9 levels. The normalizations after the resections had the relationships with pathologicial curability. For the early detections of the recurrence in the patients with curative resections, the elevations of serum CA 19-9 levels were more available than the diagnoses by CT and MRI imagings. The conculsion was as follows : the frequent measurements of serum CA 19-9 levels were available to the diagnoses and the treatments in the patients with pancretobiliary carcinomas.

### P132

### INHIBITORY EFFECT OF A CHOLECYSTOKININ ANTAGONIST ON PANCREATIC CARCINOGENESIS AFTER PANCREATOBILIARY DIVERSION

P WATANAPA, B FLAKS, H OZTAS, PH DEPREZ, J CALAM, RCN WILLIAMSON
Department of Surgery and Medicine, Hammersmith Hosp., Royal Postgraduate Medical School, Du Cane Road, London W12 ONN, United Kingdom.

The role of cholecystokinin ( CCK ) has been explored in pancreatic carcinogenesis following pancretobiliary diversion ( PBD )< using the specific CCK receptor antagonist CR-1409. Male Wistar rats ( n=80 ), aged 3 wks and weighing 180-240g, were given weekly i.p. injections of azaserine ( 30mg/kg/wk ) for 3 wks. One week later animals were randomised to receive either PBD or sham PBD and thereafter to receive s.c. injections of either saline or CR-1409 ( 10 mg/kg/day, 5 days a week ). Surviving rats were killed at 6 months, when cardiac blood was taken for CCK assay and the pancreas was excised for wet weight measurement and quantitative estimation of atypical acinar cell foci ( AACF ), the precursor of carcinoma, PBD reduced body weight ( 6-16% less than shams ) but trebled the absolute and relative pancreatic weights ( $P < 0.001$ ). CR-1409 blunted this adaptive response, reducing absolute pancreatic weight by 33% ( $P < 0.005$ .) PBD quadrupled circulating CCK concentrations, regardless of the antagonist treatment. Acidophilic AACF occurred only in rats with PBD. CR-1409 markedly reduced the number of observed acidophilic AACF by 90% ( $P < 0.001$ ). Moreover, CR-1409 reduced the mean focal diameter of each lesion by 18% ( $P < 0.005$ ), the mean focal volume by 58% ($P < 0.05$ ) and the percentage of pancreas occupied by acidophilic foci by 95% ( $P < 0.001$ ). PBD enhanced pancreatic carcinogenesis and CR-1409 inhibits this enhancement.

### P133

### EVALUATION OF THREE NEW TUMOUR MARKERS (CA M43, CA 195 AND TPS) IN PANCREATIC CANCER

A. ZERBI, S PASTORI, D. PAROLINI, G. BANRI, M. CARLUCCI, V. DI CARLO.
Pat. Chirurgica, L. Analisi-IRCCS S, Raffaele-Univ. Milan-Italy

We compared serum levels of three new tumour markers (CA M43, CA 195 and TPS) to levels of CA 19-9 in 71 patients with citologically or histologically proved pancreatic cancer (group 1) and in 21 patients with othe pancreatic diseases (13 acute pancreatits, 7 chronic pancreatitis, 1 islet cells tumour) (group 2). The following cut-offs were used: CA M43 (Genetic System-USA)< 10 U/L; CA 195 (Hybritech Europe-Belgium), 11 U/L; TPS (Beki Diagnostics-Sweden), 40 U/L; CA 19-9 (Sorin Biomedica-Italy), 37 U/L.

| | CA M43 | CA 195 | TPS | CA 19-9 |
|---|---|---|---|---|
| Sensitivity: | 53.5% | 75.4% | 91.5% | 78.9% |
| Specificity: | 96.4% | 60.7% | 21.4% | 72.4% |

The mean positive values (U/L) resulted 266 for CA M43, 453 for CA 195, 684 for TPS, 1958 for 19-9 in group 1; in group 2 they were, respectively, 12, 28, 2112, 112. CA M43 showed the best predictive positive value (97.4%), greated even than CA 19-9 (87.5%), whereas CA 195 presented the best predictive negative value (50%). Serum levels of CA M43 CA 195 and CA 19-9 were related to the stage of the tumour (higher values in more advanced stages), whereas TPS values did not show this correlation.

Our data suggest that CA 19-9 remains the most reliable marker in the management of pancreatic cancer. CA M43, although poor sensitive, is highly specific in detecting pancreatic cancer, and its role deserves further studies.

### P134

### LOCALIZED UNRESECTABLE PANCREATIC CARCINOMA: SURGERY + INTRAOPERATIVE+ EXTERNAL RADIOTHERAPY

E BALEN, JA CIENFUEGOS, F PARDO, JL HERNANDEZ, J FONZALEZ, V VILLA, C BENITO, J TORRAMADE,
Dept. of Surgery, Clinica Universitaria, Univ of Navarra, Pamplona, Spain

Different modes of radiotherapy and biliary bypasses have achieved a significant longer survival and a good local control in localized and unresectable pancreatic carcinoma. We analyze our experience in 30 patients diagnosed of pancreatic carcinoma with vascular invasion, but without distant metastases. They were operated upon to perform tumour biopsy, intraoperative radiotherapy ( 15-20 Gy ) and biliary bypass and/or gastrojejunostomy. Fractionated external beam radiotherapy was delivered up to 40-55 Gy. Symptoms : jaundice (19 cases), abdominal pain (23). Tumour size was greater than 5 cms in 7 patients, and was located in the head (13), body (8) or head-body (9) of the pancreas. Complications : related to radiotherapy, 2 cases of GI bleeding with one death; related to the surgical procedure, 1 abscess, 2 colangitis and 2 obstrictive jaundice among the 10 gallbladder biliary-bypasses. Among the 23 patients with abdominal pain. 7 were not followed-up and 15 had no pain one month after surgery (11 of them did not suffer from pain along their survival time). Medium survival time of the dead patients was 10 months (range 3-23). Currently 4 patients are alive at 3,8,10 and 10 months. A good local control was achived in the majority of cases and tumour progression was mainly to the liver, peritoneum and bone

### P135

### VARIATIONS OF PANCREATIC DUCTS RESEMBLING NEOPLASTIC CHANGES - AN ANATOMO-CLINICAL STUDY

B.STIMEC, M. BULAJIEC, M.MILICEVIC AND M. TERZIC
Institute of Anatomy, Institute of Digestive Diseases, Gyn/Ob Clinic, Faculty of Medicine, University of Belgrade, Belgrade, Yugoslavia

We investigated normal variations of the pancreatic tree which could give the false positive diagnosis of malignancy. The study was carried out on 93 specimens of human pancreas from autopsies, and on 103 endoscopic retrograde pancreatograms (ERP). The anatomic specimens underwent dissection, pancreatography and corrosion casting. Ansa pancreatica, a loop of the main pancreatic duct (MPD), which can be interpreted on CT as a tumefaction, was found in 11.3% of the anatomic specimens and in 16.5% of ERPs. Variations of the MPD course, such as sygmoid and descendent, can look like pathological displacement. They were found in 20% of the cases. Shortening of the MPD, if not belonging to pancreas divisum or the congenital short pancreas, might also be diagnosed as neoplastic changes in the region of pancreatic tail. The minimum length of the MPD was 142 mm on anatomic specimens and 131 mm on ERPs. The region of pancreatic isthmus often presents shortening and rarefication of the side branches, due to the superior mesenteric vessels. This variation, which can give the impression of neoplastic branch amputation, was found in 39% of the anatomic specimens and in 32% of ERPs. The anatomical and clinical method were highly correlative.

### P136

### INTRADUCTAL PAPILLARY TUMOR OF THE PANCREAS: A CLINICAL AND MORPHOLOGICAL STUDY OF TWO CASES

MARRANO D., SANTINI D.,* CAMPIONE O., CASADEI R., ALBERGHINI M.,* PASQUALINI E., PIVA P.
Ist. I^Clinica Chirurgica Generale e Terapia Chirurgica, Universita di Bologna (Italy)
(*) Ist. Anatomia Patologica - Universita di Bologna (Italy)

Intraductal papillary tumors of the pancreas encompass a variety of rare lesions described in literature under different terms. A clinico-pathologic study on 2 cases of intraductal papillary-cystic neoplams of the pancreas, surgically treated at the 1st Surgical Clinic, Bologna University, is reported. Both patients were male with a long history of symptoms resembling those of chronic pancreatitis. The patients underwent total pancreatectomy. Grossly the tumors diffusely involved the main pancreatic ducts in the body-head region, but sections clearly exhibited soft masses cystically distending and partly occluding the ducts. Histologic examination showed a diffuse intraductal papillary proliferation with a cystoarchitectural pattern ranging from mild atypia-dyspasia to severe dysplasia-in situ carcinoma.

Carcinomatous stromal invasion was never observed.

Interestingly the remaining non-neoplastic ducts, showed a wide and diffuse spectrum of different types of hyperplasia and metaplasia. We emphazise that this peculiar kind of intraductal neoplasm constitute a pancreatic tumor entity to distinguish from the common ductal adenocarcinoma and the mucinous cystic tumors.

Moreover the morphologic pattern the lack of stromal infiltration and all cases reported in literature suggest that these tumors may be considered low-grade malignancies.

### P137

### CLINICAL STUDY OF THE PANCREATOSCOPE (BABY SCOPE) FOR PANCREATIC DISEASES

Y. HAYASHIDA, T. SHIMIZU, N. MIZOBUCHI, H. GONDA, T. MAEKAWA, N. SAKAKIBARA,
1st Department of Surgery, Juntendo University, School of Medicine, Tokyo, Japan

The progress and development of fiber optics made possible the production of the pancreatoscope.

The examination was done in 15 pancreatic cases, panceatities and pancreatic cancer, using this pancreatoscope. The useful results of the baby scope are reported in this study. The diameter of the baby scope is only 0.8mm, and inserted into the pancreatic duct through the channel of the mother scope (duodenal fiber scope, JF type fiber scope). Furthermore, the baby scope is inserted through the catheter (diameter is 1.2mm). The baby scope is therefore inserted easily into the pancreatic duct wiht a catheter. Consequently, the observations of the ductual lesion in the pancreas in made possible by the use of the scope. Narrowing ductual mucosa of the pancreas were observed in cancer cases. Also typical findings were observed in mucinproduce pancreas cancer. In normal cases, the smooth-surfacd ductal mucosa was observed.

Using this baby scope and combining the brushing cytology, futher progress in direct vision biopsy, is made in the diagnosis of pancreatic diseases.

Good results are reported in this study of the pancreatoscope.

### P138

### INDICATIONS FOR INTRAOPERATIVE CHOLANGIOGRAPHY. RESULTS IN 'OPEN' AND LAPAROSCOPIC CHOLECYSTECTOMY.

G.ALEXIOU, M.PAPADOPOULOU, B.PAPALOIS, P.ANTONIOU, K.PAPADIMITROPOULOS.
2nd Surgical Dept., Air Force General Hospital, Athens, Greece

From August 1987 until August 1991 we performed 508 'open' cholecystectomies. An intraoperative cholangiography (IC) was performed in 379 cases and in 35 of them (9.23%) the existence of choledocholithiasis was confirmed. In 6 of those cases (17.14%) choledocholithiasis was indicated by a history of jaundice or pancreatitis (HJP), elevated liver function tests (ELFT) and ultrasonography findings (USF), in 2 cases (5.71%) by a HJP and the ELFT in 11 cases (31.42%) by a HJP and the USF, in 5 cases (14.28%) by the ELFT and the USF and in 11 cases (31.42%) only by the USF. We concluded from those results that IC may be performed selectively in laparoscopic cholecystectomy when indications based on a HJP, ELFT or USF are present. From November 1990 until November 1991 we performed 136 laparoscopic cholecystectomies and in only 7 of those cases the procedure was converted into an 'open' laparotomy. In 20 of those cases (15.5% of the laparoscopically performed cholecystectomies) IC was performed and in 3 of them the existence of choledocholithiasis was confirmed. In 2 of the 20 (10%) intraoperative cholangiographies that were performed in laparoscopic cholecystectomies choledocholithiasis was indicated by a HJP, ELFT and USF, in 1 case (5%) by the HJP and the ELFT, in 1 case (5%) by a HJP and the USF, in 7 cases (35%) by the ELFT and the USF and in 9 cases (45%) only by the USF. The 3 cases with choledocholithiasis were treated with ERCP and extraction of the common bile duct stones, while there are no indications of choledocholithiasis up to now for those patients that laparoscopic cholecystectomy was performed without IC. Our experience from the 'open' cholecystectomy and the up to date data from the performance of laparoscopic cholecystectomy suggest that the HJP, the ELFT and the USF are safe indications for the selective performance of IC in both procedures, although the IC is necessary in difficult cases where the biliary anatomy is not clear.

### P139

### PROGNOSIS OF POST-OPERATIVE COMPLICATION IN GERIATRIC PATIENTS WITH CHOLELITHIASIS

A.Ts BUTKEVICH
Department of General Surgery, 2nd Medical Institute, 1 Ostrovityanova Moscow, USSR

With the aid of Mathematic Classification methods on the basis of Patient's study group, table system of prognosis and outcome of operations carried out on Geriatric patients with bile diseases was worked out. This was based on independent estimation of the Patient's general conditions and the gravity forthcoming intervention. Dosage Physical Load (DPL) on Veloergometer, changes in ECG, haemodynamic and functional indices were used during the preoperative period in the estimation of functional reserves in 657 patients. Pneumotachograhic indices pre- and during DPL were studied in order to estimate the functional reserves of external breathing and prognosis of Pulmonary complications.

Conclusions: In many Geriatric patients cardio-pulmonary functional reserves will be found to be deficient under conditions of high demands (operations, narcosis). Index of Myocardial Adaptations (IMA) determined during DPL reflects more the adaptive of reserve state of Myocardium. The correlative dependence of intra- and postoperative cardiovascular complications with the result of IMA. ECG test during DPL helps with a high degree of accuracy to prognosis the development of arrhythmia during operations and in postoperative period. Examinations of the index of external Breathing function under DPL using the programme worked out by us helps to reveal Patients with high probability of Postoperative Pneumonia. Worked out table of prognosis of operative risk helps in the choosing rational surgical tactics thereby lowering postoperative mortality rate.

### P140

### RECURRENT COMMON DUCT BROWN STONE: AN IMPORTANT LONG TERM SIDE-EFFECT OF BOTH SURGICAL AND ENDOSCOPIC SPHINCTEROTOMY.

CETTA F, LOMBARDO F
Institute of Surgical Pathology University of Siena, Italy

The incidence of long term side effects after the damage of the sphincter of Oddi and, in particular, the occurrence of recurrent common duct stones (RCS) of the brown subtype has been evaluated in a series of 63 consecutive patients who underwent surgery for postcholecystectomy stones. Stones were recurrent (i.e. primarily formed postcholecystectomy) in 51 cases and retained ( i.e. missed at previous operation) in 12. Sixty-two per cent of patients with RCS had a previous sphincterotomy (SPHT). In particular, in a subset of 20 patients, who had stone and bile analysis, not only at the second operation, but also at the time of cholecystectomy, 11 cases were found with brown stones formed after SPHT in subjects with previous stones of other type (cholesterol or black pigment stones). In 6 of these patients (with T-tube drainage) stones also formed certainly after the onset of bile infection. In addition, the occurrence of brown RCS was specifically evaluated in a follow-up study of 145 patients with previous surgical SPHT or sphincterooplasty (mean postoperative interval + 6.1 years), 5 with previous ampullectomy and 55 with previous endoscopic

SPHT (and cholecystectomy) (mean interval + 4.3 years). It was found that 11%

of patients with surgical SPHT, 9% those with endoscopic SPHT and 66.6% of patients with ampullectomy had common duct stones of the brown subtype, always associated with bile infection.

It is suggested that, since brown RCS are secondary to bile stasis and infection the duodenum is going to be colonized by bacteria with increasing age, SPHT (and subsequent stricture), facilitating both bile contamination and bacterial overgrowth, could be considered a basic factor for the formation of these stones. Furthermore, since true RCS are mostly of the brown subtype (37 of 51, i.e. 72.5%), it is also_suggested that while SPHT can prevent the occurrence of retained stones, facilitating the flushing of stones that could be missed at the time of cholecystectomy, it undoubtedly increases the total incidence of RCS. In particular, evidence is also given that recurrent common duct brown stones and retained stones (primarily formed in the gallbladder) form according to different pathogenetic mechisms.

### P141

### PREDICTIVE FACTORS FOR CHOLEDOCHOLITHIASIS BEFORE PERFORMING LAPAROSCOPIC CHOLECYSTECTOMY

ARC, ARCIF AND A. FINGERHUT, F. LACAINE, J.M. HAY, TH. MONTARIOL

Intraoperative cholangiograms (IOC) in biliary surgery is associated with well-known performances (2% false negatives and 4% false positives). Of the latter (latent choledocholithiasis), only 10% will become symptomatic, i.e. 0.4% of the 80000 cholecystectomies performed each year in France. Obtaining intraoperative cholangiograms, however, is time consuming and costly with small benefits. Moreover, presently, endoscopic treatment of residual choledocholithiasis avoids reoperation and shortens hospital stay. In 1988, Huguier and Lacaine presented a score based on preoperative sonographic and clinical findings which measures the risk of latent choledocholithiasis (2% when the score is less than 3.5, 47% when the score is greater than 3.5). The French Associations for Surgical Research (promopted by the Ile de France chapter) conducted a prospective study which compared this score as established on sonographic findings, with IOC and intraoperative anatomical findings. Pre and intraoperative scores were quite similar and the conclusions of these quthors were thus validated. As this score has a very high negative predictive valve, the risk of choledocholithiasis when the score value is less than 3.5 is very small. Its positive predictive value, however, is weak: a score of 3.5 or more indicates that a stone may be harboured in the main biliary duct in 25 to 50% of cases. We therefore propse that : 1) IOC may be necessary in patients whose scores are less than 3.5. These patients may be operated on under laparoscopy; 2) when the score is 3.5 or more, IOC should be performed if traditional cholecystectomy is chosen. If laparoscoppic cholecystectomy is performed, there is a 25 cent chance that endoscopic treatment of choledocholithiasis will be needed before (or after) surgery.

### P142

### CHANGES IN BILE COMPONENT - MAJOR FACTOR OF PIGMENT STONE FORMATION

Z.Y. GU, K H. ZHANG
Department of Surgery, Chinese PLA General Hospital, Beijing, China

Abstract: The relationship between pigment stone (PS) formation and changes in bile component has been studied in our laboratory over 15 years.

1. Animal experimental: 125 guinea pigs disvided into two groups fed low protein chow with or without greenery. The incidence of PS formation is 49% in group I, and 94% in group II, respectively. 2. Clinical study: 61 samples of bile were analyed, among them 18 were from PS patients. Results: The major component of the bile after the stone formation in the model found that the value changes of bilirubin and bile acids were similar with those in the PS patients. Moreover, it was also found that the model lower nourish situation, the incidence of pigment stone was higher than those with better nourishment. Stones in model might be disappeared in the short time, if the nourishment of the animal improved with normal chow.

It could be postulated that the medical or dietary interventions to prevent the formation of pigment stone might be enabled.

### P143

### A CLINICAL STUDY ON HEPATOBILIARY DYNAMIC SCINTIGRAPHY IN 252 PATIENTS WITH BILIARY DISEASES

Hu Yize
Department of Surgery, Second Affiliated Hospital, Guangzhou Medical College, P.R. of China

Hepatobiliary dynamic scintigraphy with Tc-99m EHIDA was performed in patients suffering from biliary diseases. Hepatobiliary dynamic morphological imaging with the parameters of time-activity curve of hepatobiliary region of interest were investigated. All patients had ultrasound. Other examinations included PTG in 44, ERCP in 12 and CT in 35. The operation was performed in 221.

Two hundreds and fifty-two patients (M, in 117, F, in 135, Mean age 50 (21-83) years) were studied. Among them, there are bile duct stones in 200 cases (including extrahepatic duct stones in 106, multiple intrahepatic and extreahepatic duct stones in 82, and intrahepatic duct stones in 12), benign bile duct strictures in 8, biliary tumor in 13, acute cholecystitis in 31.

The result of scintigraphy showed accuracy rate to be 86% in bile duct stones, 100% in acute cholecystitis and benign bile duct strictures, and 62% in biliary tumor. Scintigraphy showed accuracy rate of intrahepatic duct stones companying with left liver fibrosis and atrophy in 87% (14/16).

The reliability of scintigraphy related to the serum bilirubin concentration. When the bilirubin level is below 205 umol/L, cholescintigraphy clear with high diagnostic evaluation. When bilirubin level is more than 205 umol/L, the cholescintigraphy is poor and diagnostic evaluation decrease.

Conclusion: Hepatobiliary dynamic scintigraphy is a simple, safe, convenient and noninvasive imaging technique with a combination of functional evaluation and morphological imaging. It is very useful for patients of bile duct stones, benign biliary structrues and acute cholecystitis. Especially when combined with Ultrasound, scintigraphy can provide and accurate rate of 92% in the investigation of obstructive jaundice.

### P144

### GALLSTONE HEPATITIS - AN INDEX OF SYMPTOMATIC BILE DUCT STONES

M. ISOGAI, K. HACHISUKA, A. YAMAGUCHI
Department of Surgery, Ogaki Municipal Hospital
Ogaki, Japan

Gallstone hepatitis, high serum transaminase elevation due to an acute inflammatory hepatic injury in the early stage of gallston impaction, has turned our attention from hepatocellular to extrahepatic biliary disease1).Attacks of bile duct stones (symptomatic bile duct stones, SBDS) may be caused by gallstone impaction. Accordingly, our diagnostic criteria for SBDS are as follows: (1) gallstone colic, (2) elevation of serum transaminase and (3) enlarged biliary tract.

During last 4 years, 132 patients were diagnosed as having SBDS and underwent operation. The mean values of SGOT (N<40 Karmen unit), SGPT (N<35 karmen unit) and bilirubin (N<1.2 mg/dl) were 302 K>U>< 224 K>U> and 2.6 mg/dl, respectively. At operation, 116 patients (87,9%) had bile duct stones, 42 (36.2%) of from had acute suppurative cholangitis. In the remaining 16 patients (12.2%), bile duct stones were not confirmed. They had either gallstone pancreatities or multiple small stones less than 4 mm in diameter in the gallbladder alone.

In summary, gallstone hepatitis can be an index of SBDS.

Reference
1) Isogai M. et al. (1991) Etiology and pathogenesis of marked elevation of serum transaminase in patients with acute gallstone disease. HPB Surgery. 4,95-107.

### P145

### ROUX-EN-Y HEPATICOJEJUNOSTOMY FOR LONG-TERM BILIARY ACCESS AND PERCUTANEOUS TRANSJEJUNAL INTRAHEPATIC STRICTURE DILATATION AND STONE EXTRACTION

JEJ KRIGE, SJ BENINGFIELD*, PC BORNMAN, J TERBLANCHE.
Departments of Surgery and Radiology*, University of Cape Town
and MRC Liver Research Centre, Cape Town, South Africa

Seventeen symptomatic adult patients (12 women; 5 men : mean age 34 years; range 22-64 years) with benign intrahepatic strictures and stones underwent 62 postoperative percutaneous transjejunal biliary balloon dilatations between 1986 and 1991. The intrahepatic strictures were secondary to iatrogenic common hepatic duct injury (5 patients) Caroli's disease (2 patients) choledocal cyst (2 patients) and primary intrahepatic stones (8 patients). All aptients underwent intraoperative stone extraction and stricture dilatation and construction of a Roux-en-Y side to-side hepaticojejunostomy with a 12 cm jejunal access loop marked with silver clips and attached to the anterior abdominal wall. Subdequent postoperative percutaneous transjejunal biliary dilatation of residual or recurrent intrahepatic strictures and stone extraction was performed using a biliary guidewire, co-axial catheter and 7 Fr Gruntzig angioplasty balloon catheter combination.

Five patients with iatrogenic strictures underwent 18 dilatations (mean 3, range 1-6) and are asymptomatic without residual stones. seven patients who had Caroli's, choledocal cysts or primary intrahepatic stones underwent 44 dilatations (mean 2.9, range 1-9). Two patients have residual intrapehatic stones and one has required a left lateral segmentectomy for removal of stones. Minor local or ductal complications related to percutaneous dilatation and stone extraction occurred in three patients wihtout long-term sequelae.

The combined radiological and surgical approach using a Roux-en-Y hepaticojejunstomy and biliary access loop with post-operative percutaneous transjejunal biliary deilatation and stone extraction provides an effective method of treating symptomatic patients with complex residual or recurrent intrahepatic strictures and stones.

### P146

### CHOLEDOCHODUODENOSTOMY. IS IT JUSTIFIED FOR BENIGN DISEASE?

T. LIAKAKOS, J. KOUFOPOULOS, D.KAKOULIDIS, D. MANDREKAS.
2nd Surgical Department of Athens General Hospital.
152 Mesogeion Av., Athens, Greece

To evaluate the long term postoperative results of choledochoduodenostomy (CDD) the records of 210 patients were retrospectively analyzed. All the patients had undergone a side to side CDD for a non malignant disease from Jaunary 1971 to December 1988. 13(6.2%) died in the immediate postoperative period and another 7(3.3%) from malignacy not previousely detected. 35 patients were lost from the follow up and the remaining 155 were followed for 3 to 13 years (mean 7.6 years). The indications for CDD were choledocholithiasis in 97 patients, ampullary stenosis in 32, echinococcosis in 15 and intrahepatic stones in 11. The common bile duct diametre ranged from 18 to 43mm and the length of CDD from 20 to 25mm. Overall morbidity was 24%. The long term follow up revealed 13 (8.4) patients being symptomatic 6 months to 11 years after CDD was performed. 2 of them had 1-3 mild pancreatic attacs and 11 had 1-25 episodes of cholangitis. In 3 cases cholangitis resolved spontaneously, in 2 an ERCP showed impacted stones and endoscopic sphincterotomy was therapeutic. Following a re-operation in the remaining 6 patients, 4 sump-syndromes and 2 fibrous anastomotic strictures were found. Two transduodenal sphincteroplasties and four Roux en-Y covertions were performed. 1 patient died and the rest are symptoms free.

We believe CDD is a quick and safe procedure with few long term complications and it should be always considered as a very good alternative in the management of the complicated problems of the biliary tract.

### P147

### POST-CHOLECYSTECTOMY STONES ASSOCIATED WITH A LONG CYSTIC DUCT REMNANT. A PECULIAR TYPE OF RECURRENT COMMON DUCT STONES

LOMBARDO F, CETTA F
Institute of Surgical Pathology University of Siena, Italy

Data are reported in 11 patients with postcholecystectomy common duct stones associated with a long cystic duct remnant. They were found during a prospective study of gallstone composition and structure related to bile analysis and to the clinical findings of patients in 1000 consecutive surgical cases. Three of these patients had the first operation during the study, so they had stone and bile analysis in both instances. In addition, 3 other patients had saved previous gallbladder stones (GBS), so permitting a comparison between recurrent common duct stones (RCS) and GBS in more than 50% of cases. Stones were cholesterol or mixed faceted, similar to those removed at the first operation in all of the 6 cases in which stones of both operations were available; mixed in 3 cases, and in the 2 last cases stones also contained suture material: they were mixed in one cases and pigment stones in the other. Four other patients with a long cystic remnant and a recent history of pancreatitis underwent operation, because of the suspected presence of microstones both in the common duct and in the cystic remnant, but no stone was found at operation. In addition, 3 patients with a history of repeated pancreatitis and a long cystic remnant were observed (two of whom had the first operation in our institution), without evident stones at ERCP. They were not operated. On the other hand, during a follow-up study of 450 patients, with previous biliary tract surgery (212 had i.v. cholangiography and/or ERCP), 21 patient were found with a long cystic remnant (>10mm) with no stones in the common duct and no history of jaundice or pancreatitis.

The possible role of the cystic remnant in the occurrence of postcholecystectomy symptoms is discussed and possible prevention and therapy is supposed. The possible implications of this pathologic entity in the long term results of laparoscopic cholecystectomy is also suggested.

In addition, the mechanism of formation fo these stones is analyzed and in particular, the possibility is stressed that these stones represent a particular type of postcholecystectomy recurrent stones, i.e. formed after gallbladder removal, but not primarily in the common duct, with a mechanism that is different from recurrent stones primarily formed in the duct.

### P148

### ENDOSCOPIC PAPILLOFISTOLOTOMY FOR PARAPAPILLARY CHOLEDOCHODUODENAL FISTULAS : FOLLOW - UP

M. RYSKA, J. SKALA, F. BELINA
Surgery department - the 3. Faculty of Medicine
Charle University, Prague, Czechoslovakia

We have diagnosed endoscopically 25 cases of parapapillary choledochoduodenal fistulas within period 1986 - 1988. 23 patients underwent cholecystectomy or revision of biliary tract and 2 patients had a gallbladder in situ / men 7, women 18, average 60 years/. In 19 cases with fistulas 1.type have suffered from choledocholithisis 16 patients. One has had sthenosis of papillae of Vater. In 6 cases with fistulas 2. type were found concrements fo common bile duct 3 times. 11 patients were treated by endoscopic papillofistulotomy.

Follow-up was performed in 10 patients during 3-5 years after papillofistulotomy. All of them were without complaints.

Endoscopic papillofistulotomy is recommended as a method of choice of the treatment of fistulas 1.type.

### P149

### THE MANAGEMENT OF COMPLICATED HEPATOLITHIASIS WITH INTRAHEPATIC BILIARY STRICTURE BY THE COMBINATION OF T-TUBE TRACT DILATATION AND ENDOSCOPIC ELECTROHYDRAULIC LITHOTRIPSY

SHYR-MING SHEEN-CHEN, M.D., FONG-FU CHOU, M.D.
Division of General Surgery, Department of Surgery
Chang Gung Memorial Hospital at Kaohsiung,
Chang Gung Medical College, Taiwan, R.O.C.

Hepatolithiasis is prevelant in South-East Asia and presents a difficult problem to manage. Retention of stones behind the intrahepatic biliary stricture is a frequent and troublesome complication. Dilatation of stricture and removal of retained stones are necessary to prevent later sequelae. Therefore, we conducted a prospective trail in 15 patients with retained stones and intrahepatic biliary strictures. Postoperative duct dilatation with PTCS (percutaneous transhepatic cholangioscopy tube, Sumitomo, Japan) tube stenting through matured T-tube tract was performed in these 15 patients. Choledochoscopic electrohydraulic lithotripsy was applied when impacted or/and big stones were encountered. Complete clearance of stones was achieved in 12 patients. The overall successful rate was 80%. Two cases developed fever after ductal dilatation, and recovered by conservative treatment. These 12 patients are well without recurrent symptom with a mean follow-up of 18 months. Postoperative T-tube tract dilatation, selectively combined with endoscopic electrohydraulic lithotripsy, is an effective and safe mehtod for complicated hepatolithiasis with biliary strictures.

### P150

### ULTRASONIC EVALUATION OF COMMON BILE DUCT (CBD) PATHOLOGY: PROSPECTIVE COMPARISON WITH INTRAOPERATIVE CHOLANGIOGRAPHY, MANOMETRY AND ERCP

P. SUNGLER, M. HEINERMAN, O. BOECKL
Ist Surg. Dept. and L.-Boltzmann-Ist. for Exp. and Gastroenterol. Surg., LKA Salzburg, Austria

Ultrasonography is the modality of choice for the diagnosis of cholelithiasis, but its role in the diagnosis of CBD pathologies is still under discussion. In the age of minimal access surgery - i.e. laparoscopic cholecystectomy - CBD stones, detected during surgery are a theapeutical dilemma, either leading to laparotomy and CBD intervention or hopefully successful postoperative ERCP. To avoid these problems, between January 1987 and August 1991 we performed a prspective study on 612 patients with symptomatic gallstone disease. All patients underwent biliary ultrasound preoperatively. Patients with a CBD diameter of less than 6 mm had a simple cholecystectomy with intraoperative cholangiography and manometry. Out of this group (n = 394), 10 patients had CBD stones, missed by preoperative ultrasound. Patients with a CBD diameter over 6 mm had preoperative ERCP (n = 199). 90% of these patients showed pathologies of the common bile duct such as CBD stones or papillary stenoses.

All these patients also underwent cholecystectomy. Sensitivity of preoperative ultrasound with regard to CBD pathologies amounted to 95.22% and specificity to 95.28%, residual stones were only found in 0.98%. In conculsion of our data, biliary ultrasound is not accurate in the diagnosis/exclusion of CBD stones, but is a very sensitive and specific tool in assessment of CBD pathology, helping to avoid management problems in laparoscopic cholecystectomy.

### P151

### CHOLEDOCHOLITHIASIS IN THE HIGH RISK PATIENT. SURGICAL TREATMENT OR ENDOSCOPIC SPHYNCTEROTOMY LEAVING THE GALLBLADDER IN SITU

TRIAS, M, TARGARONA, EM, PROS, I, MARTINEZ, J, GUEVARA, C, ROS, E, BORDAS, J, TERES, J.
Dept. of Surgery, Gastroenterology and Endoscopy. Hosp. Clinic, Univ. of Barcelona, Barcelona, Spain.

Morbidity and mortality after cholecystectomy and bile duct exploration increased with age and associated disease. Endoscopic sphyncterotomy leaving the gallbladder 'in situ' (ES-GiS) has emerged as an useful alternative. Nevertheless, studies comparing both therapeutic options are lacking. The AIM of this study has been to compare the results of endoscopic or surgical approach of bile duct lithiasis in the elderly or high risk patient. MATERIAL AND METHODS Between january 1988 to december 1990 we recorded 152 patients diagnosed of choledocholithiasis. 107 of them were surgically treated (Group I) and 45 received endoscopic treatment leaving the gallbladder "in situ" (Group II). Age over 70 or a systemic chronic illnes (SCI) were considered as risk factors.

RESULTS

| | Group I (SURGERY) | | | Group II(ENDPSCOPY) | | | |
|---|---|---|---|---|---|---|---|
| | >70y. | SCI | >70y+SCI | >70y. | SCI | >70y+SCI | P |
| N | 55 | 59 | 34 | 36 | 30 | 24 | NS |
| Morbidity | 22% | 32% | 27% | 17% | 27% | 17% | NS |
| Mortality | 9% | 6,8% | 11,8% | 2,7% | 3% | 4,2% | NS |
| Eficacy (bile duct clearance rate) | 100/107(93.5%) | | | 40/45(89%) | | | NS |
| Biliary symptoms (follow up 14±6m) | 4% | | | 13% | | | NS |

There were no statistical differences between the groups, but a lower morbi-mortality was observed in the endoscopic group. CONCLUSION. ES leaving the gallbladder in situ is an useful alternative to the classic surgical approach of choledocholithiasis. Prospective studies are needed to know the specific clinical features determining the indication of every one of these therapeutic options.

Study supported by grant 91/416 of FIS

### P152

### WHAT HAPPENS WITH THE GALLBLADDER AFTER ENDOSCOPIC SPHYNCTEROTOMY?

TARGARONA EM, PROS I, MARTINEZ J, GUEVARA C, BIANCHI L, BASSA P, ROS E, BORDAS J, TERES J, TRIAS M.
Dept, of Surgery, Gastroenterology, Endoscopy, Radiology and Nuclear Medicine. Hosp. Clinic. Univ. of Barcelona, Spain

Endoscopic sphyncterotomy leaving the gallbladder "in situ" (ES-ViS) has emerged as an alternative to surgery in the treatment of choledocholithiasis in elderly or high risk patients. Nevertheless, little attention has been paid to gallbladder function after ES, and several studies report contradictory results[1,2]. This aspect is of interest because it may justify the use of pharmacological dissolutive therapy for the residual gallbladder stones. AIM: to investigate the gallbladder function after endoscopic sphyncterotomy, studied by biliary scintigraphy and ultrasonography. MATERIAL AND METHOD: 11 patients (6 f, 5 m, Mean age 72±10) were treated by ES-ViS between January 1989 - October 1990 (follow-up: 16±10 m). ES was performed to treat gallstone pancreatitis (4 cases), acute cholangitis (6 cases) and jaundice (2 cases). ERCP demonstrated a patent cystic duct in all patients. Previously to ERCP, stones in the gallbladder were observed by ultrasonography in 6 cases (4 stones, 2 sludge). A biliary scintigraphy (HIDA) with late scan (240'), hepato-biliary ultrasonography and biochemical hepatic function tests (Bi, FA,GGt) were performed. 3 patients received urodeoxycholic acid for 24 months after ERCP. RESULTS: Biochemical parameters of biliary function were in normal range in all cases. Gallbladder filling with HIDA was observed in 7 cases (64%), with a delayed presence of the isotope in 6 cases (>240'). Ultrasonography observed that gallbladder stones disappeared after ERCP in 2 cases. UDCA failed to dissolve gallbladder stones in the 3 cases.

CONCLUSION: 1.ES do not abolish gallbladder function, and isotope filling could be observed in most cases. 2. In spite of the preservation of the gallbladder function, the efficacy of pharmacological disolutive therapy of stones after ES and ViS must be tested by prospective studies.

1. Desa et al, Br Med J. 300:1111, 1990

2. Holbrok et Al, Arch Surg, 125:738, 1991. Study supported by grant 91/416 of FIS

### P153

### A STUDY ON THE PROTEINS IN GALLSTONE

LIANG-FANG XIA, GUO-FU ZHOU, LIN-DE ZHOU, YONG-FANG ZHAO
Dept. of Surgery, Affiliated Hospital of Guiyang Medical College, Guizhou Province Institute of Geochemistry, Academia Sinica, P.R. China

The proteins in gallstone were studied by means of SDS-polyacylamide gel electrophoresis after the gallstone solutions were dialysed and concentrated, and several stainning methods were used to determine its composition. The results of the experiment show that there are generally five protein bands both for cholesterol and pigmental stones in the electrophoregram stained by Coomasie Brilliant Blue, and it is also basically identical that about a dozen of fine bands were possessed by those 2 different types of gallstone when more sensitive staining method of Coomasie Brilliant Blue-Silver was adopted. Moreover, glycoprotein and polysaccharide were found by the use of PAS procedure. The result of thin layer scanning shows that glycoprotein makes up some 50% of the total amount of the proteins. Besides, it is proved that protein-pigment complex does exists in gallstone. The experimental results suggest that it might have similar external conditions when pigment in those 2 types of gallstone is sedimented, and other proteins except glycoprotein may also play a role in the formation of gallstone.

### P154

### LATE RESULTS OF A NEW STYLE OF APJICD - ANALYSIS OF 150 CASES

CHAO-ZHEN WANG
Tianjin 2nd Central Hospital, Tiangin 300120, P.R.China

150 cases of hepatolith were treated by APJICD and followed for 7 years. Improving old style of the operation: the length of artificial papilla was added to 2 cm from 0.5cm, the length of JI was shortened to 15cm from 20-25cm. 150 cases were followed for 7 years, infection did not occur in any cases. In 12 cases the length of the artificial papilla were proved by gastroduodenosy in a range of 0.8-1.5cm. The papilla was full and wasn't atrophy.

**P155**

### LAPAROSCOPIC CHOLECYSTECTOMY: REPORT OF 260 CASES.

G. ALEXIOU, S. RIZOS, P. ANTONIOU, B. PAPALOIS, A. KOURIS
2nd Surgical Dpt., Air Force General Hospital and Department of Surgery, St. Anargiri Hospital, Athens, Greece

The new method of laparoscopic cholecystectomy is rapidly gaining acceptance by the Greek surgical community. We present the experience of 260 cases in which laparoscopic cholecystectomy was performed in two Greek surgical centers from November 1990 until November 1991. There were 93 men and 167 women with a mean age of 55 and 53 years respectively. The laparoscopic procedure was successfully completed in 249 cases while in 11 patients the procedure was converted to an open laparotomy due to unforeseen technical difficulties. Operative time decreased from an average of 130+/-51 min for the first 20 cases, to 80+/-30 min for the last 20 cases. patients were kept overnight, given clear liquids the evening of surgery, a regular diet the next morning and prescribed oral analgesics (35%). All the patients received an IV dose of amoxycillin/clavulanic acid intraoperatively and three doses after that. 20 patients underwent the procedure as outpatients. Cystic duct cholangiography was selectively performed in 53 cases based on a history of jaundice of pancreatitis, elevated liver function tests and ultrasonography findings. ERCP with extraction of the common bile duct stones was performed either before laparoscopic surgery (1 patient), or in the immediate postoperative period (5 patients). Mean hospital stay was 2.4+/-1.3 days. There were no deaths. 2 patients required delayed exploration early in our experience: 1 for a bile leak and 1 for subhepatic abscess. 1 patient suffered a trocar injury resulting in a subcutaneous haematoma. A minor infection was present in 3 cases at the area in which one of the trocars was inserted. Subcutaneous emphysema was noticed in 3 patients. Shoulder pain was encountered in 11 patients. We conclude that laparoscopic cholecystectomy is a minimal access safe surgical procedure which results in significantly decrease length of postoperative hospitalization, requirement for pain medication, faster return to work and excellent cosmetic results

**P156**

### LAPAROSCOPIC CHOLECYSTECTOMY IN THE NETHERLANDS

G.D. ALGIE, P.M.N.Y.H. GO, D.J. GOUMA
University Hospital Maastricht, The Netherlands

Laparoscopic cholecystectomy has been introduced during the past year all over The Netherlands. The aim of this study was to evaluate the early results of this new method introduced by 58 surgeons after attending a practical course.

Data of 546 patients with a mean age of 50 years (range 20-80) were collected. The indication was symptomatic gallstone disease without evidence of common bile duct stones, or cholecystitis. 70% of the surgeons performed less than 10 laparoscopic cholecystectomies. The average operation time was 95 minutes. In 8.2% of the patients the laparoscopic procedure was converted to laparotomy. Adhesions, cholecystitis or difficulty in recognition of the anatomy was responsible for the conversion in 31 of the 45 patients. In the remaining 14 patients bleeding or bile leakage during the procedure compel the surgeon to perform a laparotomy. One patient died because of bleeding from the cystic artery. Eleven patients underwent a laparotomy postoperatively because of bleeding (3) and bile leakage. Minor complications were found in 30 patients.

Laparoscopic cholecystectomy diffuses rapidly in The Netherlands. The early results are encouraging, although the experience is limited. Further registration is necessary to be able to compare the results of the laparoscopic cholecystectomy more critical to those of conventional cholecystectomy.

**P157**

### LAPAROSCOPIC CHOLECYSTECTOMY IN A DISTRICT HOSPITAL - THE FIRST FIFTY CASES

J BROCKWELL, Y P TAI, J H B KONG, L I WIJESURIYA, M K W LI
Department of Surgery, Alice Ho Miu Ling Nethersole Hospital, Bonham Road, Hong Kong

Alice Ho Miu Ling Nethersole Hospital is a 365 bed district hospital serving Hong Kong Island.

From May to November 1991 fifty one patients were planned for laparoscopic cholecystectomy, all had ultrasound examination and thirty six had pre-operative ERCP. Average age was 54 years (range24-81), and weight 60 kg (35-93), there were 23 men and 28 women.

Two ERCPs revealed CBD stones which were dealt with by papillotomy and stone extraction and followed by uneventful laparoscopic cholecystectomy. In one case laparoscopy revealed unsuspected metastatic liver tumour, and the cholecystectomy was not performed. Two cases were converted to open operation, one due to inability to identify the anatomy because of chronic cholecystitis and one due to Mirizzi's syndrome. Three cases had concomitant laparoscopic lysis of adhesions to the anterior abdominal wall.

Mean operative time was 90 minutes (range 35-280), and postoperative stay 2.8 days (1-9). There were no complications in the laparoscopic cholecystectomy group.

Clinically there were 21 cases of gallstone disease, 11 of chronic cholecystitis, 12 had adhesions from previous cholecystitis, 2 were acute on chronic cholecystitis, there was one empyema and two mucocoeles, but histology revealed one occult carcinoma of the gallbladder in one the chronic cholecystitis cases.

We have shown that in a district hospital this is a safe and cost effective procedure with dramatically less morbidity than open cholecystectomy, and is an alternative which is much appreciated by our patients.

**P158**

### NON-INVASIVE TREATMENTS FOR CHOLECYSTO-CHOLEDOCHOLTHIASIS

H. HASEGAWA, T. TAKADA, H. YASUDA, K. UCHIYAMA, Y. MISU, J. SHIKATA,
First Department of Surgery, Teikyo University, School of Medicine, Tokyo, Japan

More than 10 percent of the patients of cholecystolithiasis seem to have been complicated with choledocholithiasis. Laparoscopic cholecystectomy (LC) has become popular because of its non-invasive surgical procedure. However, laparoscopic treatment for choledocholithisasis has been insufficient and unsatisfactory. In this way we could not make advantages of LC as a non invasion treatment. In our department of surgery, we planned two stages of treatments for cholecysto-choledocholithiasis. For such cases, choledocholithiasis would be treated with endoscopic shincterotomy (EST). Later, when the result of the previous endoscopic treatment of choledocholithiasis is confirmed, LC would be performed for cholecystolithiasis. Preoperative insertion of endoscopic naso-biliary drainage (ENBD) tube would be helpful as a prevention of intraoperative bile duct damages during operation.

### P159

### PREOPERATIVE EVALUATION BY DIAGNOSTIC MODALITIES FOR THE CASES OF LAPAROSCOPIC CHOLECYSTECTOMY

K. HAYASHI, K. YANAGISAWA, M. NUMATA, Y. MUNAKATA, F. SHIMIZU, S. HASHIMOTO, A. KAI
Department of Surgery, Nippon Telegraph and Telephone (NTT) Corporation Nagano Hospital, Nagano, 380 Japan

An aim of this study is to verify the presence of adhesion in the upper abdominal cavity preoperatively for safer laparoscopic cholecystectomy (LC). We diagnosed the presence of adhesion by ultrasonography (US) and drip-infusion cholecystography (DIC). Respiratory movement was examined at the point of hepatic wedge, surrounding gallbladder and umbilicus by sagittal scan of US. Not only respiratory movement but also postural movement were evaluated by DIC. Thirty-three patients with gallstone and 4 patients of polyp of gallbladder underwent LC during 7 months. 14 patients were male, 23 were female, mean age was 48 years and 16 cases had abdominal operations previously. From the point of the respiratory movement by US, presence of adhesion between abdominal wall and intraabdominal viscera were diagnosed exactly in all cases. The presence of adhesion between gallbladder and surrounding viscera were diagnosed in 73%, and that of the adhesion with previous operation scar were diagnosed in 80%. More than 3cm postural and respiratory movement by DIC had no adhesion at the upper abdominal cavity. Preoperative precise diagnosis by dynamic US and DIC were effective for safer laparoscopic cholecystectomy.

### P160

### LAPAROSCOPIC CHOLECYSTECTOMY: THE FIRST 200 PATIENTS IN CATHAY GENERAL HOSPITAL

CHING-SHUI HUANG
Department of General Surgery, Cathay General Hospital, Taipie Taiwan

Laparoscopic cholecystectomy (LC) has rapidly gained world-wide acceptance in the last two years. The applicability, safety and efficacy of this new procedure for the treatment of cholelithias is in Taiwan however, needs evaluation. We performed L C in 200 out of 323 cases of cholelithias is treated surgically at Cathay General Hospital from 28 Dec., 1990 to 28 Oct., 1991. We found that the applicability rate was 62% of biliary stones, 78% of gall stones. The reasons for not selecting L C were: CBD stone in 44 (4 CBD stone patients underwent LC after EPT), IHS in 17, concomitant intra-abdominal malignancy in 5, acute gangranous cholecystitis, pancreatic abscess and multiple upper abdominal incision etc in 57. In the L C group, there were 160 patients with symptomatic chronic calculus cholecystitis, 31 patients with acute calculus cholecystitis and 19 patients with gall bladder polyps, the age of the patients ranged fron 27 to 83 (average 51). There were 78 males and 122 females. All of the patients had a detailed preoperative workup including complete LFT test and sonographic exam of the hepatobiliary system, additional pre-operative ERCP were done in 31 patients and intra-operative cholangiograms were done selectively in another 12 patients to confirm the absence of CBD stone or to delineate anatomy. 198 of the 200 patients completed the LC procedures with only 2 converted to open cholecystectomy (acute cholecystitis with severe adhesion). The average operation time was 60 minutes, (22 to 195 minutes). Drain tubes were used in 62 of the 200 pts, Nd-YAG laser was used in 21 pts. The post-operative hospital stay averaged 3 days, (18 hours to 14 days). The significant complications (3.5%) were 1 case of delayed presentation of right subphrenic accumulation of sterile bile stained fluid (sono-guided drainage), 2 cases of spontaneous passage of small retained CBD stones, 1 case of neglected CBD stone treated post-operatively with EPT, 1 case of CBD injury repaired by Roux-en-Y hepaticojejunostomy, 1 case of bile leakage from cystic duct stump repaired through laparotomy, one case of multiple liver abscess treated with antibiotics. Complications of transient shoulder pain (16%) and mild nausea or diarrhea (10%), wound infection(1.5%), subcutaneous emphysema (1.5%) were nonsignificant. All of the patients returned to normal daily activity within 1 week except for the 7 with significant complications. The total cost of hospitalization was about NT20,000 dollars less than for an open cholecystectomy. From the results of our initial 200 cases of LC in which the conversion rate was 1% and mortality rate were zero, the significant complication rate was on 3.5%, and the applicability rate was 62% (78% for gall bladder stones), we conclude that the laparoscopic cholecystectomy is a safe, efficient, and useful procedure for cholelithiasis patients in Taiwan.

### P161

### BILIARY SLUDGE: COMPOSITION, PATHOGENESIS AND CLINICAL ASPECTS

P. INNOCENTI, R. COSTANTINI, A. D'AULERIO, A.M. NAPOLITANO
Istituto di Patologia Chirurgica Universita di Chieti, Chieti, Italy

Gallbladder sludge consists of a suspension of calcium bilirubinate granules, cholesterol monohydrate crystals and other calcium salts in bile, which is viscous because of its high mucus content. The cholesterol and bilirubin components of sludge are derived from bile itself, but the mucus component must have originated in gallbladder. In some instances sludge appears to be a transient abnormality, but in others it is associated with gallbladder disease.

Some patients with sludge are asymptomatic but most of them have a clinical problem. In our study, we correlated the finding of gallbladder sludge by ultrasonography, CT-Scan and miroscopic examination of duodenal bile, with the patient's clinical symptoms. Of the 8 patients with sludge studied in the past three years, 4 had biliary colics, 2 had acalcolous cholecystitis and 2 had pancreatitis. Therefore, gallbladder sludge in symptomatic patients should be treated as a symptomatic gallstone disease.

### P162

### THE LAPARASCOPIC CHOLECYSTECTOMY - A STANDARD THERAPY OF FUTURE

JOSTARNDT, L., G. BONHAG.
Johannes-Hospital Dept. Surgery, JohannesstraBe 7-13, 4600 Dortmund 1
W.-Germany

The conventional cholecystectomy is now the standard therapy of symptomatic cholelithiasis with a morbidity rate of 6% and a lethality rate below 1%. These results the laparascopic cholecystectomy has to exceed, if it will be the standard procedure of future.

The risk and complications of endoscopic cholecystectomy for untrained surgeons were analysed in 300 consecutively operated patients from 01.12.90 until 30.11.91.

We found after a training phase of 50 operations a morbidity rate of 5%, and intraoperatively laparatomy of 1,5%. None of our patients died.

The laparascopic cholecystectomy has advantages because of less pain, quicker mobility and reconvalescence and will therefore be the standard therapy of choice with minimal morbidity.

**P163**

### EXPERIENCE WITH LAPAROSCOPIC SURGERY

N. KANO,T. YAMAKAWA, Y. ISHIKAWA, S. SAKAI, H. HONDA, A. TACHIBANA, A. OHMURA*, M. SHA*, T. NAKAMURA*
Department of Surgery and Department of Anesthesiology*, Teikyo University Hospital at Mizonokuchi, Kawasaki, Japan

Since the first successful laparoscopic cholecystectomy (lapcholy) has been carried out on May, 1990, 200 patients were considered to be candidates for lapcholy and 7 cases were converted to open chlecystectomy up to the end of September.

Prior to deciding an indication of this procedure, ERCP or DIC (if ERCP failed) was routinely carried out beside a standard preoperative examination. The patients with acute gangrenous cholecystities, jaundice, pancreatities, gallbladder cancer, portal hypertension and pregnancy were exluded from the candidates for lapcholy.

On 4 patients with common bile duct (CBD) stone, lapcholy was performed after removal of CBD stones by Endoscopic Sphincterotomy (EST). In one patient in whom EST had failed in removing the CBD stones, lapcholy with laparoscopic choledochotomy and T tube drainage was successfuly carried out.

The causes of conversion to open chlecystectomy in 7 patients were attributable to uncontrollable bleeding from the cystic artery in one case, venous bleeding due to portal hypertension in one, extensive adhesion of the omentum and the duodenum to the gallbladder in one and extensive adhesion of the omentum around the gallbladder in 4 cases.

On the other hand, laparoscopic appendectomy and emergency laparoscopy have been performed on 5 patients each.

Indication of laparoscopic biliary surgery is being broadened and its technology can be applicable to other abdominal surgery. However we surgeons should note that the skill of the conventional surgical procedure will become more important to prevent major complications if laparoscopic surgery will become more routine.

**P164**

### EFFECTIVENESS OF INTRAOPERATIVE EXAMINATION DURING LAPAROSCOPIC CHOLECYSTECTOMY

S. KOBAYASHI, S. SHIOMI, K. SAKAMOTO, T. MAEKAWA, N. SAKAKIBARA, N. ARAMAKI*, First Department of Surgery and Department of Gastroenterology*, Juntendo University, 2-1-1 Hongo bunkyouku Tokyo 113, Japan

The usefulness of intraoperative ultrasonography (US) and cholangiography (CG) during laparoscopic cholecystectomy (LC) are evaluated in this study.

Thirty patients, to whom successful intraoperative examinations (US and/or CG) of biliary tract could be performed, are mentioned.

The conventional method of intraoperative CG was done. The endorectal transducer probe was used for intraoperative US. The entire length of the CBDs were visible patients by using US. None of the patients revealed gall stones in the CBD. Biliary injury which could have diagnosed precisely by intraoperative CG was not detected using US in one patient who had suffered it. It could have been precisely diagnosed by intraoperative CG.

In one patient, it was made sure that CBD was absent of pathology by US'since it was impossible to insert a catheter into a cystic duct to make CG due to cystic duct occlusion. No complication related to the use of both methods was encountered.

The outcomes of this study leads to the conclusions that most of the cases with enough preoperative information about CBD do not need the intraoperative examination of CBD. However, in some cases, small stones should be examined with US and in some cases where bile duct can not be recognised should be examined with CG.

So these two methods now should be used alternatively. The improvement of ultrasonography will be an effective sustitute for cholangiography as a screening method for CBD pathlogy during laparoscopic cholecystectomy.

**P165**

### LAPAROSCOPIC CHOLECYSTECTOMY: ITALIAN EXPERIENCE

R. LA ROCCA, A CAPOMAGI, G EMMA, A. DE SANCTIS, E. CROCE*, L. NOVELLINO*
Dept. of Surgery INRCA Ancona, *Dept of Surgery FBF, Milan Italy

Laparoscopic Cholecystectomy (LC) is rapidly evolving as a therapeutic modality for the treatment of gallstone disease. The Authors report the Italian experience in LC. The retrospective survey concerns two Italian centers; Milan (M) and Ancona (A), which performed 500 LC (430 M, 70 A). Conversion to open cholecystectomy was necessary in 1.4% patients (4 M, 3A), because of anatomical difficulty (2M, 3 A) and presence of neoplasm (2 M). There were no deaths reported and the cumulative postoperative complications rate was of 2.2% (11 cases) with 2 major complications requiring re-laparotomy. Most of these complications occurred in cases of sclero-atrophic cholecystitis. 30 cases of choledoco-cholecystic lithiasis were performed by endoscopic sphincterotomy followed by LC and other 2 cases were treated by laparoscopic choledocotomy. In the group of major complications the incidence of common bile duct damage was 0.4%. Intraoperative cholangiography was performed only by M group. Mean operating time improved significantly with experience (M from 87 to 54 minutes, A from 109 to 76). The opening alvus occurred on averace after 20 hours. Total mean postoperative stay was 3.5 days. There were no need of postoperative narcotics in more than 85% of patients. In our experience, LC can be performed safely on most patients who are candidates for open cholecystectomy.

**P166**

### THORACIC EPIDURAL ANESTHESIA FOR LAPAROSCOPIC CHOLECYSTECTOMY

C.J.M. LANGENBERG, F. HUYGEN, P.M.N.Y.H. GO, D.J. GOUMA
University Hospital Maastricht, The Netherlands

The aim of the study is to evaluate the feasability to perform laparoscopic cholecystectomy under locoregional anesthesia.

Twenty five consecutive patients who had given informed consent received epidural anesthesia, mepivacaine 2% + adrenaline (1/200.000) and sufentanil forte 1-1.5 mcg/kg through a catheter which was inserted 3 cm cephalad at Th6-Th7. The level of the block was controlled with pinprick and temperature sensation. Pain sensation was scored with a visual analog scale (VAS). The incidence of side effects such as priritus, nausea, respiratory depression and hypotension were measured.

After fifteen minutes block levels varied rostral from C8-Th4 and caudal from Th8-L2. In twenty patients the analgesia was adequate (VAS=0). Five patients needed additional general anesthesia: (n=2) with a VAS score of 8 and 9, and inadequate muscle relaxation for surgical acces (n=3)

Three out of twenty patients had peroperative pruritis, one complained of nausea, and two had a slight respiratory depression. In five patients blood pressure decreased more than 20%. All side effects were easily treated, and surgery was uneventfull in all patients. Thoracic epidural anesthesia for laparoscopic cholecystectomy seems to be a safe and adequate technique.

**P167**

### ENDOSCOPIC MANAGEMENT OF RETAINED AND RECURRENT BILIARY STONES: THE RESULTS

LESZCZYSZYN J., DUREK W.,LAWINSKI M.
Endoscopy Division, PKP Hospital, Wroclaw-Poland

From 1987 to 1991 -631 ERCP have been done. We received the data of 121 therapeutic ERCP procedures which were performed on patients with recurrent or retained CBD stones during period January 1990 - June 1991.

Successful stone removal after sphincteromomy alone was achieved in 82 cases (68%); mechanical lithotripsy was necessary in 27 cases (23%); transdudenal cholangioscopy and electrohydraulic lithotripsy was performed in 7 patients (5%); temporary biliary stent was inserted in 3 patients (3%); failure of any endocopic method in 2 patients (2%). This two patients were referred to surgery after naso-bilaiary tubes had been placed on.

Complications were found in four cases (3,2%). The bleeding after sphincterotomy in two patients and after electohydraulic lithotripsy in one patient; they all recovered by conservative treatment.

One patient, after stenting, had transistent fever and chills.

**P168**

### SAFE LAPAROSCOPIC CHOLECYSTECTOMY IN A COMMUNITY SETTING, N=762

MATT B. MARTIN, M.D. Biliary Surgeons of Greensboro
311 West Wendover Avenue, Greensboro, North Carolina, 27408 U.S.A.

Laparoscopic cholecystectomy (LC) can be introduced into a community with morbidity and mortality rates equal to that of open cholecystectomy. The entire biliary surgical community (17) of Greensboro (pop. 196,000) learned the technique of LC on animal models prior to offering this innovation to the community. over the ensuing 12 months, they served as surgeons or assistant surgeons to each other on 762 LC with morbidity and mortality rates comparable to open cholecystectomy.

This retrospective study examined the first one year experience beginning 8/13/90. This work represents all of the LC performed in Greensboro and all of the surgeons participated in this review. All of the surgeries were done with an electrocautery and utilized a zero degree forward viewing scope. Cases were performed at two hospitals with surgeons as both operator and assistant and no effort was made to exclude high risk or elderly patients from this procedure.

Patients averaged 50 years of age and ranged from 14-96 yrs. Static cholangiograms were performed in 27% of patients. Conversion to open cholecystectomy was seen in 4.8%. There were two cardiac deaths(0.26%) and significant complications were seen in 3.4%. Seven patients required reoperations. There were no major common bile duct injuries.

This retrospective review indicates that this new procedure can be introduced into a community setting by novice laparoscopic surgeons acting both as operators and assistant with a morbidity and mortality rate comparable to that reported for open cholecystectomy.

**P169**

### GALL STONE ILEUS

M. HAMANO, K. OGINO, A. YAMADA, K. WAKITA, T. ICHIHARA, H. FUJIWARA, T. ASATSU, T. ISHIDA, K. TSUJIMOTO, T. MITSUNO
Yodogawa Christian Hospital, Osaka, Japan

Gall stone ileus, due to the impaction of one or more gall stones within the lumen of the bowel, is a rare complication of cholelithiasis and an equally rare cause of mechanical intestinal obstruction.

Between 1987 and 1991 we accepted 4 cases of gall stone ileus. All were women and the mean age was 68 years. three of four cases could be clinically diagnosed preoperatively as having a biliary enteric fistula, X-ray films showed air in the bile ducts (pneumobilia) and niveau, and stone changing postion. Stone size averaged 4.0cm in diameter (range: 3.0-5.0cm). Obstructing stones were located in the terminal ileum in 3 patients, in the proximal ileum or jejunum in 1 patient. In one instance, more than one stone (two) was involved. Cholecystoduodenal fistula was the most frequent fistula type (75%) followed by cholecystoduodencolonic type (25%). Surgical procedure consisted in each case by enterolithotomy alone, and a one-stage prodcedure consisting of the removal of the impacted stones, fistula repair and cholecystectomy were performed in only one patient.

In conclusion, when conditions are poor and local findings are not clear, a two-stage procedure should be planned consisting first of enterolithotomy to eliminate ileus. This should be followed by elective biliary surgery.

**P170**

### GALLSTONE ILEUS

A. MYSTAKIDIS, F. SEFERIADIS, G. SVORONOS, J. KONSTADINIDIS, L. CHRISTODOULOU, G. VELMACHOS
"SOTIRIA" General Hospital, Athens, Greece

Purpose of this presentation is to present four additional cases and to evaluate the diagnostic and therapeutic procedures by reviewing the literature. Our patients, 3 women and a man, aged from 69 to 82 years, were admitted with symptoms and signs of small bowel obstruction. The correct diagnosis was established preoperatively in two of our cases. The gallstone was impacted in the duodenum in one case, in the jejunum in a second and in the terminal ileum in the remaining two. One cholecysto-duodenal fistula was repaired, with the addition of a gastrojejunostomy, primarily, while enterolithotomy alone was performed in three cases. Leakage from the site of duodenal repair was a major postoperative complication, which was treated successfully by conservative measures, while severe respiratory sepsis caused the death in one of our cases. Twp patients had uneventful recovery. We conclude that gallstone ileus is a rare and frequently misdiagnosed condition. High index of suspicion helps to achieve the diagnosis preoperatively. Delay of the diagnosis and surgical intervention increases the morbidity and mortality rate. One-stage procedures may only be performed whenever the patients' general condition allows it. Early cholecystectomy should be carried out in patients with cholelithiasis.

### P171

### MANAGEMENT AND CLINICAL FEATURES OF ELDER PATIENTS WITH GALLSTONE

S. NAKAGAWA, A. KOSAKA, M. TANAKA
Department of Surgery, Matsusaka City Hospital, Matsusaka City, Mie 515, Japan

In order to clarify appropriate treatment selection in aged patients with gallstones, we analyzed clinical features in 150 patients treated during the past 3 years. The patients were divided, those over 70 years old (N=45, Group A) and those under 70 (N=105, Group B).

1) Incidence of the CBD stone was higher in Group A (32.7%) than in Group B(17.3%)> On chemical analysis, stones in Group A were composed mainly of pigment and those in Group B of cholesterol.

2) Bile culture yielded bacterial growth in the vast majority of patients in Group A, and incidence of acute cholangitis was higher in Group A (12.2%) than in Group B (4.5%).

3) Cardiovascular diseases and malignancies were more frequent in Group A (32.7%, 20.4%, respectively) than in Group B (10.0%, 4.5%)

4) Overall morbidity was the same in both groups.

5) Most of serious complications, such as AOSC, sepsis or MOF< occurred in patients in Group A and mortality rate of Group A was 8.2%.

Timely surgical intervention and intensive care are necessary in the management of elder patients with gallstone.

### P172

### COMPARISON OF SELECTIVE ENDOSCOPIC RETROGRADE CHOLANGIOGRAPHY WITH INFUSION INTRAVENOUS CHOLANGIOGRAPHY AS COMMON DUCT IMAGING TECHNIQUE PRIOR TO LAPAROSCOPIC CHOLECYSTECTOMY

P.D. NOTTLE
Alfred Hospital, Commercial Road, Pranhran, Melbourne, Victoria, Australia

A comparison of pre-operative selective endoscopic retrograde cholangiography (E.R.C.) and infusion intravenous cholangiography (I.V.C.) was carried out in a consecutive series of 408 laparoscopic cholecystectomies performed by one surgeon from June, 1990 to the present time.

In the first three hundred patients, selective E.R.C. was used as the method of duct imaging. The criteria for performing E.R.C. were the presence or a history of jaundice, altered liver function tests, gallstone pancreatitis, a duct greater than 8.0mm as measured on ultrasound or a stone in the duct seen on ultrasound. In the next one hundred patients, pre-operative infusion intravenous cholangiography using Biliscopin (meglumine iotroxate) was performed.

Using selective E.R.C., 43 (14%) patients had endoscopic imaging. Of these patients, 24 (8%) were found to have common duct calculi. Three (1%) further patients required post-operative E.R.C. to remove retained common duct stones.

In the I.V.C. group, ten patients (10%) were found to have common duct stones and all were removed pre-operatively. Only three patients had unsatisfactory imaging of the duct.

Both selective E.R.C. and I.V.C. are satisfactory methods of duct imaging, but I.V.C. appears to be a more accurate method of screening allowing E.R.C. to be used therapeutically rather than as a diagnostic tool.

### P173

### ULTRASOUND AND RADIOGRAPHIC EVALUATION OF PATIENTS FOLLOWING LAPAROSCOPIC CHOLECYSTECTOMY

R. PACE, R. SMITH, D. KOLYN, H. PYMAR, E. SAUERBREI,
Queens University, Kingston General Hospital, Kingston, Ontario, Canada

Laparoscopic Cholecystectomy has rapidly become the procedure of choice in many centres for the treatment of patients with cholelithiasis. Because of its rapid introduction, there has been little chance to study the normal post-operative changes due to this procedure. We prospectively studied the first 30 patients undergoing Laparoscopic Cholecystectomy at our institution by means of abdominal ultrasound and X-rays 24 hours after surgery. One patient required conversion to 'open' cholecystectomy because of adhesions and 1 patient developed post-operative bile peritonitis due to a leak from the gallbladder bed. Mean operative time was 72 minutes (35-150 min), with a mean post-operative stay of 2.0 days. The post-operative ultrasound of the upper abdomean was normal in 72% (21/29) with tiny loculations of fluid in the gallbladder fossa in 6, an errant residual stone near the porta in 1, and a small amount of perihepatic free fluid in 1 patient. The plain abdominal radiographs disclosed free intra-abdominal gas in 50% (14/28), but was a significant volume in only 4 of these patients. Abnormal gaseous distension of the large bowel was present in 79% (22 of 28), with a mean colonic diameter of 6.5 cm (range 5-12 cm). The surprising lack of ultrasound abnormality and the frequent finding of gaseous distension of the colon and small bowel on radiographic evaluation of patients following Laparoscopic Cholecystectomy must be borne in mind when assessing these patients following surgery.

### P174

### ELIGIBILITY FOR EXTRACORPOREAL SHOCK WAVE LITHOTRIPSY IN 596 CONSECUTIVE PATIENTS ATTENDING AN OUTPATIENT BILIARY CLINIC

P.W. PLAISIER, R.L.VAN DER HUL, R.DEN TOOM, H.G.T. NIJS, O.T. TERPSTRA
Dept. of Surgery, Univ. Hospital 'Dijkzigt', Rotterdam, The Netherlands

This study was designed to establish eligibility for Extracorporeal Shock Wave Lithotripsy (ESWL) in all consecutive patients (N=596), who visited our outpatient biliary clinic in the period April 1988 - April 1991. From all patients a history was taken and a physical examination was performed. Extended diagnostic work-up consisted of blood tests, ultrasonography and oral cholecystography. Results were examined retrospectively.

The studied population consisted of 445 female (mean age 48 yr) and 151 male (53 yr) and 80 patients were asymptomatic (13.4%). Quetelet-index > 27 was present in 33.4%. Ultrasound examination demonstrated no stones in 5.2%, 1 stone in 27.0%, 2-5 stones in 22.8%, 6-10 stones in 7.0%, >10 stones in 22.7% and unknown number of stones in 15.3%. Visualisation on oral cholecystography was present in 73.3% and calcified stones in 23.7% of the patients.

Exclusion criteria were: asymptomatic cholelithiasis, acute biliary disease, collapsed gallbladder, non-functional gallbladder, calcifications of stones >2mm, diameter of stones <5mm, and number of stones >10.

The ideal candidate for ESWL was considered an ASA I-II, symptomatic patient with a functional gallbladder, and 1 non-calcified stone with a diameter <21mm).

Conclusion: 28.1% of the population was eligible for ESWL and 8.1% could be considered ideal candidates.

**P175**

### MANAGEMENT OF GALLBLADDER LITHIASIS

A. PRINCIPE, A. MAZZIOTTI, M.L. LUGARESI, M. MORGANTI, A. FRENA, G. GOZZETTI
Clinica Chirurgica 2°, University of Bologna, Bologna, Italy

The high social importance of gallbladder lithiasis leads to find the definitive therapeutical solution, that should possibly be noninvasive, with a low mortality and morbidity rate and low costs. The conservative approach (dissolution with biliary acids per os or by direct contact or by the fragmentation of stones) for its limited indications (10-20% of pts) has an high rate of failure and recurrence (70-90%). The therapy of choice remains the cholecystectomy. Over the past 10 years 676 cholecystectomies where carried out : 166 pts had a complicated disease whereas 510 where operated on electively. In the first pts the morbidity rate was 15.6% with an operative mortality of 3.01% (mean hospital stay 14 days); in the elective pts the morbidity was 7.9% with an operative mortality of 0.3% (mean hospital stay 7 days ). From March to November 1991 43 additional cholecystectomies where performed under laparoscopic guidance (33 females and 10 males); mean age was 52 years, ranging from 22 to 76. Fourty-one pts had symptoms (1 empyema) and the remaining 2 where asymptomatic. There were no significant peri- or post-operative major complications. CONCLUSIONS Cholecystectomy represents the gold standard; the laparoscopic technique is to be preferred for its the numerous advantages: shorter post-operative stay (mean 2 days), better aesthetic results and absence of morbidity and mortality.

**P176**

### CHOLELITHIASIS IN YOUNG PATIENTS.

PROUSALIDIS J., TZARDINOGLOU E., FAHADIDIS E., APOSTOLIDIS A., KATSOHIS C., ALETRAS H.
A' Prop.Surg.Clinic Aristotelian University, Thessaloniki, Greece

The aim of this study is the presentation of 14 patients, 15-20 years of age, with biliary lithiasis and chronic cholecystitis admitted from 1970 to 1990 in our clinic. In the same period 1906 adult patients with gallbladder disease were also treated. Concomitant medicial or hematological problems in these young patients were not reported. Associated pancreatitis in one and jaundice in 2 cases were noted. The clinical picture was generally mild. Four patients refused operation. Ten patients underwent cholecystectomy. In addition, due to concurrent bile duct lithiasis, exploration of the common bile duct in two patients and billiodigestive anastomosis in one patient were performed. Operative cholangiography in 7 cases and choledochoscopy in 1 case were considered normal. There was one postoperative death, attributed to pulmonary embolism, concerning a 17 years of age lady suffering from morbid obesity.

Conclusion. Our experience shows that lithiasis in young people is a rare conditon and it has similar natural history with cholelithiasis in adults. Propably no special pathogenesis is implicated.

**P177**

### TACTICS FOR PATIENTS WITH CHRONIC CALCULOUS CHOLECYSTITIS IN EARLY STAGE

G.L. RATNER, B.B. KALUGHSKICH
Department of Surgery, Samara Medical Inst. Samara, U.S.S.R

Increasing number of patients, active tactics in surgery and considerable fraction of ones with postcholecystectomic syndrom-these are the reasons that made us raise the question about the gallbladder is deformed and its walls are sclerosed, if it dosen't function properly or is filled with bile, it should be ablated. However if the bladder is functioning and its walls aren't changed the operation should be only organopreserving. The results after cholecystectomy for patients in early stage of disease were bad. Out of 80 such patients we had only 17 good, 42 satisfactory and 21 bad cases. Appearance, anorganism can't sharply adapt oneself to existence without a bladder. Unfortunately, the "ideal" cholecystostomy hasn't justified our hopes, because it doesn't eliminate a single reason of forming stones. We made broad anastomosis of a bladder with choledoch after ablating all stones. It facilitated good evacuation and lessened the concentration function of bladder. They have been observed from 2 to 13 years. The results were good in 36 cases, in 7 were satisfactory and in 3- they were bad. Thus the gallbladder is an important functional organ which should be preserved for patients with early stage disease.

**P178**

### RESULTS OF STANDARD ELECTIVE CHOLECYSTECTOMY IN HIGH RISK PATIENTS - A PERSONAL EXPERIENCE

D.P. SANAN, P.K. JAIN, G. SINGH
Department of Surgery, .D.K. Hospital and G.N.D. Hospital. G.N.D. University, Amritsar, India

For Cholelithiasis standard elective cholecystectomy (SEC) still remains the operation of choice in high risk patients. However in high risk patients alternative methods for treatment of cholelithiasis are advocated.

The present study evaluates the results of S.E.C. in high risk patients treated over a period of two years(1988-1990).

Parameters used to include patients in high risk group are:

1) age>60 years. 2) Hypertension 3) Ischemic Heart Disease 4) Diabetes Mellitus 5) Jaundice 6) Associated common duct stones 7) Acute cholecystitis and its complications. A total of 211 patients underwent S.E.C. Of these patients 70 were included in the high risk group because of presence of one or more than one of the risk factors enumerated. All the high risk group patients had medical evaluations and treatment to optimize the medical status of the patients prior to surgery. Postoperatively these patients were closely monitored.

Complications such as Arrhythmias (n=5) wound infection (n=9) and incisional hernia (n=3); occured in 28.5% of high risk S.E.C. cases but with no in-hospital mortality in this series.

In our experience S.E.C. is a safe procedure when concomitant medical problems are identified and treated preoperatively. Careful postoperative monitoring and close medical cover are mandatory for good results.

### P179

### EFFECTIVENESS OF THE NEEDLE SCOPE IN LAPAROSCOPIC CHOLECYSTECTOMY

S. SHIOMI, S. KOBAYASHI, K. SAKAMOTO, T. MAEKAWA, N. SAKAKIBARA, N. ARAMAKI*
First Department of Surgery and Department of Gastroenterology*, Juntendo University School of Medicine, 2-1-1 Hongo, Bunkyo-ku, Tokyo, 113 Japan

Laparoscopic cholecystectomy (LC) has been established to supersede open cholecystectomy (OC) as a treatment of cholecystolithiasis. However, there are limitations in the application of LC compared to OC. In particular, there are many institutions which LC is not applied or is contraindicated in patients with previous abdominal surgery (PAS).

In this study, we used the needle scope, for the purpose of safely observing the peritoneal cavity and estimating the feasibility of LC in patients with PAS. There were six appendectomy, two upper, and two lower abdominal surgery of ten patients with PAS in thirty LCs. The needle scope was used in two patients with PAS.

The method was that of inserting the needle scope manufactured by Olympus Co. with a diameter of 3.4mm via 1/3 outside point of Monro-Richter's line in the left lower abdomen, prior to inserting the main trocars.

The results showed that, in both cases, the peritoneal cavity could be safely observed, and although adhesions of the peritoneal cavity were observed, LC was possible.

It is concluded that when the needle scope is aggressively used, even in patients with PAS, safe observations of the peritoneal cavity are possible, and even when adhesions are present, the application of LC is possible in some cases. Finally, it is considered that the needle scope is useful in patients with PAS.

### P180

### RETROSPECTIVE COMPARISON OF LAPAROSCOPIC CHOLECYSTECTOMY (LSK) VERSUS OPEN CHOLECYSTECTOMY (CZT) - ANALYSIS OF 202 CONSECUTIVE CASES

G. TOLKSDORFF, H.D. RAHN, H. PETERS, G. GAMSTÄTTER
Surgical Department, HSK, D-6200 Wiesbaden

202 elective cholecystectomies (LSK n=100, CZT =102) in 278 patients were carried out between October 1990 and October 1991. A conventional cholecystectomy was on principle performed in previous laparotomies, thrombopathies, pathologic cardiopulmonary findings and in patients who did not care about LSK, although they had been informed about the possibility of LSK. Life-threatening complications were not found in the two collectives. In two cases LSK was followed by a laparotomy (2%). 2 wound-healing impairments were detected after LSK compared to 5 wound-healing impairments after CZT. After conventional cholecystectomy a relaparotomy was necessary in 2 cases. Quick painlessness and discharge of 87% of patients until 3rd post-operative day after LSK were opposed to partly heavy pains and problems of mobilisation until 5th postoperative day after CZT. 91.2% of conventionally treated patients were hospitalised up to the 7th postoperative day.

Our previous results clearly show advantages of the LSK. For the moment we are in a phase of learning, in which we avoid this method in acute cholecystitis and empyemas. Accidental opening of gallbladder and difficult saving of gallstones show complications, which will be reduced with growing experience and further development of the instruments. Nevertheless the control of conventional cholecystectomy must be condifision for the performance of LSK due to the cleaning of eventual complications.

### P181

### THE INFLUENCE OF ROUTINE USE OF INTRAOPERATIVE CHOLANGIOGRAPHY ON THE INCIDENCE OF COMPLICATIONS AFTER CHOLECYSTECTOMY

TORDE L., ARSENIC V., RATKOV S., POPOV M., LATOVLJEV G., POPOV R., HESS DJ
Department of Surgery, General Hospital "Dr Dj.Joanovic" - Zrenjanin

The aim of the study is to show that routine use of intraoperative cholangiography may significantly reduce postoperative complications after cholecystectomy. We made a prospective study on 200 cholecystectomised patients in the period IX 1988 - III 1989. Patients are divided in two groups: A- control group consisting of 100 cholecystectomised patients without intraoperative cholangiography and B-also consisting of 100 patients with routinely used intraoperative cholangiography. Results are given in the following table, where the complications are separated by the time of appereance, specific signs and symptoms:

| Type of complication | group A | | group B | | Total | |
|---|---|---|---|---|---|---|
| | .n | % | n | % | n | % |
| Intra op. | 1 | 5,26 | 0 | 0.00 | 1 | 3.57 |
| Early | 4 | 21.05 | 3 | 33.33 | 7 | 25.00 |
| Intermed. | 2 | 10.53 | 3 | 33.33 | 5 | 17.86 |
| Late | 12 | 63.16 | 3 | 33.33 | 15 | 53.57 |
| Total | 19 | 100.00 | 9 | 100.00 | 28 | 100.00 |

Conclusion: Routine use of intraoperative cholangiography significantly reduces the number of complications (A:B = 19:9) which is statisticaly significant (chi-squared test, $p<0.05$).

### P182

### IS THERE AN ANSWER TO THE DILEMMA : CHOLECYSTECTOMY WITH OR WITHOUT DRAINAGE?

G.V. VELMACHOS, I.TSAGARIDOU, V.SOTIROPOULOU, G.S.VORONOS, D.BASTAS
"SOTIRIA" Gen. Hospital of Athens, Athens, Greece

The aim of the present comparative study, which was performed on patients(pts) cholecystectomized for non-acute pathology of the gallbladder was to investigate the incidence and the significance of post-cholecystectomy fluid collections detected by ultrasound and to correlate the occurence of these collections with or without drainage. A total of 235 consecutive pts underwent cholecystectomy for non-acute pathology. 132(56.1%) were women and 103(43.9%) were men. The age of the pts range from 22-82 years (mean 56.8). The gallbladder was removed and the gallbladder bed was sewn with polyglactin 910 in all cases. 85 pts(group A) were not drained and 150 (group B) were drained by a Latex tube (7mm wide) placed in Morrision's pouch. The volume and nature of the fluid drained was recorded, and the drain was removed after 48 h.

All pts were examined 24,48 h and 7 days after cholecystectomy by the same ultrasonographers. Scans of the right upper quadrant were obtained longitudinally and transversely. 47 of 85 ptsss (55.29%) of the group A were found to have subhepatic fluid collections 20-60 ml the 1st postoperative day and 0-25 ml the 2nd post .day. The other 38 pts were found to have small loculated collections, less than 20 ml. Despite the drainage 65 of 150 pts (43.3%) of the group B developed fluid collections less than 25 ml of 1st and the 2nd post-operative day. The amount of fluid drained the 1st day was 65 ml (5-90 ml) and the 2nd day 15 ml (5-30 ml). The nature of this fluid was blood-stained serum. The 7st post. day the fluid collections were not significant. No subphrenic collections were found in all 235 pts. This study suggests that the surgical drainage after every uncomplicated cholecystectomy is unnecessary.

**P183**

### THE EFFECT OF CONTINUOUS INTRAVENOUS INDOMETHACIN INFUSION ON POSTOPERATIVE PAIN AFTER CHOLECYSTECTOMY

K. VELMACHOU, A. SOFIANOU, H. KOUTSOPOULOPU, H. KOUYIOUMTZOYLOU, C. KOUTSODIMA, I. LEKATSAS,
Anaesthetic department, "KAT" General Hospital, Athens, Greece

The influence of indomethacin on the need for postoperative analgesia was investigated in a double-blind study of 60 patients ASA I & II, mean aged 48±9 years, scheduled for cholecystectomy under general anaesthesia. The patients were randomly allocated in two groups. Group I received indomethacin 0,8mg/kg B.w. i.v. bolus just before the induction of anaesthesia, followed by continuous infusion of 5-7,5 mg/h over 20h. Group II was the control group and received only Ringer's Lactated. Postoperatively in both groups supplement doses if D-propoxyphen HCL I.M. were given as needed.

The total amount of D-propoxyphen HCL during the follow-up period was registered for each patient as well as the incidence of side effects. The postoperative pain was evaluated by Visual Analogue Score.

The D-propoxyphen HCL dose(mean±s.d.) during the postoperative observation was lower in the indomethacin group (52,5±32,06 mg) than in the control group (175±53,3 mg) (P<0,001). In the indomethacin group significantly fewer patients (14/30) needed D-propoxyphen HCL I.M. more than once during the follow-up period compared with the control group (26/30)(P<0,001). The pain score values were lower in Group I (P=0,05).

In conclusion postoperative pain after cholecystectomy cannot be totally prevented by an invenous indomethacin treatment regimen. However the need of additional analgesia diminished in the postoperative period.

**P184**

### LAPAROSCOPIC CHOLECYSTECTOMY : THE INITIAL 54 CASES

M.D.YANG, C.R. HUANG, C.C.WU, T.C.WU, T.J.LIU, AND F.K.PENG
Division of General Surgery, Dept. of Surgery, Taichung Veterans General Hospital, Taichung, Taiwan, R.O.C.

Laparoscopic cholecystectomy quickly emerged as an alternative to open cholecystectomy. Between April 1991 to November 1991, we performed 54 laparoscopic cholecystectomies with no deaths and a complication rate of 5.6%. There were 32 women and 22 men, with a mean age of 56.3 years (range 23 - 78 years). Mean operative time was 110 minutes. Mean hospital stay was 3 days. The time of return to full activity was 7.5 days after operation.

The hospital costs demonstrates a modest cost advantage over standard open cholecystectomy. (NT$50,987 versus $76,460). There was no CBD injury, one intestinal injury and one wound infection. Eleven patients had prior low abdominal surgery. Six patients required conversion to open cholecystectomy. Laparoscopic cholecystectomy appears to offer a reduced hospital stay, a decreased recovery interval, diminished postoperative discomfort and reduction in health care expenses. It can be performed safely by experienced surgeons in properly selected patients.

**P185**

### CLINICOPATHOLOGICAL CHARACTERISTICS OF SILENT GALLBLADDER STONES

M. YOSHIDA, K. SHIMADA, T. SUZUKI, H. YOKOTA, G. KANEDA, S. FUNAMOTO, N. KOBAYASI, K.ASO, H.MIENO, K.SATO, H.OHMIYA, T.HIKI. A. KAKITA
Department of Surgery, Kitasato University, School of Medicine, Kanagawa, Japan

Clinicopathological chracteristis of 40 resected patients were studied to evaluate the surgical indications of the silent gallbladder stone disease. Of 40, 38 patients underwent cholecystectomy during lapalotomy for other abdominal diseases, and two patients were performed cholecystectomy only aimed at removing the disease. Oral cholecystography showed impaired visualization of the gallbladder in 13 patients (32.5%), which meant gallbladder functions were absent. In histopathological study of the resected gallbladder, chronic cholecystitis was found in all patients (100%), with dysplasia in three patients (7.5%) and adenocarcinoma in one patient (1.1%). The degree of imflammation of the gallbladder correlated incidence of impaired visualization in oral cholecystography.

In conclusion, cholecystectomy is recommended for the patient with impaired visualization of the gallblader in oral cholecystography.

**P186**

### PERCUTANEOUS GALLBLADDER CHOLANGIOGRAM TO DETERMINE BILIARY ANATOMY AND DETECT DUCT STONES PRIOR TO LAPAROSCOPIC CHOLECYSTECTOMY

CD JOHNSON, CN HACKING, K DEWBURY
University Surgical Unit, & Department of Radiology, Southampton General Hospital, Southampton

Operative cholangiography during laparoscopic cholecystectomy is difficult, time consuming and may present the surgeon with a delicate choice between leaving a possible stone in the duct, or converting to an open operation for duct exploration. Alternatively laparoscopic duct exploration may be attempted, but this can be time consuming. Preoperative diagnosis of duct stones helps planning operation lists. Cholangiography also helps to delineate biliary anatomy, but duct damage may occur before operative cholangiography (OC) has been performed. We report the preliminary results of percutaneous gall bladder puncture for cholangiography (PGBC) prior to laparoscopic cholecystectomy.

Under local anaesthesia within 24 hours of surgery, the gallbladder is punctured transhepatically and bile is aspirated. Contrast is injected to obtain a cholangiogram. Fifteen patients have been studied. PGBC failed in three and clearly demonstrated the duct anatomy in 12. One patient was found to have a definite common duct stone and underwent endoscopic sphincterotomy prior to laparoscopic cholecystectomy. One patient with possible stone on PGBC elected to defer intervention on the duct until after postoperative assessment of symptoms and liver function. OC was not attempted in one patient (after ES), and failed in five. Good films were obtained in six patients, unsatisfactory in three.

In patients with a functioning gallbladder PGBC provides excellent cholangiograms and can detect common duct stones prior to laparoscopic cholecystectomy.

### P187

### THE POSSIBILITIES OF EXTRACORPOREAL SHOCK WAVE LITHOTRIPSY (ESWL) IN THE TREATMENT OF CHOLEDOCHOLITHIASIS FOLLOWING FAILURE IN ENDOSCOPIC REMOVAL

M.BULAJIC, M. MILICEVIC, R. GRBIC, B. STIMEC, M. MARKOVIC, D. POPOVIC, L. PANDEY
Institute of Digestive Diseases, University of Belgrade, Belgrade, Yugoslavia

Unsuccessful endoscopic removal of common bile duct stones can be solved by means of extracorporeal shock wave lithotripsy (ESWL). During a period of 2 years, 20 patients (7 male, 13 female, mean age 68 years) with massive choledocholithiasis (multiple, casting and big solitary stones) underwent ESWL treatment, using the Siemens Lithostar Plus. Sixteen patients had previous endoscopic sphincterotomy. After an unsuccessful mechanic extraction a nasobiliary catheter was placed in 13 patients. Six patients had external T-tube drainage. All the patients have previously undergone Cholecystectomy. The extracorporeal shock wave lithotripsy was conducted in the prone position. Fragmentation of stones was achieved in 17 (85%) patients. Eleven patients spontaneously eliminated the fragments during initial treatment, i.e. total clearance of CBD was achieved; whereas in 6 patients the fragments were extracted using a Dormia basket, after extracorporeal shock wave lithotripsy. The number of the applied shock beats and energy ranged between 3500-5000, and 16-17.5 kV per patient, depending upon the size, shape and number of stones. The average number of sessions per patient was 1.5. During ESWL 17 patients (85%) received IV analgosedation, and there was no need for general anaesthesia in any case. No complications were recorded. In 3 patients where ESWL failed, surgical cholecystectomy was performed without complications.

### P188

### LAPAROSCOPIC CHOLECYSTECTOMY - INITIAL EXPERIENCE

M. KRAWCZYK, K. ZIENIEWICZ, W. PATKOWSKI
Department of General Surgery & Liver Diseases, Medical Academy, Warsaw, Poland

The aim of this report is a presentation of our own experience in laparoscopic cholecystectomy. From June 1990 till now we have performed 41 laparoscopic cholecystectomies in our department. There were 39 female and 2 male patients with an average age 38.5 (range : 23-67 years). The indication for this procedure was symptomatic cholecystolithisis. In 29 cases it was biliary colic, in 11 cases chronic cholecystitis and in one case acute cholecystitis. The diagnosis was confirmed by ultrasonography. Operation time ranged from 245 min till 65 min (average : 95 min). There was no bleeding or bile duct injuries during the procedure. None of the patients required conversion of the laparoscopic procedure to an open cholecystectomy. Average hospitalization was 3.5 days. There was no mortality. The morbidity was 7.3% (umbilicus wound infection was noticed in 3 cases). The median time to full activity after discharge was 15.5 days.

It is concluded that laparoscopic cholecystectomy offers a new idea in the treatment of cholecystolithiasis.

### P189

### NEW DRAIN PLACEMENT TECHNIQUE DURING LAPAROSCOPIC CHOLECYSTECTOMY

P. METZGER, M. GAMAL, I. ROZSA, J. KISS
Postgraduate Medical University, Department of Surgery, Budapest-Hungary

During Laparoscopic cholecystectomy the placement of a suitable subhepatic suction-drain is sometimes a difficult, time wasting manoeuvre. Authors worked up a new quick method for the percutaneous laparoscopic placement of the suction-drain to the abdominal cavity, and they appled this technique successfully during 200 laparoscopic cholecystectomies.

### P190

### MODIFIED PERCUTANEOUS ENDOSCOPIC CHOLECYSTOPOLYPECTOMY

HUO FENG, LUO MING-YI, MA YUAN-GUI, ZHANG ZHI-CHENG AND QIU BAO-AN
Yanjing Hospital, 00037 Beijing China

From March to December in 1991, we have performed modifed percutaneous endoscopic cholecystopolypectomy with primary suture of the gallbladder in 32 cases. Twenty four patients have multiple polypoid lesions of the gallbladder, eight have single polyp, nine have both polyps and calculae. A total of 72 protuberant lesions measuring over 0.5 cm in diameter were resected. The pathologic studies showed cholesterol polyps in 22 cases, and adenomatous polyps in 10, including four inflammatory polyps and two with active proliferation.

The modifed percutaneous endoscopic cholecystopolypectomy begins with B ultrasound scanning of the gallbladder to locate the fundus, a subcostal incision over the site of the fundus , lift the fundus with a grasper and anchor with two to three stitches. Insert an Olympus A 5037 laparoscope into the gallbladder through a stab wound. Resect the polypoid growth through the endoscope and send for frozen section. Do an intraoperative cholecystography and see that the cystic duct is patent. If the frozen section proved benign, close the gallbladder with 4/0 nontraumatic suture in two layers without drain. Patients can get up and liquid diet given in the first postoperative day and after operation when indicated.

All patients in the series have recovered uneventfully, 16 cases followed over a period of three months showed normal function of the gallbladder.

**P191**

### A PRACTICAL AND COST-EFFECTIVE WAY TO BEGIN LAPAROSCOPIC SURGERY

LUO MING-YI, MA YUAN-GUI, HUO FENG, QIU BAO-AN, ZHANG ZHI-CHENG
Yanjing Hospital, Beijing 100037, P.R. China

Sharing special instruments with other departments of the hospital and using oxygen bag as phantom for training we began to practise laparoscopic surgery since April 1991 and have succeeded in performing laparoscopic cholecystectomy, percutaneous cholecystolithotripsy and polypectomy on 72 patients. We invested only a Storz 26033 laparoscopy, a TV monitor system and a few items of endoscopic instruments. The other instruments, including a $CO_2$ insufflator (Olympus PNE-C) a lithotriptor (Wolf 2135 Riwolith), a high frequency generator (Valleylab Force 4) and a light source (Olympus CLE-F) were shared with other departments of the hospital. Thus we were able to save 50% of the investment and practise laparoscopic surgery earlier than expected.

To share costly and delicate instruments owned by different departments mutual understanding and support are mandatory. To make a reliable combination of sophisticated instruments for laparoscopic surgery, it is necessary to check the functions and specifications of every instrument, to replace with adequate set having similar function if necessary, and to get adaptor fit for different types of plug and socket on instruments supplied by different factories or of different versions.

To master the technic of manipulating through trocar sheath in an inflated abdominal cavity while watching on the TV monitor, we used oxygen bag as a phantom for drilling, which proved successful. The problem of training became simple since an oxygen bag is readily available in every hospital and anaesthesia is not required as in animal experiment. The bag can be used repeatedly and the chance of contamination is low.

In training for laparoscopic surgery, the team should learn to maintain pneumopernitoneun properly, to get used to loss of stereo-vision, to accommodate complicated orientation and coordination. The use of long high frequency hook can be practised during routine dolecystectomy.

**P192**

### FACTORS INFLUENCING TUMOR RECURRENCE AFTER RESECTION OF HEPATOCELLULAR CARCINOMA IN CIRRHOTIC LIVER

J. BALSELLS, H.ALLENDE, JL LAZARO, E.MURIO, R.CHARCO, I.DIAZ, C.MARGARIT
Liver Transplantation Unit. Department of Surgery Hospital General Vall d'Hebron. Universidad Autonoma, Barcelona, Spain

In the last 4 years, 33 hepatic resections for hepatocellular carcinoma were performed in 31 patients with liver cirrhosis in our Unit. Mean age of patients was 63±7 years. Etiology of liver cirrhosis was: alcoholic in 8, postnecrotic in 15 and others in 8. Serology for Hepatitis C was positive in 55% of patients. Tumor size was 4.4±2.9 cm. Most patients were treated with limited resection. Operative mortality was 13%. Follow-up ranges from 2 to 47 months (mean 16±12). Tumor recurrence was diagnosed in 59% of the patients: 4 distant metastasis and 12 intrahepatic recurrences (6 adjacent to resected segments, 4 in a distant segment and 2 multiple or diffuse recurrences). Nine recurrences were found within the first year and seven after 1 year. Two patients were reoperated in order to resect a nodular recurrence.

Demographics and pathologic characteristics of cirrhosis and hepatocellular carcinoma were studied for analysis of risk factor of recurrence. Hepatitis B cirrhosis, macronodular cirrhosis, non trabecular and undifferenciated tumor, tumor size bigger than 3 cm, presence of satellites or more than one nodule and vascular invasion were associated with a higher incidence of recurrence. Overall survival rate is 48% and actuarial survival at 1 and 3 years are 69% and 32%.

Conclusion: Recurrence of hepatocellular carcinoma in cirrhotic patients is high with surgical resection alone. Therefore, adjuvant chemoembolization might be indicated and liver transplantation may be the best appraoch in young cirrhotic patients with small nodular hepatocellular carcinoma.

**P193**

### PRIMARY TUMOURS AND TUMOUR-LIKE CONDITIONS OF THE LIVER

EMM.BANDOUVAS, J. ALEXANDRIDES, J,PARHARIDOU, N.ALEXANDRIDOU, E.PAPAEFTHIMIOU, A.TAGGALOS, J.KAPELLAKIS, GIANNIKOS, J.BISSODIS, S.SERMAGIAS
"Helena Venizelou" Hospital, 2, E.Venizelou Sq., GR-11521 Athens, Greece

We present representative microscopic pictures belonging to systematically classified primary tumours and tumour-like conditions of the liver. Also, we intend to make correlations among related parameters as: etiology, symptoms, microscopic findings, survival, therapy and prognosis. We are referred to 230 malignant cases, 85 benign and 110 tumour-like conditions.

Almost all benign tumours and tumour-like conditions had been curable, by excision except when uncurable associated syndromes were being, e.g. cirrchosis. Many of these benign conditions did not require any treatment. In malignant tumours, early surgery gave in favourable cases survival of some years and rarely cures. In most cases, survival was counted in weeks or months.

**P194**

### THE SECOND SURGICAL TREATMENT FOR THE RECURRENCE OF PRIMARY LIVER CANCER AFTER CURATIVE HEPATECTOMY

CHEN JI-SHENG, OU QING-JIA, WANG YING
Laboratory Memorial Hospital, Sun Yat-Sen University of Medical Sciences, Guangzhou, China

During the past 10 years we have treated 660 patients with primary hepatocellular Carcinoma (HCC). 124 of them were treated by liver resection (18.8%). Thirty-two of the patients with resectable HCCs have developed intrahepatic metastases after operation. 28 men and 4 women average 42.5 years (range 9 to 65 years), 30(93.7%) with postnecrotic cirrhosis and 5(15.5%) with portal hypertension. For the patients with cancer recurrence, 3.2% (n=4) of them underwent a second resection. The mean survival time was 26.8 mos after the second resection. 22 of the patients had already been treated with injecting absolute ethanol directly into the tumor under ultrasound guidance. 8 patients died after the injecting. The mean survival time was 8.5 mos. Twelve of the patients had been treated with transcatheter hepatic arterial chemotherapy and embolization (THACE-Lipiodol, 5Fu MMC ADR) and/or hepatic artery ligation and infusion chemotherapy. The mean survival time was 16.8 mos. Control group only 4.5 mos. It is concluded that repeat hepatectomy for recurrent liver cancer as an important approach for improving the surgical result has been proved. A typical resection are reasonable and acceptable for HCC, especially, for recurrent HCC. Ethanol injection therapy is an effective, palliative treatment for recurrent HCC, especial for the single nodule. TAE/HAL+HA I provides an effective, palliative treatment for recurrent HC. But TAE did not improve the longterm survival time.

### P195

### CRITERIA FOR CURATIVE RESECTION IN HEPATECTOMY FOR HEPATOCELLULAR CARCINOMA LARGER THAN 5 CM IN DIAMETER

T. HARADA, S. KODAMA, K. MATSUO, Y. WADA. S. IKEDA,
1 st Department of Surgery, School of Medicine, Fukuoka University, Fukuoka, Japan

The present study was designed to determine the criteria for curative resection in hepatectomy for hepatocellular carcinoma larger than 5 cm in diameter. The extent of the resected liver was closely related to recurrence, and the complete removal of the involved segments was found to be essential for curative hepatectomy. Patients with satellite nodule showed high incidence of recurrence; in particular, carcinoma recurred in every patient with multiple satellite nodules scattered over unilateral lobe, in spite of macroscopic tumour removal. On the other hand, curative resection could be achieved in patients with portal tumour thrombi within the 2nd branches of the portal vein when the tumour tissue including tumour athrombi was removed en bloc. Negative resection margin, i.e., microscopic absence of neoplastic cells within 5 mm of the margin of parenchymal dissection, did not affect the recurrence rate. On the basis of these findings we define complete removal of involved segments containing satellite nodules and portal tumor thrombi as curative resection, though curative hepatectomy cannot be achieved in patients with satellite nodules scattered over unilateral lobe and/or with tumour thrombi in portal truncus.

### P196

### A CASE OF HEPATOCELLULAR CARCINOMA IN A PATIENT WITH APLASTIC ANEMIA TREATED WITH ANABOLIC STEROID AND ORAL CONTRACEPTIVES

A. KOSAKA, A.NAKAGAWA, M. TANAKA, F. KOMADA*, M.TSUDA*
Department of Surgery, Department of Internal Medicine* Mastsusaka City Hospital
Matsusaka City, Mie 515, Japan

We report hepatocellular carcinoma in a 35-year-old female, who had undergone treatment with oxymetholone (60mg/day) for 24 months for aplastic anemia which had developed when she was 32. She was also receiving treatment with oral contraceptives (ethynodiol acetate 1mg, ethynylestradiol 0.05 mg) to control menstrual bleeding. Computed tomography and ultrasonography revealed three masses ranging from 1 to 5cm in diameter in the left lateral segment of the liver and four masses less than 2cm in diameter in the right hepatic lobe and medial segment of the liver. HBs-antigen, AFP, and CEA were negative. Under the diagnosis of hepatocellular carcinoma in the lateral segment and adenomas in the right hepatic lobe and medial segment of the liver, lateral segmentectomy was performed. The resected liver specimen was not cirrhotic. Histologically, all nodules were confirmed to be hepatocellular carcinoma. The tumour and liver tissue were positive for estrogen receptor. One month later, an hepatic arterial access device was implanted to deliver chemotherapy to remnant hepatic tumours. It is thought that the liver tumours in this case may have been induced by long-term use of anabolic steroid and oral contraceptives. We recommend that ultrasonographic and computed tomographic examination be used to screen patients with undergoing long-term anabolic steroid or oral contraceptive therapy for liver masses.

### P197

### RESECTION FOR HEPATOCELLULAR CARCINOMA WITH INVASION TO THE ADJACENT ORGANS

T.J.LIU, C.C. WU, C.J. HUANG, R.Y.HUANG, M.D. YANG, T.C. WU, F.K.P'ENG
Division of General Surgery, Dept. of Surgery, Taichung Veterans General Hospotal, Taichung, Taiwan, R.O.C.

From January 1987 to June 1991, 78 patients with hepatocellular carcinoma (HCC) underwent resection. Combined resection of the adjacent organs was carried out 19 times in 17 patients (21.8%) due to direct invasion of the tumor. The organs which were resected concomitantly were diaphragm in 13, stomach in 3, colon in 2, pancreas in 2, right kidney in 1, right adrental in 1, abdominal wall in 2 and right lung in 1. One patient underwent surgery three times for recurrent tumors. No operative death occurred in any patient and 5(29.4%) patients had postoperative complications. The primary tumor in all patients except one was larger than 6cm (average 12.9cm, range 4-21cm). The location of the main tumor in the right lobe was found in 11 patinets, left lobe in patients and both lobes in 1 patients. Until November 1991, eight patients died with a mean survival time of 9.5 months (range 5-18 months). Recurrent disease was the cause of death in 7 patients. The other 8 patients without recurrent disease survived for 6,8,8,9,12,18,24 and 59 months. One patient has been alive for 12 months wigh recurrent liver tumor. We conslude that when HCC invades to other organs the disease has reached an advanced stage; however, aggressive, combined resection of adjacent organs can provide some long term survivors.

### P198

### SURGICAL RESECTION OF LARGE HEPATOCELLULAR CARCINOMA WITH CENTRAL LOCATION

T.J. LIU, C.C. WU, and F.K. P'ENG
Dept. of Surgery, Taichung Veterans General Hospital, Taichung, Taiwan, R.O.C.

In Taiwan, hepatocellular carcinoma (HCC) is usually combined with chronic hepatitis and liver cirrhosis. The prognosis of large HCC (tumour size >5cm) is poor. Resection of large HCC located in the central area of the liver (such as liver hilum or dome region), therefore, remains challenging. Since August 1989, intraoperative ultrasonography and hepatic inflow blood clamping has been used during hepatectomy, any economic resection of HCC becomes possible.

In the past 2 years, we resected 15 HCC patients whose main tumor was located in the central area of the liver (defined as Coninaud's segment 4,5,8). The size of the main tumor in all patients was larger than 6cm. Of them, liver cirrhosis was found in 11 patients. The Child's grading of their liver function revealed 12 in Child's A and 3 in Child's B category. However, normal ICG 15' retension rate (<10%) was found in 7 patients. No patient died after operation, but patients (33.3%) had postoperative complication. During the follow aup periods till now, three patients died of recurrent disease (survival time 4,6 and 12 months),one patient had recurrent disease and has been alive for 14 months. The other 11 patients, who have had no evidence of recurrent tumor, have survived for 6-26 months (average 17.6 motns). The follow up period is too short to jump to any conclusion. Since surgical resection is the best way to treat large HCC, aggressive resection in selected patient with large HCC over central liver is till recommended.

### P199

### CLINICAL AND PATHOLOGICAL OBSERVATIONS OF TRANSCATHETER CHEMOEMBOLIZATION COMBINED WITH HEPATECTOMY FOR PRIMARY HEPATIC CARCINOMA

LIU YONGXIONG et, al.
Dpt. Hepato-biliary surgery. Great Wall Hospital, China

Transcatheter chemoembolization (TCE) followed by laporotomy were performed in patients with primary hepatic carcinoma (HCC). Each patient received TCE of 1-5 times with an interval of 4-6 weeks. The embolizing reagents included: mitomycin C (20-40mg), adriamycin (20-60mg) and 40% lipiodol (2-10ml), The clinical results showed marked decrease or normalize in AFP levels and reduction in tumour size. Of the 12 patients, partil hepatectomy were carried out in eleven. In the another one not operated, she is with extensive abdominal metastasis and Contact matastusis with epiple. The histopathological study showed:tissues necrosis and fibrosis at different stages in liver lesion especially in tumour area. Apart form sclerotic lesion one or two liver daughter nodules were found in two cases, residusla liver cancer were seen in all of th 11 cases. One case with local recurrence and metastasis in abdomen one year after liver resection was treated with hepatectomy once again; the other two with right lung metastatic tumour four to six months after operation was treated with transcatheter pulmonary arterial embolization; another one had slight increase in AFP levels two months after resection.

We considered TC could not replace the surgery operation but only a part of combined treatment of liver carcinoma. It is therefore suggested that indication of TCE are: the unresectable HCC's resectable HCCs with poor liver function, those associated with portal hypertension and recurrent HCCs to prepare for further surgery.

### P200

### EXPECTATIONS, POSSIBILITIES AND LIMITS OF SURGERY FOR HCC IN CIRRHOTIC PATIENTS

G. GOZZETTI, A. MAIOTTI, G.L. GRAZI, E. JOVINE, A. FRENA, W. GRIGIONI, A. CAVALLARI A
Clinica Chirurgica 2°, University of Bologna, Bologna, Italy

The surgical approach in pts with HCC in cirrhotics has evolved over the past ten years.

PATIENTS AND METHODS: Over the period '82'-'91', 282 cirrhotics have been admitted at our Institution because of HCC. During the pre-operative work-up 105 pts were excluded from surgical treatment mainly for intrahepatic tumoral spread or portal vein thrombosis. One hundred sixty pts were operated on: 100 curative resection, 5 palliative resections and 12 transplantations were performed. In spite of the accuracy of the pre-operative work-up, a large number of pts (37.5%) where excluded from resection at laparotomy.

RESULTS: The operative mortality of the resected pts is decreased from 16.1% to the 7.4% of the last 5 years and it is strictly correlated to the Child Pough score. The recurrence rate is 34.5% in the group of the resected, while it is 25% in the transplanted pts. The incidence of recurrence is correlated with the AFP level, presence of symptoms, no preoperative TEA, perioperative blood transfusion, absence of tumoral capsule, microvascular thrombosis. Five year actuarial survival is 41.6% for the transplanted pts and 32.7% for the resected pts.

DISCUSSION : Considering the long term results it remains controversial which therapeutical choice, between transplantation and resection, is more indicated in cases of HCC on cirrhosis.

### P201

### THE ROLE OF HEPATECTOMY IN THE TREATMENT OF HEPATOCELLULAR CARCINOMA

TAKESHI NAGAO[1], FUYO YOSHIMI[1], NOBUHIRO KAWANO[2], TETSUICHIRO MUTO[2,] SHIN OHNISHI[3], AND MICHIO IMAWAREI[3]
From the Onstitute of Medical Scinence[1,] the First Department of Surgery[2,] and the Third Department of Internal Medicine[3,] University of Tokyo, Japan

The long-term results of hepatectomy (Hx) for hepatocellular carcinoma (HCC) have not been satisfaxtory yet. The high incidence of postoperative recurrence has been a major obstacle against its improvement. In the current study, we compared the results of Hx to those of transcatheter arterial embolization (TAE). The records of patients who had undergone HX or TAE for their HCC from 1984-1990 were investigated. The patients without cirrhosis or with Child C cirrhosis were excluded because of the small number of patients in these groups. The survival curves of patients eith Child A cirrhosis were not differnet between Hx group and TAE group. The incidence of solitary tumour was significantly higher in Hx group. However, we could not find any differences in the other backgound factors, such as age, sex, HBs antigen, and tumour size. The same results were obtained in patients with Child B cirrhosis. The results of this study will cast a doubt about the role of surgeon in the treatment of HCC.

### P202

### THE STUDY OF NINETEEN CASES WITH HEPATOCELLULAR CARCINOMA THREE CENTIMETER OR LESS THAN THREE CENTIMETER IN DIAMETER

A.OGURO, H.TANIGUCHI, K.MIYATA, Y.UESHIMA, K.TAKEUCHI, N.TSUKUDA, A. HAGIWARA, T.YAMANE,T.YAMAGUCHI, K.SAWAI, O.KOJIMA &' T.TAKAHASHI
First Department of Surgery, Kyoto Perfectural University of Medicine, Kyoto, Japan

Nineteen patients with hepatocellular carcinoma (HCC) 3 cm or less than 3 cm in diameter were studied in order to ascertain whether it was possible to discriminate HCC 2 cm or less than 2 cm in diameter from HCC 3 cm or less than 3 cm and larger than 2 cm in diameter. From April 1989 to July 1991 hepatic resections were performed on 9 cases fo HCC containing 12 tumours 2 cm or less than 2 cm in diameter (group A) and 10 vcases of HCC containing 11 tumours which were 3 cm or less than 3 cm and larger than 2 cm in diameter (group B). Preoperative serum alpha-fetoprotein (AFP) level, findings of ultrasonography (US), computed tomography (CT), magnetic resonance imaging (MRI) and angiography, and pathological findings were compared in these 2 groups. The following results were obtained: (1) serum alpha-fetoprotein level of group A (129.4ng/ml) were lower than that of group B (426.9ng/ml); (2) group B tended to be detected as hyperechoic lesion by ultrasound; (3) uninodular tumors were more frequently seen in group A than in group B; (4) group A tended to have lower grade of Edmondson's classification than group B. The conclusion was as follows; it was very difficult to discriminate group A from group B.

### P203

### HEPATOCELLULAR CARCINOMA (HCC) IN CANADA; CLINICAL FEATURES AND A MODEL FOR PROGNOSIS.

S.C. PAPPAS, D. HEMPHILL, A. PETER, C. LEVINTON, S. HANNA Sunnybrook Health Sciences Centre, Toronto Canada

HCC as diagnosed in Canada may differ from that diagnosed in other parts of the world. To further assess the clinical features and develop a model for prognosis, a database of 70 patients with proven HCC was reviewed. Clinical features present at the time of diagnosis were analysed by Cox regression analysis with stepwise reduction methodology to develop a model for prognosis; survival was estimated by the Kaplan-Meire method, Median survival was 14.1 weeks; 7 patients survived longer than 2 years. Survival was not related to age (mean 62.0 yrs), sex (58M,12F), presence of cirrhosis (43/58,74%), etiology of liver disease (46% ETOH, 27% cryptogenic, 20% hep B, 7% other), evidence of extrahepatic spread (18/63;29%), size or location of tumour and singles vs, multiple tumours. By univariate analysis, bilirubin, AFP levels, and the presence of symptoms, ascites or encephalopathy at presentation were predictive of survival; by Cox regression analysis, a model for prognosis was developed using AFP levels and ascites as the two independent predictive variables. Surgery was felt to be possible in 8 patients, performed in 6 cases and was associated with a significant improvement in survival (median survival 55.7 wks vs. 13 wks, $p<0.05$). These result confirm that HCC in Canada, as in other countries, is associated with a poor prognosis but differ in other aspects from prior reports, both outside and within North America, with regards to prognostic factors. Further cross-validation of the prognostic model is required; this may facilitate thereapeutic trial planning.

### P204

### RADIOGRAPHIC FEATURES AND PROGNOSIS OF HEPATOCELLULAR CARCINOMA (HCC) IN CANADA

D. SALONEN, C. LEONHARDT, D. HEMPHILL, S. HANNA, S.C. PAPPAS
Sunnybrook Health Sciences Centre, Toronto, Canada

To determine the radiographic features of HCC as seen in Canada and their relationship to prognosis, multiple imaging studies of 40 patients with proven HCC were reviewed; patients were selected from a larger data base of 70 patients eith HCC on the basis of the availability of imaging studies for review. Six radiographic features were analysed for prognostric value in 35 patients by univariate and Cox regression analysis. Cirrhosis was present in 27/35 patients (77%) with the etiology of liver disease including ETOH (17), cryptogenic (9), hepatitis B (6) and miscellaneous causes (3). Median survival after diagnosis was 14.1 weeks. HCC was detected by CT in 27/27 cases; in 6/40 patients HCC was not detected by ultrasound (US) study. The appearance of HCC at US varied, with 13/34 (40%) hypoechoic, 7/34 (20%) hyperechoic and 14/34 (40%) mixed echogenic lesions. 6/35 (17%) cases demonstrated a diffuse infiltrative pattern of tumour involvment without discrete lesions. Tumours ranged in size from 2 to 16 cm with encapsulation suggested by an imaging study 7/35 (20%) cases. Vascular involvement was present in 8/35 (23%) cases. Regression analysis indicated no relationship between survival and tumour encapsulation, size, location, vascular involvement, echogenicity, or discrete versus diffuse infiltrative appearance. No radiographic feature, including tumour size, correlated with AFP levels (elevated in 25/35 (71%) of cases). These results confirm the variable radiographic appearance of HCC but differ in other aspect from previous reports, particularly those from outside North America. These differences may be related to varying etiology or modes of presentation and further studies are indicated.

### P205

### SELECTION OF TREATMENT MODALITIES OF HEPATOCELLULAR CARCINOMA ON THE BASIS OF THE GROSS ANATOMY OF THE TUMOUR

A. SUGIOKA, T. TSUZUKI
Department of Surgery, Keio University School of Medicine, 35 Shinanomachi, Shinjukuku, Tokyo, Japan

Long-term survival of patients with hepatocellular carcinoma in now the issue. It was disclosed in our series that 5-year survival rate of patients undergoing potentially curative resection was 49% but 5-year disease-free survival rate dropped to 19%. In order to increase disease-free survival, it is necessary to elucidate predisposing factors to recurrence and to select sutiable treatment modalities. Univariate and multivariate analyses of disease-free survival of 137 patients, who underwent potentially curative resection between July 1973 and December 1990, revealed that gross anatomy of the tumour, tumour thrombus in the portal vein and/or intrahepatic metastases, tumour thrombus in the hepatic vein, and width of resected margin were significant factors. The gross antomy provides us with information not only on the boundary of tumours but also on the incidence of tumour thrombi. Consequently, we can know radicality of the surgery and necessity of adjuvant therapy immediately after surgery. It is anticipated that an improvement in disease-free survival is obtained by this approach.

### P206

### BACTERIOLOGICAL STUDY ON HUMAN LIVER TISSUE

YOSHIFUMI TAKENAKA [1], YOSHIYUKI SHIMAMURA [2]
1)Department of Surgery, Tokyo Dental College, 6-7-1, Sugano, Ichikawa-City, Ciba-Pref., Japan 2) Department of Surgery, National Matsudo Hospital, 123-1, Takatsuka-Shinden, Matsudo-City, Chiba-pref., Japan

It is generally known that bacteria are present in the tissues of mongrel canines and bulls. But, so far as we have searched, there is no report on the bacteria of the human liver tissue. We think it important to examine them for hepatic resection or transplantation. Then we examined them in the case of hepatic resection of 68 patients with hepatocellular carcinoma who visited National Matsudo Hospital from July, 1986 to March, 1988. Bacteria of the cancer-free area were cultivated immediately after laporatomy, and those of the cancer area were cultivated as soon as hepatic resection was over. Materials were entered to the anaerobic porter and cultured by anaerobic chamber method. Out of 14 cases 22 microorganisms were detected, and out of them 10 cases were PROPINIBACTERIUM ACNES which is anaerobic gram-positive coccus. Other microorganisms were shown on the list below. But 14 patients who provided 22 microorganisms, compared with others, had no special symptoms after operations. But P.acnes is known as the baterium causing heptic failure to mouse by joining with endotoxin. Then it is important to work out measures against P.acnes for extending the indication for hepatic resection or transplantation.

List of Bacteria detected from Liver Tissue of HCC Patients

| Detected Lesions | Cancer | Non Cancer |
|---|---|---|
| Propionibacterium acnes | 5 cases | 5 cases |
| Staphylococcus group | 3 | 2 |
| Streptococcus group | 1 | 1 |
| Acinetobactor lwoffii | 1 | |
| Enterococcus faecium | 1 | 1 |
| Torulopsis galabrata | 1 | 1 |
| Total | 12 cases | 10 cases |

**P207**

### ORTHOTOPIC LIVER TRANSPLANTATION FOR PRIMARY HEPATIC MALIGNANCY

V. VOUGAS, N.D. HEATON, R. MONDRAGON, J. O'GRADY, R. WILLIAMS, K.C. TAN
Liver Transplant Surgery, King's College Hospital, Camberwell. London SE5 9RS. United Kingdom

Orthotopic liver transplantation (OLT) has been used to treat patients with small primary liver tumour associated with cirrhosis, or with tumours in sites not amenable to resection by conventional surgery.

The early results of 23 patients (20 male: 3 female; mearn age of 50 years, age range 26-66 years) with parimary hepatic maligancy treated by OLT are presented. During the same period (January 1989 - November 1991) 250 OLT have been performed in this unit.

All 23 patients had cirrhosis (Child's grade A in 4, B in 7, and C in 12). Pre-existing liver pathology included hepatitis B in 6, alcoholic liver disease in 5, cryptogenic cirrhosis in 5, chronic active hepatitis in 3, NANB hepatitis in 2, primary sclerosing cholangitis in 1 and al-antitrypsin deficiency in 1. Tumour was localized to right lobe in 16 patients, the left lobe in one and 6 patients had bilateral involvement. The tumour size was less than 4 cm in 14 patients, 4-8 cm in 8 patients and one patient had a tumour of 10 cm. Histology showed hepatocellular carcinoma (HCC) in 19, mixed hepato-cholangiocellular in 3 and one patient had an HCC in the right and a cholangiocellular carcinoma (CHC) in the left lobe. Satellite lesions were present in 14 patients and evidence of vascular invasion was present in 10 patients.

Overall 6 patients have died. One patient died in the early postoperative period (hospital mortality 4.3%), 2 patients died from tumour recurrence at 5 and 6 months postoperatively. There have been three other deaths, two from recurrent hepatitis B related liver disease 3 and 8 months postoperatively and one from sepsis 9 months postoperatively. The overall mortality at mean follow up of 12 months (range 1-32 months) is 26%.

Liver transplantation has a role in the management of selected patients with primary hepatic malignancy and underlying cirrhosis. OLT is associated with a low perioperative mortality.

**P208**

### BILIARY RECONSTRUCTION IN ORTHOTOPIC LIVER TRANSPLANTATION (OLT)

V. VOUGAS, N.D. HEATON, R. MONDRAGON, J. O'GRADY, R. WILLIAMS, K.C. TAN.
Liver Transplant Surgery, King's College Hospital Camberwell, London. SE5 9RS. United Kingdom

Different techniques have been utilised in biliary truct reconstruction following (OLT). The incidence of biliary complications after OLT is approximately 15%. The standard approach has been to insert a T-tube into the recipient duct and leave it in situ for 6-12 weeks. However, the use of a T-tube is associated with a significant number of postoperative complciations. We recently used one with an internal silastic stent (IST) in place of the T-tube.

A retrospective study has been performed between a group of 28 patients with IST and the previous 30 patients with a conventinal T-tube (T-T) anastomosis. The mean age of patients in the IST group was 43 years (range 14-66 years) and 44 years (range 20-66 years) for the T-T group. 6 out of 30 patients in the T-T group and 4 out of 28 patients in the IST group. had fulminant hepatic failure. Complicatons were considered as early (<3 months post-OLT) and late (>3 months post-OLT). 4 patients from the IST group developed early complciations (1 leak, 1 stricture, 2 obstruction by bile sludge) which were treated surgically (2 patients having biliary reconstruction with a Roux-en-Y loop) or by endoscopic dilatation. One patient presented with a late stricture and was treated by endoscopic dilatation. In the T-T group 5 patients developed early complications (4 leaks, 1 stricture) treated by endoscopic stent insertion. 8 patients experienced late complications: 3 had biliary peritonitis, 2 had failed first attempt for tube removal and 3 had local pain and fever. Three patients had a laparotomy and 2 underwent biliary reconstruction iwth Roux-en-Y loop and all the other patients settled with conservative treatment which included antibiotic therapy.

Overall morbidity was 17.8% for the IST group and 43% for the T-T group. Overall mortality was 6.9% (T-T group-1, IST group -3) and was unrelated to the biliary complications. The use of T-tube was associated with a higher percentage complication rate. The IST group tended to develop early complication and the T-T group had more late complications

**P209**

### TEN YEARS EXPERIENCE OF PRIMARY LIVER CANCER IN NORTHERN ENGLAND

P.D. WRIGHT, J. COLLIER, M.F. BASSENDINE, O F W JAMES
Medical and Surgical Liver Units, Freeman Hospital, Newcastle upon Tyne, United Kingdom

Between 1981 and 1991 92 patients with primary liver cancer (P.L.C.) were seen in the Liver Unit, 68% were male and 32% female, and the mean age was 72 years. 96% were Caucasian and 91% from Northern England. 5% of patients were diagnosed on screening, the remainder presenting with symptoms of weakness (66%), pain (66%), anorexia (60%), weight loss (56%) or nausea (43%), with a mean duration of 7 weeks (range 1-78). At admission 83% had hepatomegaly and only 27% had previously documented cirrhosis, subsequently 60% had biopsy proven cirrhosis. Investigation showed an elevated alpha -FP in 86% with a level>500 ug/ml in 52%. $Hb^{s}Ag$ + we in 9%, and $HB^{c}AB$ + ve in 22%. CT scan showed mutiple lesions in 33% and a single lesion in 23%. 16% were offered no treatment, 16% had their tumours resected and 76% had chemotherapy alone or in combinatin with surgery. Median survival was 11.5 weeks (range 1-146 weeks). This data shows how the pattern of P.L.C. differs in a Northern European population from that seen elsewhere in the World.

**P210**

### REPEAT HEPATECTOMY: ITS CONTRIBUTION TO IMPROVEMENT OF PATIENT SURVIVAL IN RECURRENT HEPATOCELLULAR CARCINOMA

FUYO YOSHIMI, TAKESHI NAGAO, SUMIO INOUE, TOSHIYUKI SUMITA, HAJIMA SHIGA, MASAHIRO ISHIMARU, MOTOHIDE SODEYAMA, YOSHIMICHI OMORI, NOBUHIRO KAWANO, AND TETSUICHIRO MUTO
First Department of Surgery, University of Tokyo, Tokyo, Japan

The contribution of repeat hepatectomy to improvement of patient survival after initial hepatectomy for hepatocellular carcinoma (HCC) was investigated in 59 patients who underwent curative hepatectomy for primary HCC over the past ten years and developed intrahepatic tumor recurrence. Without repeat hepatectomy (48 patients), 1-, 3- and 5- year cumulative survival rates were 89.4%, 39.8% and 21.2% respectively. With repeat hepatectomy (11 patients, 14 repeat hepatectomies), these values were 100.0%, 81.8% and 63.6%. The cumulative survival curve of the repeat hepatectomy (+) group was significantly better than that of the repeat hepatectomy (-) group (p<0.004). The cumulative survival curve of the repeat hepatectomy group was better, although not significantly better, than that of the transcatheter arterial embolization group. There was one operative mortality in the repeat hepatectomy group.

**P211**

### TREATMENT OF LIVER CANCER USING A TOTALLY IMPLANTABLE DRUG DELIVERY SYSTEM

X.H. ZHANG, G.N. XU,
Institute of Hepatobiliary Surgery, Shanghai Hospital, Shanghai, R.R. China.

External catheter complications of hepatic artery infusion (HAI) sometimes preclude it from wide application. Recently a totally implantable drug delivery system (DOS) has been used to minimize those complicaitons. This article describes the construction of home-made DDS, properative preparation and operative technique. Since Dec. 1989, thirteen patients with liver cancer have been treated with the DDS. In ten patients with unresectable cancer (Therapeutic group), partial remission (PR) was present in 50% of cases, minor remission (MR) in 30% and no change (NC) in 20%. So the overall remission rate was 80%. In three patients with palliatively resected cancer (Pre-ventive group), no recurrence was found except one patient after 67 months of protal vein infusion. The median survival time in the series was 9 months with the range of 3 to 13 months. It is concluded that the home-made DDS has basically fulfiled qualitative requirments with a reasonable price and might be widely utilized.

**P212**

### LONG-TERM SURVIVAL AFTER SURGERY FOR HEPATOCELLULAR CARCINOMA

Z.D. ZHOU, Z.Y.TANG, Y.Q. YU, Z.C. MA, B.H. YANG, J.Z. LU, Z.Y. LIN, C.L. TANG, D.B. XU
Liver Cancer Institute, Shanghai Medical University, Shanghai, China

Long-term survival of patients with hepatocellular carcinoma (HCC) has rarely been encountered in the past several decades. Curutchet et al collected the data from 45 authors for 65 years (1905 - 1970) and found only forty-five 5-year survivors worldwide. We report 113 patients with pathologically proven HCC surviving over 5 years after surgery during the past 25 years (1961 - 1986), Of them, 104 patients underwent resection of HCC and nine patients underwent hepatic artery ligation (HAL) and/or infusion chemotherapy (HAI) for unresectable HCC. Subclinical stage amounted to 49.6% (56/113) and moderate stage to 50.4% (57/113). There were 55 aptients with small HCC (≤5cm). Cirrhosis was present in 81.4% (92/113). Radical resection was performed in 96.2% (100/104) . Reoperation for subclinical recurrence and solitary pulmonary metastasis was done in 23 patients, and second-look resection of huge tumors, in six patients. By end of May 1991, 37 of the 113 patients survived for more than 10 years; 16 patients, more than 15 years; and seven patients, more than 20 years. One patient has survived for 29 years and 8 months after resection of HCC. These results indicate that early detection and radical resection are the principal factors influencing long-term survival; reoperation for subclinical recurrence and solitary pulmonary metastasis remains an important approach to prolong survival further after radical resection; HAL and HAI may provide a hope for unresectable huge HCC.

**P213**

### SHORT-TEAM RESULTS OF RESECTION FOR HEPATOCELLULAR CARCINOMA: PREOPERATIVE COMBINATION THERAPY

1)H. SEKIDO, 1)A. NAKANO, 1)S. FUKAZAWA, 1)T. FUKUSHIMA, 1)Y. FUJII, 1)T. FUKUSHIMA, 2) K. TANAKA, 2)S. NAKAMUR
Second Department of Surgery and Third Department of Medicine, Yokohama City University, School of Medicine, Yokohama, Japan

Hepatocellular carcinoma (HCC) is one of most malignant tumour in Asia. Surgery is the only method potentially curative of HCC. However in Japan particularly a high incidence of cirrhosis associated with HCC has resulted in limitation of hepatic resection and even if carcinoma was completely resected, recurrent disease is not infrequent. Various therapeutic modalities including transcatheter arterial embolization (TAE) therapy and intramural percutaneous ethanol injection (PEIT) therapy has been developed. In present paper, we performed preoperative combination therapy of TAE and PEIT and show that these therapies are able to prevent a recurrence of HCC. (PATIENTS AND METHODS) For past 5 years, twenty five patients (18 men and 7 women) with HCC were operated in our department. The mean age was 55.2 years. Eleven patients were treated with TAE preoperatively. Six patients were treated with TEA and PEIT preoperatively Risk of posthepatectomy recurrence was evaluated with respect to various clinical parameters. Macroscopical and histological features were assessed. The presence of recurrent HCC was analyzed and disease free survival rates were 42% of patient treated with TEA, 75% of patient with TAE and PEIT. Histological findings showed complete necrosis of the tumour. After treatment of TAE and PETI, however, a few carcinoma cells remained.

CONCLUSION: After treatment of TAE and PEIT, postoperative disease free survived rate was markedly improved.

**P214**

### SIGNIFICANCE OF MEMBRANE FLUIDITY OF HEPATOCELLULAR CARCINOMA (HCC) ON PROGNOSTIC FACTORS

TAKAYUKI KASAMATSU, JUNJI TANAKA, WANG BEN SHANG, MITSUAKI KOHMOTO, MASANORI YOSHIDA, JUN TAMURA, KEN-ICHI FUJITA, SHIGEKI ARII
The First Department of Surgery, Kyoto University School of Medicine

Prognostic factors of HCC after hepatectomy have been well established in Japan. However, the mechanism of malignancy expression still remains unclear. On the other hand, the membrane fluidity has been understood to play an important role on cellular biological activity. The present study was aimed to clarify the possible effects of membrane fluidity on prognostic factors. Patients and Mehtods; The membrane fraction was obtained from 37 resected HCC cases by gradient centrifugation. Membrance fluidity was determined by measuring fluorescence polarization (P-value) with DPH dye. Results: P-values of normal livers were 0.189 $\pm$0.024(mean $\pm$ SD). In contrast, those of cirrhotic liver and HCC were 0.225$\pm$0.045, n=28, 0.221$\pm$0.024, n=37, respectively, $p<0.01$. P-values of non-cancer tissue of resected liver were 0.223$\pm$0.016. Portal tumor thrombosis (Vp) was found in 23 of 37 cases, of which P-values were 0.208$\pm$0.019 and significantly higher than negative group (0.234$\pm$0.017,$p<0.01$). When grouped by Edmondson's criteria, well differentiated HCC showed lower P-values than poorly differentiated groups, 0.211 vs 0.236, $p<0.05$. Also, AFP positive(>200 ng/ml) HCC showed higher P-values than negative group, 0.239 vs 0.214, $p<0.01$). Conclusions: Above results suggest that membrane fluidity of hepatocellular carcinoma may modify the malignancy expression.

### P215

### DNA PLOIDY AND CELL ACCUMULATION ON G2/M PHASE IN HEPATOCELLULAR CARCINOMA - IN RELATION TO TUMOR PROGRESSION AND PROGNOSIS

JUN TAMURA, JUNJI TANAKA, KENICHI FUJITA, MASANORI YOSHIDA, MITSUAKI KOHMOTO, TAKAYUKI KASAMATSU, WANG BEN ZHANG, LIU GANG, AND TAKAYOSHI TOBE.
The First Department of Surgery, Faculty of Medicine, Kyoto University, Kyoto, Japan.

The analysis of DNA ploidy patterns in various malignancies have been performed recently, for the purpose of detecting DNA aneuplodiy, which is significantly correlated to prognosis in some of the cases. On the other hand, there may be some other parameters as to cell kinetics of tumour cells in DNA histograms. On this viewpoint, the quantification of S phase fraction using various mathematical models has been developed, but it is not so generally accepted because of poor reliability. To evaluate the significance of DNA histograms as a parameter of malignancy in hepatocellular carcinoma (HCC), we analyzed the DNA ploidy patterns and the proportion of cells in G2/M phase, investigating the correlation to prognosis and other clinicopathological data (T, fc-inf, Edmondson's grade, AFP doubling time) in patients with HCC. Seventy-two cases of HCC at the 1st Department of Surgery, Kyoto University, from 1983 to 1986, which were available for flow cytometric analysis formed the materials of this study. DNA histograms of HCC were obtained from paraffin embedded tissues using flow cytometry. Among all cases, 43(59.7%) showed diploid cases, 29(40.3%) aneuploid. The coefficient of variation was 6.46% on average, the DNA index in aneuploid cases was 1.641±0.535(mean±S.D.). A significant correlation was observed between the proportion of G2/M phase cells and AFP doubling time($p<0.05$, $r=0.384$), indicating the accumulation of tumour cells on G2/M phase cells and AFP doubling time($p<0.05$, $r=0.384$), indicating the accumulation of tumour cells on G2/M phase suggests that the tumour is slow-growing. A significantly better prognosis was seen in diploid cases than aneuploid, that is, 5 year survival rate were 39.5% in diploid cases and 9.7% in aneuploid. In conclusion, there are some HCC cases of which the tumor cells are accumulated on G2/M phase in cell cycle, and the cell cycle may possible be delayed or blocked in such cases, judging from the AFP doubling time. On the other hand, DNA ploidy pattern has a significant correlation to prognosis in hepatocellular carcinoma.

### P216

### EVIDENCE FOR INTRA-HEPATIC PORTAL SHUNTS IN THE RAT LIVER

B. ALEXANDER*, V. JAFFE, PT MATHILE.
Department of Surgery, Royal Postgraduate Medical School, Du Cane Road, London, W12 ONN.
*Department of Surgery, King's College School of Medicine and Dentistry, Denmark Hill, London, SE5 9NU

Portal hypertension was induced in male Wistar rats (275-450g), by the injection of microspheres into the caecal vein via a 3 FG cannula (1.5mm i.d). Aliquots of up to $10^6$microspheres of 15, 25, 50 and 90µm diameter were injected. The results shown below are mean ±s.e., n=6, of portal venous pressures (PVP) before and after injection (inj), of microspheres. Post-injection PVP results were measured only when steady state conditions were achieved.

| diameter µM | Pre-inj PVP (mmHg) | Post-inj PVP I (mmHg) | ncrease in PVP (mmhg) |
|---|---|---|---|
| 15 | 10.1 ± 0.6 | 15.1 ± 0.6 | 5.0 ± 0.5 |
| 25 | 9.8 ± 1.0 | 13.9 ± 1.0 | 4.1 ± 1.1 |
| 50 | 9.3 ± 0.3 | 13.3 ± 0.5 | 4.0 ± 0.6 |
| 90 | 8.7 ± 0.5 | 13/4 ± 0.9 | 4.7 ± 0.7 |

Total occlusion of the main portal vein resulted in an average increase in PVP increase following injection of different sized microspheres and the general failure to induce a greater percentage than 23.2 ± 0.6% of the total PVP availabe (60 mmhg on occlusionof the portal vein) suggests the existence of intra-hepatic shunts > 90µm in diameter. It is proposed that under 'normal' conditions these become patent during acute episodes of increased portal venous pressure and perhaps cease to function during pathological conditions.

### P217

### THE PURINERGIC REGULATION OF HEPATIC ARTERIAL VASCULAR TONE

B. ALEXANDER, PT MATHIE[1], V. RALEVIC[2], G BURNSTOCK[2].
Dept of Surgery, King's College, London, UK.
[1]Dept of Surgery, Royal Postgraduate Medical School, London, UK.
[2] Dept of Anatomy, University College, London, UK.

Patient prognosis may be directly related to the magnitude of the hyperaemic response of the hepatic artery (the buffer response) following portal flow deprivation. Pharmacological enhancement of this response could offer improved patient prognosis during periods of hepatic portal deprivation. In vivo observations in dogs with mesocaval shunts suggested some purinergic ($A_2$) regulation of the buffer response. A series of in vitro isolated dual-perfused rabbit perfusions were conducted to characterise the nature of the purinoceptors present in the rabbit hepatic arterial vasculature. At normal tone, dose-related increases in vasoconstriction, with maximal increases in perfusionpressure of 8.7 ± 1.3 mmHg were demonstrated to ATP. At raised tone (achieved by addition of $10^{-5}$M noradrenaline to the perfusate) dose-related vasodilatations to adenosine and ATP were demonstrated, with maximal decreases in perfusion pressure of 16.5 ± 2.8 mmHg and 31.3 ± 8.7 mmHg respectively. The $A_2$-purinoceptor antagonist, 8-phenyltheophylline, and the nitric oxide inhibitor, methylene blue, reduced these values to 6.8 ± 0.6 mmhg and 10.5 ± 2.2 mmHg for adenosine and ATP respectively. Finally, these inhibitors confirmed the hyperaemic signficance of adenosine and ATP purinoceptors in the regulation of hepatic arterial vascular tone.

### P218

### PORTAL VENOUS PRESSURE CHANGE BY NONSHUNTING OPERATION AND ITS INFLUENCE ON PROGNOSIS OF PATIENTS WITH LIVER CIRRHOSIS

YASUSHI HARIHARA, HIROAKIIMANISHI, HIROSHI IMAMURA, KAZUMASA OHASHI, YASUTSUGU BANDAI, KENSHO SANJO, YASUO IDEZUKI,
Second Department of Surgery, University of Tokyo, Tokyo, Japan.

Nonshunting operation for Oesophageal varices has developed as the procedure in which the portal venous blood flow to the liver can be maintained as compared with ordinary shunting operation. However, the portal venous pressure (PVP) after the nonshunting operation was not always the same as that before the operation. The purpose of this study is to elucidate the influence of PVP change by nonshunting operation on the prognosis of patients with liver cirrhosis. Seventy-nine patients with esophageal varices and liver cirrhosis who underwent the nonshunting operation (University of Tokyo method, consisting of esophageal transection, devascularization and splenectomy) and intraoperative PVP measurement berfore and after the procedure at our department from 1975 to 1982 were studied. Sixty-two were male and seventeen were female, with the mean age of 50.2. Actuarial survival rates were compared among the groups divided according to PVP changes by the procedure. The intraoperative PVP before and after the procedure was 349±65 mm saline (180-480) and 317±59 mm saline (200-480), respectively, Significant decrease in PVP was observed after the procedure by the mean value of 32 mm saline (9.2%) ($p<0.01$). Actuarial survival rates were significantly low in the groups in which PVP decreased by more than 125 mm saline (n=3) or at the rate of more than 25% (n=6) (both $p<0.05$). In conclusion, these results suggest that total liver blood flow after the nonshunting operation can be compensated by the increase of arterial flow in patients with liver cirrhosis, unless PVP was extremely reduced by the operation.

**P219**

### REVIVAL OF SHUNT CONCEPT IN BLEEDING VARICES - A PROSPECTIVE STUDY

A MULLER, A.HIRNER, P. DECKER
Bonn University, Surgical Department, Sigmund-Freud-Strasse 25, D-5300 Bonn 1, Germany

Our theory: The most valuable acute treatment of variceal bleeding is emergency sclerotherapy (rate of hemostasis in Bonn 94%: 1980 - 1990, ~ 700 patients). For prophylaxis of further bleeding, the porta-systemic shunt is having a revival because of the disadvantages of long-term-sclerotherapy: complication rate in Bonn 21%, and rate of rebleeding 41%: 1980 - 1990, ~ 1000 patients. These data are corresponding to the literature.

Our concept (planned prospectively): Emergency endoscopy, if possible immediate sclerotherapy, and angiography. Indications for early shunt operation: major initial bleeding (>4 blood units), early rebleeding in spite of sclerotherapy, bleeding of fundic varices, and hypertensive gastropathy. We prefer 2 different types of shunt: portacaval end-to-side shunt (PCA: 60%) and distal splenorenal shunt (Warren: 40%). Indications for PCA are massive bleeding, small splenic vein, and low portal liver perfusion.

Our results: From II 1989 to VIII 1991 87 patients were admitted in the study protocol. 62% underwent surgery for shunting, 38% had long-term-sclerotherapy, respectively devascularisation procedure (Sugiura). The survival rate of all shunt patients after 30 months was 65%, including the initial hospital mortality rate of 23%. Differentiated according to the initial Child-classification, patients with Child A have better long-term-survival rate of 85% than those in B and C with 46%: the difference is caused only by a higher initial hospital mortality rate. According to a study concerning life quality 88% of all operated patients are satisfied with the result; 57% are able to continue their former jobs. The out-patient follow-up-examinations showed: 15% (frequently only episodal) encephalopathy, 29% (partially progressive) liver insufficiency.

**P220**

### A PROSPECTIVE RANDOMISED CONTROLLED CLINICAL TRIAL COMPARING SANDOSTATON (SMS) AND INJECTION SCLEROTHERPY IN THE CONTROL OF ACUTE VARICEAL HAEMORRHAGE: AN INTERIM REPORT.

S A JENKINS,G COPELAND, A KINGSNORTH & R SHIELDS
UNIVERSITY OF LIVERPOOL DEPT. OF SURGERY, LIVERPOOL L69 3 BX.

Recent studies have indicated that somatostatin is a safe effective treatment for the control of acute variceal haemorrhage (AVH). Since SMS a synthetic analogue of somatostatin is relatively inexpensive compared to the naturally occuring hormone, the aim of this study was to compare the efficacy of SMS with injection sclerotherapy (IS) in the control of AVH. Forty consecutive patients admitted with endoscopically proven severe AVH were randomised to either IS or a continuous infusion of SMS (50)ug/h) for 48 hours. The aetiology of the portal hypertension was similar in the two groups as was the distribution of the patients among the categories of the Child's classification. Twenty patients received SMS twenty IS. Overall contro of bleeding was achieved in 18/20 patients randomised to IS (p = 0.70 Fischer's Exact Test ). SMS was equally effective as Is in controlling AVH in patients with mild, moderate and severe disease and those actively bleeding at the time of their diagnostic endoscopy. Mortality was not significantly different between the two groups of patients. The results of this trial suggest that SMS is: 1) a safe and effective stop-gap treatment for the control of AVH and 2) may be as efficacious as IS although at this stage a type 2 error cannot be excluded.

**P221**

### RENAL IMPAIRMENT FOLLOWING INTRAVENOUS INJECTION OF ETHANOLAMINE OLEATE (EO)

S A JENKIN, H KYNASTON, M O'DRISCOLL, J YATES, K F PARSONS AND B A TAYLOR
Departments of Surgery and Nuclear Medicine, Royal Liverpool Hospital, Liverpool UK.

It is becoming clear that injection sclerotheapy (IS) of oesophageal varices (OV) can result in systemic complications. the aim of this study was to investigate the relationship between platelet aggregation and renal blood flow (RBF) and function (FR) after administration of EO to rats and in patients after IS. Organ blood flow (microsphere technique) was measured 1h and 24h after i.v. administration of EO(0.5ml/100g) to portal hypertensive rats, and RBF and RF determined by plasma clearance of Mag 3, a renal tubular agent. Mag 3 renography and platelet counts were also measured in cirrhotic patients before and after IS. In rats intravenus EO resulted in a rapid fall in the platelet count (822+36x10/1;mean±SEM)which was associated with microscopic or macroscopic haematuia. RBF was unchanged after 1 or 24h. RF was significantly impaired 24h (after EO administraton) (o.65±0.08ml/min100g;mean±SEM) compared to controls (1.67±0.19ml/min/100g;mean±SEM). Similar but less dramatic changes were observed in patients after IS, haematuria (10-20%) and alterations in renal function in the majority.However the very low platelet counts in these patients is a confounding factor. These observations suggest that hepatic and renal necrosis and impairment of RFafter administration of EO are not associated with alterations in RBF but may be secondary to platelet microembolisation.

**P222**

### FREE OESOPHAGEAL PERFORATION AFTER FIBREOPTIC ENDOSCOPIC VARICEAL SCLEROTHERAPY IN PORTAL HYPERTENSION

JEJ Krige, PA Goldberg, PC Bornman, J Terblanche. MRC Liver Research Centre, and Dept of Surgery, University of Cape Town and Groote Schuur Hospital

This analysis sought to establish the incidence and risk factors for oesophageal perforation after injection sclerotherapy (IST) for oesophageal varices (OV).

231 consecutive patients (mean age 49.3 yrs [147 males]) with OV were studied prospectively from 1984 to 1990 after IST using ethanolamine oleate with an intra- and paravariceal technique. 1138 IST sessions during 2037 fibreiptic endoscopies were complicated by 5 free oesophageal perforations resulting in death in 4 patients. The incidence of perforation was 2.16% of patients or 0.44% of variceal injection sessions. All injections in the perforated group were performed between 7 and 25 days of the index bleed. For comparision, analysis in both groups only considered the first 25 days.

| | Perforation | | P |
|---|---|---|---|
| | No | Yes | |
| Number patients | 226 | 5 | |
| Child's A | 29 | 0 | |
| Grade B | 100 | 3 | n.s. |
| C | 97 | 2 | |
| Injection n mean (SD) | 2.5 (1.8) | 2.6(.9) | n.s. |
| Total vol injected (SD) | 36.5 (21) | 44 (25) | n.s. |
| Vol per injection (SD) | 13.7 (5.4) | 15.4 (7) | n.s. |
| Sengstaken used occasions | 81 | 3 | n.s. |
| inpatients | 57 | 1 | |
| not used in patients | 169 | 4 | |

Conclusions: The incidence of perforation is low after fibreoptic IST and the risk of perforation is greatest early after the acute index bleed.Technical and operator dependant factors rather than amount of sclerosant may be responsible for oesophageal perforation after IST.

### P223

### OESOPHAGEAL STRICTURE FOLLOWING VARICEAL SCLEROTHERAPY IN PORTAL HYPERTENSION.

JEJ KRIGE, PA GOLDBERG, R ARRELL, PC ORNMAN & J TERBLANCHE.
MRC Liver Research Centre, Surgical Gastroenterology, Groote Schuur Hospital and Department of Surgery, University of Cape Town.

Injection sclerotherapy (IST) is established treatment for bleeding oesophageal varices, but may cause oesophageal stricture (OS). This study sought to determine the incidence of OS and identify possible predisposing factors and response to therapy.

Patients and Method: 205 patients received IST on 1022 occasions at 1860 visits using a combination of intra and para-variceal techniques with ethanolamine oleate via flexible endoscopy in a prospective study between 1985 and 1990 and were followed up until September 1991. Varices were graded 1 to 5 at endoscopy.

Results: 23 patients (11%) developed OS after a mean interval of 6.5 month (range 1 to 35 months). 9 did not require oesophageal dilatation and 14 required a mean of 4 dilatations (range 1-8). No. patient who developed stenosis bled from the oesophagus after obliteration of varices. There was no difference in the 2 groups of patients in the number of times they bled from the gastro intestinal tract both before and after obliteration of varices.

| | No Stricture | Stricture | P |
|---|---|---|---|
| number | 158 | 23 | |
| initial grade of varices | 4 | 4 | n.s. |
| slough* | 128 | 22 | n.s. |
| number of IST | $4 \pm 3$ | $6 \pm 4$ | <.001 |
| Total vol (ml) | $58 \pm 38$ | $84 \pm 47$ | <.004 |
| Vol per inj | $13.4 \pm 4.9$ | $11.7 \pm 3.8$ | n.s. |

number of parients with slough after IST.

**Conclusion:** Oesophageal stricture is more likely to occur in patients who receive repeated IST and large cumulative volumes of sclerosant. Stenosis is usually easily managed with dilatation.

### P224

### THE EVALUATION OF THORACIC DUCT LYMPH DECOMPRESSION IN EXPERIMENTALLY PRODUCED ASCITES IN DOGS

F.LUKIC, V. SIMCIC, M.SNOJ.
The Institute of Oncology, Zaloska 2, 61105 Ljubljana, Slovenia.

The diameter of the supradiaphragmal part of the vena cava was reduced by polyethylene wrapping for about 75% through right thoracotomy in endotracheal anesthesia. The animals developed ascites 3-4 weeks following surgery. The ascites persisted for 4-6 months, and disapperared spontaneously later on. The animals were normal , excpet for their swollen abdomens. The protein level of such ascitic fluid from 0.3 to 4.7%. At the time of surgery the pressure in the place of vena cava stenosis was 15.5 - 20 cm of water (average 17.8 cm) and above the stenosis 4 -5.5 cm of water (average 4.5 cm). The pressure of the thoracic duct lymph 3 weeks after surgery was 20 -40 cm of water (average 31 cm), and the pressure in the vena cava was 17-20 cm of water ( average 17.8 cm). After 8 weeks the pressure in the thoracic duct was 2-10 cm of water (average 5.9 cm) and in the vena cava 21-25 cm of water (average 22 cm). Ascites depletion by punction normalised the pressure in the vena cava and thoracic duct in a few hours.

Three weeks after the appearance of ascities, thoracic duct to jugular vein anastomosis was performed for decompression of the thoracic duct lymph flow. Ascites disappeared 3-4 weeks later. The thoracic duct lymph flow represents a by-pass for portal hypertension in the first 8-10 weeks; later on the pressure in the thoracic duct becomes normal, which renders the by-pass questionable.

### P225

### SCHISTOSOMAL VARICEAL BLEEDING IN BLACK SOUTH AFRICANS: $Tc^{99m}$ DTPA HEPATIC PERFUSION INDEX AND GALACTOSE ELIMINATION CAPACITY

MCM MODIBA, D PANTANOWITZ, J ESSER, I SEGAL, J A MYBURGH
Department of Surgery, Medicine, Nuclear Medicine, University of the Witwatersrand

Forty-four patients were treated for schistosomal variceal bleeding at Baragwanath Hospital over 3 years.

HYPOTHESIS: In schistosomiasis the hepatic artery is encased in a periportal fibrosis, this may limit hepatic arterial compensation for portal venous flow loss after distal splenorenal shunting (DSRS).

METHODS: Galactose elimination capacity (GEC) and $Tc^{99m}$ DTPA hepatic perfusion index were measured in schistosomiasis patients requiring treatment of bleeding oesophageal varices. Arterial supply was estimated by measuring $T_1$ (1).

RESULTS: $T_1$ was $0.17 \pm 0.08$ in 13 patients with schistosomiasis as compared to $0.33 \pm 0.16$ in 13 patients with portal vein thrombosis (Mann Whitney p=0.005). In 8 patients with Hep B Sag +ive chronic active hepatitis associated with schistosmiasis $T_1$ increased to $0.36 \pm 0.13$ at 10 days (Wilcoxon p=0.01) but fell to $0.22 \pm 0.15$ at 6 to 12 months (NS). GEC was $338 \pm 50$mg/min in schistomiasis and $298 \pm 38$ in portal vein thrombosis (p=0.12).

CONCLUSION: In schistosomiasis the hepatic arterial response to DSRS is impaired.

Reference: J A Myburgh. Selective shunts: the Johannesburg experience. Am J Surg 1990; 160: 67 - 74

### P226

### SHUNTS FOR PORTAL HYPERTENSION: POSTOPERATIVE ASSESSMENT WITH COLOR DOPPLER SONOGRAPHY (CDS) AND MR-ANGIOGRAPHY (MRA)

A. MULLER, A. STEUDEL, C. REICHEL, A. HIRNER
Dept. of Surgery and Dept. of Radiology, University Medical School Bonn, Germany

Two noninvasive imaging modalities were used for perform shunt patency of 42 partocaval (PCA) or splenorenal (Warren) shunts. Angiography only evaluated cases that showed different results of CDS and MRA.

38 patients with liver cirrhosis were studied after shunt surgery. Colour Doppler Sonography (CDS) were performed using standard techniques of vessels insonation and commercially available equipement (Quantum Angiodynography (MRA) was performed on a 1.5T, Philips) using the 2 D-inflow gradient echo sequence (FFE) with 50/15/60 (TR/TE/rf pulse angle). All CDS and MRA images were assessed for shunt patency and flow direction in the main portal vein.

Shunt patency was proven considered in all but one patient in the synopsis of both imaging modalities. MRA showed shunt stenosis in another patient which was confirmed by angiography and shunt revision. All MRA were of diagnostic sufficient quality. In 18% of CDS studies shunts were not sufficiently available.

CDS and MRA give reliable and accurate information to determine shunt patency. Angiography is only necessary in special cases.

**P227**

### LONG TERM FOLLOW-UP OF PORTO SYSTEMIC SHUNTS

A. MÜLLER, A. HIRNER, P. BLAUERT
Department of Surgery, University Medical School

Porto systemic shunts as well as sclerotherapy play a major role in the treatment of esophageal varices hemorrhage in portal hypertension of patients with liver cirrhosis. Especially in emergency situations shunt operations can lead to a definitive hemostasis. Main points of discussion are the disadvantages like hepatic encephalopathy of the portocaval shunt.

87 patients were treated with acute bleeding at our department during the last 2 1/2 years, 54 patients (62%) underwent a porto systemic shunt. The hospital mortality rate was 22% (12 pat.), 7 patients died during the follow-up.

The follow-up included 35 patients who were investigated for recurrent bleeding, liver function, shunt patency and flow direction.

A focus of interest was the hepatic encephalopathy especially in patients with portocaval shunt.

Encephalopathy was found in 15% of our patients, liver insufficiency of minor degree in 29%. More than half of these patients were working in their former profession, 88% lived a normal life. Two third of the alcoholics were abstinent in the postoperative period.

Our results show that the disadvantages of porto systemic shunts can prevented by a consistent outpatient management.

**P228**

### LONG TERM RESULTS OF DISTAL SPLENORENAL SHUNT

A. PIETRABISSA, A. DI VITO, P.C. GIULIANOTTI, F. D'ELIA, E.SCARCELLO, M. FERRARI, M. OLEGGINI, F. MOSCA
Istiuto di Chirurgia Generale e Sperimentable, Universita di Pisa, Italy

The place of distal splenorenal shunt (DSRS) in the management of portal hypertension is controversial. Data on 65 patients subjected to this procedure was collected over a 12-year period. A standard DSRS was carried out in 54 cases, while hemodynamically equivalent shunts were performed on 11 occasions. Eighty four per cent of patients were Child A; 16% B or C Alcoholic cirrhosis was the cause of portal hypertension in 15% 5 patients DSRS was done on emergency: Operative mortality was 4.6% Rebleeding after surgery occurred in 6.7%. Fifty six patients operated on before 1987 were elegible for 5-year follow up analysis with a mean follow up time of 82 months. Overall 1-to 5- years survival rates were 93%, 89%, 84%, 73%, and 70% respectively. Some degree of post shunt encephalophaty was detectable in 19% of patients. Portal and splenic vein hemodynamics were studied by Duplex sonography in 23 patients at an average of 88 months post shunt. A reduction of the diameter of the portal vein as compared to early postoperative values was detected in all cases and was significantly narrower in those with alcoholic cirrhosis. Portal vein thrombosis occurred in 7 The mean blood flow was 307 ml/min in the protal vein and 825 ml/min in the splnic vein. In conclusion this study confirms that the long term results of DSRS are strongly affected by the evolution of underlying disease and by the loss of shunt selectivity over the time. Our approach to selection of patients for DSRS is currently done taking into account the reported good results of injection sclerotherapy, which remains our first choice, particularly in emergency. Our indications to DSRS are failure of sclerotherapy, varices of the gastric fundus and patients unsuitable to chronic sclerotherapy management.

**P229**

### ULTRASTRUCTURAL ALTERATIONS OF GASTRIC MUCOSA IN PORTAL HYPERTENSIVE RATS, AFTER USE OF TETRANDRINE, SALVIA MILTIORRHIZAE AND PROPRANOLOL

SHEN YAO ZONG, MU YI, HAN BIN
Research laboratory of hepato-biliary surgery
Xuzhou Medical College, Jiangsu, China

The ultrastructure of gastric mucosa was observed by transmission electron microscope in rats with experimental portal hypertension (PHT) and those treated by tetrandrine, salvia miltiorrhizae and propranolol. In addition, a comparative study with normal and liver cirrhosis with PHT control group had been made. In PHT rats, capillaries in gastric mucosa were remarkably dilated, cytoplasm of endothlial cells were very thin, endothelial cells damaged and blood plasma in caplillaries leaked out; blood in capillaries was stagnated; the interspace between capillaries and gland cells transformed, organelles, secretion decreased; cells joint was destroyed to become an edema cavity. The secretory function of partietal cells decreased, organelles dissolved to form an empty cavity. In all treated rats with PHT, the above-mentioned pathologic alterations disappeared, the ultrastructure of gastric mucosa appeared to be normal. These results proved that the destruction of microcirculation in gastric mucosa is the foundation of gastric mucosal lesions (GML) and the tetrandrine, salvis miltiorrhizae and propranolol are effective in treatment for GML.

**P230**

### FACTORS AFFECTING INCIDENCE OF ENCEPHALOPATHY POSTPORTACAVAL SHUNT

K. R. SIRINEK AND B.A. KEVINE,
The University of Texas Health Science Centre, San Antonio, TX, U.S.A.

Proponents of selcetive and small diameter (8mm), interposition-graft portosystemic shunts maintain that encephalopathy post-total portosystemic shunt is secondary to loss of hepatic portal flow across a low pressure gradient anastomosis. This study assessed incidence of post-op encephalopathy in 72 patients undergoing a side-to-side portacaval anastomosis (25mm) who had had portal hemodynamic stuidies pre-op, intra-op, and post-op.\Sixteen patients (22%) developed clinically-evident post-op encephalopathy: 9/16 were not encephalopathic pre-op, 6/16 occurred early (<30 days), 10/16 were late (sepsis 4, dietary indiscretion 3, multipe and incapacitating 3), and 4/16 died post-op. There was no differnce in measured portal hemodynamic parameters in these 16 patients compared to 56 patients without encephalopathy. Encephalopathic patients had worse hepatic function (Childs' class C 70% vs 46%) and higher incidence of emergency shunts (38% vs 9%). Encephalopathy occurs post large diameter portacaval anastomosis as a result of failure to acutely control bleeeding in patients with poor hepatic reserve and is not secondary to the size of the anastomosis or the post-shunt anastomotic pressure gradient. A large diameter (25mm) side-to-side portacaval shunt with an 8% mortality, a 22% encephalopathy rate (new 12%, incapacitating 4%), and no recurrent variceal bleeding remains the "gold standard" for the treatment of variceal hemorrhage in patients with alcoholic cirrhosis.

**P231**

### THE FIBRINOLYTIC ACTIVITY OF ALCOHOL-INDUCED ASCITES

DM SCOTT-COOMBES, SA WHAWELL, JN THOMPSON
Department of Surgery, Hammersmith Hospital, Du Cane Road, London, England

The relative contributions of primary fibrinolysis and disseminated intravascular coagulation towards the aetiology of coagulopathy following insertion of a peritoneo-venous shunt remains controversial. The aim of this study was to measure the overall fibrinolytic activity and individual fibrinolytic system mediators in alcohol-induced ascites.

Samples of ascites were aspirated from ten patients (9 male, 1 female; age range 37-62 years) who had chronic alcoholic liver disease.. The concentration of tissue plasminogen activator (t-PA) was a median 28ng/ml (range 13-99) and the concentration of plasminogen activator inhibitor-1 (PAI-1), the potent inhibitor of t-PA, was 17ng/ml (<2-43). All the samples posessed plasminogen activator activity (median 0.9IU/ml, rnage 0.3-1.5) and this correlated with the excess of t-PA (in molar terms) over PAI-1 (r=0.71, P<0.05).

These results demonstrate that alcoholic ascites posesses substantial fibrinolytic activity and that its magnitude is largely determined by the balance of t-PA and PAI-1. This fibrinolytic activity could be responsible for a coagulopathy following peritoneovenous shunting mediated by primary fibrinolysis. This activity may also contribute to the intraperitoneal haemorrhagic complications that are encountered following procedures such as percutaneous liver biopsy and laparotomy.

**P232**

### THE LIVER MICROCIRCULATION IN PORTAL HYPERTENSION

J.D. WILLIAMS, P. MACK
Department of Surgery, Singapore General Hospital, Singapore

Using the technique of in vivo microscopy for qualitative study, striking changes were noted in the dynamic microcirculation in the livers of cirrhotic rats. After carbon tetrachloride-induced hepatic cirrhosis, the appearance of the hepatic microvasculature was noted to have undergone distinct morphological changes when compared to normal controls. The hepatic and portal venules assumed a crooked and meandering appearance, and the blood flow was decreased in many. There was less branching of the vessels and fewer number of vessels per microscopic field when compared with normal rat livers. Since portal hypertension is present in both cirrhosis and biliary obstruction, rats with common bile duct ligation were also studied with the intravital microscope. In both conditions, the microvasculature assumed a similar bizarre tortuous appearance. Cirrhosis and biliary obstruction of varying durations were studied under in vivo microscopy and correlated with liver function tests and histology. We conclude that the morphological derangement in the the hepatic microcirculation in cirrhosis and biliary obstruction is similar in appearance and may be related to portal hypertension.

**P233**

### ENDOSCOPIC SCLEROSIS FOR BLEEDING ESOPHAGEAL VARICES

XU MAN-YUAN
Hospital of Xin Zhou Prefecture, ShanXi, China

Hemorrhage from esophageal varices is one of the major complications in cirrhotic paients with portal hypertension, which associates with a high mortality. For many years, the treatment has consisted of Ballon tamponade and/or the use parenteral vasopressin infusion. But the results of this therapy have been consistly unsatisfactory. In order to evaluate sclerotherapy, since 1982, we have treated 102 cases of acute hemorrhage from esophageal varices with endoscopic sclerotherapy. All the patients underwent emergency fiberoptic endoscopic sclerotherapy with Olympus Giftypek fiberogastroscopy. A self-made 5 gauge injector needle of 3mm long was passed through the channel of the scopy, and about 2 mm of 5% sodium morrhuate was injected into paravariceal submucous tissue. Several varices were injected on different plane at each procedure. The total dose was below 30ml. Sclerotherapy was repeated 7 days later. All the patients have been followed for one year. By using self-made 5 gauge injector needle of 3mm long, we have prevented esophageal wall from necrosis and perforation due to injection of sodium morrhuate into mascular layer. Also by injection of sodium morrhuate on different plane of varices, we have prevented esophagus from stricture. The results of our study are shown in table one. It demonstrates that sclerotherapy for bleeding esophageal varices is effective and safe.

Table 1 Morbidity and mortality after sclerotherapy

| | Child A | Child B | Child C | Total |
|---|---|---|---|---|
| No. | 16 | 32 | 54 | 102 |
| Age, Yr. | 43 | 48.5 | 53.5 | 50.1 |
| M/F | 14/2 | 27/5 | 47/7 | 98/14 |
| Rebleed | 1(6.2%) | 3(9.3%) | 14(25.9%) | 18(17.1%) |
| Mortality | 1(6.2%) | 4(12.5%) | 17(31.5%) | 22(21.5%) |

**P234**

### ASSOCIATION OF PREOPERATIVE ACUTE RENAL FAILURE AND FIBROSIS ON LIVER BIOPSIES IN BUDD-CHIARI SYNDROME (BCS) A SEVERE PROGNOSIS FACTOR IN SHUNTED PATIENTS

G. ZEITOUN, L. ESTEVES-LIMA, M.J. BOUDET, J.M.HAY, G, PARMENTIE, A, KATZ, J.COHEN-SOLAL, V. LABORIE
Hospital Louis Mourier, Paris, France

The standard surgical treatment of BCS is still a shunt procedure between the portal and the caval system. Depending on whether or not the inferior vena cava (IVC) is obstructed, the presence of a negative or weakly positive porto-IVC gradient precludes a shunt bypass toward the IVC system. In that feature, a shunt bypass toward the superior vena cava (SVC) system is needed. In our experience, meso artrial shunt, that is an intrapericardial shunt (IPS) can lead to severe pericardial complications. This prompted us to design extrapericardial shunts (EPS) avoiding opening the pericardium.

From 1.1973 to 6.1991, we performed 69 shunts on 60 patients. In 36 cases (33 pts) the portal flow was shunted toward the IVC. In 33 cases (27 pts), the portal flow was shunted toward the SVC using 17 IPS and 16 EPS. All pts had pre and intra-operative biopsies and in most of them a liver biopsy (LB) was performed on the left, the right and the caudate lobes. LB were classified as follows: centrilobular necrosis (CLN) alone, CLN and severe fibrosis (F), F alone. There were: 16 CLN, 26 CLN + F, 27 F. On laboratory studies, 12 pts had a preoperative acute renal failure (POARF) defined by a blood creatinine level > 120 umol/l. Of these 12 pts , 9 had F.

Results : The overalll mortality was 29%: 9 out of the 12 pts with POARF died (75%); 8 of these 9 patients had F on LB (90%); 1 patient over 9 with POARF and F survived (10%). The 2 pts with POARF and no F survived. Statistical analysis showed the following results: POARF vs no POARF (p<001); F and POARF vs F and no POARF (p<001).

Conclusion: 1) Shunt is indicated in patients with BCS when patient has no POARF or POARF but no Fibrosis on liver biopsies. 2) Shunt is not indicated in patients with POARF and Fibrosis on liver biopsies.

P235

### AN EXPERIMENTAL STUDY OF E. COLI INDUCED IDIOPATHIC PORTAL HYPERTENSION (IPH) IN RABBITS

YUQUAN TAN AND MINJIE ZHANG
Surgery Department, No. 1 College of Clinical Medicine, Norman Bethune University of Medical Sciences. Changchun, P.R..China

Animal models of IPH were performed in 23 rabbits by injection of E. Coli suspension through portal vein. The animals were divided into 4 groups: Group I, E. Coli suspension. Group II, mixture of E. Coli and rabbit anti-serum.. Control I, saline. Control II, rabbit anti-serum were injected every 3 wks, 3 times. The animals were killed 1 month after the last injection. In comparison to the control group, animals of group I and group II gave a picture of portal hypertension both clinically and hemodynamically. We believe intraperitoneal infection is one of the etiological factors of IPH, which induce inflammatory reaction, cellular infiltration and fibrosis of the portal triad, which finally results in the obliteration of intrahepatic terminal portal branches. So actually it's a type of presinusoid portal hypertension like that due to schistosomiasis.

P236

### ENDOCRINE TUMORS OF THE PANCREAS AND PERIAMPULLARY AREA

R. BELLANTONE, G.B. DOGLIETTO, D. FRONTERA, A. FERRANTE, G. VIOLA, F. CRUCITTI
Department of Surgery, Catholic University School of Medicine, Rome, Italy

We observed 7 cases of neuroendocrine tumors out of 220 pancreatic and periampullary neoplasms admitted from 1981 to 1991. There were 4 pancreatic insulinomas (head:1, body-tail:3), 2 carcinoids (ampulla:1, pancreatic tail:1) and 1 duodenal somatostatinoma. The patients' average age was 53 yrs. (range 30 - 64). Only insulinomas were clinically functioning and these patients underwent specific endocrine evaluation (positive results in 3 cases). Site diagnosis of Insulinomas was obtained with ETG in 1 case, with CT scan in another; angiography was useful in 2 patients. Three pts. had a preoperative biopsy (echo-assisted FNB or endoscopic biospsy) but only in 1 case the histology showed neuroendocrine neoplasia.

Surgical treatment was performed in all patients as follows: 4 distal pancreatectomies (3 insulinoma, 1 carcinoid) with concomitant left nephrectomy and colic resection in one case because of local diffusion; 1 enucleation (insulinoma), 1 Whipple procedure (carcinoid), 1 explorative laparotomy (duodenal somatostatinoma with unexpected liver metastases), Operative mortality was 0%. We observed a pancreatic fistula which healed spontaneously following distal pancreatectomy.

The 4 patients operated for insulinoma are alive and disease free more than 24 months postoperatively. The patient submitted to distal pancreatectomy for carcinoid is alive 42 months after surgery; she had CT scan evidence of hepatic metastases but refused further surgery. The patient with ampullary carcinoid (Whipple) developed hepatic and diffuse lymphnidal metastases 4 months postoperatively. The patient explored for duodenal somatostatinoma had 7 cycles of chemotherapy (5-FU) with partial response; she is alive and in good conditions 9 months after the laparotomy.

We think surgery is the only effective therapy for neuroendocrine tumors, especially in benign and functioning types. Resection seems justified also in malignant tumors, although with a cytoreductive aim only, in order to improve the effects of adjuvant therapies and relieve symptoms associated with functioning tumors.

P237

### CYSTIC NEOPLASMS OF THE PANCREAS

G. BELLI, G.ROMANO, A.MONACO, A. D'AGOSTINO and M.L. SANTANGELO
Istituto Chirurgia Generale e Trapianti II Faculty
University of Naples, Naples, Italy

Cystoadenomas and cystoadenocarcinomas are rare but not exceptional tumours of the pancreas. The serous cystoadenoma is benign, the mucinous cystoadenoma has to be considered as potentially malignant. The cystoadenocarcinoma is malignant by definition. At our Institution between 1984 and 1990, 20 patients with cystic pancreatic lesions were treated. Of these 15 were pseudocysts and 5 tumours. The location of cystic neoplasms was : tail 3, head 2. Three distal pancreasectomy and two cephaloduodenopancreasectomy were performed including one patient with previous internal drainage for a misdiagnosed pseudocyst. One pancreatic fistula and one epigastric collection were observed, both treated conservatively. Histologically there were two serous cystoadenomas, two cystoadenocarcinomas and one mucinous cystic neoplasm. The resection of benign lesions is justified for malignant degeneration cannot be ruled out by CYST biopsy. Therefore, we strongly advise any cystic neoplasm be totally excised even as prognosis of cystoadenocarcinoma is much better than adenocarcinoma of the pancreas.

P238

### PANCREATIC CYSTIC NEOPLASMS

G.R. FRONDA, S. ENRICO, M.P. CAPOZZI, M. TOPPINO, R. LENZO, U. CATTANEO
Department of Surgery. University of Torino (Italy)
(Head: Prof F. Morino)

Malignant and benign pancreatic cystic neoplasma represent gererally 10% of cystic lesions of this gland. Macroscopically, these tumors manifest themselves as well capsulated circumscribed lesions. As far as the anatomo-pathological aspect is concerned we can distinguish them as cystoadenomas of the serous type or mucinous type (based on the glandular activity) and cistoadenocarcinomas in which the neoplastic proliferation could transpass the cupsula. The symptomatology, quite aspecific, is represented essentially by abdominal pain, anorexy and loss of weight, symptons related to the compression of the duodenum (of biliary tract more frequent and precocious in malignat forms). In more advanced cases there could be present pancreatic insufficiency (endocrine and esocrine). The diagnosis is based on US and CT; angiography permits to evaluate the invasivity of the neoplasm. The three patients that came to our observation presented an advanced clinical situation with pancreatic insufficiency (eso and endocrine) loss of weight, jaundice and pain. US and CT both significative have evidenced two malignant cases. In the case of cephalic localization we carried out a duodenopancreatectomy : the patient after 13 months did not show signs of recurrence. In the second case the gland appeared globally affected, with vascular involvement: a total pancreatecomy with splenectomy was performed; the patient did not show recurrences after three years. The last patient underwent chemotherapy for inoperability and died after 15 months. The therapy of choice must aim at the total exeresis of the tumor with glandular resections, more or less extended depending on the site Because this type of neoplasia is slow-growing and it usually remains localized for a long time, the total pancreatectomy is reserved to those cases in which most of the gland is affected by the neoplasia.

**P239**

### MUCIN-PRODUCING TUMOUR OF THE PANCREAS: REPORT OF TWO CASES

KOJIMA,Y [1)]., KINAMI Y[1), 2)]., AKIYAMA, T[1)]., SAITO, H[1)]., KOSAKA, T[1)]., KITA, I[1)]., TAKASHIMA, S[1)]., KONISHI,F[3)]., MATSUNO,H[4)]
The Second Department of Surgery[1)], The first[3)] and the Second[4)] Department of Pathology, Division of Cancer Research, Medical Research Institute[2)]. Kanazawa Medical University, Ishikawa, Japan

Mucin-producing carcinoma of the pancreas is characterized by marked dilatation of the main pancreatic duct due to the overfilling of mucin secreted from carcinoma cells. It was first reported in Japan as a new variant of the pancreatic cancer, and now all tumors of the pancreas characterized by the above-mentioned findings are called mucin-producing tumours, because benign lesions can also show a similar appearance on imaging examinations.

This paper reports two cases of mucin-producing tumour of the pancreas. Both patients had a markedly dilated main pancreatic duct detected by ultrasound (US) and computed tomography (CT). In addition, an enlarged papilla of Vater with a widely open orifice was shown by endoscopy. Pancreatectomy was performed in both cases and resected specimens were opened along the line of the main duct for examination. The tumours featured the intraductal proliferation of tall, mucin-producing, columunar epithelium. The histological diagnosis of the first case was intraductal papillary adenoma, while the second was a multicenteric papillary adenocarcinoma with microfocal periductal invasion. Both patients are currently in good health 7 and 5 months after the operation.

The diagnosis of this tumour can be made definitely by endoscopic retrograde pancreatography. Thus, when marked dilatation of the main pancreatic duct is encountered on US and/or CT, this examination is mandatory to confirm or exclude a mucin-producing pancreatic tumour.

**P240**

### CYSTIC NEOPLASM OF THE PANCREAS: CYSTADENOMA AND CYSTADENOCARCINOMA

M. MORENO; J.M. JOVER; M. LIMONES; L.M. DIAZ; J. ALVAREZ y C. MAILLO
Hospital Universitario de Getafe. Getafe. Madrid.

Cystic pancreatic neoplasms are not frequent and represent 10-15% of pancreatic cysts and 1% of the exocrine tumours. In this poster five cases treated in our Department are presented.

The cases correspond to four females and one male with an average age of 47.2 years. Symptomatology was inexpressive except in the malignant one in which an abdominal mass was palpated in the left hypocondrium. All cases were diagnosed preoperatively with ultrasonography, CT scan (3), angyogram (2) and aspirative cytology (1))

Malignant cyst (mucous cystadenocarcinoma) was located in the body and tail and was non resectable because of liver secondaries and retroperitoneal infiltration. Benign cysts (serous cystadenoma) was located in the head (2) (Whipple procedure one with piloryc preservation), body (1) (resection of body and tail with preservation of the spleen with its vessels) and tail (1) (distal esplenopancreatectomy) respectively. The average size was 5 cms.

The postoperative course was uneventful. They were treated with continuous perfusion of somatostatine (as is our protocole for pancreatic surgery).

The hospital stay was 9.2 days and in the follow-up (average 10.3 m.) all cases with benign cyst are asymptomatic.

Surgery is curative in benign cysts, and although malignant one have better prognosis than adenocarcinoma, they must be diagnosed at an early stage for resection.

**P241**

### THE RESULTS OF SURGICAL TREATMENT FOR PANCREATIC CYSTIC DISEASES

T. SUZUKI, M. YOSHIDA, K.SHIMADA, K.FURUTA, Y..ITOH, H.IZUMIKA, H.YOKOTA, G.KANEDA, S.FUNAMOTO, N.KOBAYASI, K.ASO, H.MIENO, K.SATO, H.OHMIYA, Y.HIKI, A.KAKITA
Department of Surgery, Kitasato University, School of Medicine, Kanagawa, Japan

This study is to evaluate the propriety of our surgical treatment for pancreatic cystic disease.

We have experienced 72 patients with pancreatic cystic disease including 49 with pseudocyst, six with cystadenoma, seven with cystadenocarcinoma, 10 with retension cyst. Twenty five cases underwent surgical treatment. (51.0%). The indications of surgery were abdominal pain, dysphagia due to compression of gastrointestinal tract, suspicion of malignancy, intracystic bleeding, pleural effusion, etc.

The surgical procedures for pseudocyst were mainly resection and cystjejunostomy. Twenty two patients were doing well after surgery. Six cases with cystadenoma, all were resected and still alive. Of seven cases with cystadenocarcinoma, two were resected, and one of them is still alive five years after surgery. Of 10 cases with retention cyst, one was resected and nine were followed without surgery.

In conclusion, complete removal or cystjejunostomy are the faorable proceduers for pancreatic pseudocyst. Cystadenocarcinoma should be resected and early detection is important to improve the result.

**P242**

### THE 'DUCT-ECTATIC' VARIANT OF MUCINOUS CYSTIC NEOPLASM OF THE PANCREAS : CLINICAL AND RADIOLOGIC STUDIES OF SEVEN CASES

TAKEDA T, NAGAKAWA T, NAKANO T, MORI K, KAYAHARA M, OHTA T, UENO K, MIYAZAKI I
Department of Surgery (II), School of Medicine, Kanazawa University, Kanazawa 920, Japan.

Seven cases of the "duct-ectatic" variant of mucinous cystic neoplasm of the pancreas are presented. In the present study, the "duct-ectatic" variant of mucinous cystic neoplasm of the pancreas was defined as the pancreatic cyst having the following characteristic radiologic and pathologic findings:(1), Pancreatic cysts are present which are at most 3-4 cm in size, and are in a diffusely duct-ectatic configuration, and are visualized as multilocular cysts communicating with the main pancreatic duct on ERP;(2)< Histologically, the cysts are multilocular and the epithelia of the cysts are composed of tall, mucin-producing columnar cells with atypia which are arranged in a single row or stratified pattern, and are indistinguishable from those of the classic "megacystic" type of mucinous cystic neoplasm. This new entity likely masquerades as pancreatic pseudocyst clinically and radiologically. However, it has the characteristic pathologic findings of the classic "megacystic" type of mucinous cystic neoplasm. If there are cysts visualized on pancreaticogram which are in a diffusely duce-ectatic configuration and are in communication with the main pancreatic duct, the "duct-ectatic" variant of mucinous cystic neoplasm should be strongly suspected rather than the pancreatic pseudocyst.

### P243

### CYSTIC NEOPLASMS OF THE PANCREAS

T. YANO, T.Iida, K. TANIGAWA, H.KIDA, T.SUZUKI, K.FUJIMORI
Department of Surgery, Saiseikai Matsusaka Hospital Matsusaka City, Mie 515, Japan

Cystic neoplasms of the pancreas are rare, accounting for 10% of pancreatic cysts. During the past 11 years, we treated 4 patients with cystic neoplasms of the pancreas.

Clinical Features of Patients with Pancreatic Cystic Tumour

| Patient# | Age & Sex | Symptoms | Imaging Dx | Preop. Dx. |
|---|---|---|---|---|
| 1 | 65 Male | epig. pain | US, CT, ERP | cystadenoma |
| 2 | 39 Female | abd. mass | US, CT, ERP | cystadenocarci |
| 3 | 72 Male | symptom (–) | US, CT, ERP | mucinous ductal ectasia |
| 4 | 57 Male | nausea | CT | pancreas cancer |

| Patient# | Operation | Tumor Size | Histological Prognosis | Diagnosis |
|---|---|---|---|---|
| 1 | PD | 1x1.2cm | serouscystadenoma | alive (4Y) |
| 2 | distal Px | 7x6cm | mucinous cystadenoma | alive (3Y) |
| 3 | PD | 2x3cm | atypical ductal hyperplasia | alive (1Y) |
| 4 | PD | 9x6cm | pleomorphic carcinoma | died (3M) |

Cytic neoplasms of the pancreas encompass a variety of tumours. While the pathological features of cystadenoma have been well described, mucinous ductal ectasia is a newly recognized premalignant lesion. Pleomorphic carcinoma is a very rare tumour, presenting cystic appearance.All of these tumours have shared clinical features that make preoperative discrimination difficult, and we believe cystic tumors of the pancreas should be removed.

### P244

### GEP NEUROENDOCRINE NEOPLASMS WITH BIOLOGICAL INACTIVITY:A NEW CLASSIFICATION

V. PERCOPO, L. COBELLIS, M.N.D. MAGLIO, F.P. D'ARMIENTO, B. TESAURO
1st Surgery Dept., 2nd Medical School, University of Naples, Italy

Neuroendocrine tumours can be classified, on the basis of presence or absence of typical endocrine syndrome, in Neuroendocrine Neoplasms with Biological Activity (NNBA) and Neuroendocrine Neoplasms with Biological Inactivity (NNBI). It is very difficult to classify the NNBI: we introduce a new subclassification. We have observed 70 patients with GEP neuroendocrine neoplasms: 55 (78.2%) with biological activity (NNBA), 15(21.8%) biologically inactive (NNBI). Only 1(6.6%), a gastrinoma, was a secreting NNBI: it is likely that this tumour produces an inactive hormone or has a receptorial lack or block. Fourteen non-secreting NNBI have been observed: among these we can distinguish forms with positive immunoistochemistry for specific hormone (11 carcinoid observed), and forms with negative specific immunoistochemistry, but with positivity to NSE and/or chromogranine. We have observed 3 of these forms (2 pancreatic, 1 gastric): they represent the 4.2% of GEP neuroendocrine tumors of our experience. This gastric tumour was been classified as anaplastic carcinoma, but because of a surprising survival it has been defined neuroendocrine carcinoma. These forms, positive to NSE and chromogranine, have a better prognosis than carcinomas, but the prognosis is generally poor if compared to the neuroendocrine differentiated tumours.

We know that frequently the anaplastic carcinomas can be confused with non-secreting NNBI. Therefore this subclassification is very useful to define the prognosis, because of the NNBI has a low proliferative index. In conclusion, our observations suggest that a routine dosage of NSE and chromogranine in neoplastic tissue might be useful for the prognostic evaluation and therapeutic strategy.

### P245

### PANCREATIC EXOCRINE AND ENDOCRINE FUNCTION AFTER OPERATIONS FOR CHRONIC PANCREATITIS.

RP JALLEH & RCN WILLIAMSON
Department of Surgery, Royal Postgraduate Medical School, Hammersmith Hospital, London, United Kingdom

Operations for chronic pancreatitis can radically affect pancreatic function. Exocrine and endocrine functions were assessed in the early postoperative period ($\leq$ 2 mo) and at long-term follow-up (mean 25 mo, range 3-120) in 103 patients (69 males, 34 females; age=42±11 years) undergoing operation for this condition. Alcohol was the main aetiological agent (69%). Drainage procedures (n=23) did not alter pancreatic function, even on long-term follow-up. In the early postoperative period, distal pancreatectomy (n=42) compromised endocrine but not exocrine function. Although only 7 patients (17%, $p<0.01$) became diabetic, mean GTT values in 28 cases showed substantial elevation of blood glucose ($p<0.05$). On follow-up, another 10 patients developed endocrine failure ($p<0.01$) and 11 exocrine failure ($p<0.01$).

Proximal pancreatectomy (n=38) precipitated clinical exocrine insufficiency in 14 patients (37%, $p<0.01$), but pancreoluaryl tests (n=18) did not confirm any measurable change. Endocrine function was initially spared, but 6 additional patients (16%, $p<0.05$) required treatment for diabetes at a mean of 19 months (range 3-34) after operation. Residual pancreatic function was often preserved therefore after these major procedures, although distal resection greatly impaired glucose tolerance and proximal resection unmasked a low enzyme output. Progression of disease probably accounts for subsequent deterioration. Drainage operations appear to delay this decline in function.

### P246

### SECONDARY EXOCRINE PANCREATIC INSUFFICIENCY FOLLOWING DIFFERENT TYPES OF GASTRECTOMY

H.KÖHLER, R.NUSTEDE, M.BARTHEL, F.E.LÜDTKE, A.SCHAFMAYER
Dept. of General Surgery University of Goettingen, Goettingen, FRG

In a follow-up study 19 patients after Billroth-I, 22 patients after Billroth-II resection and 38 patients after total gastrectomy (15 with Longmire-Gutegmann and 23 with Roux-en-Y reconstruction) underwent a stool fat determination, an indirect pancreatic function test with fluorescein dilaurate in serum and urine, and determination of CCK and neurotensin plasma levels. Results : In 9 of 19 cases after Billroth-I resection and in 12 of 14 patients after Billroth-II resection there were pathological PLT test results. In 9 of 15 patients after total gastrectomy and reconstruction of the duodenal passage and in 20 of 23 patients with exclusion of the duodendum PLT results were pathologic. CCK and neurotensin levels were elevated in all groups of patients. Highest level of the peptides were measured in gastrectomized patients with reconstruction of the duodenal passage.

### P247

### SEVERE DIABETUS MELLITUS : ANGIOGRAPHIC ASSESSMENT OF THE EFFECTS OF SURGERY

V.V.PORTNENKO, A.A.KLEMBOVSKY, N.F.KUZOVLEV, T.G.DUZHEVA
The Sechenov Medical Academy, Moscow, Russia

Angiographic and clinical assessment of the effeciency of pancreatic deportalization was performed in 35 patients with severe diabetes mellitus. Six months after the intervention, the obtained data were compared with the changes in blood glucose, acetonemia predisposition, the degree of polyneuritis. The study demonstrated that clinical improvement occurs with satisfactory blood flow in the shunt > 60 ml/min and the absence of collateral blood flow to the portal system in the left gastric vein. The collateral flow in the major curvature veins has no crucial importance in patients with satisfactory shunt dynamics. In patients with unsatisfactory results, an additional binding of the left ventricular vein, performed after six months, leads to the stablization of the condition. Thus performing surgical pancreatic deportalization in patients with diabetes mellitus, it is necessary to attain the satisfactory blood flow in the splenic vein, and, if possible, to block the development blood flow in the portal system through the binding of the left ventricular vein.

### P248

### EXPERIMENTAL STUDY OF EXOGENOUS INSULIN CAUSING GUINEA PIG GALLSTONE FORMATION AND EFFECTING ON ITS BILE ACID METABOLISM

J.S. SHI, Q.J. MA, S.G. LIU.
Hepato-Biliary Research Lab, First Affiliated Hospital of Xi'an Medical University, Xi'an P.R. China

Thirty guinea pigs were divided into two groups : group A and group B. In group a, 15 guinea pigs were injected general insulin 4μ twice a day subcutaneously, and in group B, 15 guinea pigs were injected normal saline 0.1ml twice a day. The injection continued 6 weeks, Blood samples were collected by heart puncture before start of the experiment , 3h after the injection and closely prior to the animals being killed respectively. RESULTS : 1. There were 12 versus 15 animals which lived to the end of the experiment in group A and in group B respectively. There were 7 versus 1 animals that have had gallstone formation in group A and in group B respectively (the morbidity were 58% versus 6.7%). There are a significant difference in morbidity of gallstone formation between group A and group B ($P<0.05$). 2. The volume of the tourchenodeoxycholic acid (TCDCA) are $0.597\pm0.39$mg/ml in group A and $0.757\pm0.43$mg.ml in group B. The volume of the glycochenodeoxycholic acid (GCDCA) are $2.4702\pm1.59$mg/ml in group A and $3.296\pm2.47$mg/ml in group B ($P<0.01$). 3. The volume of serum insulin is $120.07\pm40.38$(micro-until/ml) and $213.50\pm35.90$ before and after the injections respectively in group A ($P<0.01$). The results suggested that the exogenous insulin could reduce the volume of the bile acids in guinea pig bile and promote gallstone formation successively.

### P249

### LITHOGENOUS BILE IN SUBJECTS WITHOUT CHOLECYSTIC CALCULOSIS.

M. VELEGRAKIS, G. PERPIRAKIS, N.NIKOLAKAKIS, G. DOLAPSAKIS, A. APALAKIS.
Surgical department of Venizelio Gen. Hospital, Heraklion Creta

The aim of this study is to determine the lithogenous composition of bile in subjects without cholecystic calculosis and the search of bile with potential lithogenous composition. We studied bile samples of 14 patients without gall bladder calculi and with normal extrahepatic biliary tract. Bile speciments was taken by puncture from gall bladder during laparotomy for extra hepatobiliary disease. In those speciments cholesterol, phospholipids and bile salts was determinated quantitatively. The results was registred in triangular order. The mean value of percentage ratio of those components was 7.1%, 12.01% and 80.86% respectively. Values of cholic lipids in 9 bile samples was in the limits of highest cholesterol solubility. In the other 5 speciments the values of cholic lipids were found out of limits of cholesterol saturation, which indicates that those speciments have potential lithogenous composition. In conclusion we found that it is possible to have lithogenous bile in non chololithiasic subjects. There is a high possibility in these cases to form bile calculi in the future.

### P250

### FREE RADICAL APPEARS IN GALLSTONES IN VIVO*

T.SHEN, C.LIN, X.B.FU, X.S.ZHOU
Department of Surgery, Third Teaching Hospital, Beijing Medical University, Beijing 100083, China

Using electron paramagnetic resonance spectroscopy, Free radical (FR) signal was detected from either bilirubin or pigment gallstones (PS) dried in air. This study was designed for clarifying whether FR signal appears in gallstones in vivo. Gallbladders of 18 patients with stones in it were resected intactly. Under anoxic condition, fresh gallstone aliquot (anoxic sample) from each case were subjected to FR signal inspection at once. Another fresh aliquot exposed to the air served as control. Calcium bilirubinate (CaUCB) contents in stones were assaied with Fourier transform infra-red spectroscopy. FR signal with g=2.0038 was detected from the anoxic samples containing CaUCB, and a linear correlation between the intensity of FR signals and CaUCB contents revealed ($r=+0.95$; $p<0.0005$). The FR signal intensity in controls were many times stronger than that in the corresponding anoxic samples. During desiccation in air, FR intensity of anoxic samples increased faster than that of control did, eventually reached the intensity of controls and became stable.

DISCUSSION: FR signal initially appeared on CaUCB in PS in vivo and strengthed by the action of air oxygen on bilirubin. Most CaUCB in PS is present as its polymer, polymerization is often initiated by FR, and FR signal appeared in PS in vivo. This series of facts implied that FR might play an important role in pigment gallstone formation. We have confirmed this in animal modle and reported eleswhere. [* Supported by National Natural Science foundation of China]

**P251**

### CLASSIFICATION AND CHARACTERISTICS OF THE MACRO-GALLSTONES

ZHANG REN, LI JIN-CHI
Dept. of Surgery, The 2nd Central Hospital, Tianjin, P.R. China

We collected and observed some macro-gallstones from 25 patients from Jan. 1980 to Jan. 1991.

According to their cross-section structure and outward, the 25 specimens can be categorized into 5 patterns.

1. Stratified-Nucleus Stone: It's color is deep brown. It's section can be clearly divided into two parts-stratifications and nucleus.

(1) One kind of the nuclei is like piled sand.

(2) The other kind is collected crystalline grain.

Typical specimen: 9.5x6x5 $cm^3$ weight 102 g. (Fig. 1)

2. Silt-like stone: It's color is dark or deep grey and silt-like.

Typical specimen: 10x9x7 $cm^3$ weight 130 g. (Fig. 2)

3. Concretion stone: It's color is light yellow. A certain number of stones formed a kind of macro-stone tightly bounded up by adhesive aggregation.

Typical specimen: 7x5x5 $cm^3$, weight 60 g. (Fig. 3)

4. Shrapnel-like stone: It's color is brown or yellow-green.

It's shape is just the same as usual gallstone, but it's substance consists of several small stones.

Typical specimen. (Fig. 4)

Partly Shrapnel-like stone (Fig. 5)

5. Integument stone: A certain number of stones are encapsulated by fibrous integument. (Fig. 6)

In our opinion, besides the well-know macro-gallstones, the concretion stone and the integument stone can be included in this kind.

**P252**

### THE RELATIONSHIP BETWEEN THE REGIONAL ENVIRONMENT AND THE GENESIS OF GALLSTONES

LIANG FANG XIA*, LIN-DE ZHOU, MING-JU XIANG
*Dept. of Surgery, Affiliated Hospital of Guiyang Medical College, Guizhou Province
Institute of Geochemistry, Academia Sinica P.R. China

The relationship of regional environment and pathogenesis of gallstones in Guiyang City and Congjian Country had been studied. The geologic, geographic and climatic situations of these 2 places are different. Guiyang seats in alpine basin of middle Guizhou covered by limestone with a temperate climate, Congjian in south-east of Yunnan-Guizhou Plateau covered by slate with a subtropical mild-humid climate. The differences are also shown in gallstone types. There are 77.56% cholesterol stone in Guiyang while 68.89% pigmental stone in Congjian. The content K Na Mg Mn Cl Fe Cu Cr Co Al and P of stone in Guiyang and higher than those in Congjian. The drinking water of Guiyang is alkali hard water, pH 7.9, rich in Ca Mg ions and dissolved oxygen while Congjian is acidic soft water, pH 6.03, rich in Na K Cl ions. The content of Pb Ni and Cr in soil of the former are 21, 18 and 84 times greater than those in the latter respectively. On conclusion, it seems that cholesterol stones are mostly formed in alkali hard water area and pigmental stones in acidic soft water area.

**P253**

### NUTRITIONAL AND IMMUNOLOGICAL ASSESSMENT OF PATIENT WITH OBSTRUCTIVE JAUNDICE; EFFECT OF BILIARY DECOMPRESSION AND ABDOMINAL SURGERY

YUICHI ISHIDA, CHIKASHI WADA, KAZUHIKO MEIGATA, KENSHI WATANABE, YOSHIFUMI BECK, KUNJI MITA, FUYO YOSHIMI, SUMIO INOUE, SHINJI TOMIKAWA, HISAYUKI SUGIMOTO, TAKESHI NAGAO, AND HISANORI UCHIDA.
From the Institute of Medical Science, The University of Tokyo, Tokyo, Japan

A clinical study was undertaken to evaluate nutritional and immunological status of patient with obstructive jaundice who had had serum bilirubin level of 5 mg/dl or above (N=5). Non-icteric control was patient with cholecystolithiasis or gallbladder polyp who had undergone abdominal surgery (N=14).

Nutritional status was assessed by prognostic nutritional index (PNI), biochemical liver function, rapid turnover protein. Cellular and humoral immunity were assessed by natural killer assay, lymphocyte stimulating test, antibody dependent cell-mediated cytotoxicity, and serum immunoglobulines. Obstructive jaundice, benign or malignant, was associated with deterioration of both nutritional and immunological status, which were further depressed immediately after laparotomy. In postoperative convalescence, these indices started improving but not in a parallel fashion; recovery of nutritional indices was delayed relatively to the immunological ones. Considering known effects of nutrition on immunity, intensive nutritional support is thought to be mandatory for better perioperative management of patients with obstructive jaundice.

**P254**

### NEUTROPHIL RESPIRATORY BURST AND PRIMING BY IL-1 AND IL-6 IN OBSTRUCTIVE JAUNDICED PATIENTS

WG JIANG, MCA PUNTIS and MB HALLETT
University Department of Surgery, University of Wales College of Medicine, Cardiff, UK

Excessive production of neutrophil oxidative products are harmful, in sepsis and ARDS. We studied neutrophil respiratory burst and priming by Interleukin 1 & 6(IL-1,6) in obstructive jaundice patients.

10 patients with obstructive jaundice were studied with a matched control group. Respiratory burst was detected by a luminol-dependent chemiluminiscence (CL). Cells cultured with or without IL-1, IL-6 for 10 minutes and then stimulated with fmlp in the presence cytochalasin B. CL shown as mean±SEM CPS (count per second).

| | With medium | With IL-1 | With IL-6 |
|---|---|---|---|
| Control | 572.9±94.2 | 772.3±59.0* | 685.8±51.6* |
| Jaundice | 1147.1±213# | 1319.2±207.6# | 1253.8±199.6# |

*p<0.05 vs with medium, #vs control by Student T test.

Neutrophils from jaundiced patients show a greatly increased respiratory burst. Cells from controls can be primed by IL-1 and IL-6, but jaundiced patients cells failed to be further primed by these cytokines.

We conclude that neutrophils from obstructive jaundice are pre-primed in vivo and fail to be further primed by IL-1 and IL-6 in vitro. The increased production of oxidative products by these cells may therefore related to patients in vivo IL-1 and IL-6 level and may be related to patients clinical prognosis.

**P255**

### ROLE OF LEFT HEPATICOJEJUNOSTOMY IN MALIGNANT HILAR BILIARY OBSTRUCTION

T.K.MALIK, S.MADAN, R.MALIK
Dept. of Surgery, M.A.M. College, New Delhi

Analysis of the palliative biliary-eneteric by pass in 34 patients with unresectable malignant obstruction at the confluence of hepatic ducts was carried out. There were 16 men and 18 women, aged 31-73 years. The site of obstruction was shown on percutaneous transhepatic cholangiography. Left hepatic duct was used for anastomosis by lowering the left hepatic ductal system from the under surface of the quadrate lobe in 10 patients and the round ligament approach being used in 21 patients. In three patients, hepaticojejunostomy by the technique of longmire and sand ford was preferred. Overall hospital mortality was 21% and the mean survival was 10 months. Although tumor removal with adequate biliary-enteric repair is preferred treatment, but in patients with advanced disease, palliative biliary-enteric bypass is a suitable procedure because it avoids the problems of indwelling tubes which may become blocked and require replacement.

**P256**

### HEPATIC DYSFUNCTION IN BILE SECRETION FOLLOWING THE RELIEF OF OBSTRUCTIVE JAUNDICE

JUN MATSUMOTO, TOMOHITO MINAMI
Department of Surgery, Tokyo Metropolitan Fuchu Hospital, Tokyo, Japan.

Hepatic functional recovery, as indicated by bile secretion, after percutaneous transhepatic biliary drainage (PTBD) was investigated in 20 patients with obstructive jaundice with PTBD and 10 patients with bile duct stones with T-tube drainage as the control group. No differences between the PTBD and T-tube groups were found in bile flow and bilirubin output in the bile. However, the bile acid output in the bile was significantly less in the PTBD group than the the T-tube group. In the PTBD group the serum totoal bilirubin level was less than 5 mg/dl, the capacity of bilirubin output and indocyanine green secretion in the bile were not different from those of the T-tube group but the capacity of the bile acid secretion in the bile and the ursodeoxycholic acid tolerance test (UDCA-TT) were lower than in the T-tube group. The preoperative values in the UDCA-TT reflected the postoperative increase in serum total bilirubin in the PTBD group. The suppression of bile acid secretion still occurred in the patients with obstructive jaundice with serum total bilirubin levels of even less than 5 mg/dl after PTBD. This hepatocellular dysfunction could be a factor in hepatic failure following major surgery including hepatectomy. The UDCA-TT could be a predictive test for the capacity of bile acid secretion after PTBD.

**P257**

### NEUTROPHIL OXIDATIVE RESPONSE AND ITS RELATIONSHIP TO CLINICAL OUTCOME IN JAUNDICED PATIENTS

MCA PUNTIS, WG JIANG and MB HALLETT
Department of Surgery, University of Wales College of Medicine, Cardiff, UK.

Neutrophil oxidative products are known to cause tissue damage and they have been reported to be increased in trauma and sepsis. We have studied the neutrophil respiratory burst in surgical obstructive jaundice.

20 jaundiced patients were studied with matched controls. Neutrophils were purified and their respiratory burst studied by using luminol-dependent chemiluminiscence. Cells were stimulated by f-met-leu-phe (fmlp 1.0 μM) in the presence of cytochalasin B (5.0 μg/ml). The bursts were recorded before and after stimulation and are shown as counts per second (mean±SEM with 95% confidence intervals in brackets).

| | Before fmlp | After fmlp |
|---|---|---|
| Control | 130.0±20.2 | 9444.8±1317.2 |
| | (87.2-172.8) | (6651.9-12237.8) |
| Jaundice | 187.4±41.7 | 26698.9±2997.8 |
| | (100.1-274.7) | (20422.9-32975.0) |
| p value | 0.22 | <0.0001 |

Jaundiced neutrophils showed a slightly increased base line response and a massively increased oxidative response to fmlp. The three patients with the shortest survival time (2 to 50 days) in the group we studied had significantly higher levels of respiratory burst after fmlp stimulation (43097±6192, $p<0.05$) compared with the rest.

Conclusion: Neutrophils from obstructive jaundiced patients are activated, having a massively increased oxidative response the magnitude of which is related to the patients' outcome.

**P258**

### OPERATIVE TREATMENT OF PRIMARY DUODENAL MALIGNANT TUMOR-31 CASES REPORT

MENG SHAOQING, ET AL
Dept. of H-B Surgery, First Affiliated , Hospital of Xi'an Medical University

This paper analyses and summarizes 31 cases of primary duodenal malignant tumors during 1957-1986. The diagnosis being confirmed by operative findings and pathologic characteristics (radical operation in 17 cases, local or palliative resection in 4 cases, internal drainage in 10 cases). The longest postoperative survival reached 11 years. It is difficult to differentiate the diagnosis of primary duodenal malignant tumor from the cancer of periampulla and the head of pancreas. The purpose of this paper is to analyse and discuss the incidence, diagnostic methods and the effects of the operation on primary duodenal malignant tumor. It was emphasized that the early diagnosis was the key procedure to prolong the postoperative five-year survival. It was also proposed that local resection and palliative. Operation be taken for the old and weaken patients or the patients with localized lesions to prolong the survival time.

Key words : primary duodenal malignant tumor; operative treatment; incidence

### P259

### IMPAIRED GALLBLADDER FUNCTION FOLLOWING ABLATION OF THE SPHINCTER OF ODDI.

RP JALLEH, LA DESA, M RODDIE, RCN WILLIAMSON & JN THOMPSON
Departments of Surgery & Radiology, Royal postgraduate Medical School, Hammersmith Hospital, London, United Kingdom.

A poorly functioning gallbaladder is susceptible to stone formation, infection and carcinoma. Gallbladder function may be impaired by the various operative and endoscopic procedures that destroy or remove the lower bile duct sphincter. Gallbladder function was studied, using HIDA scanning and real-time ultrasonography, in 12 patients without a functioning sphincter of Oddi (previous operation n=6, endoscopic sphincterotomy n=6).

Gallbladder filling was not seen on HIDA scan 11 patients (92%). Ultrasound examination was performed with fatty meal stimulation (Lipomul 0.7 ml/kg body weight) and/or cholecystokinin stimulation (1 Ivy dog unit/kg body weight). Abnormal gallbladder emptying was detected in eight of these patients (73%) : no contraction n=3, paradoxical relaxation n=2 and suboptimal (<50%) contraction n=3. Normal (<50%) contraction was observed in three patients, two of whom had gallstones. Our data demonstrate that both gallbladder filling and emptying are markedly impaired in patients without a functioning sphincter of Oddi.

### P260

### SURGICAL TREAMTENT FOR PERIPAPILLARY SYNDROME IN THE AGED

YAN SEN HUA, YANG YI CUN,
Department of surgery, Chengdu Third People's Hospital, Chengdu, Sichuan, China.

Peripapillary syndrome (PPS) requires surgical treatment, and its reasonable operative procedure is worth-while to study. Since 1982, 4 cases of PPS, aged 64 - 73 have been performed electively with gastrectomy, gastrojejunostomy, cholecystectomy and end-to-side choledochoduodenostomy. They have been followed up from 1 to 9 years with good results. We suppose for a large diverticulum of the desending part of duodenum and the terminal part of the bile duct with disturbed function exelusions of both of them would be suitable. Besides, this combined operations could have a wide bilary-intestinal drainage and prevent the unavoidable biliary reflux and "sumpling" of the commonly used side-to-side choledochoduodenostomy.

### P261

### A CLINICAL STUDY OF POLYPOID AND WALL THICKENING LESIONS OF THE GALLBLADDER

T. AOKI, K. KIMURA, Y. KOYANAGI, A. TSUCHIDA, H. OZAWA, T. OZAWA, D. YASUDA, K. KASUYA,
Department of Surgery, Tokyo Medical College, Tokyo, Japan.

We analysed 81 cases of polypoid and wall thickening lesions of the gallbladder which were resected surgically at the view point of ultrasonography and clinicopathology.

The cases consisted of 29 cholesterol polyps, 3 hypertrophic polyps, 3 adenomas, 19 adenomyomatosis and others. They were 74 benign and 7 malignant lesions. In 49 polypoid lesions, 22 cases which measured under 5 mm indiameter were all benign, though 2 cases which measured above 5 mm in diameter were maligant. With respect to the shape of polypoid lesions cholesterol polyp predominated in type 1 and 2, adenoma and cancer in 2 (multiphyllous papillary) and 3, adenomyomatosis in 2 (simple papillary) and 4. 5 of 32 wall thickning lesions were malignant, though it was very difficult to distinguish between chronic cholecyctitis and them.

We concluded that it was necessary to take surgical treatment in polypoid lesions which measured above 5 mm in diameter except cholesterol polyp and wall thickening lesions not to be distinguished from chronic cholecystitis.

### P262

### PERCUTANEOUS TRANSHEPATIC BILIARY DRAINAGE (PTBD): INDICATIONS AND LIMITS (14 YEARS EXPERIENCE)

F. COLTURANI, G. TAGLIABUE, E. FALESCHINI, G. NERVETTI AND V. ROVATI
Istituto Scienze Biomediche L. Sacco - Catt. Patologia Chirurgica - Universita di Milano - Italia.

We present our experience of PTBD in patients with prolonged jaundice. In our Department, from 1976 to 1990, 527 patients underwent PTC. They were referred with prolonged obstructive jaundice with billirubinemia 10 mg/dl and dilated ducts. PTBD was performed in 468 pts. (88,9%). Malignant tumours were present in 349 pts. (62,2%): -pancreatic carcinoma: 202 pts. (57.8%) -gallbladder cancer: 21pts. (6%) -ampullary cancer: 11 pts. (3,1%) -liver metastases: 48 pts. (13,7%) -miscellaneous causes: 4 pts. (1,1%). Non malignant diseases were present in 119 pts. (33,8%): -bile duct stones: 71 pts. (59,6%) -iatrogenic injury to biliary ducts: 18 pts. (15,1%) -chronic pancreatitis: 18 pts. (15,1%) -inflammatory stenosis of Vater's papilla: 10pts. (8,4%) -acute cholangitis: 2pts. (1,6%). The PTBD was kept for an average period of 10 days (range 4-21). Major complications occurred in 23 pts. (4, 9%): -septic complications: 7 pts. (1,4%) -bleeding: 12 pts. (2,5%) -displacement: 35 pts. (7,4%) -miscellaneous causes: 4 pts. (0.8%). 3 pts. underwent emergency surgery (0.6%). Displacement of the drainage, promptly restored, occurred in 35 pts. We believe that PTBD should be the treatment of choice for jaundiced patients when immediate surgical procedure is not available. Low incidency of complications confirms this choice.

**P263**

### MAY THE T-TUBE BE THE CAUSE OF ANATOMICAL ALTERATIONS OF THE COMMON BILE DUCT, THROUGH THE TIME ?

P. CARBOGNANI, L. SPAGGIARI, P.DELL'ABATE ,P. SOLIANI, R. SABBAGH, A. MISELLI AND E.FOGGI.
Department of General, Thoracic and Vascular Surgery, University of Parma, Parma, Italy.

The aim of the work is to assess if the T-tube used to drain a choledochotomy, performed for different pathologies, has caused anatomical alterations of the common bile duct after a long period of time. We have recalled, in September 1991, 40 patients, 15 males (37%) and 25 females (63%), (mean age 48 years, range 29-74 years), that, from January 1985 to October 1986, have undergone a choledochotomy, for choledocholithiasis in 37 cases (92%) and cholangitis in 3 cases (8%), leavng a T-tude in the common bile duct. In all the patients was performed a supraduodenal choledochotomy closed with an interrupted suture of absorbable monofilament. The main diameter of the common bile duct on preoperative ultrasound was of 14mm. (range 8-20mm). The drains were removed after 10 days from the operation, previous x-Ray and manometric control. In one patient was performed an endoscopic sphincterotomy for a residual stone, and the drain was removed after 20 days from surgery. In the last September (mean 70 months from surgery; range 59-81 months) all the patients have undergone an ultrasound examination, performed by the same radiologist with specific experience. We have noticed that : in 36 cases (90%) the common bile duct was normal in diameter (>4mm. and <9mm.) with regular walls; in one case (2%) the diameter was of 3mm. without signs of pericholedochal inflammation; in the remaining 3 patients the common duct was slightly dilated (11mm.), with a stone in only one patient. These data show that, unlike different experiences, there aren't, after years alterations of the common bile duct caused by the the T-tube.

**P264**

### INFECTIVE COMPLICATIONS OF BILIARY SURGERY.

N.DORAIRAJAN
Department of Surgery, Madras Medical College, Madras

Abstract : In a series of 120 patients under going cholecystectomy with or without Common bile duct (CBD) exploration, bacteriological study was carried out on gall bladder (GB) tissue and bile, CBD bile and blood and post-operatively on T-tube bile, T-tube limb and blood. GB tissue and bile culture was positive in 27.3% and 40% respectively. The CBD was explored in 18% and its bile was positive in 68.2% of cases. Pre-operative blood cultures were positive in 10.3%. The commonest organism isolated was E.Coli. Post-operative T-tube bile was positive in 60.1% on the 3rd day, 45.6% on the 7th day and 40.5% on the 10th day with decrease in E.Coli and corresponding increase in exogenous Staph aureus. T-tube on removal showed 50.8% positive cultures with predominance of Staph. aureus. Post-operative blood culture was positive in 3.3%. 1.5% cases had wound infection. Septicaemia occured in 0.6% cases. The study suggests that the T-tube is a constant source of infection and a cause for post-operative morbidity.

**P265**

### REPLACEMENT OF THE T TUBE AFTER BILIARY SURGERY: OUR PERSONAL METHOD.

G.R. FRONDA, S. ENRICO, M.P. CAPOZZI, M. TOPPINO, U. CATTANEO
Department of Surgery,. University of Torino (Italy)
(Head : Prof. F. Morino)

The T tube (Kehr) is an excellent solution following choledocotomy although subsequent removal of the tube presents a number complications. In order to reduce the risks involved, an easily applied method has been used, which has given good results in our experience. This method has been applied to eight patients in which a T tube had been inserted following choledocotomy for removal a choledocal stones. After an average period of 10 days, an x ray examination of the biliary tree was made through the Kehr tube. Having excluded any type of alteration affecting the biliary tree,. a metal guide was inserted through the Kehr tube, the T tube was removed and external biliary drainage was inserted. After this replacement, no biliary collection was noted in the following days while a good flow of bile from the drainage was observed. After an average period of 24-48 hours, with an absolute assurance about the absence of any biliary fistula, the drainage was removed. The advantages of this method can be summarised as follows : fairly early removal of the T tube, the possibility of draining the biliary tree, thereby promoting easier closing due to the more reduced collection bile permitted by this method, easier closing of any laceration, or disruption of the choledocotomy without requiring positioning of percutaneous or endoscopic biliary drainage.

**P266**

### STUDY OF LABELLING INDEX OF BROMODEOXYURIDINE AND ORNITHINE DECARBOXYLASE ACTIVITY IN HUMAN GALLBLADDER MUCOSA.

TAKASHI FUJITA, HIROSHI SHIMADA, GIZO NAKAGAWARA, The First Department of Surgery, Fukui Medical School, 23 Shimoaizuki, Matsuoka-cho, Yoshida-gun, Fukui, 910-11, Japan.

To investigate the pathogenesis of gallbladder cancer labeling index of bromodeoxyuridine (BrdU LI), which indicate DNA synthesis rate, and ornithine decarboxylase (ODC) activity, which indicate promotional activity of oncogenicity were examined in 20 cases of human gallbladder mucosa with reference to its relation to pathological findings or biliary biochemical elements. The high value of BrdU LI & ODC activity of gallbladder mucosa were seen in order: in cancerous mucosa, noncancerous mucosa with gallbladder cancer, mucosa with cholecystolithiasis, mucosa with pancreaticobiliary malfunction, mucosa with no choledocal disease. With relation to pathological findings high frequency of metaplastic change showed high values of BrdU LI & ODC activity in the cases with gallbladder cancer, pancreaticobiliary maljunction and cholecystolithiasis (LI>5%).

The rate of lithocholic acid was most related to high values of BrdU LI & ODC activity in the cases with cholecystolithiases.

In conculusion, high frequency of metaplastic change in gallbladder mucosa and the rate of lithocholic acid in bile were thought to be important as pathogenic factors of gallbladder cancer.

### P267

### CHARACTERISTICS OF AMINO ACID COMPONENT IN TWO TYPES OF GALLSTONE

LIANG-FANG XIA, ET AL
Guiyang Medical College

Amino acid components and their contents in 8 gallstones collected from Guizhou Province had been analyzed. The total content was determined to be 1158.08-78141.7ug/g, and there were 17 kinds of amino acids in these stones, such as GLY, ALA, YAL, ILE, LEU, SER, THR, CYS, MET, ASP, GLU, ARG, LYS, PHE, TYR, HIS and PRO, of which 11 kinds of amino acids occured in all samples, while ARG, HIS and MET were only found in different single samples. Obvious differences between bilirubinate and cholesterol stones were observed as follows : (1) The total amino acid content of the former was higher than that of the latter; (2) GLU and ASP were the principal components in the former, and on the contrary, GLY was the major one in the latter; (3) CYS was absent in all samples of the former but was found in all samples of the latter; (4) Although the neutral amino acid was the principal component in both types of gallstone, yet the ratio of acid to alkaline in the former was greater and higher than that of the latter. As acid-salts are only stable under alkaline conditions, bilirubinate gallstones might be formed under alkaline environment, and cholesterol stones under weak-alkaline to acidic environment.

### P268

### EXTRACORPOREAL SHOCK WAVE LITHOTRIPSY IN THE MANAGEMENT OF RECURRENT COMMON BILE DUCT STONE

CHI-REN HWANG, CHENG-CHUNG WU, MEI-DUE YANG, TAIN-CHENG WU, TSE-JIA LIU, AND FANG-KU PENG
Division of General Surgery, Dept. of Surgery, Taichung Veterans General Hospital, Taichung, Taiwan, R.O.C.

The use of extracorporeal shock wave lithotripsy (ESWL) in the management of 16 patients with acute cholangitis and recurrent common bile duct stones is described. Using a SIMENS Lithostar lithotriper, fragmentation of the stones was done without general anesthesia. In all cases, naso-biliary catheters were established before the procedure to allow access for radiographic localization during the procedure and ensure free drainage of infected bile.

Fragmentation of the stone was successful in 13 patients by ESWL and the extraction of the stone was done by endoscopic mechanical lithotripsy. Clearance of the bile duct stone was confirmed by cholangiography and sonography. Morbidity was minimal and ESWL can be a valuable adjunct in the management of patients with recurrent CBD stones.

### P269

### A STUDY OF THE MINERAL COMPONENT OF GALLSTONES

LIANG-FANG XIA, et al
Guiyang Medical College

The results of mineralogical study on gallstones are presented in this paper. Six kinds of minerals, native zinc, native aluminum, magnetite, pyrite, gypsium and aragonite have been found in them. Except for the last one, all the others are first discovered in gallstone. Although the minerals are minor components in it, yet they may carry some valuable information about the formation environment of gallstones.

### P270

### PROSPECTIVE RANDOMISED STUDY COMPARING AUGMENTIN AND CEFAMANDOLE IN PREVENTING WOUND INFECTION IN HIGH RISK PATIENTS HAVING ELECTIVE SURGERY FOR GALLSTONES.

JEJ KRIGE, EJ IMMELMAN.
Department of Surgery, University of Cape Town and Groote Schuur Hospital, Cape Town, South Africa.

In a prospective randomised single blind study, we compared the efficacy of Augmentin and Cefamandole in preventing wound infection in 150 high risk patients undergoing elective surgery for gallstones. Each patient had at least 1 risk factor for biliary sepsis. Randomisation to receive one of the two study drugs was by computer-derived random numbers in sealed opaque envelopes with 75 patients in each group. Augmentin (1.2gm) was given q8 hrs for 3 doses beginning 30 mins before operation and 4 doses of Cefamandole (lgm) were given q6 hrs. Mean age (A57.8 vs C 58.0 years) and ratio of men to women (A21:54 vs C 22.53) were similar. Risk factors for post-operative infection in each group included acute cholecystitis <1/12 (A 32 vs C 34) recent rigors (A 23 vs C 16) CBD stones (A 17 vs C II) diabetes (A 7 vs C 12) and were similar. Mean pre-operative biochemical parameters in each group were: alk phos: A 286 vs C 209 (normal <100), WBC A 8.7 vs C 8.6 and ESR (mm/hr Westergren) A 51 vs C 42. The number of positive bile cultures were A 48 vs C 34. In the Augmentin group, 51 patients underwent cholecystectomy alone compared to 55 in the Cefamandole group, CBDE (A 11 vs C 9) CDD (A 11 vs C 5) revision biliary drainage (A 1 vs C 1) hepaticojejunostomy (A 1 vs C 5). Similar number of patients had an uncomplicated post-operative course (A 70%) vs C 73%). The incidence of post-operative fever were similar. Four patients in each group had wound infection. The number of days in hospital: Augmentin 10.1±4.7 and Cefamandole 9.7±5.6 were similar.

We conclude that the efficacy of Augmentin and Cefamandole in preventing wound sepsis in high risk biliary surgery is similar. Economic factors may favour the use of Augmentin.

### P271

### HEPATIC ARTERY ISCHAEMIA IN RATS WITH CHOLESTASIS. DIFFERENT RESPONSE OF THE LOBES.

L. LORENTE, M.A. ALLER, S. ALONSO, J. ARIAS, H. DURAN, J.L. BALIBREA.
Department of Surgery I. Hopsital Universitario San Carlos, Madrid, Spain.

The tolerance of the rat to the cholestasis could be influenced by the different distribution of the hepatic artery. Wistar rats were divided in three groups: Groups I was the sham operate rats (14), Group II (14) the bile duct was ligated and divided, Group III (22) underwent ligation and section of the bile duct and hepatic artery. In Group II: 35.7% of the rats died during the first sixteen postoperative days, 35% had sepsis and 7.1% local infection. In Group III, 60% of the cases, the macroscopic aspect of the liver was homogeneous (subgroup C1), while the rest of the animals (subgroup C2), some of the lobes had a yellow coloration and were enclosed in a big bile duct cyst. In this subgroup 44.4% of the animals died, 33% had sepsis and the serum bilirubin were normal. In subgroup C1 there were no deaths, 7.6% had sepsis and the serum bilirubin was increased. The different evolution of the animals suggests different responses of the liver lobes when associated with bile duct and hepatic artery ligation.

### P272

### TREATMENT OF COMMON BILE DUCT INJURIES VIA LAPAROSCOPIC PROCEDURES

F.E. LÜDTKE, H. KÖHLER, T. NEUFANG, A. SCHAFMAYER, G. LEPSIEN
Dept. of General Surgery, Univ. of Göttingen

Iatrogenic injury to the common bile duct during laparoscopic cholecystectomy has previously necessitated an immediate laparotomy to alleviate bile leakage. In the course of 171 laparoscopic cholecystectomies at our hospital, an intraoperative common bile duct injury occured in two patients that, in each case, was successfully treated utilizing a laparoscopically placed T-tube. A laparotomy was therefore not necessary. This novel intraoperative procedure was sufficient in treating common bile duct injury without the occurence of postoperative complications.

### P273

### TUMOUR NECROSIS FACTOR PRODUCTION BY MONOCYTES IS NOT INCREASED IN PATIENTS WITH OBSTRUCTIVE JAUNDICE

D MCCRORY, I HALLIDAY, M HOPER, S STEPHENS, B ROWLANDS
Dept. of Surgery, The Queen's University of Belfast.

Introduction: Endotoxin and its cytokine mediator Tumour Necrosis Factor (TNF) are thought to play a major role in the complications associated with biliary obstruction. We hypothesize that monocytes from jaundiced patients may produce increased basal levels of TNF and may be over-responsive or refractory to stimulation by exogenous endotoxin due to prior exposure

Methodology: Peripheral blood monocytes (PBM) from 15 jaundiced and 10 control patients were isolated by centrifugation over histopaque and percol gradients. $2x10^5$ monocytes were aliquoted into duplicate wells and into each pair either 0 or 10 ng/ml lipopolysaccharide (LPS) was added. Supernatants were harvested at 5 and 24 hours. TNF was measured by an ELISA. Cytospins were analysed and TNF quantified per $10^6$ monocytes.

Results: TNFpg/ml/$10^6$ monocytes (Median+interquartile range)

| | Ong/ml LPS 5 hours | Ong/ml LPS 24 hours | 10ng/ml LPS 5 hours | 10ng/ml LPS 24 hours |
|---|---|---|---|---|
| **Jaundice** | **342** | **74** | **3092** | **714** |
| | (124-687) | (29-174) | (1520-6134) | (224-3805) |
| **Control** | **244** | **118** | **3905** | **1026** |
| | (86-1432) | (77-375) | (2633-9952) | (528-2180) |

No significant difference jaundice vs controls at both time points and LPS concentrations (p>0.05 Mann Whitney)

Conclusions The similar response to endotoxin of PBM from jaundiced and control patients refutes our hypotesis and indicates the need for further study of the mechanisms of production and significance of TNF in obstructive jaundice.

### P274

### PROLIFERATIVE ACTIVITY OF BILE DUCT EPITHELIUM FOLLOWING BACTERIAL INFECTION IN DOGS

OHTA T, NAGAKAWA T, TSUKIOKA Y, TAKEDA T, MORI K, NAKANO T, KAYAHARA M, UENO K, AND ITSUO MIYAZAKI.
Departments of Surgery (II), School of Medicine, Kanazawa University, Kanazawa, Japan.

In order to clarify the relationship between proliferative activity in bile duct epithelia and bacterial infection, we induced obstructive cholestasis with a bacterial infection in two lobes of the liver. The bile duct branch draining the left lateral lobes of the liver was cannulated in all mongrel dogs. The dogs were divided into three groups and treated as follows: Group 1, the cannula was clamped after the injection of $10^7$ E-coli cells; and Group 3, the cannula was clamped without the injection of any bacteria. Three months and nine months later, dogs from each group were sacrificed and their livers were examined. In the Group 1 dogs, papillary hyperplasia and severe dysplasia were noted in association with chronic cholangitis on 3 months and 9 months, respectively, after their operation. In the Group 2 dogs, periductal fibrosis was severe, but epithelial papillary hyperplasia was found to be less pronounced than in the Group 1 dogs at each period. In the Group 3 dogs, there was no periductal fibrosis or epithelial papillary hyperplasia. These findings suggest that papillary hyperplasia and/or severe dysplasia of the bile duct epithelium may be caused by aerobic and anaerobic bacterial infection in combination with bile stasis.

### P275

### COMPARATIVE VALUE OF ULTRASONOGRAPHY (US), PRE- AND INTRAOPERATIVE X-RAY STUDIES IN EXAMINATION OF PATIENTS WITH GALLSTONE DISEASE (GD).

H. POOLA, A. VIIKLEPP, J. TROOST, P. ALAS
Dep. of Surgery, Estonia Seamen's Hospital, Tallinn, Estonia.

The aim of the study was to analyse the role of preoperative (PO) US in diagnosis of GD, to compare the results to PO biligraphic examinations and to find out the necessity of intraoperative (IO) roentgenological telecholangioscopy and -graphy (ORTCG). 600 patients were operated on for GD from 1986-1990. US was performed in 95.5% of cases (in recent 3 years 100%). PO infusion cholangiography was used in 14.7% of cases (in 1986-40.4%). In US there were 6 false-positive (1%) and 15 false-negative cases (2.5%). In these cases we found the gallstones by intravenous cholangiography. The value of US was 96.5%. ORTCG was made through the cannulated cystic duct in 45.5% of patients, in 1990-23.7%. ORTCG was performed in cases of jaundice or pancreatitis, dilated CBD, unclear findings in US and PO cholangiography. ORTCG was also made in cases of dilated cystic duct in connection with a great number of small gallstones. Choledochotomy was performed in 12.3% of cases. The number of CBD exploration decreased in connection with increased number of planned operations and with improvement of PO and IO examinations. We conclude that US is the most informative method in the diagnosis of GD. PO infusion cholangiography is a complementary method in cases of unclear findings in US> Indications to ORTCG must be individualized according to the case histroy, PO and IO findings.

### P276

### INTRA-OPERATIVE ULTRASOUND OF THE BILIARY TREE AT A MIDSIZED COMMUNITY/MILITARY HOSPITAL

JEFFREY D. SEDLACK, M.D., AND BRIAN F. STAINKEN, M.D.
United States Naval Hospital, Camp Pendleton, CA, USA

We studied the feasibility of intra-operative ultrasound examinations of the biliary tree at a 100 bed hospital and examined our own philosophy of intra-operative management of a finding of tissue at choledochotomy.

Four patients came under our care in one year with distal ductal obstructive jaundice and were taken for scheduled, daytime exploration. In all cases, the ultra-sound machine and radiologist were called for intra-operative examination. In two cases with pancreatic head masses, the expected wait for the machine exceeded 1 hour and the request was withdrawn. In two cases of ductal pathology the time from call to complete set-up was less than 1/2 hour and evaluation was obtained. In each of the cases, incision of a dilated common bile duct had an expression of obstruction tissue (1-carcinoma, 1-blood clot). The patient with hemobilia had normal fiberoptic choledochoscopy and intra-operative ultrasonography; the blood was felt to be from hemorrhagic cholecystitis. The other patient had a proximal 1/3 common duct tumour identified by the intra-operative ultrasound. He underwent high ductal resection but had metastases by 7 months.

We have concluded that in a smaller hospital without a dedicated ultrasound in the operating area, the routine use of intra-operative ultrasound is not expedient. However, with tissue expressed at choledochotomy, intra-operative ultrasound is a useful tool for intra-operative decisions.

*The views expressed in this abstract by Navy authors do not reflect the official policy or position of the Department of the Navy, Department of Defense, nor the U.S. Government.

### P277

### ARF (ACUTE RENAL FAILURE) AS SEVERE COMPLICATION IN PATIENTS WITH OBSTRUCTIVE JAUNDICE.

C. SIMOPOULOS, A. POLYCHRONIDES, A. BOUNOVAS, A. PAPAVASILIOU.
Propedeutic Surgery Clinic, Democritus University of Thrace, Alexandroupolis, Greece.

ARF as a postoperative complication in jaundice.

Patients with obstructive jaundice are a group of high mortality and complication after operation. The post-operative ARF in patients with obstructive jaundice is the scope of this study.

We refer to (112) patients - 62 males and 50 females (average age 62,4 years).

The patogenesis of jaundice in 52 cases was benign and in 60 malignant. After surgery there was an increase of blood urea and creatinine in 38 patients (33,9%) and severe ARF in 8 patients (8,9%) 6 of those died (5,3%).

The main Risk factors are:

a) Ht>30%

b) Bilirubin >10g.

c) Age>65 years

Conclusion: A.R.F. in a severe complication in patients with obstructive jaundice and these patients must be treated with great care.

### P278

### THE FIBRINOLYTIC SYSTEM AND THE BILIARY TREE

DM SCOTT-COOMBES, SA WHAWELL, JN THOMPSON
Department of Surgery, Hammersmith Hospital,
Du Cane Road, London, England

The aim of this study was to investigate the firbinolytic properties of bile and gallbladder tissue.

Paired biopsies of gallbladder serosa and mucosa and aspirations of gallbladder bile were obtained from eight patients undergoing routine cholecystectomy Tissue homogenates and bile were assayed for plasminogen activator activity (PAA) and the concentrations of the fibrinolytic mediators; tissue plasminogen activator (t-PA), plasminogen activator inhibitor 1 and 2 (PAI-1, PAI-2) and urokinase (u-PA).

Both bile and gallbladder tissue possessed PAA (median; bile:0.3IU/ml, serosa:3.3IU/$cm^2$, mucosa:1.4IU/$cm^2$). The bile PAA was predominantly due to t-PA (median 24.0ng/ml) rather than u-PA (median 0.3ng/ml). Fibrinolysis in the bile may be controlled by the presence of PAI-1 (median 6.4ng/ml) and PAI-2 (median 370.0ng/ml). Both t-PA (serosa 3.6ng/ml, mocosa 2.4ng/ml) and u-PA (serosa 1.9ng/ml, moucosa 1.9ng/ml) were detected in the tissues but the concentrations of PAI-1 and PAI-2 were below the level of detection.

we have demonstrated the presence of PAA within the biliary system. This fibrinolytic activity may be responsible for anastomotic biliary leaks may also act to prevent deposits of fibrin acting as a nidus for gallstone formation.

### P279

### POLYPOID LESIONS OF THE GALLBLADDER: CLINICAL AND PATHOLOGICAL CORRELATIONS

G.N.XU, ET AL,
Institute of Hepatobiliary Surgery, Changhai Hospital,
Shanghai, P.R.China

Fourty patients (21 males, 19 females; mean age 43.95±11.58 years) and had polypoid lesons of the gallbladder, which were confirmed surgically and pathologically. The incidence of the disease was 15.8% in patients undergoing cholecystectomy and 1.4% in patients having ultrasonography. In the 40 patients,23 (57.5%) had cholesterol polyps,5 (12.5%) inflammatory polys,4 (10%) adenocarcinomas,3 (7.5%) xanthomatous polyps,2 (5%) papillary adenomas, 1 mixed polyp,1 neurofibroma, and 1 adenomatoid proliferative polyp. The mean age of patients with nontumorous polyp 41.27±10.16 years. In these patients, 78.8% had polyps less than 5 mm in diameter, and 69,7% had multipe lesions. However, the mean age of patients with tumorous polyp was 56.71±9.79 years and 71.4% of the patients had polyps more than 5 mm in diameter and 85.7% single lesions.72.5% of the patients had clinical symptoms. Polyps were detected ultrasonographically, but one third of them were overlooked by either cholecystography or CT.

### P280

### CHOLESTEROLOSIS OF THE GALLBLADDER : DYSPEPTIC SYNDROME OR SURGICAL DISEASE?

M. SIANESI, F. CETTA*, A.M.FARINON*, P.TARASCONI, E. ZANELLA*
Patologia Chirurgica - Universita' Di Parma *Patologia Chirurgica - II Universita' Di Roma

In order ro evaluate the clinical significance of cholesterolosis, a particular chronic gallbladder disease, 41 patients, 32 females and 9 males (F/M ratio, 3.5:1), aging 19 to 61 years (mean age: 43.3 years), who undergone cholecystectomy between 1982 and 1991 were considered. Patients with concomitant presence of stones or adenomyomatosis were excluded. In 18 cases a strawberry gallbladder, in 15 a single cholesterol polyp, in 8 two or more cholesterol polyps were discovered. It is not an unusual clinical problem, being present with an incidence of 5-25%, but in symptomatic patients without gallstones it is not always clear when surgical treatment is indicated. Each dyspeptic symptom and biliary pain, colic or not, have been considered. The most useful methods for the detection of these lesions are ultrasonography and oral cholecystography; cholecystokinin test for pain reproduction was used in 8 patients only, because of undesired effect on colonic motility. Cholescintigraphy, gastroduodenoscopy and sometimes laparoscopy are useful in differential diagnosis. Follow-up after cholecystectomy was satisfactory in all but one patient. Therefore we recommend the surgical treatment in all symptomatic patients when there is no evidence of other gastrointestinal disease.

### P281

### MANAGEMENT OF SEVERE BILIARY TRACT BLEEDING WITH BALLOON CATHETER

ZHANG CHANGGONG, ET AL
The hepatobiliary Department of Surgery, The Second Hospital of Chongqing, China

To treat severe biliary tract bleeding (SBTB), hepatocholangiostomy, ligation of hepatic artery and lobectomy of liver are often used clinically. Here, we introduce a new method, balloon tamponade, with which 12 patients suffering from SBTB have been treated with good results.

A modified single balloon with tri-cavity catheter (4mm outside calibre) was used. In the front and the back of the balloon, there are two separated drainageways and openings through which we can observe the characteristics of the drain and hemostatic result. It is also useful for us to perform other procedures, such as local drainage, lavage, drug injection and cholangiography, and thereafter further diagnosis and treatment.

During exploration, duct incision is somewhat superior in order to expose the branch of bilateral hepatobiliary duct. Bleeding site is identified after clearing hematocele. Tamponade is then complished by inserting a balloon catheter to control hemorrhage temporarily. After that, another similar catheter is inserted through the papilla to take the place of the former one. The balloon is inflated to press the bleeding area. The catheter is fixed after adjusting at the best site. The end of the catheter is brought out through the abdominal incision out of the body. Six hours later, the balloon is deflated for 30 minutes. If there is no persistent hemorrhage, the balloon is not inflated any more. If there is a little oozing of blood, wash and inject some hemostatics. Other surgical maneuver should be performed if severe bleeding is not controlled.

Using this method, we have treated 12 patients with acute SBTB, four of whom were preoperative hemorrhage, severe anemia in three, medium anemia in one. Another eight cases developed acute SBTB operatively, the lost blood was 400-1, 200ml each, with an average of 580ml. Nine patients' bleeding was controlled immediately, two after eight hours, one after forty hours, based on drain observation. None needed further operation nor died. All patients recovered and were discharged from hospital in 25 - 35 hospital days.

The balloon catheter not only can be used to control SBTB but also has a function of choledochostomy which is superior to T-tube in drainage of bile duct except hemorrhagic area. While tamponading, the drain from two drainageways can be compared and examined, and different bile duct segments can be given drugs irragated and cholangiographed. It is of important significance in further treating and predicting prognosis afterwards. The balloon presses directly the bleeding area, exerting an acute hemostatic effect because it decreases blood perfusion, slows the blood flow of the tissue around the hemorrhagic area, and tend to form embolism. The method is so simple, less damaging, safe and easy to perform that any basic hospitals which hold condition for ordinary biliary surgery can popularize it.

### P282

### MEMBRANE FATTY ACID SATURATION RISES IN OBSTRUCTIVE JAUNDICE

MW SCRIVEN, JCM STEWART*, DF HORROBIN*, MCA PUNTIS
Dept Surgery, University Wales College Medicine,
University Wales Hospital, Gardiff, UK and *Efamol Research Institute, Kenttville, Nova Scotia, Canada.

We investigated the effect of obstructive jaundice (OJ) on the saturation of membrane fatty acids (FA).

FA in red cell (RBC) phospholipid were measured by gas chromatography in 42 patients with OJ (mean age 65, 19F:23M, mean bilirubin 247μmol/1, 12 benign:30 malignant) and 42 matched controls. The results were compared by Mann-Whitney U tests.

Total saturated FA (SFA) ($p<0.001$) and total monounsaturated FA (MUFA) ($p=0,001$) were raised in the OJ patients. There was a corresponding fall in total polyunsaturated FA (PUFA) ($p<0.001$), total n-6 PUFA ($p=0.001$) and total n-3 PUFA ($p<0.001$). These changes were reflected by a fall in the mean number of double bonds per FA ($p<0.001$) and a rise in the ratio of MUFA+SFA:PUFA ($p<0.001$), indicating an overall rise in saturation.

Certain aspects of membrane function, in particular phagocytosis by reticuloendothelial cells, are abnormal in OJ. Changes in membrane FA can have the same effect. If reflected in other cells these highly significant changes in RBC FA saturation may thus explain some of the defects in membrane function in OJ and open new afenues of therapy.

### P283

### PLASMA AND MEMBRANE EICOSANOID PRECURSORS ARE ABNORMAL IN OBSTRUCTIVE JAUNDICE

MW SCRIVEN, JCM STEWART*, DF HORROBIN*, MCA PUNTIS
Dept Surgery, University Wales College Medicine, University Hospital Wales, Cardiff, UK and *Efamol Research Institute, Kentville, Nova Scotia, Canada

This study investigated the effect of obstructive jaundice (OJ) on plasma and membrane polyunsaturated fatty acid (PUFA) prescursors of eicosanoids.

Plasma and red cell (RBC) phospholipid PUFA were measured in 42 patients with OJ (mean age 65, 19F:23M, eman bilirubin 247μmol/1, 12 benign 30 malignant) and 42 matched controls, by gas chromatography. The levels of the 3 major 20 carbon PUFA eicosanoid precursors arachidonic acid (20:4n-6), dihommogammalinolenic acid (20:3n-6) and eicosapentaenoic acid (20:5n-3), and total 20 carbon PUFA (c20-PUFA) were compared using Mann-Whitney U tests.

C20-PUFA was lower in the OJ group ($p<0.001$ in each fraction), and each precursor was lower in both fractions ($p<0.005$ in each case). The balance of the individual precursors was abnormal, with a rise in 20:4n-6 as a proportion of c20-PUFA ($p<0.001$ plasma, $p<0.05$ RBC) and corresponding falls in the proportions of 20:3n-6 and 20:5n-3,

Substrate availability is a major determinant of eicosanoid production. As the eicosanoids are an important influence on immune and reticuloendothelial function, the changes demonstrated by this study, in particular the shift towards the immunosuppressive 20:4n-6, may thus explain some of the cell defects in these systems and allow new modes of therapy.

### P284

### INTEGUMENT STONE ON BILIARY TRACT

ZHANG REN, WANG CHONG
Dept. of Surgery, The 2nd Central Hospital, Tianjin, China

In clinical practice, we discovered a special kind of cholelithiasis. It, was confirmed by the information retrieval, DIALOG ON-LINE, for the first report in the world. According to its characteristic we name it "Integument or Encapsulation Stone."

**CLINICAL MATERIAL**

Integument stone may lie in gallbladder or choledoch in flowing state. Among 1,500 gallstone specimens, the author himself analyzes and observes only two specimens of integument stone.

**1. Typical Case :**

Woman, aged 55, suffered from chronic cholelithiasis for ten years. Because of choleperitoneum, she was treated with operation, from which we discovered choledoch perforation, gallbladder seriously inflammed and existence of a stone. Twenty days after operation, she recovered.

**Specimens :**

By dissecting gallbladder, we noticed that gallbladder was full of green liquid and flowing a conical body, very irregular, 7cm in height and 2.5cm base diameter. Integument looked pale, and not of strong tissue.

Dissecting integument,we found there were seven stones, round or oval in shape, large and small, the surface of each stone was dry and smooth, without membrane.

Inside the body, there were no separations, nor any liquid filling the space between the stones. (Fig 1)

After pathologic examination of integument, it is concluded as fibrous tissue. (Fig 2)

By Masson's staining analysis, it mainly contains myofibrilla and small quantity of collagenous fibres. (Fig 3. 4)

**DISCUSSION ON THE CAUSES, MECHANISMS OF INTEGUMENT STONE**

1) Integument tissue may be made from stratum fibrous tissue.

(a) when biliary tract is inflamed or collisions by stones, mucous membrance of gallbladder may be injured, broken or destroyed. By meaned of cell implantation or transmigration integument stone may be gradually formed.

(b) large slices of mucous membrance fibrous tissue flap in gallbladder fall off if just meet the stone and cover on it.

2) Occult hemobilia on biliary tract.

(a) The injury of gallbladder may cause bleeding in small quantity, and finally, clot binds up stone. Because clot cannot be diluted by bile in time, thus it may stay in stable state, making clot fibrosis and forming membrane.

(b) Occult hemobilia on Biliary duct forms integument stone. For the above-mentioned reason integument stone can be formed in biliary duct.

Similarly, it can also be in gallbladder, if blood frequently flow into it.

3) The two causes as mentioned above acting together may cause integument stone.

**DIFFERENTIAL DIAGNOSIS ON INTEGUMENT STONE**

1) Pseudocapsule stone often inlays at the neck of gallbladder, mostly a single stone. It may be tightly bound up, partly or wholly, by the wall of gallbladder, But its appearance is quite different from integument stone. (Fig 5)

2) Diverticular or pressure diverticulum of gallbladder may inlay stones which may be partly bound around the wall of gallbladder tissue. (Fig 6)

3) Concretion. A certain number of stones are tightly bound up by adhesive aggregation. After pathologic examination only some scattered, decayed cells can be seen, but it is quite different from pyogenic infection. At present, this phenomenon cannot be explained. (Fig 7)

4) A single stone, as large as a gallbladder, inlays inside, and is bound tightly and completely up by the walls of gallbladder where it is in, forming gallbladder a solid body. (Fig 8)

**CLINICAL CHARACTERISTIC AND SIGNIFICANCE**

1) Patient usually has long case history due to the opposition against operation treatment, so that serious biliary tract affection repeatedly relapse.

2) If a number of stones are encapsulated in one, integument stone practically becomes a big stone with integument protected. Its big volume may easily cause obstruction in the biliary tract. Therefore, the appropriate method to be adopted should be operation.

3) Careful diagnosis must be taken, as clots in the biliary tract may often be mis-diagnosed as gallstone.

After all, the cause of integument stone, we believe, is due to bleeding in the biliary tract. Because its appearance is so much like that fibrous menbrane remaining from old ectopic pregnancy found in a later operation, its leads us to believe it to be true, although Masson's staining confirms it is mainly of myofibrilla. But the definite causes need further discussion.

**P285**

### THE RESEARCH OF MICRO-EXPLOSION OF BILIARY CALCULI

YANGDE ZHANG, et al
First Affilited Hospital, Hunan Meical University City, Country Changsha, Hunan 410008, P. R. China

The achievement of this research is a successful application of the technique of micro-explosion to the disintegration of biliary calculi (stones in the intrahepatic bile ducts) of patients withour surgery. Up to the present, two hundred and fifty cases of biliary duct and gallbladder calculi have been successfully treated. The success rate of blasting stones was 100% by use of the directional micro-explosive instrument made by us, the once success of micro-explosion was 95.2% and the result of treatment are satisfactory.

Considering the density of the blood vessels in the human liver, limitation of space in the hepatic duct, the security and adaptation, we have designed two type of micro-explosive equipment by different ways to initiate explosion by electric initiation and nonelectric initiation. The diameter of the explosive head is only 1.5-1.7mm so as to fit with the diameter of the chaledscope. We have performed 2850 sample experiments on each of the designed condition parameter and the products of explosion by means of micro-pressure transducer of 1.8mm head and a week signal amplifier which was manufactured by us. In addition, we used the technique of high speed photography, hologram and so on. We observed and analysed the course of explosion of biliary stones, the pressure of struck wave and the gaseous products of explosion.

Because these equipment have satisfactory properties and reliable detective and monitoring device, they have been widely used clinically. In addition, they have been used widely in many industrial fields. It has been proved that the directional property of the micro-explosive instrument is safe and reliable by animal tests and 1805 clinic cases.

**P286**

### EXTENDED HEMIHEPATECTOMY WITH USING ND : YAG LASER RADIATION

V. P. BASHILO, V.V. UTKIN, M.U. BOBROVSKY
Central Clinical Hospital, Moscow, USSR

The purpose of our investigation is profylaxis of intraabdominal complications following liver resection. We solve this task by perfecting the surgical technique by way of implementation of Nd: YAG Laser.

7 right-side extended hempatectomy and 9 left-side ones were performed, 12 of them were performed in case of single cancer metastasis and 4 - in case of hepatocellular cancer. The specifics of the operation is disfocusing Nd: YAG Laser ray treatment of operated liver surface. The method provides fuller hemostasis and cholestasis. As a result the amount of postoperational discharges decrease.

There were no postoperational complications.

**P287**

### SERUM BILE ACIDS IN LIVER RESECTED PATIENTS

U. BOLDER, J. TACKE, M. IMHOFF, D. LOHLEIN
Dept. of Abdominal Surgery, Klinikum Dortmund, Beurhausstr. 40, 4800 Dortmund 1, BRD

Liver resection is an accepted surgical option in the treatment of liver secondaries of colorectal cancer or other malignant diseases. Nevertheless the surgical trauma is hardly to evaluate. The main factors that may contribute to surgical trauma are the mass of resected functioning liver parenchyma and the extension of the perioperative ischemic period due to the pringle procedure. Also the patient's age may be a reason for reduced tolerance to any kind of trauma. The aim of this study was to evaluate whether there is a parameter that parallels to the perioperative trauma.

Patients : 43 liver resected patients were investigated. 16 trisegmentectomies and hemihepatectomies (group 1; major resection) and 27 bisegmentectomies or segmentectomies (group 2; minor resection) were compared to 10 controls with major upper GI-operations. Methods: Besides standard serum profiles the course of serum lactate, ammonia and bile acids were correlated to extension of resected parenchyma, ischemic trauma, and age.

Results: Bile acid levels were highest in group 1 followed by group 2 and the controls. A significant distinction correlated to the amount of resection (p<.05) could be made untill postoperative day 7 in the Wilconon test. Values of serumlactate were significantly different only on the operative day. No differences were found in the postoperative ammonia levels. For investigation of the ischemic trauma 3 groups with different ischemic periods (0-30min, 31-50min,>50min) were regarded. Results of serum bile acids and lactate were significantly different (o<.05) untill the first postoperative day only. Ammonialevels were equal in all groups. When correlated to patients age (<45y,46-65y,>65y) no parameter showed any difference.

Conclusion:
-Levels of serum bile acids are correlated to the extension of parenchyma resection in the first postoperative week.

-The investigated parameters do not represent reliable the ischemic trauma due to the pringle procedure.

-A specific course related to the patients age could not observed in any of the traced parameters.

**P288**

### THE VALUE OF INTRA OPERATIVE ULTRASOUND (IOUS) IN HEPATIC SURGERY

S.S. HANNA
Division of General Surgery, Department of Surgery,
Sunnybrook Health Science Centre, University of Toronto, Toronto, Ontario, Canada

A prospective study of all patients undergoing IOUS during hepatic surgery was initiated with the goal of determining its value.

Between October 19, 1989 and November 19, 1991; 29 patients underwent IOUS during the conduct of liver procedures. In 7 of the 29 patients (24.1%) undergoing IOUS there was a change in the operative plan because of the findings of IOUS. In 6 cases the planned liver procedure was abandoned. In one case the procedure was extended from a segment (seg) 2,3 excision to a seg 2,3, 4A and 4B excision in order to obtain a better tumour margin as a result of IOUS findings. Two of these 7 patients wre cirrhotic; in 1 patient an additional metastasis was seen and in another tumour invasion of the portal vein by a hepatoma precluded resection. 5/7 were not cirrhotic; in 1 invasion of the left hepatic vein was seen; in 2 patients additional metastases were seen in seg 1. In one patient an additional metastasis in seg 3 made resection impossible.

In conclusion, IOUS was found useful in 24% of patients undergoing this examination during hepatic surgery. In this subgroup of patients, it prevented us from proceeding with liver resections that would have been oncologically useless.

### P289

### HEPATIC SEGMENTECTOMY USING A MICROWAVE TISSUE COAGULATOR AND GUIDED BY INTRAOPERATIVE ULTRASONOGRAPHY

S. HE, Hepato-Pancreato-Biliary Research Unit,
The First University Hospital, West China University of Medical Sciences, Chengdu, Sichuan, China

The aim of this study is the application of Microwave Tissue Coagulator (MTC) in hepatic segmentectomy to minimize perioperative bleeding. 26 patients were selected in this study among whom 20 were primary hepatocellular carcinoma and the rest consisted of angioma of the liver, cancer of the gallbladder and intrahepatic stone. The performance of the hepatic segmentectomy was done by the application of microwave hyperthermia cancer-therapy system with the insert applicators made in Sichuan University and was guided by Intraoperative Ultrasounography (IOU).

The results showed: the amount of blood lost ranged from 100 to 2,000 ml per patient with as average of 420 ml per patient and the amount of blood transfusion needed per patient ranged from 200 to 2,400 ml with an average of 500 ml.

Complications: Hemoglobinuria occured in 8 cases and bile leakage in 1 case. No intraoperative death or other complications related to this operation whatever.

It was, therefore, concluded that Hepatic Segmentectomy using a MYC guided by IOU is considered to be a safe and effective operational procedure for liver cancer management.

### P290

### BACTERIAL TRANSLOCATION INTO PORTAL BLOOD FROM GUT DURING PORTAL TRIAD OCCLUSION IN RATS

LX LIU, B JEPPSSON, S BENGMARK
Department of Surgery, Lund University, S-221 85 Lund, Sweden

Occlusion of the portal triad (PTO) is used during liver resection and liver transplantation. This study was undertaken to evaluate if bacterial translocation occurs during PTO and to explore any relationship with portal blood flow and endotoxemia.

METHODS: PTO was performed in rats for 15, 40, 60, 90, 120'. Bacterial translocation was studied in portal and systemic blood after intraluminal instillation of $^{131}$I-E.coli. Blood flow in the intestinal wall and portal vein was studied by laser Doppler flowmetry and portal $pO_2$ as well as endotoxin levels were measured.

RESULTS: Bacterial translocation into the portal blood could be detected already 15' after PTO. This increased with increasing length of PTO. Blood levels of E.coli were 3-4 times higher in portal than systemic circulation. Plasma endotoxin levels in portal and systemic blood were significantly increased. Laser blood flow in the gut mucosa and mean portal $pO_2$ were decreased in all experimental groups during PTO. The appearance of bacteria was proportional to increased endotoxin levels and inversely proportional to decreased blood flow in the gut.

SUMMARY: There may be an intimate relationship between increased bacterial translocation to portal blood and decreases blood flow of the gut mucosa. Increased bacterial translocation may play a role in postoperative infections after PTO in hepatectomy and after liver transplantation.

### P291

### PLATELET ACTIVATING FACTOR ANTAGONIST (PAF) WEB 2170 BS IMPROVES REPERFUSION INDEX OF THE LIVER AND INCREASES SURVIVAL AFTER HEPATIC INFLOW OCCLUSION IN RATS

LX LIU, B JEPPSSON, HO HEUER, S BENGMARK LUND
Department of Surgery, Lund University, S-221 85 Lund Sweden; Department of Pharmacology, Boehringer Ingelheim KG, Rhein, Germany

Ischemic organ injury is associated with microcirculatory failure. PAF has been proposed as a potential key- mediator of this. It is released from endothelial cells, platelet and neutrophils. We tested a new potent platelet activating factor antagonist, WEB 2170 BS in the prevention and protection of the liver from ischemic injury in rats.

METHODS: Normal rats had portal triad occlusion (PTO) for 90' during normothermia. WEB 2170 BS was administered in a dose of 1 mg/kg body weight or 3 mg/kg body weight intravenously in the jugular or portal vein 10' before PTO and 3' prior to reopening of PTO. Perfusion fo the liver was determined by laser Doppler flowmetry.

RESULTS: Administration of WEB 2170 BS before and after PTO significantly increased the survival rate from 8% in control rats to 86%. The reperfusion index of ischemic liver was also significantly elevated to 95% from 55% in control animals 1h after declamping of portal triad. On histological examination the ischemic damage was also reduced in groups treated with WEB 2170 BS.

SUMMARY: It seems that the platelet activating factor antagonist WEB 2170 BS is effective in prevention and protection of the liver from warm ischemic damage.

### P292

### LIVER REGENERATION AFTER HEPATIC RESECTION FOR BENIGN AND MALIGNANT LIVER DISEASE

U.KANIA, J.KALFF, M.ZIEGLER
Department of Surgery, Bonn University Medical School, Sigmund-Freud-Str. 25, 5300 Bonn 1, Germany

24 patients were investigated before and after elective hepatic resection in a prospective trial. 16 of these patients had malignant liver lesions, the remaining 8 suffered from benign liver tumors. Due to inoperability and other reasons the follow-up could only be achieved in 11 patients, 6 with malignant and 5 with benign liver disease.

The results showed a total regeneration of the removed functional liver tissue within 3 months. The volume of the removed tumor mass was not regenerated. A significant difference between benign and malignant tumors could not be demonstrated. The extent of the hepatic resection showed no influence on the regenerative process.

**P293**

### SURGICAL INDICATION AND RESULT OF HEPATIC RESECTION

S. T. KIM, K. U. LEE, G. P. KIM
Department of Surgery, College of Medicine, Seoul National University, Seoul National University Hospital, Seoul, Korea

Between 1981 and 1990, a total of 402 hepatic resections were completed including 319 primary malignancies (279 hepatocellular carcinoma(HC); 29 cholangiocellular carcinoma(CC); 3 mixed carcinoma; and 5 other primary malignancies), 42 intrahepatic cholelithiasis, 35 benign masses, 4 secondary malignancies, and 2 gallbladder carcinomas. Other primary malignancies included hepatoblastoma, malignant lymphoma, biliary cystadenocarcinoma, undifferentiated embryonal sarcoma, and leiomyosarcoma. Benign masses included hemangioma (12), abcess(8), cyst(7), adenomatous hyperplasia(3),armatoma(2), adenoma(1), cystadenoma(1), and pseudolymphoma(1).

214 patients experienced 241 nonlethal complications. Spesis was the most frequent complication, manifested primarily as wound infection(71) or intra-abdominal infection(25). Nonfatal hepatic failure occured in 9 patients with cirrhosis and 1 patient without cirrhosis.

Overall operative mortality was 9 %. Factors contributing to the death were hepatic failure(25), intra-abdominal sepsis(5), hemorrage(3), esoageal varix beldding(3), renal failure(1), and severe chest infection(1). 28 were patients who had pre-existing cirrhosis. Details of mortality after various kinds of resection are right trisegmentectomy, 1/4; left trisegmentectomy, 1/1; right lobectomy, 9/73; left lobectomy, 0/36; central bisegmentectomy, 0/3; right segmentectomy, 9/49; left segmentectomy, 2/72; subsegmentectomy or partial resection, 15/164.

The mortality after resection for HC, CC, intrahepatic cholelithiasis, and benign mass, respectivelym was 32/282, 4/24, 1/42, and 1/35. Mortality rate of HC with and without cirrhosis were 11.3% and 11.5%, respectively.

**P294**

### EARLY POSTOPERATIVE CHANGES IN DENSITY AND SIZE OF THE LIVER AFTER PARTIAL HEPATECTOMY

G. KRUPSKI[1)], R. MAAS[2)], U. MEYER-PANNWITT[1)]
University of Hamburg. Dep. for General Surgery[1)] and Radiology [2)]

Modern CT not only offers imaging but metric tools as well. Because we noticed postoperative swelling and chnages in density after partial hepatectomy we were interested is using these technique to quantify our observations in a prospective approach. We performed CT-scans in 20 patients who underwent partial hepatectomy for benign and malignant lesions 2 weeks, 3 and 6 months after resection in order to prove these findings statistically.

Results: We can demonstrate a statistically significant postoperative liver-volume increase of 232 ml (16%) ($p<0,001$; $T=5,92$) compared to the remaining parenchyma. Parallel there is a decrease in liver density from 59 H.U. to 53 H.U.

Possible reasons for these changes like e.g. narcotic drug effects, extent of resection, duration of operatin, duration of ligation of the hepatoduodenal ligament will be discussed.

**P295**

### FLUID AND AIR COLLECTION AFTER PARTIAL HEPATECTOMY: PRELIMINARY RESULTS OF PROSPECTIVE STUDY

G. KRUPSKI[1)],R. MAAS[2)], U. MEYER-PANNWITT[1)]
University of Hamburg. Dep. for General Surgery[1)] and Radiology [2)]

Due to the increasing frequency of liver surgery both radiologists and surgeons more often have to face with postoperative liver imaging. Particularly to differentiate between ill defined fluid collections (abcesses) and seroma is difficult.

We performed CT-sscans in 20 patients who underwent partial hepatectomy for benign and malignant lesions 2 weeks, 3,6 and 12 months postoperatively to rule out what still is "normal".

In 75% (15/20) 2 weeks postoperatively large fluid collections and in 90% air in the fluid collection were visible. Abcesses could be proved using clinical data, laboratory findings and microbiological fluid sample examinations in 5 out of 15 patients (33%) initially presenting with large fluid collections and air content.

| Examination | fluid(*) | | | | free air |
|---|---|---|---|---|---|
| | >4cm | 2-4cm | <2cm | no | |
| after 2 weeks | 15 | 3 | 1 | 1 | 18 |
| after 3 months | 5 | 6 | 7 | 2 | 5 |

To conclude our results: fluid and air collections are common postoperative findings after partial hepatectomy. Only laboratory and clinical findings in combination with CT-imaging can lead to the likely diagnosis of an abcess.

**P296**

### BAND TECHNIQUE FOR RESECTION OF THE LIVER

CHUNG-HAN LEE, KYUNG-HYUN CHOI, SUNG-DO LEE, JAE-KWAN SEO AND YOUNG-HOON PARK
Dept. of Surgery Kosin Medical College, Pusan, Korea

Now today liver resection can be performed quiet safety. Nonetheless, hemorrhage from a raw liver is the most common problem during hepatic resection. Therefore new instruments and methods for hepatic resection were designed and developed for controlling of the hemorrhage and shortening of the operation time. This study is a result of use to band technique of the liver resection for disease of the liver during 2 years from 1990 to 1991. The band (Panduit (R)) is a polyrophylene strip with a self locking key that measures 70cm in length, 0.8cm in width, 0.2cm in thickness and can be formed a loop with 20cm in a maximum diameter.

The indication of resection were primary liver cancer in 12 patients, secondary liver cancer in one patient, benign tumor in 4 patients and trauma in one patient. Of the hepatic resections performed, extended right lobectomy was done one case, right lobectomy in 14 cases, left lateral segmentectomy in 2 cases and partial resection in one case, with total 18 cases.

The average time of operation, amount of cut marginal bleeding, volume of total hemorrhage and volume of blood transfusion were 3 hours 50 minutes, 200cc, 3000cc and 7 units in whole blood, respectively. Thirty day-postoperative death was one case (5.6%) and due to hepatic failure. The method of band technique has good results compare to conventional hepatic resections at our hospital in same period.

So that this study suggested that is a useful operative method for major hepatic resection.

**P297**

### ANALYSIS ON 286 CASES OF HEPATIC LOBECTOMY

LIU ZI-XIEM AND ZHENG JIANG-HUA
Department of General Surgery, People's Hospital of Guangdong Province

286 cases of hepatic lobectomy have been operated from 1965 to 1990 (192 males, 94 females). Among them 154 cases were undergone hepatic lobectomy or removal of segements and 132 cases with hemihepatectomy or more than that. The operations were performed mainly by irregular lobectomy. Hemostasis was carried out by intermittent ligation of hepatoportal blood flow, manual compression (with fingers) or cutting with microwave blade during the operation. Post-operative complications were observed in 41 cases, 12 died within one month postoperatively. None died during the operation.

Primary and secondary hepatoma, hepatolithiasis are the major indication for surgical treatment. Most patients having hepatoma complicated with hepatic cirrhosis. Special attention should be paid to retain the functional hepatic tissues during the operation. Rupture of hepatoma causing severe bleeding can be stopped successfully by means of hepatic lobectomy. The results are good in short term. While multiple hepatoliathiasis having indication for hepatic lobectomy, a better prognosis was seen in partial hepatectomy compared with merely taking stones out through hepatic duct. Short term hepatic failure after hepatic lobectomy may be due to temporary imbalance (decompensation) of hepatic function. Active action should be taken immediately.

**P298**

### SIGNIFICANCE OF HEPATIC VEIN RECONSTRUCTION IN HEPATECTOMY

S. NAKAMURA, R. NISHIYAMA, Y, YOKOI, Y. OONUKI, S. SAKAGUCHI, S. BABA
Second Dept. of Surgery, Hamamatsu Univ. Sch. of Med. Hamamatsu, Japan

Hepatectomy combined with reconstruction of hepatic vein was performed in 8 patients who had carcinoma located close to the confluence of the hepatic vein and the inferior vena cava. Reconstruction of the right hepatic vein was performed in three patients, that of the middle hepatic vein was done in four, and that of the left hepatic vein was done in one. Iliac veins, superficial femoral veins and great saphenous veins were used for autografting. Follow-up periods ranged from 2 to 36 months.

There was no operative death. Patency rate of the veins was 75% one month after operation. Prothrombin activity and serum AST level were 69 ± 8.7% and 285±166 IU on the 1st POD, respectively. Hepatic scintigraphy of the two patients who had an occluded graft revealed a reduced uptake one month after operation. Eight patients are all doing well without evidence of recurrence of carcinoma.

In such patients, hepatic vein reconstruction contributed to preserving the residual liver function and achieving a more radical operation. Hepatic vein reconstruction is to be a new surgical option in hepatectomy.

**P299**

### INTERACTION BETWEEN VAGAL AND IMMUNOSUPPRESSIVE CONTROL ON LIVER REGENERATION IN RATS

M. OHTAKE, T. SAKAGUCHI*, K. NAKADAIRA, T. AONO, K. YOSHIDA, T. MUTO
Departments of Surgery and Physiology*, Niigata University School of Medicine, Niigata 951, Japan

The vagus nerve has been shown to stimulate liver regeneration (LR). Recentl, it was revealed that an immunosuppressive agent FK-506 (FK) also stimulates LR> This study was examined the interaction betwen the vagal and immunosuppressive control on LR in rats. The animals weighing about 180g were used. The 2/3 hepatectomized rats were classified into four groups; control, hepatic branch vagotomized, FK (0.1 mg/kg) administered, and FK with vagotomy. LR was evaluated by the liver weight as a percentage of body weight (RLW), and the mitotic index of the hepatocytes (MI). Plasma scores showing hepatic or renal function were estimated. The control and hepatic branch vagotomized had no difference in RLW following 3 days after hepatectomy. FK increased RLW and FK 3 days after hepatectomy compared to the control. However, no increase in RLW and MI was seen when vagotomy and FK administration were combined. Seven days later, the vagotomy reduced RLW compared to the control but there was no difference in RLW between the control and FK with vagotomy. The plasma scores maintained within normal range in all grups of animals. These observations indicate that LR by vagal control is predominantly active than by immunosuppressive control within several days after hepatectomy, but the latter becomes active thereafter.

**P300**

### SIMPLIFIED INTRAABDOMINAL VENOUS BYPASS DURING HEPATIC TOTAL VASCULAR EXCLUSION IN CANINE LIVER SURGERY

Q.QU, K. Y. HOU, X.S. ZHOU, H.H. CHEN, J. YUAN, C. LIN, W.X. ZHANG, Y.Z. LI, J. N. LU, J. SHI
Department of Surgery, 3rd. Hospital of Beijing Medical University, Beijing, P. R. China

An intraabdominal venous bypass was performed in 10 dogs for liver surgery during hepatic total vascular exclusion with the use of a modified method without pump and vascular anastomosis. A special catheter was designed and adopted to settle in the intrahepatic vena cava and to shunt the portal vein with a branch of the catheter while occluding the total hepatic blood flow. During 4-6 hours period of bypass, the hemodynamic alterations were monitored and multipe hematological biochemical indices were observed at periodic intervals. Biopsies were taken from the small intestine, pancreas and kidney at the end of the operations. Portal and IVC pressure were measure to evaluate the amount of blood flow through catheter according to a pressure-quantity curve obtained from extracorporal experiment. The results signify that the hemodynamic alternations were promptly returned to approximate prebypass level and the hematological biochemical indices were stable. It also shows there was no evidence of the congestion in the splanchnic system. This study proved to be a technically simple and satisfactory procedure in complicated major liver resection and transplantation.

### P301

### LIVER REGENERATION AFTER PARTIAL HEPATECTOMY IN RATS: A QUANTITATIVE ULTRASTRUCTURAL STUDY

C.G. SCHULZE, U. N. RIEDE
Departments of Surgery and Pathology University of Freibury, Germany

Hepatic regeneration in rats was examined by quantitative electron microscopy, Liver biosies were performed on 25 rats 7, 14, 30, and 60 days after partial hepatectomy and compared with those made on 20 adult male Sprague-Dawley rats. Specific ultrastructural chandes occur in the mitochondria, in comparison to the control group the hepatocytes of all the rats subjected to partial hepatectomy showed a larger number of smaller mitochondria and an increase in the amount of cristae membranes per cell. The volume density of the rough endoplasmic reticulum (RER) was greater in all rats after resection than in the controls. These changes in the ultrastructure increased up to day 30 after resection. On day 60 the values were below those recorded on day 30, but above those of the controls. The increase in the amount of cristae membranes and increase in density of RER indicate raised respiratory activity in the hepatocytes as well as an increase in the rate of protein synthesis during liver regeneration following partial hepatectomy.

### P302

### HEPATOCELLULAR CARCINOMA IN CIRRHOSIS. PROGNOSTIC FACTORS FOR LONG-TERM SURVIVAL AFTER SURGICAL RESECTION.

G.P. SPINA.L.CAPUSSOTTI*, E. OPOCHER, R. SANTAMBROGIO, B. ONGARI, H.BOUZARI*. University of Milan: S. Paolo Institute of Biomedical Sciences;
*Department of Surgery, Mauriziano Umberto I° Hospital-Torin, Italy

The purpose of the study is to determine the prognostic factors for long-term survival after heptaic resection for hepatocellular carcinoma (HHC). 72 patients who underwent resection between 1983 and 1989 were included inthis retrospective study. In a first step 15 variables were chosen, as indicated by the literature and our own surgical experience, and divided in 3 groups: clinical, tumoral and surgical. The variables were then studied with a univariate and Cox' multivariate analysis. The favoring conditions determined by Cox' analysis were: 1) patient's age over 60 years, 2) presence of a capsule, 3) monfocal lesions. Vascular infiltration, well differentiated carcinoma and a resection margin >1 cm., significant only by the univariate analysis are recommended buth not determining factors.

In conclusion from this study of prognostic factors in HCC we can say that tumoral anatomopathologic features are the most important factors in determining long-term survival.

### P303

### FACTORS RELATING TO DIC AND COAGULOFIBRINOLYSIS AFTER HEPATIC RESECTION

H. T. TANIGUCHI, A. OGURO, K. MIYATA, Y. UESHIMA, K. TAKEUCHI, N. TSUKUDA, K. SEIKI, M. HONDA, A. NOGUCHI, K. KITAMURA, A. HAGIWARA, T. YAMANE, T. YAMAGUCHI, K. SAWAI, O. KOJIMA, AND T. TAKAGASHU
First Department of Surgery, Kyoto Prefectural University of Medicine, Kyoto, Japan

The changes of factors relating to DIC and coagulofibrinolysis after hepatic resection were examined. In a blood survey of 24 patients who had undergone hepatic resection, the serum levels of thrombin-antithrombin III complex, $\propto_2$-plasmin inhibitor-plasmin complex, prostaglandin $E_2$, D-dimer, fibrinogen, and FDP increased in the early postoperative stage. The level of 6-keto-prostaglandin $F_1\propto$ increased after the operations. The level of thromboxane $B_2$ and platelet count decreased at first, and then gradully increased and returned to its preoperative level on postoperative day 7. Superoxide dismutase activity increased at first, and then gradually decreaded, postoperatively. That is, biodefensive ractions which protect patients against the shift to disseminated intravascular coagulation and superoxide dismutase activity, despite the induction of a predisseminated intravascular coagulation state after liver resection, due to overacceleration of coagulofibrinolytic function.

### P304

### THE EFFECT OF PROSTAGLANDIN E1 ON HEPATIC FUNTION AFTER LIVER RESECTION

Y.UESHIMA, H.TANIGUCHI, K.MIYATA, K.TAKEUCH, A.OGURO, N.TSUKUDA, T.TAKAHASHI,
First Department of Surgery, Kyoto Prefectural University of Medicine, Kyoto, Japan

Whether prostaglandin E1(PGE1)might support hepatic function or not after liver resection was examined. Materials were 34 patients who underwent liver resection inour department. Thirteen were given given PGE1 intravenously (PGE1(+)group) and 21 were not (PGE1(-)group). PGE1 was infused in a dose of 0.03μg/kg/minute during surgery and 0.01μg/kg/minute for 72 hours after surgery. The hepatic function was estimated by sGOT, sGPT, total bilirubin level and prothrombin time measured on postoperative day 0, 1, 3 and 7. To further evaluation, they were classified into 4 subgroups as follows: (1)non-cirrhotic PGE1(+) group, (2)cirrhotic PGE1(+) group, (3)non-cirrhotic PGE1(-) group and (4)cirrhotic PGE1(-) group. There were 17 patients with liver cirrhosis. Patients' characteristics were similar among each group. The sGOT and sGPT levels on postoperative day 3 were lower in the PGE1(+) group. The sGOT levels on postoperative day 1, 3 and the sGPT level on postoperative day 3 were lower in the non-cirrhotic PGE1(+) group than the non-cirrhotic PGE1 (-) group. In the cirrhotic PGE1(+) group, the total bilirubin levels were lower than the cirrhotic PGE1(-) group. Particularyly, there was a significant difference on postoperative day 1 ($p<0.05$).. It was concluded that continuous intravenous administration of PGE1 seems to be benefical for the liver function after hepatic resection.

### P305

### LIVER FUNCTION AFTER RESECTION FOR HCC ON CIRRHOSIS

BRESADOLA F., UZZAU A., CARCOFORO P., INTINI S., MICHELIZZA T.
General Surgery, University of Udine - Italy

Following pre-surgical volumetric measurements (Okamoto 1983), cirrhotic patients were monitored for evidence that liver resection induces early post-operative functional deficits. Subjects were 44 patients of whom a) 13 with HCC (84.6% cirrhotic), b) 21 with metastases and c) 10 with benign lesions. In surgical cirrhotic patients Okamoto's index was less than 50%. Following surgery conditions were monitored by haematochemical tests on post-operative days 1, 3, 5, and 7. Results were compared by Student's t-test. The 3 groups of patients were homogeneous for age, duration of anaesthesia, blood and plasma transfused during surgery, and post-operative diet. Significant differences were observed between metastases and benign lesions vs. HCC in the values of gammaGT, bilirubinaemia, glycaemia, cholesterolaemia, albuminaemia, transferrinaemia and fibrinogenaemia. Results were interpreted as indicating decreaseed protidosynthetic and glucuronic activities, reduced insulin response and decreased proteins of the flogistic response, and alteration of the lipidic metabolism in cirrhotic vs, healthy livers.

### P306

### STIMULATING EFFECT OF THE EXTRACT OF SMALL INTESTINAL MUCOSA ON RAT LIVER REGENERATION

1) A.Nakano, 1)T.Fukushima, 1) H. Sekido, 1)S Fukazawa, 1) U.Fujii 1) T.Fukushima, 2)N.Takashashi
1)Second Department fo Surgery, Yokohama CIty University, School of Medicine, Yokohama Jam=pan

2)Division of Agricultural chemistry, School of Agriculture, Meiji University.

Many studies have been done on the hypothesis that factors present in blood of the portal vein greatly participate in liver regeneration. We hypothesized that there are liver regenerating factors in the epithelial mucosa of the small intestine, the present studies were carried out whether extracts of the intestinal mucosa can stimulate the live regeneration in vitro and in vivo.

MATERIAL AND METHOD: I) 1.Preparation of the extract of the intestinal mucosa: Wistar adult rats were sacrified and the epithelial mucosa of intestine was separated. The specimens were homogenized, ultracentrifuged and heated at 100° c.2.Isolated hepatocyte: Primary hepatocyte cell culture was made by the modified method of Seglen. William's medium is suitable as a basic medium. 3. Cell culture: Rat hepatocytes were incubated in dishes. Extract of intestinal mucosa was added to the standard medium (5%). Numbers of cells and enzyme activities in the medium were measured. II) 2/3 intestinal resection and hepatectomy was carried out and anastomosed by interrupted suture. After operation extract of intestinal mucosa was injected intrapeitonelly every day for 4 days. We measured regerated rate of liver by histochemical method by using BrdU monoclonal antibody.

RESULTS: Hepatocytes developed to form the functional micro-liver by the action of small intestinal mucosa in the media. After intestinal resection, hepatic regeration was improved if the extract of intestinal mucosa was injected.

CONCLUSION: Studies of effect of intestinal mucosa may provide new insights into mechanisms of liver regeneration.

### P307

### BILIARY ANASTOMOSIS FOLLOWING LIVER TRANSPLANTATION DOES NOT BENEFIT FROM T TUBE SPLINTAGE

K. J. DAWSON, J. R. NOVELL, A.K. BURROUGHS, K. ROLLES
Liver Transplantation Group, The Royal Free Hospital School of Medicine, Hampstead, London, United Kingdom

Different techniques of biliary anastomosis were assessed in 90 consecutive liver transplants, performed by one surgeon. In 80 patients a direct end-to-end anastomosis was fashioned between donor and recipient common bile ducts. The first 16 anastomoses were splinted with a T tube; no splint was employed in the remaining 64 patients. In two patients early in the series a biliary conduit was fashioned from the gallbladder, and in seven patients biliary drainage was via a Roux-en-Y jejunal loop. One patient succumbed to pulmonary hypertension during surgery and no anastomosis was performed. There were no biliary leaks from the two conduits or from the six jejunal loops. Three of 16 patients with splinted duct-to-duct anastomoses sustained leakage, requiring Roux loop conversion in two and removal of the T tube in one. Seven of 64 unsplinted anastomoses leaked, necessitating Roux loop conversion in 6 and percutaneous drainage in the remaining patient. Biliary leakage was invariably due to donor duct necrosis. Three strictures occurred following duct-to-duct anastomosis without t-tube drainage. All were successfully managed by endoscopic dilatation. There was no significant difference in leak rate between splinted and unsplinted anastomoses. The groups were well matched for age, sex and indications for transplantation. It is concluded that T tube splintage confers no advantage in direct duct-to-duct anastomosis following liver transplantation.

### P308

### BLOOD TRANSFUSION REQUIREMENTS IN LIVER TRANSPLANTATION

K. DAWSON, O. SMITH, G. HAZLEHURST, B. BROZOVIC, A. BURROUGHS, S. MALLETT, A. MEHTA, K. ROLLES
Royal Free Hospital and School of Medicine, London NW3 2QG, United Kingdom

Orthotopic liver transplantation (OLT) has become an established therapeutic option in the treatment of end-stage liver disease resulting from a wide variety of congenital and acquired disordrss. However, it does impose sustantial logistical and financial burdens on the transfusion service. Clinical features predicting greater blood product requirments include: (i) cirrhosis; (ii) ascites; (iii) coagulation abnormalities; (iv) previous upper abdominal surgery.

High blood product requirments have been associated with significantly increased mortality rates. The purpose of this study was to evaluate the haematological support needed to support a liver transplant programme and to determine if intraoperative aprotinin (an inhibitor of fibrinolysis) administration had a significant impact on blood product usage in liver transplantation. Ninety consecutive patients underwent OLT performed by the same surgeon using a standard anaesthetic procedure. The blood products monitored were red cells, fresh frozen plasma and platelets. Our data suggest that the use of aprotinin significantly reduces blood component usage for OLT ($p<0.05$). High risk cases, in terms of potential blood loss, have their risk reduced to normal levels by the use of aprotinin. In addition both ITU stay ($p<0.05$) and the time taken to achieve operative haemostasis ($p<0.05$) were significantly reduced.

P309

### QUANTITATIVE VOLUMETRIC STUDY OF THE LIVER

L.F. FRIGERIO, C.SPREAFICO, A.M ARCHIANO', G.COLELLA, C. SEGURA, M.L. TATONETT, F.ZUCCHI AND B.DAMASCELLI

All patients undergoing liver surgery are studied to assess the exact staging of the disease. All imaging techniques now available (US, CT, CT-LUF, CT-AP) are applied for determining the number, site, diameter and vascular relationship of the tumor and its satellite nodules. When performing a CT-Arterial Portography (CT-AP), we usually evaluated the hepatic volume of the whole liver and of the tumor nodules. This procedure is very important in liver transplantation as it allows an optimization for using the donors'organ and in hepatic surgery as it is likely to correlate the percentage of resceted non neoplastic parenchima to the pre-surgical functional tests so to give a predictive index of the remaining liver function and the rigenerative index. In all cases of liver transplantation we compared our measurements with the actual weight of the explanted liver which was provided by the anatomo-pathologist.

P310

### CT-ARTERIAL PORTOGRAPHY FOR STAGING THE HEPATOCELLULAR CARCINOMA IN PATIENTS UNDERGOING LIVER TRANSPLANTATION: ANATOMO-RADIOLOGICAL CORRELATION

C.SPREAFICO, L F FRIGERIO, A. MARCHIANO' S.ANDREOLA, F. GARBAGNATI, V.MAZZAFERRO, M.L. TATONETTI, AND B. DAMASCELLI
Special Radiologic Procedures Dept. - Istituto Nazionale Tumori - 1, v. G. Venezian - 20133 Milano - Italy

All patients scheduled for liver transplantation are avaluated by a complete radiologic study of the liver (US, CT, DSA and CT-AP). In particular, before the angiographic study the catheter is postitioned into the superior mesenteric artery and a CT study is carried out by opacifying the liver through the portal system. This method allows a higher sensitivity in detecting both the primary tumor and its satellite nodules. The site, number and diameter of the lesions as well as their relationship with the venous system (both portal and suprahepatic) are compared with the anatomo-pathologic findings obtained on the explanted liver.

The work is in progress and comparison and statistical evaluation of the results will be available later on.

P311

### TREATMENT OF PRIMARY GRAFT NON FUNCTION AFTER OLT WITH PROSTAGLANDINS $PGE_1$

G. L. GRAZI, E. JOVINE, G. F. STEFANINI, C. SAMA, A. MAZZIOTTI, G. GOZZETTI
Clinica Chirurgica 2°, University of Bologna, Bologna, Italy

Primary liver graft non function (PNF) remains one of the most life-threatening complications after orthotopic transplantation (OLT).

PATIENTS AND METHODS: Over the 96 OLTs performed at our institution, diagnosis of PNF has been assessed in 5 cases. In the latter three cases PNF has been treated with Prostaglandins $PGE_1$ (Prostin VR, Upjohn, Puurs, Belgium). Two of these OLTs were carried out without major technical problems and blood losses were minimal, whereas in the third a subcapsular hematoma complicated surgery. Liver enzymes and bilirubin suddenly increased in all the three cases, with a parallel fall of the coagulative profile. No other morphological failures could be identificated. $PGE_1$ were started at 10mg/hr and increased by 10μg/hr until a maximum does of 0.6 μg/Kg/hr was achieved. No cardiovascular symptoms appeared. In all pts a marked improvement in liver enzymes was observed after a 12 hours infusion. $PGE_1$ were discontinued after 6, 8 and 10 days respectively. All the pts recovered from PNF and the first two were eventually discharged: they are currently alive after 11 and 7 months from OLT respectively.

CONCLUSIONS: In the absence of a well-defined alternative for the therapy of PNF, the infusion of $PGE_1$ should be started as a first-aid treatment during the waiting period before retransplantation.

P312

### SEGMENTAL LIVING LIVER TRANSPLANTATION

MEHMET HABERAL, NEBIL BÜYÜKPAMUKCU, HASAN TELATAR, NEVZAT BILGIN, GÜLNAZ ARSLAN, MUALLA KARAMEHMETOGLU
Transplantation unit,Transplantation and Burn Foundation Hospital, Ankara, TURKEY

In order to overcome the highly critical shortage of cadaveric liver organs that has been the cause of death of 50% of the chronic liver disease patients on our Center's waiting list, who were unable to wait out the long term for receiving a cadaver liver, we have turned to another alternative, namely our technique of "Semental Living Liver Transplatation (tx)."

MATERIAL AND METHOD: Our technique of "Segmental Living Liver Tx." differs from the technique of segmental living related liver tx. performed by other teams, due to the fact that dissection of the left hepatic duct is perments II, III, the falciform ligament, together with 2 to 3cm. of tissue from segment IV. Finally, without any means of alteration or interruption in the graft and remaining liver, the following implantion of the falciform ligament is sutured to the recipient's falciform ligament preventing rotation of the implanted segements-as in trisegmentectomy.

RESULTS: From Dec.9, 1988 to May 9,1991, 7" segmental living liver tx. "has been performed at our Center. Of these recipients. 5 were adults with the mean age of 32.8 years and 2 were children with the mean age of 10.5 years. The donors were 2 fathers, 1 sister, 1 husband and 1 wife (mean age 41.9 years). One of the adult recipients has given birth to a completely healthy baby. Both the mother and the baby are doing well with normal liver functions. The remaining donors are also doing well with normal liver functions.

CONCLUSION: Our method of "segmental living liver tx."has shown to be completely harmless for both the donor and the recipient. It has no side effects upon the donor during or after hepatectomy.

**P313**

### THE EFFECT OF THE SIZE OF THE DONOR LIVER ON THE REGENERATIVE RESPONSE AFTER LIVER TRANSPLANTATION.

G. MAHLATI, D.KAHN, MARILYN TYLER, ZOE LOTZ, SUE BIRD, ROSEMARY HICKMAN.
Department of Surgery & Medical Research Council LIver Research Centre, University of Cape Town

The transplanted liver is known to undergo hepatic regeneration in response to the various injuries which occur in the perioperative period. In this study we investigated the effect of the size of the donor liver on the regenerative response after liver transplantation.

Large White X Landrace pigs were subjected to orthotopic liver transplantation using different sized donor and recipient animals as follows: Group I - donar larger than recipient; Group II - donor and recipient similar in size; Group III - donor smaller than the recipient. Liver biopsies were taken pretransplant and at 6,18,24,48,72 and 96 hours after the transplant and used to determine thymidine kinase activity, ornithine decarboxylase activity and mitotic index.

Using the above indices of hepatic regeneration, there was no regenerative response when a large donor liver was transplanted into a smaller recipient and a small response when the donor and recipient were of similar size. A significant regenerative response was noted in the livers transplanted from a small donor into a large recipient.

These studies show that the size of the donor liver does modify the regenerative response in the transplanted liver. These findings may have important implications in the matching of the donor and rcipient in clinical liver transplantation.

**P314**

### OUR EXPERIENCE IN LIVER RE-TRANSPALANTATION (RE-TX)

MORENO G.E.; GOMEX S.R.; LOINAZ S.C.; MAFFETTONE V.; GARCIA G. I.; GONZALEZ-PINTO I.; JIMENEZ R.C.; PALOMO S. J.C.; PA;MA C.F.; MARCELLO F.M.
Digestive Surgery Department. Hospital 12 de Octubre. Madrid. Spain

We present our experience on liver RE-TX during 5 years from starting of the program. The poor clinical condition of the patients and the need of urgent procedure can be a very serious medical and surgical problem.

Between 1986 and November 1991 we hav eperformed 238 orthotopic liver transplantation (OLT) in 193 patients. In 37 patients we carried out 45 RE-TX (2 RE-TX in 7 patients and 3RE-TX in one). The indications for RE-TX were: chronic rejection (22), primary graft failure (10), hepatic artery thrombosis (6), acute rejection (2), hepatitis and/or cirrhosis recurrence (4( and acute massive hepatic necrosis (1) The mean time for RE-TX was: chronic rejection 11.2 ± 9.54 months, primary graft failure 6.5 ± 9.7 days, hepatic arterial thrombosis 3.1 ± 3.2 months, acute rejection 32 ± 16 days, hepatitis and/or cirrhosis recurrence 15.3 ± 5.6 months and acute massive hepatic necrosis 45 days.

The overall mortality was 32.4% (12 Patients); 8 patients (21.6%) of them died in per-operative period (6.8 ± 7.3 days) and the causes were; sepsis (4 pts), acute pancreatitis (1pt), brain death (2 pts) and 1 for haemorrhage from partial graft. The others 4 patients (13.7%) died at the follow up (5.5 ± 3.3 months) from sepsis (2 pts). The actuarial survival rate at 1, 3 and 4 years is 88.2%, 80.2% and 80.2% respectively.

RE-TX is sometimes an unavoilable rescue procedure for liver transplant patients with irreversible graft failure.

**P315**

### RESULTS OF ORTHOTOPIC LIVER TRANSPLANTATION IN FULMINANT HEPATIC FAILURE

MORENO G.E.; GARCIA G.I.; LOINAZ S.C.; GOMEZ S.R.; GONP.; RIAÑO C.D.; DAVILA M.P.; VERCEDO M.J.
Digestive Surgery Department. Hospital 12 de Octubre. Madrid. Spain

Twenty-four orthotopic liver transplants were done on fulminant hepatic failure (FHF), among 232 liver transplants performed from April 1986 to September 1991.

MATERIAL AND METHODS

Age limits were 3 to 60 (average 31.5). The ethiology of FHF was unknown in 19; HBsAg+ in 4 and toxicity of isoniazide in the last. Blood group (donor/recipient) were identical in 15, compatible in 6 and incompatible in 3. The graft was total liver in 18 and "reduced liver" (left lobe) in 6 (one case of split liver). 5 patients received a second graft and 2 a third graft.

RESULTS

Mortality: Four patinets died during the 30 days postoperative period (16%). Seven patients received a second graft (chronic rejection, 3 patients; primary nonfunction, 2 patients; cirrhosis, one;' arterial thrombosis, one). Two patients of this group need a third graft for chronic rejection and postnecrotic cirrhosis.

Follow-up: Four patients died between 4 months to 19 months as result of multiorgan failure, pneumoniae and subacute neumoniae.

Survival: Ten patients are still living between 3 months to 4 years. From the 7 retransplanted patients, three survived between 2.5 to 19 months, and three are surviving between 22 months to three years.

**P316**

### FINE NEEDLE ASPIRATION CYTOLOGY IN THE MONITORING OF LIVER ALLOGRAFTS IN THE PIG

M.F. NIELSEN[1,2], M.HOKLAND[2], M.H. SHOKOUH-AMIRI[1], L.S.JENSEN[1] P.WARA1AND S.L. JENSEN[1]
[1]Department of sugery L, Transplant Unit, Aarhus University Hospital, Section ÅKH, [2]Institute of Medical Microbiology, Department of Immunology, University of Aarhus, Aarhus Denmark

The diagnosis of acute liver allograft refuction is difficult to establish, as clinical signs and biochemical findings of liver dysfunction are rather nonspecific. The diagnosis of allograft rejection is mainly based on needle biopsy histology. However, the core needle biopsy (NB) may be associated with complications such as bleedings or infections. Recently fine needle aspiration cytology has been applied for monitoring liver transplants and it has proven useful for the diagnosis of acute liver allograft rejection. In the present study, fine needle aspiration biopsy(FNAB) was performed in pigs undergoing orthotopic liver allotransplantation and the results compared with histology in specimens obtained concomitantly by NB. Special emphasis was focused on cholestasis, fatty changes, necrosis, glycogen and RNA synthesis as well as on the relation between inflammatory cell pattern in FNAB specimens and the severity of inflammatory cell infiltration on cryostat sections using the indirect immunoperoxidase assay. Our results indicate onset of acute cellular rejection on the 4th day agter transplantation with lymphoid blasts and lymphocyte infiltration, later accompanied by monocytes and macrophages. Maximal intensity of inflammation detected as total corrected increment values was recored in FNAB on day 10 to 14 after transplantation. Histological evaluation of the graft based on NB specimen demonstrated distincr inflammation in the portal area on day 4 eventually followed by mononuclear cell infiltration of the intralobular parenchyma. With the indirect immunoperoxidase, technique, lymphoid cell subpopulation analysis of FNAB and NB demonstrated an increase of both T Helper/inducer, T suppressor/cytotoxic and B lymphocytes during actue cellular rejection. In additon, an increase of class II and IL-2 positive lymphoid cells could be detected in the graft during rejection episodes. Thus, we conslude that FNAB is a safe, reliable and atraumatic method for detecting early episodes of acute liver allograft rejection without any risk to the graft or the graft recipient, However, FNAB gives no information about the degree of vascular rejection and when additional information about parenchymal degeneration and location of pathological processes in needed, NB has to be applied.

**P317**

### HISTIDINE-TRYPTOPHAN-KETOGLUTRATE (HTK) PRESERVATION SOLUTION IN PORCINE LIVER TRANSPLANTATION. INFLUENCE ON THE HEPATIC CIRCULATION

K.J. OLDHAFER, G GUBERNATIS, J. HAUSS, R. PICHLMAYR
Hannover Medical School, Department of Surgery, F.R.G.

The HTK solution has been successfully used for kidney preservation. We have now investigated the HTK solution in experimental liver transplantation. The purpose of the investigation was to study the hepatic blood flow and vascular resistance in HTK stored livers.

METHODS; Orthotopic liver transplantation has been performed in 12 pigs. The cold HTK solution was flushed simultaneously via aorta and portal vein for 8 min. The cold ischemic tiem was 5.9± 0.48 hours. Hepatic arterial and portal venous flows wedre measured with an electromagnetic flowmeter (Cliniflow II, Model FM701D) in the donor and at least 1 hour after reperfusion in the recipient. Pressures were obtained directly form the portal and hepatic vein and arterially.

RESULTS: The flow of the hepatic artery was 143.0 ± 33.5 ml/min in the donor and 198.4 ± 68.2 ml/min after reperfusion. The arterial vascular resistance was 79.3 ± 22.9 Pa min/ml in the donor and 65.9 ± 19.8 Pa min/ml in the recipient. The flow of the portal vein was 490.5 ± 173.7 ml/min in the donor and 649.1 ± 185.3 ml/min after reperfusion. The vascular resistance was 2.04 ± 1.7 Pa min/ml in the donor and 1.62 ± 0.69 Pa min/ml in the recepient.

CONCLUSION: From histological analysis it is known that swelling of sinusoidal lining cells and hepatocytes after cold preservation and reperfusion is likely. So, the result that the vascular resistance after reperfusion did not increase in HTK stored livers was unexpected. It may be interpreted as a sign of an intact microcirculation. However, shunts bypassing the sinusoids or even the influence of denervation could not be excluded. This remains to be further clarified by simultaneous monitoring of the microcirculation.

**P318**

### PROSTAGLANDIN $E_1$ ($PGE_1$) THERAPY DURING ORTHOTOPIC LIVER TRANSPLANTATION (OLT) - PROTECTIVE EFFECTS ON PLATELET FUNCTION

H. RIESS, G. HIMMELREICH, K. HUNDT, G. BLUMHARDT, R, ROISSANT, P. NEUHAUS.
Departments of Internal Medicine, Surgery and Anaestesiology, University Hospital Rudolf Virchow, D-1000 Berlin 19.

We recently showed that in addition to the well-known drop in platelet count platelet function decreased after revascularisation fo the graft in OLT. This may be important for the development of bleeding complications in OLT. $PGE_1$ has been shown to have platelet protective effects in extracorporal circulations.

In an open, prospective and randomized study we studied the effect of a continuous PGE1-infusion on platelet count and platelet function in 10 vs.' 10 patients during OLT.

As compared with the control group $PGE_1$-infusion (10-40ug/h) resulted in a better maintenance of ex vivo platelet aggregability in response to ADP (1, 2 umol/l), ristocetin (1, 2 mg/ml) and collagen (0.5, 1 ug/ml). Furthermore $PGE_1$-infusion prevented the drop in platelet count observed in the controls after reperfusion.

Blood product requirements during OLT and in th first three postoperative days were comparable.

In conclusion, $PGE_1$ therapy during OLT preserves platelet function and prevents the drop in platelet count observed in controls after revascularisation. If these effects may result in less bleeding complications or less transfusion requirements has to be defined in clinical trials.

**P319**

### PROSTAGLANDIN $E_1$ ($PGE_1$) THERAPY DURING ORTHOTOPIC LIVER TRANSPLANTATION (OLT) - EFFECTS ON PLASMA HEMOSTASIS

G. HIMMELREICH, P. BREINDL, W.O. BECHSTEIN, R. ROISSANT, P. NEUHAUS, H. RIESS
Departments of Internal Medicine, Surgery and Anaestesiology, University Hospital Rudolf Virchow, D-1000 Berlin 19.

We and others showed that bleeding complications in OLT most likely are due to the deterioration of an already disturbed hemostasis by the influx of mediators released in the graft liver during reperfusion. $PGE_1$ has been shown to have beneficial effects in the treatment of ischemic injury of the liver.

In an open, prospective and randomized study we studied the effect of a continuous $PGE_1$- infusion on several parameters of hemostasis in 10 vs. 10 patients during OLT.

As compared with the control group $PGE_1$- infusion (10-40ug/h) resulted in an increase of t-PA-activity paralleled by more pronounced plasminogen and antiplasmin consumtions during the anhepatic phase. There were no differences in parameters of the intrinsic fibrinolytic system (scuPA, u-PA) nor in the incidence of hyperfibrinolysis in thrombelatography (0/10 vs. 0/10).

Parameters of the coagulation system (scuPA, u-PA) nor in the incidence of hyperfibrinolysis in thrombelatography (0/10 vs. 0/10). Parameters of the coagulation system (fibrinogen, fibrinmonomers, D-dimers, antithrombin III, protein C, and C1-inhibitor) did not differ between both groups.

Blood product requirements during OLT and in the first three postoperative days were comparable.

In conclusion, $PGE_1$ therapy during OLT results in minor changes in plasma parameters of hemostatis. The clinical role of $PGE_1$ in OLT remains to defined.

**P320**

### AUTOTRANSPLANTATION OF LIVER TISSUE IN ISLOATED SEGMENT OF SMALL INTESTINE IN PIG

M.H. SHOKOUH-AMIRI, A.O. GABER, L.W. GABER, M. BAYAT, S. RAHIMI-SABER, S. RUSTO, S.L. JENSEN, H. GREWAL, L.G. BRITT
Department of Surgery, Division of Transplantation, University of Tennessee, Memphis, USA, and Department of Surgery L, Aarhus Kommunehospital, Aarhus University Aarhus, Denmark

Liver trasnplantation is an excellent but expensive treatment for acute liver failure and end stage liver diseases. However, its use is limited by a donor shortage. In order to develop a bridging procedure model, especially in patients with acute fulminant liver failure, autotransplantation of sliced liver tissue in isolated segment of small bowel was performed in pigs. Six pigs were submitted to left hemihepatectomy. Twenty gr of the removed liver tissue to left were cut into slices of 2-3 mm thickness. A twenty cm long isolated segment of small intestine with intact circulations was prepared in the mid jejunum. Mucosal layer was removed and the liver slices were placed in this isloated segment of bowel evenly, and fixed with 5/0 abosrable sutures. Both ends of the bowel segment were closed and the whole segment was fixed to the nearby bowel. The animals were observed for six months. In one animal moderate regeneration of the implanted liver tissue was observed, whereas no regeneration was found in the other 5 animals. We conclude that implantatation of liver tissue in an isolated segment of small intestine does not result in a satisfactory regeneration of implanted tissue. Addition of some trophic agents such as pancreatic tissue may enchance the regeneration of liver tissue in this ectopic site.

**P321**

### CHANGES IN HEPATIC BLOOD FLOW AND PLASMA CATECHOLAMINES AFTER REDUCED LIVER TRANSPLANT

STAPLETON GN, METS B*, JANICKI P#, HICKMAN ROSEMARY.
Departments of Surgery, Pharmacology, Anaesthetics# and The MRC Liver Center, University of Cape Town.

Because hepatic blood flow has been inadequately studied after orthotopic liver transplantation (OLT) and not at all after reduced liver transplantation (RLT) which is now increasingly employed in paediatric OLT, where hepatic artery thrombosis is a common problem, blood flow in the Portal vein (PV) and Hepatic artery (HA) have been studied before, during and for 3 hours after experimental RLT under carefully controlled anaesthetic conditions, with measurement of plasma adrenalin (ad) and noradrenalin (NA).

METHODS: 6 pigs had RLT, 6 had autografts (AG) and 6 had sham operations. RLTs and AGs were performed with passive portosystemic bypass. Anaesthesia was induced with 750ng thiopentone i.v. and maintained with 2.5g thiopentone i.m. $N_20$ and $O_2$by volume cycle ventillator, and pancuronium bromide 4mg i.v. as required for muscle relaxation. Cardio-resporatory status was constantly monitored by intraarterial pressure readings and ECG and measurement of central venous pressure (CVP), pulmonary capillary wedge pressure (PCWP), cardiac output and arterial bolld gases at 13 predetermined stages of the experiment, when blood samples were also taken for catecholamine levels. At the same stages blood flow was measured in the HA and PV by Ultrasonic cuffs placed around the vessels and computed by a Transonic flowmeter.

SUMMARY OF RESULTS: Haemodynamic changes after RLT were similar to those after AG. Hepatic blood flow was decreased after both procedures compared with shams. Portal flow recovered quickly whereas HA flow took longer. There was, however, no difference in flow per gram of liver between autografts and RLTs. Noradrenalin remained significantly higher after revascularization of reduced grafts than autografts

CONCLUSION: Hepatic blood flow after RLT adapts to the same flow per gram of liver as the intact liver autograft.

**P322**

### THE BLOOD SUPPLY OF THE RIGHT AND LEFT HEPATIC DUCTS OF HUMAN LIVERS

G.N. STAPLETON, R.HICKMAN, J. TERBLANCHE
M.R.C. liver Research Centre, Department of Surgery, University of Cape Town, RSA.

AIM: To determine the anatomy of the blood supply of the right and left hepatic ducts to establish whether right or left hepatic lobes should be preferred for reduced liver grafts, which are now widely employed for treating end stage liver disease in paediatric patients.

METHODS: Livers were removed from human cadavers within 18 hours of death. Corrosion casts were made of the hepatic arterial and portal venous systems with Batson's no 17 corrosion compound. The casts were microscopically dissected to reveal the peribiliary plexuese (PBP). The dissections were sketched with the major arteries coloured differently. Vessels entering the PBP were coloured according to the main artery of origin. The diameters of all main arteries and branches to PBP were measured with a reflex microscope. Diameters of arteries feeding PBP from right and left hepatic and gastroduodenal arteries were summated and expresses as a ratio of the parent vessel diameter.

RESULTS:

9 casts were analysed

| | GDA | GDbr | RHA | RHAbr | LHA | LHAbr |
|---|---|---|---|---|---|---|
| Mean | 2.67 | 1.17 | 2.8 | 4.12 | 2.47 | 2.51 |
| SD | 0.63 | 0.89 | 0.99 | 1.47 | 0.62 | 1.66 |

Mean ratios of total branch diameter/main vessel diameter were: GD 0.42; RHA 1.5 and LHA 0.97. The segment IV artery, main suppler to the PBP on the left, arose from the RHA in 2 of the 9 casts.

CONCLUSIONS

Right hepatic artery was a more consistent supplier to the peribiliary plexus than the left hepatic or gastroduodenal arteries.

**P323**

### COLD PRESERVATION OF CANINE LIVER GRAFTS UNDER HYPERBARIC OXYGEN OR COMPLETE LACK OF OXYGEN.

T.M. VAN GULIK, C.R. NOI, A.J. VAN DER KLEIJ, D.J. BAKKER, P.J. KLOPPER, M.N.VAN DER HEYDE. Dept. of Surgery, Academic Medical Centre, 9 Meibergdreed, 1105 AZ Amsterdam, The Netherlands

After in situ flush with cold UW-solution, canine donor livers were transferred to a pressure chamber and pressurized (1.5ATA) with either 100% oxygen or 100% nitrogen. Following 24h or 48h of cold storge (CS) (4°C), the liver was orthotopically transplanted. Graft function was assessed by peak SGOT values and survival of the dog. The results were compared with CS under room air and normal ambient pressure (Reference group 24hrs CS; mean peakSGOT 2463 u U/I (SEM 897,n=4) 48 hrs CS: mean peakSGOT 4575 U/I(SEM781,n=6); Survival >6 days).

| Dog | Storge | Gas /pressure | Peak | SGOT | Survival |
|---|---|---|---|---|---|
| 1 | 24hours | 100% 02 /1.5ATA | 1584 | U/I | 6 days |
| 2 | | | 3718 | | 1 |
| 3 | 48hours | 100% 02 /1.0ATA | 3566 | | 9 |
| 4 | | 100% 02 /1.5ATA | 5615 | | 13 |
| 5 | | | 5720 | | 9 |
| 6 | | 100% N2 /1.5ATA | 5000 | | 8 |
| 7 | | | 1960 | | 8 |
| 8 | | | 3234 | | 6 |

Increased oxygen concentration (100% 02, 1-1.5ATA) did not result in better preserved livers, up to 48 hrs CS. Livers which were deprived of oxygen by saturation with 100% nitrogen (1.5ATA) also showed life-supporting function with peak enzyme values comparable with the reference group (48hrs CS). It can be concluded that neither the lack of oxygen of its excess (up to 100% 02, 1.5ATA0, does act upon the quality of preservation or the injury sustained on reperfusion of liver grafts, in terms of functional outcome after transplantation.

**P324**

### IMMUNOSUPPRESSION AND LYMPHOPROLIFERATIVE DISORDERS (PTLD) AFTER LIVER TRANSPLANTATION

Z.YILMAZ, V.McALISTER, A. ROY, D.R.GRANT, C.GHENT, W.J.WALL
Multi-organ transplant unit, University Hospital, London, Ontario, Canada. N6A 5A5.

The price of powerful immunosuppression has been an inceasing incidence of sepsis and post transplant malignancy. We reviewed our esperience since 1977 to document the incidence of PTLD after liver transplantation and possibly changing risk factors. Prior to 1988, immunosuppression was induced with anti-lymphocyte glogulin(ALG); subsequently, OKT3 was used. All patients after 1980 received cyclosporine.

Ten of 330 patients developed PTLD; four are alive 2-65 months after diagnosis. Patients who developed Ptld received OKT3, ALG or high does steroid for longer, 18 days (+/-7.6), than those who did not, 6 days (+/-5.3) P<0.01. Similar total doses of ALG, OKT3, steroid and azathioprine were used. No differences were seen in age, sex or preoperative diagnosis. Pre and postoperative. Epstein Barr virus IgG titres were the same in both groups.

We conclude that it is not the type of profoundly immunosuppressive agent but the length of time for which it is used that is the risk factor in the development of PTLD.

**P325**

### LIVER GROWTH, BLOOD FLOW AND FUNCTION AFTER PARTIAL ORTHOTOPIC TRANSPLANTATION IN THE RAT.

E.ZOUCAS, A FOSS, R ANDERSSON, B AHRÉN & S BENGMARK
Department of Surgery, Univ Hosp, S-221 85 Lund, Sweden

Orthotopic liver transplantation after 34% resection of the total liver weight was performed in Sprague-Dawley rats. A microsurgical technique for the end-to-end anastomosis of the supra-, infra-hepatic vena cava and the portal vein was used. The transplanted liver was fully arterialized. Untreated animals, as well as liver resected (34%) rats were used as controls. All animals were followed up for 28 days after operation. Liver growth was studied by repeated measurements of the organ, using Vomprterized Tomo-graphy (CT0. Blood flow was measured by Laser doppler flowmeter immediately after operation, as well as four weeks later. Liver function was assayed by Protrombin-Time (PT) and by the antipyrine clearance test. Tests were obtained 3,7,14 an 22 days after organ uptake of $I^{131}$-labelled E. coli bacteria.

All animals survived 34% liver resection. In the transplanted group survival rate was 60%. There was a significant increase in transplant weight and volume compared to the weight of the resected liver post operatively. Liver blood flow was increased immediately after liver resection and after liver transplantation compared to untreated controls. Upon sacrifice a significant increase of blood flow was observed in the median lobe, while blood flow to the caudate lobe was decreased. P{T and antipyrine clearance were decreased 7 and 14 days after transplantation. RES function was similar in all groups.

In conclusion, partial liver transplantation with arterialization of the graft may be used for the study of liver transplantation in the rat. Using microsurgical techniques, blood flow was readily resrtored in the graft. Growth in the volume of the transplant was evident, as early as 3 days after was superssed up to 14 days after transplantation. RES function of the transplant was intact.

**P326**

### LIVER TRANSPLANTATION IN THE ELDERLY

K.-W.JAUCH, E. PRATSCHKE, M. STANGL, M. ANTHUBER, F.W. SCHILDBERG, J. GROH, G. PAPE
Liver Transplant Group, Clinic GroBhadern, Munich, FRG

LTx has become a standard procedure in end stage liver disease and usually upper age limit is set by 60 years. We compared the outlook in our first 125 Ltx patients since 1985 over 60 years (n=16) with the younger patients below 59 years (N=85).

In the elderly, liver tumours were responsible for nearly 50% of all indications in comparison to 25% in younger people. Postoperative complications didn't differ except primary Tx-failure which was observed in four patients (25%) of the elderly during the first three years when we didn't use a venous bypass. Length of ventilatory support and ICu-Stay wasn't different too. The long term survival was nearly identical with 62% vs 71% first-year survival in all indications.

In properly selected elderly patients LTx may be performed with the same success as in younger patients.

**P327**

### HEPATIC TRANSPLANTATION AS SEEN BY CT AND MRI

M McPHILLIPS, R, FEHR AND B. MARINCEK
Department of Medical Radiology, University Hospital, Zurich, Switzerland

A study was performed to assess the usefulness of computed tomography (CT) and magnetic resonance imaging (MRI) in diagnosis and management after liver transplantation.

We reviewed the CT scans and MR examinations of 24 patients. The imaging was done between 2 days and 28 months after transplantation.

In all patients a periportal and pericaval oedema was seen, due to disruption of lymphatic drainage. This resolves, sometimes very slowly. The evolution and resolution of post-operative complications such as peri-hepatic fluid collections, hepatic perfusion defects and haemorrhage of the right adrenal gland were documented. Several patients had biliary leaks, of which no evidence was seen on CT or MRI, confirming that these should not be the primary imaging modalities for biliary pathology. Two of our patients received livers from child donors and another a 'reduced-size' liver. Hepatic hypertrophy was visible as earlu as the seventh post-operative day.

Many of the complications seen post-transplantation will resolve, but monitoring with CT or MRI helps to confirm resolution or to reveal the need for intervention.

**P328**

### RIGHT LOBECTOMY FOR LIVER TRAUMA.

G.DE SENA, G.M. BUONANNO, G. AMATUCCI, P.FESTA, S.BORRIERO? F. CHIANESE, A. BRIGANTE, L. DE RIENZO, A. SANTA MARIA, L. CROCCO, L. MANSI, C. LOMBARDI, G. FIORDIENZI.
Department of Surgery - "G. Moscati" Hospital Avellino-Italy

The authors relate about a case of hepatic trauma found in a young man of fortyseven that, falling from a scaffolding, suffered a fracture of the right liver. The himely aid and the ready diagnosis by an hepatosplenic scintigraphy at Tc99 allowed the immediate operation that showed a massive haemoperitoneum . It was generated by a frontal fracture of the right liver involving the right hepatic vein and the pedicles of the anterior and posterior segments. The good reanimation happened before the operation and during it, that based itself prevalently on "Emagel" and blood transfusions, and Pringle's clampage that reduced partly the bleeding during the operation allowed the execution of a right lobectomy. The patient lived the hospital 24 days after. The very high mortality (50% Walt; 60% Blumgart et Defore) for extended hepatic resections for traumatic lesions (this operation is already very rareCalne 0,5-7%) justifies the interest in these cases crowned with success. Furthermore, Lorimer and Kraft report, respectively, a 75% and 82% mortality in the hepatic trauma with lesions of big vessels.

### P329

### THE MANAGEMENT OF TRAUMATIC HEPATIC INJURIES

D.M.K.S. KAULESAR SUKUL, E.J. JOHANNES, P.H. HARRBRINK
Department of Traumatology/general Surgery, University Hospital Rotterdam, Dijkzigt, 3015 GD Rotterdam, The Netherlands

To contribute to accurate diagnostics and treatment of patients with hepatic injuries, we reviewed the medical records of 120 consecutive patients, who were treated in our hospital, for traumatic hepatic lesions in the period of 1980 to 1991.

Of all patients the mechanism of the injury, the diagnostic methods, the associated injuries, the location of the hepatic injury, the presence of vascular and bile duct lesion, the treatment and the final results and complications are discussed.

The high mortality rate of patients with hepatic trarma, is in our opinion not due to the hepatic injury per se, but due to the severe concomitant injuries resulting from a high energy trauma, which could be confirmed in our analysis.

Early consideration of packing the liver might be advantageous in those cases where an anatomical restoration fo the liver proved to be impossible. It seems that the best results are achieved in the group where hemodynamic stability is obtained at an early stage.

Based on the literature and our own experience we designed an algorithm for the management of hepatic injuries.

### P330

### ULTRASOUND PERCUTANEOUS MANAGEMENT OF INTRA-ABDOMINAL BILOMAS

MEKKY F., SHEHAB W., IBRAHEM E., GAMAL M. SALEM M., KHAHIL MS.
Alexandria University, Alexandria, Egypt

In a two-years period, 13 bilomas developed in 10 patients. Seven of the patients had solitary biloma. While the other 3 patients had two bilomas. The major two causes, which equally shared in the formation of bilomas, were liver trauma and biliary tract procedures. Five bilomas were managed by US guided percutaneous repeated needle aspiration, while 8 bilomas were managed by US guided percutaneous, pig-tail 8-french, catheter drainage. Culture was positive for organisms in 9 patients, of them was E.coli. Amikacin was the most sensitive antibiotic to be used. Cure was achieved in 7 patients with a mean cure time of 18.2 ± 3.4 days, while partial cure was achieved in the other 3 patients. No failure, mortalities or morbidities were met with among these patients.

### P331

### EXPERIMENTAL STUDY ON ENERGY METABOLISM OF ISCHAEMIC LIVER WITH OBSTRUCTIVE JAUNDICE USING $^{31}$P-MRS

AKIYAMA, T[1)]., KINAMI, Y[1,2)]., KOJIMA, Y[1)]., KITA, I[1)]., TAKASHIMA, S[1)]
The Second Department of Surgery [1)]., Division of Cancer Research, Medical Research Institute [2)] . Kanazawa Medical University, Ishikawa, Japan

Energy metabolism of ischemic liver with obstructive jaundice was investigated in rats using $^{31}$ P-MRS. In the jaundice group the common bile duct was artificially obstructed for 3 weeks, while in the control group sham operation was performed. In both group hepatic artery and portal vein were clamped for 15 minutes, and $^{31}$ P-MRS was werially investigated before clamp, during clamp, and 10, 30, 60, 120 minutes after declamp, then B-ATP/Pi was examined. In the control group the value of B-ATP/Pi before clamp was 0.848±0.143 (mean ± SD), and then during clamp decrease to 0.133 ± 0.009. After declamp B-ATP/Pi was recovered rapidly as follows: 0.530±0.196 (10 minutes after declamp), 0.735±0.043 (120 minutes). On the other hand, in the jaundice group the value of B-ATP/Pi before clamp was 0.686±0.183, lower than the control group. Recovery after declamp was delayed compared with control group as follows: 0.228±0.070 (during clamp), 0.422±0.310 (10 minutes after clamps), 0.492±0.298 ( 30 minutes), 0.629±0.251 (60 minutes), 0.732±0.224 (120 minutes). From these results, it is suggested that on energy metabolism the hepatic cellular damage may be induced by liver ischemia in obstructive jaundice.

### P332

### THE INFLUENCE OF HEPATIC PERFUSION UPON THE SURVIVAL OF THE ISOLATED PERFUSED RAT BRAIN.

B ALEXANDER, M ASLAM1, IS BENJAMIN.
Dept. of Surgery, King's College, London, UK. Dept. of Surgery, Royal Postgraduate Medical School, London, UK.

The Pathogenesis of portal-systemic encephalopathy is complex and still remains poorly understood. The multifaxtorial nature of the diseases and the effect which other organs may have upon brain metabolism may complicate and perhaps overshadow malfunctioned subtle biochemical interrelationshipos which may exist between the liver and brain Geiger and Magnes (1947) first suggested that such an interrelationship could exist: this they concluded from a series of isolated perfused cat brain and liver perfusions which have never been repeated or confirmed. Modern technological developments enabled us to construct an isolated rat brain perfusion circuit which could also accommodate an isolated perfused rat liver perfused concomitantly. Brains which were perfused at a physiological perfusion pressure of 120 mmhg (aflow rate of 2.5-3.0 ml/min) were shown to remain alive for a median of 35 minutes (range 22 to 53 minutes, n+12) as measured by the gradual reduction in spontaneous EEG activity to a background nose of less than 5hz. Concomitant perfusion with an isolated perfused rat liver, extended this survival time to a median of 210 (range 172 to 480 minutes). These results suggest the liver may produce a substance or substances normally required for brain homeostasis.

Geiger A., Magnes M (1947:): Am J Phusiol 149: 517-537

### P333

### THE EFFECT OF PORTAL HYPERTENSION ON BACTERIALTRANSLOCTAION IN THE RAT

R.ANDERSOON, X.D. WANG, V. SOLTESZ*, S. BENGMARK
Departments of Surgery and Midical Microbiology*, Lund, Sweden

Intraabdominal infectious complications are frequently encountered following major liver resection, In pervious experimental studies we have demonstrated an increased incidence in enteric bacterial translocation following major liver resection. The influence of alterations in portal venous pressure on bacterial tranlocation following major liver resection is, however, not known.

70 or 90% liver resections or portal venous ligation were performed in the rat. Mortality was encountered, as well as the incidence of bacterial translocation to mesenteric lymph nodes, portal and systemic blood and visceral organs (liver, spleen, kidneys, lungs) at various time-points after operation. Systemic arterial and portal bolld pressure and subserosal blood flow int eh intestine were determined.

The incidence of bacterial translocation significantly increased in animals with 90% hepatectomy. Portal pressure increase both in group treated with 90% hepatectomy and portalligation alone. Subserosal blood flow in the small intestine diminished in animals with both major liver resection and portal hypertension alone, while diminish in the colon was only seen in 90% hepatectomy.

We conclude that the increase in portal venous pressure per se following major liver resection could hardly be a major contributory factor explaining the increased incidence of bacterial translocation following major liver resection.

### P334

### THE INFLUENCE OF ACUTE LIVER FAILURE ON THE INTESTINE IN THE RAT

R. ANDERSSON, S.D. WANG, A. AR' RAJAB, V. SOLTESZ[1], WENQI WANG[2], M.L. SVENSSON[3], C. SVANBORG[3], S. BENGMARK
Departments of Surgery, Medical Microbiology[1], Internal Medicine[2] and Clinical Immunology[3], Lund, Sweden

Bacterial infections are frequently encountered following acute liver failure (ALF). We have previously demonstrated the occurrence of bacterial translocation in ALF. The influence on the intestine has, however, not been evaluated.

ALF was induced by 90% hepatectomy in the rat. Systemic and local portal circulation as well as intestinal microcirculation was evaluated at various timepoints following 90% hepatectomy. Changes in intestinal morphology was estimate by light microscopy and morphometric system. Mucosal mass, protein content, bile secretion and bacterial content and overgrowth was also determined.

90% hepatectomy resulted in an immediate decrease in systemic arterial blood pressure and an increase in portal venous prssure. Subserosal micro-circulation and small arterial circulation in proximal and distal small intestine and colon significantly diminished by time following 90% hepatectomy. Microvillous height significantly decreased from 1h and on, and villous height and area in the distal small intestine form 2 h after operation Small intestinal mucosal mass decreased 2 h after hepartecromy. Protein content in enterocytes and bile secretion from the liver remnant were markedly reduced in hepatectomized rats. Overgrowth and colonization of E.coli occured in the distal small intestine from 1h and on after hepatectomy.

In conclusion, the present study demonstrates intestinal alterations that can contribute in explaining the enteric bacterial translocation occuring in surgically induced acute liver failure.

### P335

### BACTERIAL TRANSLOCATION IN ACUTE LIVER FAILURE INDUCED BY 90% HEPATECTOMY IN THE RAT

R. ANDESSON, X.D. WANG, V SOLTESZ*, S. BENGMARK
Department of Surgery and Medical Microbiology*, Lund, Sweden

Bacterial infections and bacteremia are quite frequently observed following major liver resection and in patients with acute liver failure (ALF). The exact source of the bacteria, as well as the pathophysiological mechanisms explaining the development of infection remain unclear.

ALF was induce by 90% hepatectomy in the rat. Mesenteric lymph nodes (MLN), visceral organ, (liver, spleen, kidneys, lungs) and blood were aseptically harvested for bacteriological cultures before and at various time points after 90% hepatectomy. Liver function tests, arterial blood pressure as well as whole body and gut oxygen extraction were assayed. Reticuloendothelial system (RES) function was determined by blood clearance and organ localization of intravenously injected $^{125}$Ilabeled E. coli.

Translocation of enteric bacteria to MLN was evident 2 h postoperatively and significantly increased to the various organs and blood by time following hepatectomy. Overgrowth of E.coli in the distal intestine started 2 h postoperatively. RES function decreased immediately after 90% hepatectomy, although uptake rates per gram tissue in the various organs significantly increased. Also gut oxygen extraction decreased following hepatectomy.

In clnclusion, bacterial transloction occurred in the early phase after hepatectomy-induced ALF, associated with a decrease in RES function and gut oxygen extraction and an intestinal overgrowth of E.coli.

### P336

### PRIMARY LYMPHOMA OF THE LIVER TREATED WITH LEFT HEPATIC LOBECTOMY

C.W.BIERMANN; G. FRÖSCHLE; H. J. WHH; U.MEUER-PANNEITT; CH.E. BROELSCH
Department of Surgery and Oncology; The University of Hamburg; Germany

1976 Kim et al were the first, who described primary lymphomas in the liver. Until now 48 cases have been publicated in the international literatury. We report about a 71-years old patient who was diagnosed a spaceoccupy of the left liver lobe through routine ultrasound. The extensive diagnoses by DSA and Angio-CT confirmed a 5 x 5 cm solide space-occupy in the left liver lobe as well as kidney cysts right and a liver cysts in the right liver lobe. Except gamma-GT (62 U/l) and AP (107 U/l) all laboratory findings had normal value. An explorative laparotomy with left lobectomy and segmentectomy (VII) was done. Postoperative course with CHOP-cycles followed as well as regular staging. Two years after primary surgical thereapy no relapse was diagnosed and the patient is in good condition. Although the number of these patients are small, the data suggest aggressive surgical resection where possible. in conjunction with chemotherapy.

### P337

### CHARGES IN HEPATIC CIRCULATION IN CONSCIOUS DOGS: EFFECT OF GASTROINTESTINAL HORMONES

K INOUE, MKOGIRE, S SUMI, S HIGASHIDE, K TRAKAORE, K UCHIDA, T TOBE
First Department of Sugery, Faculty of Medicine, Kyoto university, Kyoto 606

Effects of vasoactive intestional peptide (VIP), Epidermal growth factor (EGF) and glucagon on hepatic circulation were studied in the conscious state.

Under pentobarbital anesthesia, probes of an ultrasonic transitime volume flowmeter (T201, Transonic System Inc)were applied on the portal vein and hopatic artery in beagle dogs. After seven says recovery, measurement of hopatic circulation in the conscious state was started. The basal blood flows of the portal bein and hopatic artery in the conscious were 345 ± 52ml/min and 59 ± 10ml/min, respectively (n=7). VIP,EGF and glucagon stimulated dose-dependent increases in in portal venous flow, showing the maximal invrement of 61 ± 10% (n=7), 61 ± 16% (n=5) and 61 ±16% (n=5), respectively.

Hepatic arterial blood flow was significantly increased, showing the maximal increment of 572 ± 158% (n=7) and 61 ± 14% (n=5), respectively, in response to VIP and EGF. Glucagon exerted no effect on hepatic arterial flow. This study suggests that fastrointestinal hormones may influence hepatic circulation throug different mechanisms.

### P338

### SANDOSTATIN PROTECTS AGAINST ENDOTOXIN-INDUCED LIVER AND KIDNEY NECROSIS AND THE IMPAIRMENT OF RENAL FUNCTION

S A JENKINS, H KYNASTON, N DAVIES, M O'DRISCOLL, J YATES, K PARSONS, AND B A TAYLOR
Departments of Surgery and Nuckear Medicine, Royal Liverpool Hospital, Liverpool

Previous studies have demonstrated that sandostation (SMS) ameliorates hepatic necrosis and renal tubular damage following E. Coli. endotoxin adminstration in rats. The aim of the present study was to establish whether the beneficial effects of SMS on the liver and kidney could be explained by alterations in blood flow to these two organs. Groups of male Wistar rats (n=10) received either intraperitoneal endotoxin (2mg/100g) or saline. A further group received SMS immediately prior to and 12 hours after adminstration of endotoxin. Hepatic and renal blood flow were measured by the microsphere method and renal function was assessed by plasma clearance of Mag 3, a renal tubular agent. SMS prevented the hepatic necrosis and renal tubular damage observed in rats treated with endotoxin alone. Cardiac output was significantly reduced in the two groups that received endotoxin ($p<0.001$, ANOVA). There was no significant difference in hepatic or renal blood flow between the groups. Mag 3 clearance was significantly reduced in the group that received endotoxin (0.76 ± 0.05ml/min/100g;mean ± SEM) compared to controls (1.11± 0.09ml/min/100g; mean ±SEM) and SMS treated animals (1.23±0.09ml/min/100g ; mean ± SEM). endotoxaemic hepatic necrosis and renal tubular damage appears to result from a direct effect on the liver and kidney rather than alterations in haemodynamics. Renal tubular function is protected by prior administration of SMS although the precise mode of action is unclear.

### P339

### EFFECT OF TOTAL INTRAVENOUS ANESTHESIA WITH PROPOFOL ON HEPATIC FUNCTION

E. KARAMICHALI, A. STOUMBOU, B. CHRISOVERGI, N. PAPARIZOU, E. KURGIOU.
Department of Anaesthesia. Evangelismos Hospital Athens, Greece.

There is a need to study the potential toxic effects of each new anaesthetic in circumstances similar to which they will be used in clinical practice. The aim of the present study was to demonstrate the influence of propofol on liver function.

Methods: Twenty patients physical status, I or II ASA, with normal liver function, scheduled for non haemorragic surgery whose duration was 91±4 min., were studied in tow groups. The groups were comparable with regard to age and weight. All patients received for induction of anaesthesia propofol single bolus injection 2.5 mg/kg. Maintenance of anaesthesia was with $N_2O$ 66% in $O_2$, supplemental A group received fentanyl 4μg/kg plus diazepam 0.15 - 0.2mg/kg and B group received continuous infusion of propofol at a rate 6-12 mg/kg/h plus fentanyl 4μg/kg. Muscle relaxant was pancuronium. All patients were premedicated with droperdiol 2.5mg and fentanyl 100μg. We selected to monitor liver function the following laboratory testings: transaminase (SGOT, SGPT), alkaline phosphatase (ALP) gamma glutamyl transferase (YGT), 5-nucleotidase, chloesterol, albumin (A), globulin (G), ratio A/G and total protein. The measurements were received direct before (control) and after the use of propofol at the 1st, 2nd, 3rd post operative days. The statistical analysis was performed by Student's test for paired or unpaired values. P values of less than 0.05 were considered significant.

Results: The incidence of changes was low and not significant in all measurements in two groups and was not statistically different between groups.

Discussion: It is important to establish that there is no evidence of liver dysfunction following exposure to new intravenous agent propofol. It has been shown that continuous intravenous anaesthesia may cause liver enzyme changes particularly with althesin and ketamine during operations of intermediate duration. Recently it is observed that the pharmacocinetics of propofol might be affected by the presence of cirrhosis when prolonged infusions are used. Our results demonstrate no adverse effects by using normal induction and continuous infusion dose of propofol and also indicate no significant changes in liver function.

### P340

### REDUCTION OF THE TOXICITY OF CONTINUOUS HEPATIC ARTERY INFUSION OF INTERLEUKIN-2 WITH CIRCADIAN PATTERNING

M.M.KEMENY, G.ALAVA, J.OLIVER, F.B.SMITH
St. Vincent's Hospital and Medical Centre, New York City, New York, USA

Toxicity with continuous infusions of IL-2 have limited the usefulness and efficacy of this therapy as an antineoplastic agent. A new approach to try to reduce the toxicities with circadian patterning of the hepatic artery infusions is presented in this study. Hepatic artery catheters were placed into Sprague Dawley and Fisher rats. Different cycles and doses of IL-2 were tried to examine the toxicity and tolerability. When a constant continuous infusion was used only a dose of 1mg/kg/d could be tolerated without mortality. The mean survival times for 2,5, and 10mg/kg/d were 14.2,7, and 4 days respectively. If the IL-2 was given in a "day cycle" which was a continuous infusion but with maximum flow from 3 PM to 9PM and minimum flow from 3AM to 9AM the animals tolerate up to 10mg/kg/d with no mortalities. If the IL-2 was given in a "night cycle" with maximum flow from 3AM to 9AM and minimum flow from 3PM to 9PM then the animals could tolerate doses as high as 5mg/kg/d. But at 10mg/kg/d all the animals died with a mean survival of 8.0 days. Circadian patterning with a "day cycle" allowed up to 10 times the doses of IL-s to be given with no mortality and a reduced pathologic liver toxicity.

### P341

### OXIDATION DAMAGE AND ACTIVITIES OF ANTIOXIDATIVE ENZYMES IN THE LIVER OF SENESCENCE ACCELERATED MOUSE

J.S. KIM*, S.T. KIM
Dept of Surgery, College of Medicine, Hallym Univ*., Seoul National Univ., Seoul, Korea

A role of oxygen free radical (OFR) has been suggested in aging process, but most evidences are limited in the studies of non-mammalian animals. So we used Senescence Accelerated Mouse (SAM-P) as a mammalian model, because this mouse ages earlier and has about two-thirds of life span of normal mouse (SAM-R), Degree of aging, oxidant damage, and the activities of OFR-scavenging enzymes were measured and compared in various organs of both groups (n=6, each group). RESULTS: A significant increase of fluorescent age pigment (FAP), a biochemical index of aging, was seen in the liver and the kidney of SAM-P. Conjugated dienes (CD), peroxidation products of membrane lipid, was also increased in the liver and the kidney of SAM-P. The contents of FAP and CD in the liver were highly correlated (r=0.0885). The activity of OFR-scavenging enzymes was measured in the liver of two groups of animals. The activity of superoxide dismutase (SOD) and glutathione reductase were markedly reduced in SAM-P, but no difference was observed in those of catalse and glutathine peroxidase. SOD in the mitochondrial fraction of the liver was assayed by the electrophoresis and indicated as Cu-Zn type. These date suggest that SAM-P appeared to be damaged more by OFR and the liver was damaged most prominently. Such higher degree of aging and oxidant damage may be related to decreased activity of some OFR-scavenging enzymes.

### P342

### HEPATIC PROTEIN SYNTHESIS AND CYTOKINES IN OBSTRUCTIVE JAUNDICE

F. KIMURA, M. MIYAZAKI*, T. SUWA, K. HAYASHIDA, S. KAKIZAKI
Department of Surgery, Omiya Red Cross Hospital, Yono-Shi, Saitama-Ken, Japan
*The First Department of Surgery, Chiba University School of Medicine, Chiba-shi, Chiba-Ken, Japan

Hepatic protein synthesis and cytokine activities were investigated in obstructive jaundice. The contents of the following in the peripheral blood were determined in 21 patients with obstructive jaundice before and two weeks after percutaneous transhepatic biliary drainage (PTBD): interleukin-1 (IL-1), interleukin-6 (IL-6), tumor necrosis factor (TNF),endotoxin (Et), acute-phase protein (APP)[$\alpha_1$-antitrypsin, $\alpha_1$-acid glycoprotein, and fibrinogen] and negative acute-phase protein (NAPP) [prealbumin and retinol binding protein]. The results: (1) IL-1 and Il-6 were significantly high ($p<0.05$) but was reduced after PTBD; no TNF was detected; (2) Et was not detected; (3) APP was significantly higher ($p<0.05$) but showed a tendency to decline after PTBD; (4) NAPP was significantly low ($p<0.01$) but the content was restored to a normal level after PTBD; (5) obstructive jaundice was associated with rised in the peripheral blood IL-1 and IL-6 levels, increased hepatic production of APP and reduced synthesis of NAPP.

### P343

### HAEMOBILIA: MANAGEMENT BY PERCUTANEOUS SELECTIVE HEPATIC AFTERY EMBOLIZATION

JEJ KRIGE, SJ BENINGFIELD*, J TERBLANCHE
Departments of Surgery, Radiology* and MRC Liver Research Centre, University of Cape town and Groote Schuur Hospital, Cape Town, South Africa

Fourteen patients (10 men, 4 women, mean age 36.6 years, range 18-36 years) with major haemobilia originating in either the liver (13) or gallbladder (1) were treated between 1977 and 1986. Bleeding was due to penetrating (4) or blunt (1) liver trauma, iatrogenic (3) [percutaneous transhepatic endoprosthetic stent placement (2) liver biopsy (1)], arteriovenous malformation (2) right hepatic artery aneurysm (2), pyogenic liver abscess (1) and chronic cholecystitis with gallstones (1). All patients had melaena, 5 had haematemesis and RUQ opain was present in 8 patients. Only 2 patients were jaundiced. Bleeding from the ampulla was identified in 2 patients during ERCP. Endoscopy identified fresh blood in the second part of the duodenum in 7 pf 10 occasions. A liver lesion was identified in 6 of 10 patients who underwent either CT scanning or liver ultrasound. Selective hepatic angiography demonstrated an intrahepatic bleeding source in 13 patients. An arteriobiliary fistula in the gallbladder in 1 patient was not identified by angiography. Selective hepatic arterial embolization using either gelfoam pledgets or Gianturco coils controlled bleeding in 10 of 12 patients. Embolization failed in 2 patients (1 with segmental liver necrosis required a right hepatic lobectomy and a second patient underwent surgery and ligation of the left hepatic artery). Bleeding from the gallbladder in 1 patient was treated by cholecystectomy. Selective hepatic artery embolization was not attempted in 1 patient who underwent a left hepatic lobectomy. Selective hepatic artery embolization was successful in 10 of 12 patients (83%) of whom 1 patient developed subsequent complications.

Selective hepatic artery embolization provides definitive control of liver bleeding with a low incidence of complications and should be considered the primary treatment of choice for intrahepatic haemobilia.

### P344

### EFFECTS OF THE UPPER ABDOMINAL OPERATION ON HEPATIC BLOOD FLOW

K.MIYATA, H.TANIGUCHI, Y.UESHIMA, K.TAKEUCHI, A.OGURO, N.TSUKUA and T.TAKAHASHI
First department of surgery, Kyoto prefectural university of medicine, Kyoto, Japan

The changes of hepatic blood flow during upper abdominal operation were measured. Twenty five patients who had upper abdominal operation were classified into the following three groups. Fourteen had subtotal gastrectomy with resional lymphnodes dissection (10males and 4 females, mean age: 62 years old), six underwent total gastrectomy with resional lymphnodes dissection (2 males and 4 females, mean : 61 y.o.), and five had cholecystectomy (3 males and 2 females, mean age : 51 y.o.). The hepatic blood flow was measured using laserdoppler blood flow meter (Peri Flux 3, Perimed Inc. Sweden), soon after laparotomy and organ resection. The changes of hepatic blood flow were compared among three groups, and correlations to distribution of age, duration of operation, blood loss, blood transfusion required, and the grade of dissection were tested. There were no correlations between changes of blood flow and range of age, duration of operation, blood loss, requirement of blood transinfusion, and the grade of dissection. Hepatic blood flow after total gastrectomy (mean 77.3%) is significantly decreased than after subtotal gastrectomy (112.7%) or after cholecystectomy (101.1%) ($p<0.05$). In conclusion, methods of operation were thought to affect hepatic blood flowduring the operation.

### P345

### UPPER GASTROINTESTINAL BLEEDING AS A COMPLICATION IN JAUNDICED PATIENTS

A. POLUCHRONIDES, C/ SIMOPOULOS, D. BAXEVANIDES, G. HANOS
Propedeutic Surgery Clinic, Democritus University of Thrace, Alexandroupolis - Greece

This is a retrospective clinical study which deals with upper gastrointestinal bleeding as postoperative operation in jaundiced patients.

This study concerns 80 patients (45 males - 35 females) with obstructive jaundice (hilirubin 3 gr.) who have been treated for the last five years (average 64,2 years) the cause of the jaundice was genign in 36 patients and malignant in 24 patients.

The patients received Vit. K ànd 35 of them prophylactic antibiotics.

Patients out of 71 (7,5%) developed hemorrage from the uppper g.i. in the early postoperative period.

### P346

### ARGON BEAM COAGULATION AND HEPATECTOMY IN THE PIG

R.R.POSTEMA, P.W.PLAISIER, F.J.W. TEN KATE*, O.T.TERPSTRA
The departments of General Surgery and Pathology*, University Hospital 'Dijkzigt', Rotterdam, The Netherlands

Background: Argon beam coagulation is a new device for hemostasis after surgery on parenchymatous organs. To conduct radiofrequency current to the tissue it uses the flow of argon gas while the conventional electrocoagulator uses air for electrofulguration. Little or no data is available on its efficacy and tissue effect after hepatic resection.

Methods: In 52 liver lobes of 13 pigs we assessed the blood loss, the time needed to achieve adequate hemostasis, the number of additional sutures and the histology after argon beam coagulation compared with suture ligation only, mattress sture technique and tissue glue application. The treatment was randomly assigned to any of the liver lobes in each pig.

Results: The blood loss after argon beam coagulation was 15 ml (median; range 2 - 47 ml) while after simple suture ligation this was 68 ml (2 - 260 ml, p<0.02). The time needed to achieve adequate hemostasis after the argon beam coagulation was 4 min. (2 - 7 ), (control group 18 min. (2 - 48), p<0.005). there was no difference in these parameters between argon beam coagulation and the tissue glue technique. Both were also superior to the use of mattress sutures. Argon beam coagulation resulted in less tissue reaction than the use of tissue glue or mattress suturing.

Conclusion: Argon beam coagulation is an efficient device to achieve hemostasis after partial liver resection in the pig.

### P347

### ALTERATIONS OF SERUM PROTEINS IN LIVER REGENERATION AFTER PARTIAL HEPATECTOMY IN RAT

AL.MARGELI, S.SKALTSAS, S. TEOCHARIS AND B.KEKIS
Department of Experimental Pharmacology, University of Athens

Department of Surgery. Athens Medical Centre, Athens.

In the rat, partial hepatectomy (PH) with removal of approximately 70% of the liver mass, induces a rapid cell proliferation in the remaining hepatic tissue. Male rats in groups of seven of the Quinster strain were partial hepatectomised. Liver regeneration was estimated at time intervals of 4,8,12,16,20,24,28,32,36,40,44,48,60 and 72 hours. The incorporation of $^{3}$H-thymidine into DNA was determined at the time intervals, as an index of liver proliferative activity. The Helena Serum Protein was intended by electrophoresis on cellulose acetate.

DNA synthesis presented a peak within 24 and 32 hours and a second one at 40 hours. Albumin values presented a decrease, more evident 20 to 72 hours, presenting a minimum at 48 hours. a1globulin was decreased during the time examined except of 48 and 60 hours, where the a2 globulin was increased. βand γ globulins decreased form 4 hours, presenting the lower values between 20 and 28 hours, while for β globulin another low value was observed also at 40 hours.

In Summary: the parameters examined, albumin al, a2, βand γ globulins presented more intense variations during the time intervals of the maximum liver regenarative process.

### P348

### THE INFLUENCE OF SURGICAL TREATMENT TO THE LIVER FUNCTION

K.TAKEUCHI, H.TANIGUCHI, Y.UESHIMA, A.OGURO, K. MIYATA , N. TSUKUDA, AND T.TALAHASHI
The First department university of medicine, Kyoto, Japan

Materials were 140 patients without any liver disease or severe postoperative complications like as DIC orMOF. They underwent operations under general anesthesia, excluding esophagectomy, liverresection or pancreatoduodenectomy. The values of AST, ALT and T-Bil on post-operative day 0,1,3,7 and 14 were measured. These patients were classified into following 3 group; A: patients with operation of the upper abdomen, B: patients withoperation of the lower abdomen, C: patients given operation without laparotomy. There is no significant difference among these groups in age, duration of operation, blood loss, duration of anesthesia or preoperative AST, ALT and T-Bil. On the 1st post-operative day, AST and T-bil elevated significantly in all groups, but ALT elevated significantly only in group A. The values of AST and ALT of patients in group A were significantly higher than those of patients in other groups. It is thought that this elevation is mainly due to the surgical treatment such as procedure to the liver by retractor or lymphnodes dissection which made a change of hepatic blood during and after operation. In conclusion, it can be said that the surgical treatment of upper abdomen is responsible for the liver disfunction in early post-operative days.

### P349

### SIGNIFICANCE OF ALTERED MEMBRANE FLUIDITY IN MECHANISM OF MULTIPLE ORGAN FAILURE

WANG BEN ZHANG, JUNJI TANAKA, MITSUAKI KOHMOTO, MASANORI YOSHIDA, TAKAYUKI KASAMATSU, JUNTAMURA, KEN-ICHI FUJITA
The First Department of Surgery, Kyoto University School Of Medicine

Multiple organ failure (MOF) is one of most important problems in surgical field. On the other hand, memebrane fluidity is recently suggested to play a major role on cellular function. The present study was aimed to investigate membrane fluidity in ccirrhotic and jaundiced patients as prerequisite MOF. Patients and methods: 28 cirrhotic, 8 jaundiced and 13 normal liver patients, a small pieace of livers were taken and membrande fraction was collected by gradient centrifgation. Membrane fluidity was determined by measuring fluorescence polarization (P-value) with DPH as probe dye. Results; Cirrhotic and jaundiced livers had higher P-values than that of normal (0.225±0.025, 0.231±0.020, $p<0.01$, compared to 0.189±0.017 of normal). Among them, 3 major complications and 5 operative death cases revealed, all of which P-values were higher than 0.225. In seperate experiment, serum from MOF patients was incubated with normal memebrances to investigate direct effect of the serum on memebrances. Normal serum induced an increase in integrated intensities, but without changes in P-values. In contrast, MOF serum induced a drastic decrease in integrated intensities with an increase in P-values. conclusion: The increased fluorescence polarization (decreased membrane fluidity) is indicative of risk factor for postoperative complications and MOF serum may have direct factor(s) on membrane fluidity.

### P350

### MORPHOPHYSIOLOGICAL CHANGES IN THE LIVER OF GUINEA-PIG FOR CHEMOTHERAPY OF EXPERIMETNAL TRICHINELLOSIS

T.K. RAISOV, M.R. MIZHANOV
Semipalatinsk's Medical Institue, Kazakhastan, U.S.S.R.

The treatmetn of trichinellosis is very difficult problem for our region. We have stutied the disturbens of the function of the liver in 32- guinea pigs with experimental trichinellosis by morphological, biochemical and electronic microscope methods.

The results of our experimental investigation has shown dystrophycal and inflammatory process in the liver ofguinea-pigs with trichinellosis. We have found out dystrophycal changes of hepatocytes.

We worked out combined chemotherapy by infusion mebendazol, mebendazol and voltazen or mebendazol with T-aktivin. It was very effective for treatment of trichinellossis. The aamelioration of the function of the liver was more marked after combined chemotherapy.

On the basis of this investigation we can say, thet combined chemotherapy is very effective and available for practice.

### P351

### VALUE OF DIAGNOSTIC PROCEDURES IN BUDD-CHIARI SYNDROME

LANG H.,K. OLDHAFER, B. RINGE, R. PICHLMAYR
Klinik fur Abdominal - und Transplantationschirurgie, Medizinische Hoschschule Hannover

The Budd-Chiari Syndrome (BCS0 is caused by occlusion of hepatic veins which otften leads to ascites, hepatomegaly and abdominal pain as initial clinical symptoms. Though BCS often appears to be a rapidly progressive disease there is sometimes a long period between first presentation of symptoms and final diagnosis. Clinical signs and laboratory findings are usually very unspecific and of little diagnostic value. To evaluate different diagnostic methods, a retrospective analysis of diagnostic procedures in patients with BCS was performed. Patients and Methods: Data of 30 patients (24 female, 6 male) with a mean age of 31 years (range 12-450 were analysed. In all aptients BCS was confirmed by pathology after liver transplantation. Diahnostic methods were: Cavography (n=22), hepatic venography (n=20), CT-scan (n=20), NMR (n=4), hepatic scintiscan (n=13) and ultrasound (n=29)

Results: Cavography showed stenosis of the retrohepatic inferior vena (IVC) in 16 cases. In two patients there was a complete thrombosis of the IVC, in four cases no pathologic result was found. Hepatic venography revealed thrombosis or obstruction of liver veins in all cases and in 20% a "spider-web". There was no false negative result in hepatic venography. CT-scan presented compression of the IVC (n=6), rarefication fo liver veins (n=4), a hypertrophic caudate lobe (n=7) and thrombosis of the IVC (n=3). NMR demonstrated stenosis of the IVC and obstruction of liver veins in all cases. Hepatic scintiscan revealed reduced arterial perfusion (n=11), inhomogeneous uptake (n=7) and focal accumulation fo tracer (n=2). Ultrasound showed obstruction of liver veins in 20, a hypertrophic caudate lobe in 16 and a stenosis of the IVC in 7 patients. In two cases ultrasound was not suggestive of BCS initially, but demonstrated obstruction of the hepatic outflow tract when repeated. Combination fo ultrasound, cavography and gepatic venography were suggestive of BCS in 29 patients.Catheterisation of the IVC and hepatic veins presented pathologic changes typical of BCS in 100%. In one case NMR lead to diagnosis.

Conclusions: Ultrasound, cavography and hepatic venography proved to be most important and reliable diagnostic procedures in detecting thrombiosis and obstruction fohepatic veins or the IVC, CT-scan and scintiscan seemed to be of minor diagnostic value, wheras NMR might be helpful in some cases.

### P352

### HEPATECTOMY WITH Nd: YAG LASER AN EXPERIMENTAL AND CLINICAL STUDY

XU MIN, WU ZAI-DE, CHEN XIO-PING
Department of General Surgery, Tongji Hospital , Tongji Medical University, WuHan, Hubei (430030) P.R.China

We conducted an experimental and clinical study of hepatectomy with Nd: YAG laser. The results showed that outing effect artificial sapphire contact Nd:YAG laser scalpel was better than that of quartz fiber contact and noncontact Nd:YAG laser. The tissue damage was minimal in the former group whereas, the blood loss was similar in both groups. Seven patients were operated on, with quartz fiber and 12 with artificial sapphire laser scalpel. No complications were related to the use of laser. we consider that partial liver resection can be performed effectively with artificial sapphire contact Nd:YAG laser scalpel because of its better outing, coagulation effects, and less tissue damage.

**P353**

### CLINICAL PRESENTATIONS AND DIAGNOSTIC METHODS IN HYDATIDOSIS DISEASES OF THE LIVER

LOINAZ C, G-UREÑA, JIMENEZ C, BERCEDO J, PALMA F, GOMEZ SANZ R, MORENO C, MORENO E.

The experience of our service in diagnoses of the hydatidosis and its clinical presentation is studied.

Since 1974 to 1989 410 patients with 561 cysts were operated. We divided the period of time in two groups : GROUP A: since 1974 to 1984 (322 patients), and GROUP B: since 1985 to 1989 (88 pts). We compared both groups.

Previous surgery for hydatidosis: Lung (A=4.9% B=3.4%), Liver (A=8.6% B=9.1%); Hepatopulmonary (A=0.3% B=82.3%); Multiple (A=0 B+4.5%). Signs and symptoms: Hepatomegalia (A=49.3% B=22.7% p<0.05); Tumor (A=38.1 & B=19.3% p<0.001). Diagnosis: Echograpfy (A=32.2% B=90.9% p 0.01); Liver gammagraphy (A=72.7% B=18.2% p<0.01); CT (A=11.5% B=19.3% p<0.01) Laparoscopy (A=10.8% B=1.1% p<0.05); Arteriography (A=8.4% B=10.3%). The number of cysts were similar in both groups and so was the content of the cysts: biliar (A=25.5% B=19.3%); clear (A=36.1% B=6.6%); Pus contamination (A=18.1% B=10.1% p<0.1); Hematic (A=0.2% B=0.7%); Non specific (A=14% B=34.6%). The need for urgent operation or elective were similar in both groups.

Nowadays there are an increase of the number of patients without symptoms. Ecography and CT are the best diagnostic procedures in the hydatid liver disease.

**P354**

### SPHINCTEROTOMY & SPHINCTEROPLASTY IN COMPLICATED HYDATID DISEASE OF THE LIVER

JIMENEZ C, BERCEDO J, G-URENA M., LOINAZ C., PALMA F., VEGA V., MORENO E.

The surgical treatment in the biliary tract and its complications in patients with hydatid hepatic cysts is studied

69 patients with hepatic hydatidosis are analysed. Sphincteroplasty was made in 56(81.2%) and sphincterotomy in 13(18.8%). The average age was 44.2(16-76). 36 were male and 33female. The indications for one or other technique were choledocholitiasis in 3 (4.4%), biliary fistula 46 (66.6%), hydatidic memebranes inmain biliary duct 25 (36.250 and oedoma of papila 13(18.8%).

Intraoperative mortality was 0%. In the postoperative time 4 patients died (5.8%) from hepatic failure (1), hepatorenal failure (1), and pneumonia (2). Two of them had to be reoperated for subphrenic abcess. There were 25 (36.2%) cases of transient biliary fistula required no surgical management Postoperative complication were: subphrenic abcess 11 (15.9%); intrahepatic abcess 2 (2.9%); pneumonian 2 (2.9%); hepatic failure 1 (1.5%); ascitis 1 (1.5%); erosive gastritis 1 (1.5%).

The sin terotomy and sinteroplasty are useful procedures in the prevention of biliary complications in hydatidic liver disease.

**P355**

### CLINICAL CHARACTERISTICS OF HEPATO-SPLEEN PREGNANCY

ZHANG REN, LI JIN-CHI
Dept. of Surger, The 2nd Central Hospital, Tianjin, China

After consulting all medicine literature in China published from Jan. 1980 to Oct. 1991 we collected 9 reports and our hospital put forward one report. We are going to make following analysis.

Age. 26-30 years 2, 31-39 years 7, 40.48 years 1. Menoschesis. 39-59 days 6, 60-70 days 3, (-) 1 (the form of lithopedion).

Typal acute abdomen. The onset of symptoms is sudden left epigastralgia, sometimes this initial attack of pain may be associated with radiating left omalgia but quickly spreading throughout the whole abdomen.

Acute manifestation of abdominal cavity hemorrhage.

BUS. This is the only one BUS Fig. of spleen pregnancy (Fig.1) Nidus locus. Left lobe of liver 1 case. Spleen 9 cases. Mortality 2 cases. Fig. 2 specimen of spleen pregnancy.

1. the reason can not be diagnosed correctly before the operation. (1) The lack of knowledge about this disease. (2) Not in detail of the medical history. (3) At the onset imminence or in no condition to necessary examination.

2. The diagnosis of hepato-spleen pregnancy depends, in the main, up the recognition of its clinical feature. For such dubiousness patients, quantitive analysis and ultrasongraphy should be routine.

3. The causes of hepato-spleen pregnancy are related to lay up intrauterine device and pelvic inflammation.

**P356**

### TACHOCOMB, A NOVEL COMBINATION OF A COLLAGEN FLEECE WITH FIBRIN GLUE : A NEW APPROACH IN THE RISK MANAGEMENT PARENCHYMAL SURGERY

SAMHABER EM
Triester Strape 50, A-1100 Vienna, Austria

An absorbable wound dressing in a ready-to-use combination of a collagen fleece coated with the components of fibrin glue, i.e. fibrinogen, thrombin and aprotinin, will be presented.

In case of medical emergencies involving splenic or hepatic rupture, immediate hemostasis is often live-saving and can be achieved using a new product in parenchymal surgery: TachoComb.

The TachoComb fleece actively promotes the coagulation process at the wound surface. Complete, fast and reliable hemostasis can be achieved by firmly pressing the coated collagen fleece against would surfaces due to e.g. hepatic and pancreatic resections, cholecystectomies and traumatic ruptures, for 3-5 minutes. Precious time can be saved in parenchymal surgery on liver, spleen, pancreas, lungs and kidneys.

The fleece is subsequently resorbed and converted into connective scar tissue within appr. 3-6 weeks.

TachoComb is an efficient and uniquely disigned product helping the surgeon to minimize risk in parenchymal surgery.

### P357

### TACHOCOMB, A NOVEL COMBINATION OF A COLLAGEN FLEECE WITH FIBRIN GLUE: A NEW APPROACH IN LIVER SURGERY

SAMHABER EM
Triester Strape 50, A-1100 Vienna, Austria

Acombined application of sheets of collagen covered with freshly prepared fibrin glue improved local haemostasis to a great extent. Large areas of capillary bleedings can be treated successfully. But due to the relatively complicated preparation required at the operation site, this method has not been used on a large scale. these drawbacks have been overcome with the latest development in this field - a sheet of collagen covered with a fixed layer of the solid components of fibrin glue (fibrinogen, thrombin and aprotinin).

In a prospective study, 225 cases of liver resections due to metastases (43%), primary liver carcinomas (16%), intraoperational injuries of Glisson's capsule (10%), liver ruptures, benign tumors, bleeding subsequent to punch biopsies, echinococcus cysts and others were included. The assessment of the haemostyptic properties of TachoComb was "very good" and "good" in 95%, "satisfactory" in 4 % and unsatisfactory in 1% of the cases.

In several cases, particularly difficult bleeding situations could be controlled, e.g. those with massive coagulation disturbances or hepatic stasis. No complications attributable to TachoComb occurred.

So far, more than 280 patients with liver resections have been treated successfully with TachoComb in clincal trials. In a liver transplantation study with 27 patients, haemostasis with TachoComb was assessed as "very good" and "good" in 96% of the cases.

### P358

### SUBTOTAL PANCREATECTOMY WITH INTRAOPERATIVE RADIATION THERAPY - PRELIMINARY RESULT

R. BELLANTONE, G.B. DOGLIETTO, D. FRONTERA, A. FERRANTE, G. VIOLA, F. CRUCITTI
Department of Surgery, Catholic University School of Medicine, Rome, Italy

Although few cases have been reported, intraoperative radiation therapy (IORY) seems to be an interesting technique for integrated treatment of pancreatic cancer in assoication with resective surgery.

Recently, our Institution, a protocol for integrated therapy of resectable pancreatic neoplasms has been started. The protocol includes a subtotal pancreatectomy (or total when necessary) with pyloric preservation and a IORT (10 Gy) of the celiac area, the hepatic hilum, the upper mesenteric vessels, the suprarenal vena cava and aorta. The irradiated area does not include the pancreatic stump; the intestinal loops and the right kidney are kept out of the cylinder. External beam radiotherapy is given postoperatively in doses of 50 Gy with a 3-fields technique along 4-5 weeks. Six patients underwent the above described treatment: there were 4 women and 2 men, from the age of 58 to 74 years, all affected by resectable pancreatic adenocarcinoma. The overall average time required for IORT (which includes transport of the patient to the Radiation Therapy Department, selection and placement of the cylinder, irradiation and return to the operatory room) was 54 minutes.

We had no operative mortality. Two patients had an infected abdominal wound adn three cases presented a transient gastroparesis (average 14 days). Symptoms of acute radiation intoxication have not been observed in any patient.

Four patients died because of liver metastases after six, ten, eleven and fifteen months, respectively; the average time before recurrence of disease was 6 months. Twp patients are still alive and free of disease after five and fifteen months. We did not observe, in the CT scan of the abdomen, local recurrence in all cases.

Therefore, we think IORT may be an effective tool to reduce local recurrence rates, although our experience is still limited an further cases are requested to obtain more definitive results.

### P359

### GRADED FLOW RATES THROUGH THE PORTAL-SYSTEMIC BYPASS DURING ACUTE PORTAL OCCLUSION

K. KASAHARA, Y. FUKUMOTO, Y. YASUDA, T. YASUDA, Y. KONDO, H. NAGAI
Department of Surgery, Jichi Medical School, Minamikawachi-machi, Tochigi, 329-04, Japan

Using graded flow rates through portal-systemic bypass, effects of portal congestion per se on splanchnic hemodynamic and metabolic conditions, liver tolerance to interrupted portal flow itself, and critical bypass flow were evaluated in dogs. To exclude the effects caused by reduction in the circulating blood volume, preocclusion levels of cardiac output were maintained constant throughout the study by means of increasing intravenous infusion rate. With decreasing bypass flow rates during portal occlusion, portal pressure increased up to the maximum of some 10% of the basal level without bypass, and hepatic arterial flow increased up to more than two times of the basal level without bypass. In dogs with 50% and 10% bypass during two hours' portal occlusion, there were no remarkable decrease of energy charge and TAN of the liver tissue, and serum transaminase levels remained low postoperatively. However, in dogs with 50% bypass during one hour's hepatic inflow occlusion, there were significant decrease of energy charge and TAN of the liver tissue and remarkable rise of postoperative transaminase levels.

### P360

### IS THERE A PLACE FOR 'SPLIT" PANCREATICOJEJUNOSTOMY IN THE SURGICAL TREATMENT OF CHRONIC PANCREATITIS?

M. MULDER, T. M. VAN GULIK, L.TH.DE WUT, D.J.VAN LEEUWEN, P.C.M. VERBEEK, E.DE JONG, N.J. LYGIDAKIS, M.N.VAN DER HEYDE
Dept. of Surgery, Academic Medical Center, 9 Meibergdreef, 1105 AZ Amsterdam, The Netherlands

In 1967, Marvin James described an operation for the treatment of chronic pancreatitis (CP) which he called "split" pancreaticojejunostomy (PJ). This procedure consisted of a vertical, partial transection of the pancreas with a double anastomosis of the sectioned pancreatic duct to a Roux-en-Y limb of jejunum. We used a modified version of this method for the treatment of CP in which the main pancreatic duct appeared to have a normal caliber. Herein we report the long term results of this method in 8 patients (4 alcoholic CP, 3 familial CP, 1 idiopathic CP). ERCP in these patients showed irregular, but non-dilated main pancreatic ducts, precluding the use of a lateral, side-to-side PJ. The pancreas was totally transected in teh region of the corpus allowing prompt identification of the sectioned pancreatic duct. Both sides of teh duct were anastomosed face to face, to a Roux-en-Y jejunal limb using a mucosa-to-mucosa technic. Double, transanastomotic silicon stents were left during 10 days. Port-operative complications consisted of intra-abdominal abscess (1x) and sepsis/ARDS (1x, in conjuction with a simultaneous cystoduodenostomy. Follow-up was 20-25 months. Clinical outcome was rated good/fair/bad, according to relief of pain. 5 out of 8 patients had a long lasting improvement of symptoms (4 good, 1 fair, 3 bad). From these results we conclude that "split" PJ is a valid alternative for the treatment of CP, when a conventional, lateral PJ is not feasible.

### P361

### FUNCTIONAL RESULT AFTER PYLORUS PRESERVING PANCREATODUODENECTOMY

DR B. RADOVANOVIC, DR N. RADOVANOVIC
Hirursko odeljenje bolnice Pozarevac;
Institut za digestivnu hirurgiju KC Beograd, Yugoslavia

Poster represent functional result of gastric secretion and motion five years after pylorus preserving pancreatoduodenectomy for carcinoma of ampulla Vatery. This case have longest survival between another eight, of which four died, and another four had shorter survival period. The performed operation was standard version recommended by Traverso and Longmire. Complete gastric examination includes standard X-ray, radioactive meal following, endoscopic biopsio. Standard BAO, MAO tests were performed. Weight of the patient was measured regularu, and subjective feeling too.

In conclusion we can say that this type of operation give good functional postoperative results, with minimal patients inconveniences. This results confirm the usage of this method in select cases.

### P362

### PRESERVATION OF THE PORCINE PANCREAS WITH HTK AND EURO-COLLINS SOLUTION, STUDIES IN A REPERFUSION SYSTEM

H.KÖHLER, F.E.LUDTKE, R. NUSTEDE, A. SCHAFMAYER
Dept. of General Surgery University of Geottingen, Gottingen, FRG.

The present study compares the preservation of the porcine pancreas by the standard Euro-Collins solution or the cardioplegic histidine-tryptophan-ketoglutarate solution (HTK). The explanted pancreas was stored at $4^0$ C for 6 and 24 h respectively, following which organ quality was assessed in areperfusion chamber measuring physiological and biomedical parameters. After a 6 h ischaemia, the amount of lactate was significantly lower when HTK was used for protections. Other parameters like insulin release, amylase release, vascular resistance and oxygen consumption of the pancreas did not indicate a significant difference. Protection with HTK significantly improved pancreas preservation after 24 h ischaemia: lactate content in the reperfusate was lower (HTK: 5.7±0.91 ml $min^{-1}$ v.EC: 3.0±0.26ml $min^{-1}$ ), and the pancreatic oxygen consumptions was increased (HTK: 2.15±0.22 ul $O_2$ $min^{-1}g^{-1}$). We conclude that pancreas preservativion can be improved in vitro by protection with HTK solution.

### P363

### TRAUMATIC PANCREATIC PSEUDOCYSTS

GM LEWIS, JEJ KRIGE, PC BORNMAN
Surgical Gastroenterology and Department of Surgery, Unviersity of Cape Town and Groote Schuur Hospital, Cape Town South Africa

Fifteen of 64 patients treated for pancreatic trauma between 1980 and 1989 developed pseudocysts and were evaluated to determine outcome in relation to the nature and site of the pancreatic duct injury. The 13 men, 2 women, had a mean age of 35 (r 24-68) years. 11 had blunt and 4 penetrating (3 gunshot, 1 stab) trauma. Pseudocysts occurred in 8 patients after operation for abdominal trauma and in 7 patients who were treated conservatively. In none was ductal injury diagnosed during the initial management. Median presentation was 20 (mean : 59; range : 8-360) days after injury . In 14 patients pseudocysts (mean diameter: 9 cm; range 3-16mm) were confirmed on CT or US and at laparotomy in 1 patient. The site and nature of the ductal injury was demonstrated in 13 patients using ERCP(n=8), operative pancreatography (n=1) and surgery (n=4). Two patients with peripheral duct injuries on ERCP were successfully treated conservatively. Pseudocysts arising from distal duct injuries (n=4) were treated by percutaneous aspiration or catheter drainage. Duct injuries in the body (n=3) underwent distal pancreatectomy. Proximal duct injury with mature pseudocysts were drained internally. There were no major complications. In 3 patients complications of the pseudocyst (haemorrhage : 2; sepsis: 1) necessitated emergency laparotomy and external drainage of whom 1 died of sepsis.

On the basis of this study, we recommend that pseudocysts that follow peripheral pancreatic duct injuries can be treated conservatively, distal injuries by percutaneous aspiration or drainage, but proximal injuries require surgery.

### P364

### MANAGEMENT OF PANCREATIC TRAUMA

T. YAMAMOTO, T.SEKOGUCHI, Y. KATSUMINE, S. INAMORI
Department of Surgery, Ise Municipal Hospital
Ise-city, Mie, 516, Japan

During the past 10 years, we treated 10 patients with pancreatic trauma. Cause of pancreatic trauma was traffic accident in 8, and other kinds of accidents in 2. Severe abdominal pain and increase of serum amylase level were observed in all patients, nausea in 4, and hemorrhagic shock in one. CT scan revealed pancreatic swelling or peripancreatic fluid collection in all patients. Of 6 patients who underwent conservative treatment, in 3 pancreatitis healed uneventfully and in the other 3 pseudocyst developed.

Spontaneous resolution was observed in one patient with pseudocyst, and cystgastrostomy or cyst resection was performed in the other two. Emergency surgery had been performed in 2 patients; pancreaticoduodenectomy (PD) was performed in one with extensive crush injury around the pancreas head, and primary suture of the pancreatic parenchyma with duct stenting in the other, who had complete disruption of the pancreatic body. The remaining 2 patients had undergone drainage at another hospital and were referred to our hospital because of external pancreatic fistula and pyloric stenosis; distal pancreatectomy and partial gastrectomy was carried out in both. Favourable outcome was obtained in all paients except one who had undergone PD, who died of MOF followed by anastomotic leakage.

In conclusion, conservative treatment in conjunction with serial CT scan is appropriate choice of treatment for most patients with pancreatic trauma. However, patients with crush injury or complete disruption of the pancreas require emergency surgery.

### P365

### THE MANAGEMENT OF PANCREATIC INJURIES

D.M.K.S. KAULESAR SUKUL, E.J. JOHANNES, H.E. LONT
Department of Traumatology/General Surgery, University Hospital Rotterdam, Dijkzigt, City, Rotterdam, The Netherlands.

To contribute to accurate diagnostics and treatment of patients with pancreatic injuries, we reviewed the medical records of all patients, who were treated in our hospital, for traumatic pancreatic lesions in the period of 1971 to 1987. Off all twenty-four male patients the mechanism of the injury, the diagnostic methods, the associated injuries, the location of the pancreatic injury, the presence of pancreatic duct lesion, the treatment and the final results and complications are described. The high mortality rate of patients with pancreatic trauma, is in our opinion not due to the pancreatic injury per se, but due to the severe concomitant injuries resulting from a high energy trauma. Based on the literature and our own experience we designed an algorithm for the management of pancreatic injuries. If anamnestic and physical examination indicate that intra-abdominal injury might be present, roentgenorgraphic and ultrasound investigation are the diagnostic methods of choice. Patients without pancreatic duct lesion can be treated with debridement and external suction drainage. If pancreatic duct lesion is present and is located to the right of the superior mesenteric vessels, treatment should consist of partial pancreatic resection and pancreatico-jejunostomy or a Whipple procedure. If the pancreatic duct lesion is located to the left of the superior mesenteric vessels, treatment should consist of distal pancreatectomy and splenectomy with pancreatico-jejunostomy.

### P366

### SURGICAL TREATMENT OF SEVERE PANCREATIC TRAMA

QIAN GUANG-XIAN
Institue of Hepatobiliary Surgery, Changhai Hospital, Shanghai, P R China

Pancreatic trauma constitutes one of the most serious abdominal trauma. It carries a mortality of 20 %, or even as high as40 - 50% when associated with duodenal perforation. A series of complications may follow. Its treatment is rather difficult. Experiences in the management of 21 cases of severe pancreatic trauma (3 penetrating and 18 blunt injures) were analyzed. Associated intra-abdominal injures existed in all patients including duodenal perforation (6 cases) and colon perforation (5 cases). Patients could be classified into 4 groups: mild contusion (1 case), serious lacerative contusion (11 cases), destructive injury (3 cases), combined pancreato-duodenal injury (6 cases). The following operations had been performed: pancreaticoduodenectomy (2 cases) distal pancreato-jejunostomy (3 cases), pancreatorrhaphy (7 cases). There was no mortality in this series. According to the location, severity and with or without duodenal perforation, the classification and diagnosis were discussed. Elevation of amylase in blood and abdominal cavity fluid, B-US, CT and ERP were helpful in diagnosing pancreatic trauma. Miss in diagnosis of pancreatic and duodenal trauma during laparotomy must be avoided. Appropriate operative procedure, fully effective intraabdominal drainage and a good nutritional support were the primary importance in treating pancreatic trauma and preventing complications.

### P367

### PHARMACOKINETICS OF CIPROFLOXACIN IN PANCREATIC JUICE AFTER PANCREATIC SURGERY

I. BACA, T. BOMMER, H.G. SCHAFER, I. KLEMPA

Allgemein - und Gefäßchirurgische Klinik, ZKH St. - Jürgen-straße, 2800 Bremen, Germany

Ciprofloxacin has remarkably good activity against many microorganism known to cause pancreatic infection. The aim of this study was to establish the penetration of ciprofloxacin into pure human pancreatic juice after pancreatic surgery.

In a prospective trial the pentration of ciprofloxacin into pancreatic juice following a single parenteral dose was investigated in 10 patients who had undergone pancreatektomy (whipple procedure) for cancer. A small catheter, which was placed into the duct of pancreatic remnant gave access to the pancreatic juice. Blood and pancreatic juice samples were taken prior to the administration of the day and then 0.5, 1.5, 2, 3, 4, 5, 6.5, 8 h after drug intake. The samples were frozen and stored at -20 C until examination. The concentration of ciprofloxacin in blood and in pancreatic juice as well as amylase, lipase and protein were analysed. Following a single i.v. dose of 200 mg of ciprofloxacin peak serum concentration were 3.2± 0.6 mg/1(M±SEM). The mean half live was 5 h. The mean pancreatic juice/serum concentration ratio were 0.61.

The concentration of ciprofloxacin achieve the-rapeutic concentration for most of organism cousin infection in pancreas after an i.v. dose.

### P368

### OCTREOTIDE AND PANCREATIC FISTULA

G. CAMPANELLI, P. CAMPAGNOLI, M. ROVATI, E. LEOPALDI
Clinica Chirurgica I, University of Mila, Luigi Sacco Hospital, Italy

In 1973 Brazeu and coll. succeeded in identifying and extracting from ovine ipotalamus a peptide endowed with an inhibitory action on GH secretion, called somatostatina. The somatostatina spreads throughout the organism, particularly in the ipotalamus. The main biological actions of somatostatina are represented by an inhibitory function of some organs and apparatus: hypophysis, alimentary tract. Besides it should inhibit the gastroduodenal motility including the one relative to the biliopancreatic secretion. Ovviously the similar synthetics of the somatostatina revealed their great utility in regulating hormonal secretions and the proliferation phenomena in many pathologies. Actually an important limitation to the therapeutic use of somatostatina depends on the short life of its effects that condition its high and continuous administration. The elaboration of a synthetic preparation similar to somatostatina as octapeptide has allowed the use of a more lasting and equally active molecule in vivo, the octreotide, that allows the utilization of this molecule for long therapies. Among other advantages octreotide can be used as a subcutaneous injections. Considering these pharmacologic acquisition and the clinic experience found in literature, the Authors have treated two cases of enterocutaneous fistula, complications of operations on the digestive tract, with octreotide (LONGASTATINA). In this work, the Authors present the therapeutic course essentially based on the total parenteral nutrition (TPN) and one the employment of Longastatina. In both cases, in the course of four months the clinic status cleard up.

**P369**

### GAMMA LINOLENIC ACID POTENTIATES TRANSFORMING GROWTH FACTOR BETA INHIBITION OF EPITHELIAL CELL PROLIFERATION

WG JIANG and MCA PUNTIS
University Department of Surgery, University of Wales College of Medicine, Cardiff, U.K.

Transforming growth factor beta (TGFβ) is a growth inhibitor for epithelial & some malignant cells. We studied the effects of gamma linolenic acid (GLA) on TGFβ inhibition of epithelial cell. A epithelial cell line: mv 1 lu (CCL64) was used in the study. Cells were treated with either TGFβ alone or in combination with GLA at different concentrations. After 3 days of culture cells were quantified. The inhibition is shown as median numbers of remaining cells as a percentage of its own control, statistics is Mann-Whitney U test. (RU=International Reference Unit)

| | TGF10RU | TGF2.5RU | TGF0.6RU | TGF0.3RU |
|---|---|---|---|---|
| Medium | 61.0% | 74.5% | 86.3% | 93.6% |
| GLA(25μM) | 65.3% | 73.9% | 91.3% | 98.2% |
| GLA (50μM) | 49.7%* | 61.6%# | 76.7%# | 89.5% |
| GLA (100μM) | 44.7%* | 55.6%* | 73.4%* | 85.2% |
| GLA (150μM) | 34.9%* | 43.7%* | 58.3%* | 77.9%* |
| GLA (200μM) | 22.4%* | 34.4%* | 50.6%* | 73.8%* |

#p<0.05 *p=0.01 against control

GLA from 25-200 μM increased TGF inhibition of epithelial proliferation and this was in a dose dependent manner (r>0.96 for the concentrations tested, p<0.01).

Concludsion: Gamma linolenic acid potentiates transforming growth factor inhibition of epithelial proliferation. This suggest that GLA has a role in the control of the growth of epithelial cells which may have therapeutic implications.

**P370**

### HISTOPATHOLOGICAL AND IMMUNOHISTOLOGICAL STUDY OF ACINAR CELL NODULES IN HUMAN PANCREAS

K. SAKAMOTO, T. INAYOSHI, S. SHIOMI, T. KAMANO, T. MAEKAWA, N. SAKAKIBARA,
Juntendo University School of Medicine, First Department of Surgery, 2-1-1 HongoBunkyoku Tokyo, 113, Japan.

To explicate a role of acinar cell nodules(ACN) in human pancreas, specimens of surgically-excised pancreatic tissue were examined histopathologically and immunohistochemically in this study. The paraffin sections derived from 65 pancreatectomy cases were examined. The ACN was defined well circumscribed from the surrounding acini cells by HE stain. ACN-constituting cells were examined in histopathological study. Furthermore anti-human amylase antibody and anti-human trypsin antibody were used in immunohistochemical study.

Among examined series of sections, 62 ACN were found in 18 cases. ACN-constituting cells were compared with the surrounding acini cells, but no data indicated atypia of ACN cells in histopathological study. By anti-lipase staining, 14 ACN were positive, 37 ACN showed no tonal difference from surrounding acini cells, and the remaining 11 ACN were negative. By anti-amylase staining, 3 ACN were positive, 11 ACN showed no tonal difference, and the remaining 48 ACN were negative. By anti-trypsin staining, 7 ACN were positive, 45 ACN showed no tonal difference, and the remaining 10 ACN were negative. It is suggested that ACN are not pathological changes leading to pancreatic acinar cell carcinoma, but are possibly yielded as degenerative products which might be reveresible. It was revealed that the cells of ACN are functional nodules in which a certain pancreatic exocrine activity have been specifically decreased.

**P371**

### THE SURGICAL TREATMENT OF INSULINOMA AND LONG TERM FOLLOW UP

XIE WEN-JIN AND LI CHI-CHENG
Guangdong Provincial People's Hospital
Guangzhou 510080, China

The Surgical treatment of 15 cases of insulinoma from 1972 to 1990 is reported. 8 male, 7 female; aged 25 - 56. 33 insulinoma were resected and all proved to be benign in nature pathologically. 14 patients recovered and one died due to the complication of acute pancreatitis after operation. 13 of them (92.8%) had been followed up from 4 months to 19 years after operation, average 7 years. The symptom of hypoglycaemia disappeared, and blood guscose test was normal in all 13 cases. These 13 patients now run a normal life. Our experience indicates that the surgery is the most effective treatment of insulinoma. The presence and location of the tumour larger than 1 cm in diameter can be detected by selective celiac angiography accurately in all 5 of our cases. Probing the pancreas with care during operation should be emphasized, thus the insulinoma can be detected and resected. The determination of the blood glucose level during operation has practical significance in judging a thorough resection of insulinoma. It is usually difficult to detect the minute adenomas which often constitute the major cause of the failure of a surgical treatment. Patient having relapse of symptoms after the surgery may be operated again. In our series, one patient with recurrence eventually recovered after second operation.

**P372**

### PARTIAL DISTAL PANCREATECTOMY WITH Nd:YAG LASER IN THE DOG : AN EXPERIMENTAL STUDY

XU MIN, WU ZAI-DE, CHEN XIO- PING
Department of General Surgery, Tongji Hospital, Tonji Medical University, Wuhan, Hubei (430030), P.R. China

This study reported that partial distal Pancreatectomy was performed in dogs with Nd:YAG laser. Results were compared with that obtained with use of scalpel and sutures. The cuting edge of the pancreatic tail was coagulated effectively until hemostasis was achieved. The Nd:YAG laser can cut pancreatic tail quickly and effectively. Postoperative normal amylasemia and blood suger appeared in all the animals. Histological study of laser groups showed thermal injury at the cuting section with damage to the nearby parenchyma. Pancreatography showed a sealed pancreatic duct in laser groups although no duct ligation was perfored.

